# Abstracts from the 3rd International Genomic Medicine Conference (3rd IGMC 2015): Jeddah, Kingdom of Saudi Arabia. 30 November - 3 December 2015

**DOI:** 10.1186/s12864-016-2858-0

**Published:** 2016-07-20

**Authors:** Jerry W. Shay, Noriko Homma, Ruyun Zhou, Muhammad Imran Naseer, Adeel G. Chaudhary, Mohammed Al-Qahtani, Nobutaka Hirokawa, Maryam Goudarzi, Albert J. Fornace, Saleh Baeesa, Deema Hussain, Mohammed Bangash, Fahad Alghamdi, Hans-Juergen Schulten, Angel Carracedo, Ishaq Khan, Hanadi Qashqari, Nawal Madkhali, Mohamad Saka, Kulvinder S. Saini, Awatif Jamal, Jaudah Al-Maghrabi, Adel Abuzenadah, Adeel Chaudhary, Mohammed Al Qahtani, Ghazi Damanhouri, Heba Alkhatabi, Anne Goodeve, Laura Crookes, Nikolas Niksic, Nicholas Beauchamp, Adel M. Abuzenadah, Jim Vaught, Bruce Budowle, Mourad Assidi, Abdelbaset Buhmeida, Jaudah Al-Maghrabi, Abdelbaset Buhmeida, Mourad Assidi, Leena Merdad, Sudhir Kumar, Sayaka Miura, Karen Gomez, Angel Carracedo, Mahmood Rasool, Ahmed Rebai, Sajjad Karim, Hend F. Nour Eldin, Heba Abusamra, Elham M. Alhathli, Nada Salem, Mohammed H. Al-Qahtani, Sudhir Kumar, Hossam Faheem, Ashok Agarwa, Eberhard Nieschlag, Joachim Wistuba, Oliver S. Damm, Mohd A. Beg, Taha A. Abdel-Meguid, Hisham A. Mosli, Osama S. Bajouh, Adel M. Abuzenadah, Mohammed H. Al-Qahtani, Serdar Coskun, Muhammad Abu-Elmagd, Abdelbaset Buhmeida, Ashraf Dallol, Jaudah Al-Maghrabi, Sahar Hakamy, Wejdan Al-Qahtani, Asia Al-Harbi, Shireen Hussain, Mourad Assidi, Mohammed Al-Qahtani, Adel Abuzenadah, Burak Ozkosem, Rick DuBois, Safia S. Messaoudi, Maryam T. Dandana, Touhami Mahjoub, Wassim Y. Almawi, S. Abdalla, M. Nabil Al-Aama, Asmaa Elzawahry, Tsuyoshi Takahashi, Sachiyo Mimaki, Eisaku Furukawa, Rie Nakatsuka, Isao Kurosaka, Takahiko Nishigaki, Hiromi Nakamura, Satoshi Serada, Tetsuji Naka, Seiichi Hirota, Tatsuhiro Shibata, Katsuya Tsuchihara, Toshirou Nishida, Mamoru Kato, Sajid Mehmood, Naeem Mahmood Ashraf, Awais Asif, Muhammad Bilal, Malik Siddique Mehmood, Aadil Hussain, Qazi Mohammad Sajid Jamal, Mughees Uddin Siddiqui, Mohammad A. Alzohairy, Mohammad A. Al Karaawi, Taoufik Nedjadi, Jaudah Al-Maghrabi, Mourad Assidi, Heba Al-Khattabi, Adel Al-Ammari, Ahmed Al-Sayyad, Abdelbaset Buhmeida, Mohammed Al-Qahtani, Hédia Zitouni, Nozha Raguema, Marwa Ben Ali, Wided Malah, Raja Lfalah, Wassim Almawi, Touhami Mahjoub, Mohammed Elanbari, Andrey Ptitsyn, Sana Mahjoub, Rabeb El Ghali, Bechir Achour, Nidhal Ben Amor, Mourad Assidi, Brahim N’siri, Hamid Morjani, Taoufik Nedjadi, Adel Al-Ammari, Ahmed Al-Sayyad, Nada Salem, Esam Azhar, Jaudah Al-Maghrabi, Vera Chayeb, Maryam Dendena, Hedia Zitouni, Khedija Zouari-Limayem, Touhami Mahjoub, Bassem Refaat, Ahmed M. Ashshi, Sarah A. Batwa, Hazem Ramadan, Amal Awad, Ahmed Ateya, Adel Galal Ahmed El-Shemi, Ahmad Ashshi, Mohammed Basalamah, Youjin Na, Chae-Ok Yun, Adel Galal Ahmed El-Shemi, Ahmad Ashshi, Mohammed Basalamah, Youjin Na, Chae-Ok Yun, Adel Galal El-Shemi, Bassem Refaat, Osama Kensara, Amr Abdelfattah, Batol Imran Dheeb, Mohammed M. F. Al-Halbosiy, Rghad Kadhim Al lihabi, Basim Mohammed Khashman, Djouhri Laiche, Chaudhary Adeel, Nedjadi Taoufik, Hani Al-Afghani, Maria Łastowska, Haya H. Al-Balool, Harsh Sheth, Emma Mercer, Jonathan M. Coxhead, Chris P. F. Redfern, Heiko Peters, Alastair D. Burt, Mauro Santibanez-Koref, Chris M. Bacon, Louis Chesler, Alistair G. Rust, David J. Adams, Daniel Williamson, Steven C. Clifford, Michael S. Jackson, Mala Singh, Mohmmad Shoab Mansuri, Shahnawaz D. Jadeja, Hima Patel, Yogesh S. Marfatia, Rasheedunnisa Begum, Amal M. Mohamed, Alaa K. Kamel, Nivin A. Helmy, Sayda A. Hammad, Hesham F. Kayed, Marwa I. Shehab, Assad El Gerzawy, Maha M. Ead, Ola M. Ead, Mona Mekkawy, Innas Mazen, Mona El-Ruby, S. M. A. Shahid, Qazi Mohammad Sajid Jamal, J. M. Arif, Mohtashim Lohani, Moumni Imen, Chaouch Leila, Ouragini Houyem, Douzi Kais, Chaouachi Dorra Mellouli Fethi, Bejaoui Mohamed, Abbes Salem, Areeg Faggad, Amanuel T. Gebreslasie, Hani Y. Zaki, Badreldin E. Abdalla, Maha S. AlShammari, Rhaya Al-Ali, Nader Al-Balawi, Mansour Al-Enazi, Ali Al-Muraikhi, Fadi Busaleh, Ali Al-Sahwan, Francis Borgio, Abdulazeez Sayyed, Amein Al-Ali, Sadananda Acharya, Maha S. Zaki, Hala T. El-Bassyouni, Marwa I. Shehab, Mohammed F. Elshal, Kaleemuddin M., Alia M. Aldahlawi, Omar Saadah, J. Philip McCoy, Adel E. El-Tarras, Nabil S. Awad, Abdulla A. Alharthi, Mohamed M. M. Ibrahim, Haneen S. Alsehli, Ashraf Dallol, Abdullah M. Gari, Mohammed M. Abbas, Roaa A. Kadam, Mazen M. Gari, Mohmmed H. Alkaff, Adel M. Abuzenadah, Mamdooh A. Gari, Heba Abusamra, Sajjad Karim, Hend F. Nour eldin, Elham M. Alhathli, Nada Salem, Sudhir Kumar, Mohammed H. Al-Qahtani, Fatima A. Moradi, Omran M. Rashidi, Zuhier A. Awan, Ibrahim Hamza Kaya, Olfat Al-Harazi, Dilek Colak, Nabila A. Alkousi, Takis Athanasopoulos, Afnan O. Bahmaid, Etimad A. Alhwait, Mamdooh A. Gari, Haneen S. Alsehli, Mohammed M. Abbas, Mohammed H. Alkaf, Roaa Kadam, Ashraf Dallol, Gauthaman Kalamegam, Hend F. Nour Eldin, Sajjad Karim, Heba Abusamra, Elham Alhathli, Nada Salem, Mohammed H. Al-Qahtani, Sudhir Kumar, Salma N. Alsayed, Fawziah H. Aljohani, Samaher M. Habeeb, Rawan A. Almashali, Sulman Basit, Samia M. Ahmed, Rakesh Sharma, Ashok Agarwal, Damayanthi Durairajanayagam, Luna Samanta, Muhammad Abu-Elmagd, Adel M. Abuzenadah, Edmund S. Sabanegh, Mourad Assidi, Mohammed Al-Qahtani, Ashok Agarwal, Rakesh Sharma, Luna Samanta, Damayanthi Durairajanayagam, Mourad Assidi, Muhammad Abu-Elmagd, Mohammed Al-Qahtani, Adel M. Abuzenadah, Edmund S. Sabanegh, Luna Samanta, Ashok Agarwal, Rakesh Sharma, Zhihong Cui, Mourad Assidi, Adel M. Abuzenadah, Muhammad Abu-Elmagd, Mohammed Al-Qahtani, Alaa A. Alboogmi, Nuha A. Alansari, Maha M. Al-Quaiti, Fai T. Ashgan, Afnan Bandah, Hasan S. Jamal, Abdullraheem Rozi, Zeenat Mirza, Adel M. Abuzenadah, Sajjad Karim, Mohammed H. Al-Qahtani, Sajjad Karim, Hans-Juergen Schulten, Ahmad J. Al Sayyad, Hasan M. A. Farsi, Jaudah A. Al-Maghrabi, Zeenat Mirza, Reem Alotibi, Alaa Al-Ahmadi, Nuha A. Alansari, Alaa A. Albogmi, Maha M. Al-Quaiti, Fai T. Ashgan, Afnan Bandah, Mohammed H. Al-Qahtani, Rasha A. Ebiya, Samia M. Darwish, Metwally M. Montaser, Heba Abusamra, Vladimir B. Bajic, Jaudah Al-Maghrabi, Wafaey Gomaa, Mehenaz Hanbazazh, Mahmoud Al-Ahwal, Asia Al-Harbi, Wejdan Al-Qahtani, Saher Hakamy, Ghali Baba, Abdelbaset Buhmeida, Mohammed Al-Qahtani, Jaudah Al-Maghrabi, Abdullah Al-Harbi, Mahmoud Al-Ahwal, Asia Al-Harbi, Wejdan Al-Qahtani, Sahar Hakamy, Ghalia Baba, Abdelbaset Buhmeida, Mohammed Al-Qahtani, Elham M. Alhathli, Sajjad Karim, Nada Salem, Hend Nour Eldin, Heba Abusamra, Sudhir Kumar, Mohammed H. Al-Qahtani, Aisha A. Alyamani, Gauthaman Kalamegam, Etimad A. Alhwait, Mamdooh A. Gari, Mohammed M. Abbas, Mohammed H. Alkaf, Haneen S. Alsehli, Roaa A. Kadam, Mohammed Al-Qahtani, Rawan Gadi, Abdelbaset Buhmeida, Mourad Assidi, Adeel Chaudhary, Leena Merdad, Saadiah M. Alfakeeh, Etimad A. Alhwait, Mamdooh A. Gari, Mohammed M. Abbas, Mohammed H. Alkaf, Haneen S. Alsehli, Roaa Kadam, Gauthaman Kalamegam, Rubi Ghazala, Shilu Mathew, M. Haroon Hamed, Mourad Assidi, Mohammed Al-Qahtani, Ishtiaq Qadri, Shilu Mathew, Lobna Mira, Manal Shaabad, Shireen Hussain, Mourad Assidi, Muhammad Abu-Elmagd, Mohammed Al-Qahtani, Shilu Mathew, Manal Shaabad, Lobna Mira, Shireen Hussain, Mourad Assidi, Muhammad Abu-Elmagd, Mohammed Al-Qahtani, Ahmed Rebai, Mourad Assidi, Abdelbaset Buhmeida, Muhammad Abu-Elmagd, Ashraf Dallol, Jerry W. Shay, Mikhlid H. Almutairi, Angie Ambers, Jennifer Churchill, Jonathan King, Monika Stoljarova, Harrell Gill-King, Mourad Assidi, Muhammad Abu-Elmagd, Abdelbaset Buhmeida, Muhammad Al-Qatani, Bruce Budowle, Muhammad Abu-Elmagd, Farid Ahmed, Ashraf Dallol, Mourad Assidi, Taha Abo Almagd, Sahar Hakamy, Ashok Agarwal, Muhammad Al-Qahtani, Adel Abuzenadah, Sajjad Karim, Hans-Juergen Schulten, Ahmad J. Al Sayyad, Hasan M. A. Farsi, Jaudah A. Al-Maghrabi, Abdelbaset Buhmaida, Zeenat Mirza, Reem Alotibi, Alaa Al-Ahmadi, Nuha A. Alansari, Alaa A. Albogmi, Maha M. Al-Quaiti, Fai T. Ashgan, Afnan Bandah, Mohammed H. Al-Qahtani, Rukhsana Satar, Mahmood Rasool, Waseem Ahmad, Nazia Nazam, Mohamad I. Lone, Muhammad I. Naseer, Mohammad S. Jamal, Syed K. Zaidi, Peter N. Pushparaj, Mohammad A. Jafri, Shakeel A. Ansari, Mohammed H. Alqahtani, Hanan Bashier, Abrar Al Qahtani, Shilu Mathew, Amal M. Nour, Heba Alkhatabi, Adel M. Abu Zenadah, Abdelbaset Buhmeida, Mourad Assidi, Muhammed Al Qahtani, Muhammad Faheem, Shilu Mathew, Shiny Mathew, Peter Natesan Pushparaj, Mohammad H. Al-Qahtani, Hani A. Alhadrami, Ashraf Dallol, Adel Abuzenadah, Ibtessam R. Hussein, Adeel G. Chaudhary, Rima S. Bader, Randa Bassiouni, Maha Alquaiti, Fai Ashgan, Hans Schulten, Mohamed Nabil Alama, Mohammad H. Al Qahtani, Mohammad I. Lone, Nazia Nizam, Waseem Ahmad, Mohammad A. Jafri, Mahmood Rasool, Shakeel A. Ansari, Muhammed H. Al-Qahtani, Eradah Alshihri, Muhammad Abu-Elmagd, Lina Alharbi, Mourad Assidi, Mohammed Al-Qahtani, Shilu Mathew, Peter Pushparaj Natesan, Muhammed Al Qahtani, Gauthaman Kalamegam, Peter Natesan Pushparaj, Fazal Khan, Roaa Kadam, Farid Ahmed, Mourad Assidi, Khalid Hussain Wali Sait, Nisreen Anfinan, Mohammed Al Qahtani, Muhammad I. Naseer, Adeel G. Chaudhary, Mohammad S. Jamal, Shilu Mathew, Lobna S. Mira, Peter N. Pushparaj, Shakeel A. Ansari, Mahmood Rasool, Mohammed H. AlQahtani, Muhammad I. Naseer, Adeel G. Chaudhary, Shilu Mathew, Lobna S. Mira, Mohammad S. Jamal, Sameera Sogaty, Randa I. Bassiouni, Mahmood Rasool, Mohammed H. AlQahtani, Mahmood Rasool, Shakeel A. Ansari, Mohammad S. Jamal, Peter N. Pushparaj, Abdulrahman M. S. Sibiani, Waseem Ahmad, Abdelbaset Buhmeida, Mohammad A. Jafri, Mohiuddin K. Warsi, Muhammad I. Naseer, Mohammed H. Al-Qahtani, Rubi, Kundan Kumar, Ahmad A. T. Naqvi, Faizan Ahmad, Md I. Hassan, Mohammad S. Jamal, Mahmood Rasool, Mohammed H. AlQahtani, Ashraf Ali, Jummanah Jarullah, Mahmood Rasool, Abdelbasit Buhmeida, Shahida Khan, Ghufrana Abdussami, Maryam Mahfooz, Mohammad A. Kamal, Ghazi A. Damanhouri, Mohammad S. Jamal, Bushra Jarullah, Jummanah Jarullah, Mohammad S. S. Jarullah, Ashraf Ali, Mahmood Rasool, Mohammad S. Jamal, Mourad Assidi, Muhammad Abu-Elmagd, Osama Bajouh, Peter Natesan Pushparaj, Mohammed Al-Qahtani, Adel Abuzenadah, Mohammad S. Jamal, Jummanah Jarullah, Abdulah E. A. Mathkoor, Hashim M. A. Alsalmi, Anas M. M. Oun, Ghazi A. Damanhauri, Mahmood Rasool, Mohammed H. AlQahtani, Muhammad I. Naseer, Mahmood Rasool, Sameera Sogaty, Adeel G. Chudhary, Yousif A. Abutalib, Daniele Merico, Susan Walker, Christian R. Marshall, Mehdi Zarrei, Stephen W. Scherer, Mohammad H. Al-Qahtani, Muhammad I. Naseer, Muhammad Faheem, Adeel G. Chaudhary, Mahmood Rasool, Gauthaman Kalamegam, Fai Talal Ashgan, Mourad Assidi, Farid Ahmed, Syed Kashif Zaidi, Mohammed M. Jan, Mohammad H. Al-Qahtani, Maryam Al-Zahrani, Sahira Lary, Sahar Hakamy, Ashraf Dallol, Mahmoud Al-Ahwal, Jaudah Al-Maghrabi, Emmanuel Dermitzakis, Adel Abuzenadah, Abdelbaset Buhmeida, Mohammed Al-Qahtani, Abeer A. Al-refai, Mona Saleh, Rehab I. Yassien, Mahmmoud Kamel, Rabab M. Habeb, Najlaa Filimban, Ashraf Dallol, Nadia Ghannam, Mohammed Al-Qahtani, Adel Mohammed Abuzenadah, Fehmida Bibi, Sana Akhtar, Esam I. Azhar, Muhammad Yasir, Muhammad I. Nasser, Asif A. Jiman-Fatani, Ali Sawan, Ruaa A. Lahzah, Asho Ali, Syed A. Hassan, Seyed E. Hasnain, Iftikhar A. Tayubi, Hamza A. Abujabal, Alaa O. Magrabi, Fazal Khan, Gauthaman Kalamegam, Peter Natesan Pushparaj, Adel Abuzenada, Taha Abduallah Kumosani, Elie Barbour, Mohammed Al-Qahtani, Manal Shabaad, Shilu Mathew, Ashraf Dallol, Adnan Merdad, Abdelbaset Buhmeida, Mohammed Al-Qahtani, Mourad Assidi, Muhammad Abu-Elmagd, Kalamegam Gauthaman, Mamdooh Gari, Adeel Chaudhary, Adel Abuzenadah, Peter Natesan Pushparaj, Mohammed Al-Qahtani, Syed A. Hassan, Iftikhar A. Tayubi, Hani M. A. Aljahdali, Reham Al Nono, Mamdooh Gari, Haneen Alsehli, Farid Ahmed, Mohammed Abbas, Gauthaman Kalamegam, Mohammed Al-Qahtani, Shilu Mathew, Fazal Khan, Mahmood Rasool, Mohammed Sarwar Jamal, Muhammad Imran Naseer, Zeenat Mirza, Sajjad Karim, Shakeel Ansari, Mourad Assidi, Gauthaman Kalamegam, Mamdooh Gari, Adeel Chaudhary, Adel Abuzenadah, Peter Natesan Pushparaj, Mohammed Al-Qahtani, Muhammad Abu-Elmagd, Gauthaman Kalamegam, Roaa Kadam, Mansour A. Alghamdi, Magdy Shamy, Max Costa, Mamdouh I. Khoder, Mourad Assidi, Peter Natesan Pushparaj, Mamdooh Gari, Mohammed Al-Qahtani, Najla Kharrat, Sabrine Belmabrouk, Rania Abdelhedi, Riadh Benmarzoug, Mourad Assidi, Mohammed H. Al Qahtani, Ahmed Rebai, Ghazi Dhamanhouri, Peter Natesan Pushparaj, Abdelwahab Noorwali, Mohammad Khalid Alwasiyah, Afnan Bahamaid, Saadiah Alfakeeh, Aisha Alyamani, Haneen Alsehli, Mohammed Abbas, Mamdooh Gari, Ali Mobasheri, Gauthaman Kalamegam, Mohammed Al-Qahtani, Muhammad Faheem, Shilu Mathew, Peter Natesan Pushparaj, Mohammad H. Al-Qahtani, Shilu Mathew, Muhammad Faheem, Shiny Mathew, Peter Natesan Pushparaj, Mohammad H. Al-Qahtani, Mohammad Sarwar Jamal, Syed Kashif Zaidi, Raziuddin Khan, Kanchan Bhatia, Mohammed H. Al-Qahtani, Saif Ahmad, Iftikhar AslamTayubi, Manish Tripathi, Syed Asif Hassan, Rahul Shrivastava, Iftikhar A. Tayubi, Syed Hassan, Hamza A. S. Abujabal, Ishani Shah, Bushra Jarullah, Mohammad S. Jamal, Jummanah Jarullah, Ishfaq A. Sheikh, Ejaz Ahmad, Mohammad S. Jamal, Mohd Rehan, Muhammad Abu-Elmagd, Iftikhar A. Tayubi, Samera F. AlBasri, Osama S. Bajouh, Rola F. Turki, Adel M. Abuzenadah, Ghazi A. Damanhouri, Mohd A. Beg, Mohammed Al-Qahtani, Sahar A. F. Hammoudah, Khalid M. AlHarbi, Lama M. El-Attar, Ahmed M. Z. Darwish, Sara M. Ibrahim, Ashraf Dallol, Hani Choudhry, Adel Abuzenadah, Jalaludden Awlia, Adeel Chaudhary, Farid Ahmed, Mohammed Al-Qahtani, Mohammad A. Jafri, Muhammad Abu-Elmagd, Mourad Assidi, Mohammed Al-Qahtani, Imran khan, Muhammad Yasir, Esam I. Azhar, Sameera Al-basri, Elie Barbour, Taha Kumosani, Fazal Khan, Gauthaman Kalamegam, Peter Natesan Pushparaj, Adel Abuzenada, Taha Abduallah Kumosani, Elie Barbour, Heba M. EL Sayed, Eman A. Hafez, Hans-Juergen Schulten, Aisha Hassan Elaimi, Ibtessam R. Hussein, Randa Ibrahim Bassiouni, Mohammad Khalid Alwasiyah, Richard F. Wintle, Adeel Chaudhary, Stephen W. Scherer, Mohammed Al-Qahtani, Zeenat Mirza, Vikram Gopalakrishna Pillai, Sajjad Karim, Sujata Sharma, Punit Kaur, Alagiri Srinivasan, Tej P. Singh, Mohammed Al-Qahtani, Reem Alotibi, Alaa Al-Ahmadi, Fatima Al-Adwani, Deema Hussein, Sajjad Karim, Mona Al-Sharif, Awatif Jamal, Fahad Al-Ghamdi, Jaudah Al-Maghrabi, Saleh S. Baeesa, Mohammed Bangash, Adeel Chaudhary, Hans-Juergen Schulten, Mohammed Al-Qahtani, Muhammad Faheem, Peter Natesan Pushparaj, Shilu Mathew, Taha Abdullah Kumosani, Gauthaman Kalamegam, Mohammed Al-Qahtani, Faisal A. Al-Allaf, Zainularifeen Abduljaleel, Abdullah Alashwal, Mohiuddin M. Taher, Abdellatif Bouazzaoui, Halah Abalkhail, Faisal A. Ba-Hammam, Mohammad Athar, Gauthaman Kalamegam, Peter Natesan Pushparaj, Muhammad Abu-Elmagd, Farid Ahmed, Khalid HussainWali Sait, Nisreen Anfinan, Mamdooh Gari, Adeel Chaudhary, Adel Abuzenadah, Mourad Assidi, Mohammed Al-Qahtani, Naira Ben Mami, Yosr Z. Haffani, Mouna Medhioub, Lamine Hamzaoui, Ameur Cherif, Msadok Azouz, Gauthaman Kalamegam, Fazal Khan, Shilu Mathew, Mohammed Imran Nasser, Mahmood Rasool, Farid Ahmed, Peter Natesan Pushparaj, Mohammed Al-Qahtani, Shereen A. Turkistany, Lina M. Al-harbi, Ashraf Dallol, Jamal Sabir, Adeel Chaudhary, Adel Abuzenadah, Basmah Al-Madoudi, Bayan Al-Aslani, Khulud Al-Harbi, Rwan Al-Jahdali, Hanadi Qudaih, Emad Al Hamzy, Mourad Assidi, Mohammed Al Qahtani, Asad M. Ilyas, Youssri Ahmed, Mamdooh Gari, Farid Ahmed, Mohammed Alqahtani, Nada Salem, Sajjad Karim, Elham M. Alhathli, Heba Abusamra, Hend F. Nour Eldin, Mohammed H. Al-Qahtani, Sudhir Kumar, Fatima Al-Adwani, Deema Hussein, Mona Al-Sharif, Awatif Jamal, Fahad Al-Ghamdi, Jaudah Al-Maghrabi, Saleh S. Baeesa, Mohammed Bangash, Adeel Chaudhary, Mohammed Al-Qahtani, Hans-Juergen Schulten, Alaa Alamandi, Reem Alotibi, Deema Hussein, Sajjad Karim, Jaudah Al-Maghrabi, Fahad Al-Ghamdi, Awatif Jamal, Saleh S. Baeesa, Mohammed Bangash, Adeel Chaudhary, Hans-Juergen Schulten, Mohammed Al-Qahtani, Ohoud Subhi, Nadia Bagatian, Sajjad Karim, Adel Al-Johari, Osman Abdel Al-Hamour, Hosam Al-Aradati, Abdulmonem Al-Mutawa, Faisal Al-Mashat, Jaudah Al-Maghrabi, Hans-Juergen Schulten, Mohammad Al-Qahtani, Nadia Bagatian, Ohoud Subhi, Sajjad Karim, Adel Al-Johari, Osman Abdel Al-Hamour, Abdulmonem Al-Mutawa, Hosam Al-Aradati, Faisal Al-Mashat, Mohammad Al-Qahtani, Hans-Juergen Schulten, Jaudah Al-Maghrabi, Muhammad W. shah, Muhammad Yasir, Esam I Azhar, Saad Al-Masoodi, Yosr Z. Haffani, Msadok Azouz, Emna Khamla, Chaima Jlassi, Ahmed S. Masmoudi, Ameur Cherif, Lassaad Belbahri, Shadi Al-Khayyat, Roba Attas, Atlal Abu-Sanad, Mohammed Abuzinadah, Adnan Merdad, Ashraf Dallol, Adeel Chaudhary, Mohammed Al-Qahtani, Adel Abuzenadah, Habib Bouazzi, Carlos Trujillo, Mohammad Khalid Alwasiyah, Mohammed Al-Qahtani, Maha Alotaibi, Rami Nassir, Ishfaq A. Sheikh, Mohammad A. Kamal, Essam H. Jiffri, Ghulam M. Ashraf, Mohd A. Beg, Mohammad A. Aziz, Rizwan Ali, Mahmood Rasool, Mohammad S. Jamal, Nusaibah Samman, Ghufrana Abdussami, Sathish Periyasamy, Mohiuddin K. Warsi, Mohammed Aldress, Majed Al Otaibi, Zeyad Al Yousef, Mohamed Boudjelal, Abdelbasit Buhmeida, Mohammed H. Al-Qahtani, Ibrahim AlAbdulkarim, Rubi Ghazala, Shilu Mathew, M. Haroon Hamed, Mourad Assidi, Mohammed Al-Qahtani, Ishtiaq Qadri, Ishfaq A. Sheikh, Muhammad Abu-Elmagd, Rola F. Turki, Ghazi A. Damanhouri, Mohd A. Beg, Mohd Suhail, Abid Qureshi, Adil Jamal, Peter Natesan Pushparaj, Mohammad Al-Qahtani, Ishtiaq Qadri, Mahmoud Z. El-Readi, Safaa Y. Eid, Michael Wink, Ahmed M. Isa, Lulu Alnuaim, Johara Almutawa, Basim Abu-Rafae, Saleh Alasiri, Saleh Binsaleh, Nazia Nazam, Mohamad I. Lone, Waseem Ahmad, Shakeel A. Ansari, Mohamed H. Alqahtani

**Affiliations:** 1 University of Texas Southwestern Medical Center, Dallas, TX USA; 2 Department of Cell Biology and Anatomy, Graduate School of Medicine, the University of Tokyo, Tokyo, 113-0033 Japan; 3 Center of Excellence in Genomic Medicine Research, King Abdulaziz University, Jeddah, 21589 Saudi Arabia; 4 Department of Biochemistry and Molecular & Cellular Biology, Lombardi Comprehensive Cancer Center, Georgetown University, Washington, DC USA; 5 Department of Oncology, Lombardi Comprehensive Cancer Center, Georgetown University, Washington, DC USA; 6 Department of Radiation Medicine, Lombardi Comprehensive Cancer Center, Georgetown University, Washington, DC USA; 7 Center of Excellence in Genomic Medicine Research (CEGMR), King Abdulaziz University, Jeddah, Kingdom of Saudi Arabia; 8 King Fahd Medical Research Centre, King Abdulaziz University, Jeddah, Saudi Arabia; 9 Centre of Excellence in Genomic Medicine Research, King Abdulaziz University, Jeddah, Saudi Arabia; 10 Sheffield Diagnostic Genetic Service, Sheffield Children’s NHS Foundation Trust, Western Bank, Sheffield, UK; 11 Center of Innovations in Personalized Medicine, King Abdulaziz University, Jeddah, Saudi Arabia; 12 President, ISBER, Editor-in-Chief, Biopreservation & Biobanking, ᅟ, USA; 13 Institute of Applied Genetics, Department of Molecular and Medical Genetics, University of North Texas Health Science Center, Fort Worth, TX 76107 USA; 14 Center of Excellence in Genomic Medicine Research, King Abdulaziz University, Jeddah, Saudi Arabia; 15 Department of Pathology, Faculty of Medicine, King Abdulaziz University, Jeddah, Saudi Arabia; 16 Center of Excellence in Genomic Medicine Research, King Abdulaziz University, Jeddah, Saudi Arabia; 17 Department of Dental Public Health, Faculty of Dentistry, King Abdulaziz University, Jeddah, Saudi Arabia; 18 Institute for Genomics and Evolutionary Medicine, Temple University, Philadelphia, PA 19122 USA; 19 Genomic Medicine Group- Galician Foundation of Genomic Medicine (SERGAS), CIBERER, University of Santiago de Compostela, Santiago de Compostela, Spain; 20 Center of Excellence in Genomic Medicine, King Abdulaziz University, Jeddah, Kingdom of Saudi Arabia; 21 Laboratory of Molecular and Cellular Screening Processes (Bioinformatics Group), Centre of Biotechnology of Sfax, P.O. Box “1177”, Sfax, Tunisia; 22 Center of Excellence in Genomic Medicine Research, King Abdulaziz University, PO Box 80216, Jeddah, 21589 Saudi Arabia; 23 Institute for Genomics and Evolutionary Medicine, Temple University (SERC 602A), Philadelphia, PA 19122 USA; 24 Fujitsu Technology Solutions International, ᅟ, ᅟ; 25 Case Western Reserve University, School of Medicine and Lerner College of Medicine, Cleveland Clinic Foundation, Cleveland, Ohio USA; 26 American Center for Reproductive Medicine, Andrology Center, Cleveland, Ohio USA; 27 Center for Reproductive Medicine and Andrology, University of Münster, Münster, Germany; 28 Center of Excellence in Genomic Medicine Research, King Abdulaziz University, Jeddah, Saudi Arabia; 29 Department of Urology, King Abdulaziz University Hospital, Jeddah, Saudi Arabia; 30 Department of Obstetrics and Gynecology, King Abdulaziz University Hospital, Jeddah, Kingdom of Saudi Arabia; 31 KACST Technology Innovation Center in Personalized Medicine, King Abdulaziz University, Jeddah, Saudi Arabia; 32 Assisted Reproductive Technology Laboratory, Department of Pathology and Laboratory Medicine, King Faisal Specialist Hospital and Research Center, Riyadh, Kingdom of Saudi Arabia; 33 Center of Excellence in Genomic Medicine Research, King Abdulaziz University, Jeddah, Saudi Arabia; 34 Center of Innovation in Personalized Medicine, King Abdulaziz University, Jeddah, Saudi Arabia; 35 Department of Pathology, Faculty of medicine, King Abdulaziz University, Jeddah, Saudi Arabia; 36 Biodesign Institute, Arizona State University, Tempe, AZ 85281 USA; 37 Department of Chemistry and Biochemistry, Arizona State University, Tempe, AZ 85287 USA; 38 Forensic Biology Department, College of Forensic Sciences, Naif Arab University for Security Sciences, Riyadh, Kingdom of Saudi Arabia; 39 College of Pharmacy of Monastir, Monastir, Tunisia; 40 Department of Medical Biochemistry, Arabian Gulf University, Manama, Bahrain; 41 Department of Physics, Faculty of Science, King Abdulaziz University Jeddah, P.O. Box 80203, Jeddah, 21589 Saudi Arabia; 42 Internal Medicine and Cardiology, King Abdulaziz University Medical School, Jeddah, Saudi Arabia; 43 Department of Bioinformatics, National Cancer Center Research Institute, 5-1-1 Tsukiji, Chuuoo-ku, Tokyo, 104-0045 Japan; 44 Department of Gastroenterological Surgery, Osaka University Graduate School of Medicine, 2-2 E2, Yamadaoka, Suita City, Osaka 565-0871 Japan; 45 Division of Translational Research, Exploratory Oncology Research and Clinical Trial Center, National Cancer Center, 6-5-1 Kashiwanoha, Kashiwa, Chiba 277-8577 Japan; 46 Division of Cancer Genomics, National Cancer Center Research Institute, 5-1-1 Tsukiji, Chuuoo-ku, Tokyo, 104-0045 Japan; 47 Laboratory for Immune Signal, National Institute of Biomedical Innovation, 7-6-8 Saito-Asagi, Ibaraki City, Osaka 567-0085 Japan; 48 Department of Surgical Pathology, Hyogo Medical College, 1-1, Mukogawa-cho, Nishinomiya City, Hyogo 663-8501 Japan; 49 Department of Surgery, National Cancer Center Hospital East, 6-5-1 Kashiwanoha, Kashiwa, Chiba 277-8577 Japan; 50 Clinical Biochemistry Group, Department of Biochemistry & Molecular Biology, University of Gujrat, Punjab, Pakistan; 51 Department of Health Information Management, College of Applied Medical Sciences, Buraydah Colleges, Al Qassim, Saudi Arabia; 52 Department of Medical Laboratory, College of Applied Medical Sciences, Buraydah Colleges, Al Qassim, Saudi Arabia; 53 King Fahd Medical Research Centre, KAU, Jeddah, Kingdom of Saudi Arabia; 54 Department of Pathology, Faculty of Medicine, KAU, Jeddah, Kingdom of Saudi Arabia; 55 Centre for Excellence in Genomic Medicine Research, Jeddah, Kingdom of Saudi Arabia; 56 Department of Urology, King Faisal Specialist Hospital and research Centre, Jeddah, Kingdom of Saudi Arabia; 57 Department of Urology, KAUH, Jeddah, Kingdom of Saudi Arabia; 58 Research’s Laboratory of Human Genome and Multi-factorial diseases LR12ES07, College of Pharmacy, Monastir, 5000 Tunisia; 59 Faculty of Sciences, Bizerte, 7021 Tunisia; 60 Maternity and Neonatal Centre, Monastir, 5000 Tunisia; 61 Department of Medical Biochemistry, College of Medicine and Medical Sciences, Arabian Gulf University, P.O. Box 22979, Manama, Bahrain; 62 Sidra Medical and Research Center, PO Box 26999, Doha, Qatar; 63 Laboratory of Human Genome and Multifactorials diseases, Faculty of Pharmacy, Monastir, Tunisia; 64 Center of Excellence in Genomic Medicine Research (CEGMR), King Abdulaziz University, Jeddah, Kingdom of Saudi Arabia; 65 Research Unit Extracellular Matrix and Cellular Dynamic, Faculty of Pharmacy, Monastir, Tunisia; 66 King Fahd Medical Research Centre, KAU, Jeddah, Saudi Arabia; 67 Department of Urology, King Faisal Specialist Hospital and research Centre, Jeddah, Saudi Arabia; 68 Department of Urology, KAUH, Jeddah, Saudi Arabia; 69 Centre for Excellence in Genomic Medicine Research, Jeddah, Saudi Arabia; 70 Department of Pathology, Faculty of Medicine, KAU, Jeddah, Saudi Arabia; 71 Human Genome and Multifactorial Diseases Laboratory, Faculty of Pharmacy, University of Monastir, Monastir, 5000 Tunisia; 72 Faculty of Science of Bizerte, University of Carthage, Jarzouna, 7021 Tunisia; 73 Research Unit: Colorectal cancer in Young Subjects - Faculty of Medecine, University of Monastir, Monastir, 5000 Tunisia; 74 Surgery Department, University Hospital Fattouma Bourguiba, University of Monastir, Monastir, 5000 Tunisia; 75 Laboratory Medicine Department, Faculty of Applied Medical Sciences, Umm Al-Qura University, Makkah, Kingdom of Saudi Arabia; 76 Obstertics and Gynaecology Department, Maternity and Children Hospital, Jeddah, Kingdom of Saudi Arabia; 77 Hygiene and Zoonoses Department, Faculty of Veterinary Medicine, Mansoura University, Mansoura, 35516 Egypt; 78 Bacteriology, Mycology and Immunology Department, Faculty of Veterinary Medicine, Mansoura University, Mansoura, 35516 Egypt; 79 Animal Husbandry and Animal Wealth Development Department, Faculty of Veterinary Medicine, Mansoura University, Mansoura, 35516 Egypt; 80 Department of Laboratory Medicine, Faculty of Applied Medical Sciences, Umm Al-Qura University, PO Box 7607, Holy Makkah, Kingdom of Saudi Arabia; 81 Department of Pharmacology, Faculty of Medicine, Assiut University, Asyut, Egypt; 82 Department of Bioengineering, College of Engineering, Hanyang University, 222 Wangsinmi-ro, Seongdong-gu, Seoul, 133-791 Republic of Korea; 83 Department of Laboratory Medicine Department, Faculty of Applied Medical Sciences, Umm Al-Qura University, Makkah, PO Box 7607, Saudi Arabia; 84 Department of Laboratory Medicine, Faculty of Applied Medical Sciences, Umm Al-Qura University, PO Box 7607, Holy Makkah, Saudi Arabia; 85 Department of Pharmacology, Faculty of Medicine, Assiut University, Assiut, Egypt; 86 Department of Clinical Nutrition, Faculty of Applied Medical Sciences, Umm Al-Qura University, PO Box 7607, Holy Makkah, Saudi Arabia; 87 Biology Department, College of Education, Iraqia University, Baghdad, Iraq; 88 Biotechnology Research Center, Al- Nahrain University, Baghdad, Iraq; 89 College of dentistry, Baghdad University, Baghdad, Iraq; 90 Department of Physiology, College of Medicine, King Saud University, Riyadh, Kingdom of Saudi Arabia; 91 Centre for Excellence in Genomic Medicine Research, King Abdulaziz University, Jeddah, Kingdom of Saudi Arabia; 92 King Fahd Medical Research Centre, King Abdulaziz University, Jeddah, Kingdom of Saudi Arabia; 93 Institute of Genetic Medicine, Newcastle University, Newcastle upon Tyne, UK; 94 Security Forces Hospital, General Directorate of Medical Services, Ministry of Interior, Makkah, Kingdom of Saudi Arabia; 95 Department of Biochemistry, Faculty of Science, The M.S. University of Baroda, Vadodara, 390 002 Gujarat India; 96 Department of Skin andVD, Sir Sayajirao Gaikwad Medical College, The Maharaja Sayajirao University of Baroda, Vadodara, Gujarat India; 97 Human Cytogenetics Department, National Research Centre, Dokki, Egypt; 98 Clinical Genetics Department, National Research Centre, Dokki, Egypt; 99 Department of Biochemistry, College of Medicine, University of Hail, Hail, Saudi Arabia; 100 Department of Health Information Management, College of Applied Medical Sciences, Buraydah Colleges, Al Qassim, Saudi Arabia; 101 Department of Biosciences, Faculty of Applied Science, Integral University, Lucknow, India; 102 Laboratory of molecular and cellular Hematology, Pasteur Institute of Tunis, University of Tunis El-Manar, 74 PO Box. 1002, Belvedere, Tunis, Tunisia; 103 Service d’Immuno-Hématologie pédiatrique, Centre National de Greffe de Moelle Osseuse, Tunis, Tunisia; 104 Department of Molecular Biology, National Cancer Institute (NCI-UG), University of Gezira, Wad Madani, Sudan; 105 Department of Biochemistry and Nutrition, Faculty of Medicine, University of Gezira, Wad Madani, Sudan; 106 Department of Biochemistry, Orotta School of Medicine and Dentistry, ᅟ, Eritrea; 107 Department of Biochemistry, Faculty of Science, King Abdulaziz University, Jeddah, Saudi Arabia; 108 College of Medicine, Institute for Research and Medical Consultations, University of Dammam, Dammam, Saudi Arabia; 109 Clinical Genetics Department, National Research Centre, Cairo, 12622 Egypt; 110 Cytogenetics Department, National Research Centre, Cairo, 12622 Egypt; 111 Biochemistry Department, Faculty of Sciences, King Abdulaziz University, Jeddah, Saudi Arabia; 112 Biological Sciences Department - Faculty of Sciences, King Abdulaziz University, Jeddah, Saudi Arabia; 113 Department of Pediatrics, Faculty of Medicine, King Abdulaziz University, Jeddah, Saudi Arabia; 114 Flow Cytometry Core Facility, National Heart, Lung, and Blood Institute, National Institutes of Health, Bethesda, MD USA; 115 Biotechnology and Genetic Engineering Research Unit, Deanship of Scientific Research, Taif University, Taif, Kingdom of Saudi Arabia; 116 Department of Genetics, Faculty of Agriculture and Natural Resources, Aswan University, Aswan, Egypt; 117 College of Biotechnology, Misr University for Science and Technology, Sixth of October City, Egypt; 118 Sheikh Salem Bin Mahfouz Scientific Chair for Treatment of Osteoarthritis by Stem Cells, King Abdulaziz University, Jeddah, Saudi Arabia; 119 Center of Innovation in Personalized Medicine, King Abdulaziz University, Jeddah, Saudi Arabia; 120 Department of Hematology, Faculty of Medicine, King Abdulaziz University Hospital, King Abdulaziz University, Jeddah, Saudi Arabia; 121 Department of Orthopedic Surgery, Faculty of Medicine, King Abdulaziz University, Jeddah, Saudi Arabia; 122 Stem Cell Unit, Centre of Excellence in Genomic Medicine Research, King Abdulaziz University, Jeddah, Saudi Arabia; 123 Department of Medical Laboratory Technology, Faculty of Applied Medical Sciences, King Abdulaziz University, Jeddah, Saudi Arabia; 124 Center of Excellence in Genomic Medicine Research, King Abdulaziz University, PO Box 80216, Jeddah, 21589 Saudi Arabia; 125 Institute for Genomics and Evolutionary Medicine, Temple University, Philadelphia, PA 19122 USA; 126 Department of Biology, Genomic and Biotechnology Section, King Abdulaziz University, Jeddah, Saudi Arabia; 127 Princess Al-Jawhara Al-Ibrahim Centre of Excellence in Research of Hereditary Disorders, Faculty of Medicine, King Abdul-Aziz University, Jeddah, Saudi Arabia; 128 Department of Clinical Biochemistry, Faculty of Medicine, King Abdulaziz University, Jeddah, Saudi Arabia; 129 Future Scientist Program Trainee at Genetics Department, King Faisal Specialist Hospital and Research Centre, Riyadh, Saudi Arabia; 130 Department of Biostatistics, Epidemiology and Scientific Computing, King Faisal Specialist Hospital and Research Centre, Riyadh, Saudi Arabia; 131 University of Wolverhampton, Wolverhampton, West Midlands WV1 1LY UK; 132 Department of Biochemistry, Faculty of Science, King Abdulaziz University, Jeddah, Saudi Arabia; 133 Department of Medical Laboratory Technology, Faculty of Applied Medical Sciences, King Abdulaziz University, Jeddah, Saudi Arabia; 134 Sheikh Salem Bin Mahfouz Scientific Chair for Treatment of Osteoarthritis by Stem Cells, King Abdulaziz University, Jeddah, Saudi Arabia; 135 Stem Cells Unit, Center of Excellence in Genomic Medicine Research (CEGMR), King Abdulaziz University, Jeddah, Saudi Arabia; 136 Center of Innovation in Personalized Medicine, King Abdulaziz University, Jeddah, Saudi Arabia; 137 Department of Orthopaedic Surgery, Faculty of Medicine, King Abdulaziz University Hospital, King Abdulaziz University, Jeddah, Saudi Arabia; 138 Center of Excellence in Genomic Medicine Research, King Abdulaziz University, Jeddah, Saudi Arabia; 139 Institute for Genomics and Evolutionary Medicine, Temple University, Philadelphia, PA 19122 USA; 140 Medical Laboratories Technology Department, Applied Medical Science College, Taibah University, Al Madinah, Al Munawarah, Saudi Arabia; 141 Center for Genetics and Inherited Diseases, Taibah University, Al Madinah, Al Munawarah, Saudi Arabia; 142 American Center for Reproductive Medicine, Cleveland Clinic, Cleveland, OH USA; 143 Physiology, Universiti Teknologi, Sungai Buloh, MARA, Malaysia; 144 Redox Biology Laboratory, School of Life Science, Ravenshaw University, Orissa, India; 145 Center of Excellence in Genomic Medicine Research, Jeddah, Saudi Arabia; 146 KACST Center of Innovation in Personalized Medicine at King Abdulaziz University, Jeddah, Saudi Arabia; 147 Urology Department, Cleveland Clinic, Cleveland, OH USA; 148 American Center for Reproductive Medicine, Cleveland Clinic, Cleveland, Ohio USA; 149 Redox Biology Laboratory, School of Life Science, Ravenshaw University, Orissa, India; 150 Physiology, Universiti Teknologi MARA, Sungai Buloh, Malaysia; 151 Center of Excellence in Genomic Medicine Research, King Abdulaziz University, Jeddah, Saudi Arabia; 152 KACST Center of Innovation in Personalized Medicine, King Abdulaziz University, Jeddah, Saudi Arabia; 153 Urology Department, Cleveland Clinic, Cleveland, OH USA; 154 American Center For Reproductive Medicine, Cleveland Clinic, Cleveland, OH USA; 155 Redox Biology Laboratory, School of Life Science, Ravenshaw University, Orissa, India; 156 Institute of Toxicology, Third Military Medical University, Chongqing, China; 157 Center of Excellence in Genomic Medicine Research, King Abdulaziz University, Jeddah, Saudi Arabia; 158 KACST Center of Innovation in Personalized Medicine, King Abdulaziz University, Jeddah, Saudi Arabia; 159 Center of Excellence in Genomic Medicine Research, King Abdulaziz University, Jeddah, 21589 Saudi Arabia; 160 Department of Obstetrics and Gynecology, King Abdulaziz University Hospital, Faculty of Medicine, King Abdulaziz University, Jeddah, 21589 Saudi Arabia; 161 King Fahd Medical Research Center, King Abdulaziz University, Jeddah, 21589 Saudi Arabia; 162 Center of Excellence in Genomic Medicine Research, King Abdulaziz University, PO BOX 80216, Jeddah, 21589 Saudi Arabia; 163 Department of Urology, Faculty of Medicine, King Abdulaziz University, Jeddah, Saudi Arabia; 164 Department of Pathology, Faculty of Medicine, King Abdulaziz University, Jeddah, Saudi Arabia; 165 Department of Pathology, King Faisal Specialist Hospital and Research Center, Jeddah, Saudi Arabia; 166 King Fahd Medical Research Center, King Abdulaziz University, PO BOX 80216, Jeddah, 21589 Saudi Arabia; 167 Zoology Department, Women’s College for Arts, Science & Education, Ain Shams University, Cairo, Egypt; 168 Biology Department, Faculty of Applied Science, Umm Al- Qura University, Makka, Saudi Arabia; 169 Zoology Department, Faculty of Science, Al-Azhar University, Cairo, Egypt; 170 Biotechnology Department, Faculty of Science, Taif University, Taif, Saudi Arabia; 171 King Abdullah University of Science and Technology (KAUST), Computer, Electrical and Mathematical Sciences and Engineering Division, Thuwal, 23955-6900 Saudi Arabia; 172 Center of Excellence in Genomic Medicine Research, King Abdulaziz University, PO Box 80216, Jeddah, 21589 Saudi Arabia; 173 King Abdullah University of Science and Technology (KAUST), Computational Bioscience Research Center, Thuwal, Jeddah, 23955-6900 Saudi Arabia; 174 Department of Pathology, King Abdulaziz University, Jeddah, Saudi Arabia; 175 Department of Pathology, Faculty of Medicine, Minia University, Al Minia, Egypt; 176 Department of Medicine, King Abdulaziz University, Jeddah, Saudi Arabia; 177 Center of Excellence in Genomic Medicine Research, King Abdulaziz University, Jeddah, Saudi Arabia; 178 Department of Pathology, King Abdulaziz University, Jeddah, Saudi Arabia; 179 Department of Medicine, King Abdulaziz University, Jeddah, Saudi Arabia; 180 Center of Excellence in Genomic Medicine Research, King Abdulaziz University, Jeddah, Saudi Arabia; 181 Center of Excellence in Genomic Medicine Research, King Abdulaziz University, P.O. Box 80216, Jeddah, 21589 Saudi Arabia; 182 Institute for Genomics and Evolutionary Medicine, Temple University, Philadelphia, PA 19122 USA; 183 Department of Biochemistry, Faculty of Science, King Abdulaziz University, Jeddah, Saudi Arabia; 184 Stem Cells Unit, Centre of Excellence in Genomic Medicine Research (CEGMR), King Abdulaziz University, Jeddah, Saudi Arabia; 185 Sheikh Salem Bin Mahfouz Scientific Chair for Treatment of Osteoarthritis by Stem Cells, King Abdulaziz University, Jeddah, Saudi Arabia; 186 Department of Medical Laboratory Technology, Faculty of Applied Medical Sciences, King Abdulaziz University, Jeddah, Saudi Arabia; 187 Department of Orthopaedic Surgery, Faculty of Medicine, King Abdulaziz University, Jeddah, Saudi Arabia; 188 Center of Innovation in Personalized Medicine, King Abdulaziz University, Jeddah, Saudi Arabia; 189 Faculty of Dentistry, King Abdulaziz University, Jeddah, Saudi Arabia; 190 Center of Excellence in Genomic Medicine Research, King Abdulaziz University, Jeddah, Saudi Arabia; 191 KACST Technology Innovation Center in Personalized Medicine at King AbdulAziz University, Jeddah, Saudi Arabia; 192 Dental public health department, Faculty of Dentistry, King Abdulaziz University, Jeddah, Saudi Arabia; 193 Department of Biochemistry, Faculty of Science, King Abdulaziz University, Jeddah, Saudi Arabia; 194 Department of Medical Laboratory Technology, Faculty of Applied Medical Sciences, King Abdulaziz University, Jeddah, Saudi Arabia; 195 Sheikh Salem Bin Mahfouz Scientific Chair for Treatment of Osteoarthritis by Stem Cells, King Abdulaziz University, Jeddah, Saudi Arabia; 196 Stem Cells Unit, Centre of Excellence in Genomic Medicine Research (CEGMR), King Abdulaziz University, Jeddah, Saudi Arabia; 197 Department of Orthopaedic Surgery, Faculty of Medicine, King Abdulaziz University Hospital, Jeddah, Saudi Arabia; 198 Center of Innovation in Personalized Medicine, King Abdulaziz University, Jeddah, Saudi Arabia; 199 Department of Medicine, University of Health Science, Lahore, Pakistan; 200 Center of Excellence in Genomic Medicine Research, King Abdulaziz University, Jeddah, Saudi Arabia; 201 Department of Biology, King Abdul-Aziz University, Jeddah, Saudi Arabia; 202 Center of Innovation in Personalized Medicine at King Abdulaziz University, Jeddah, Saudi Arabia; 203 King Fahd Medical Research Center, King Abdul Aziz University, Jeddah, Saudi Arabia; 204 Center of Excellence in Genomic Medicine Research, King Abdulaziz University, Jeddah, Saudi Arabia; 205 Center of Innovation in Personalized Medicine, King Abdulaziz University, Jeddah, Saudi Arabia; 206 Center of Excellence in Genomic Medicine Research, King Abdulaziz University, Jeddah, Saudi Arabia; 207 Center of Innovation in Personalized Medicine at King Abdulaziz University, Jeddah, Saudi Arabia; 208 Bioinformatics Group, Centre of Biotechnology of Sfax, Sfax, Tunisia; 209 Center of Excellence in Genomic Medicine Research, King Abdulaziz University, Jeddah, Saudi Arabia; 210 Center of Innovation in Personalized Medicine, King Abdulaziz University, Jeddah, Saudi Arabia; 211 Department of Cell Biology, The University of Texas Southwestern Medical Center, Dallas, TX USA; 212 Zoology Department, College of Sciences, King Saud University, Riyadh, Saudi Arabia; 213 Institute of Applied Genetics, Department of Molecular and Medical Genetics, University of North Texas Health Science Center, Fort Worth, TX 76107 USA; 214 Institute of Gene Technology, Department of Molecular Diagnostics, Tallinn University of Technology, Akadeemia tee 15A-604, Tallinn, 12618 Estonia; 215 Laboratory of Forensic Anthropology, Center for Human Identification, Department of Biological Sciences, University of North Texas, 1511 West Sycamore, Denton, Texas USA; 216 Center of Excellence in Genomic Medicine Research, King Abdulaziz University, Jeddah, Saudi Arabia; 217 Center of Excellence in Genomic Medicine Research, King Abdulaziz University, Jeddah, Saudi Arabia; 218 Center of Innovation in Personalized Medicine, King Abdulaziz University, Jeddah, Saudi Arabia; 219 Urology Department, College of Medicine, King Abdulaziz University, Jeddah, Saudi Arabia; 220 Center for Reproductive Medicine, Cleveland Clinic, Cleveland, OH USA; 221 Center of Excellence in Genomic Medicine Research, King Abdulaziz University, PO Box 80216, Jeddah, 21589 Saudi Arabia; 222 Department of Urology, Faculty of Medicine, King Abdulaziz University, Jeddah, Saudi Arabia; 223 Department of Pathology, Faculty of Medicine, King Abdulaziz University, Jeddah, Saudi Arabia; 224 Department of Pathology, King Faisal Specialist Hospital and Research Center, Jeddah, Saudi Arabia; 225 King Fahd Medical Research Center, King Abdulaziz University, PO Box 80216, Jeddah, 21589 Saudi Arabia; 226 Department of Biochemistry, Ibn Sina National College for Medical Sciences, Jeddah, 21418 Kingdom of Saudi Arabia; 227 Center of Excellence in Genomic Medicine Research, King Abdulaziz University, Jeddah, 21589 Kingdom of Saudi Arabia; 228 Toxicogemics Laboratory, Division of Genetics, Department of Zoology, Aligarh Muslim University, Aligarh, India; 229 King Fahd Medical Research Center, King Abdulaziz University, Jeddah, 21589 Kingdom of Saudi Arabia; 230 Center of Excellence in Genomic Medicine (CEGMR), King Abdulaziz University, Jeddah, Saudi Arabia; 231 Department of Biochemistry, Faculty of Science, King Abdulaziz University, Jeddah, Kingdom of Saudi Arabia; 232 Center of Excellence in Genomic Medicine Research, King Abdulaziz University, Jeddah, Kingdom of Saudi Arabia; 233 Department of Multimedia Technology, Karunya University, Coimbatore, India; 234 Faculy of Applied Medical Sciences, King Abdulaziz University, Jeddah, Saudi Arabia; 235 Centre of Innovation in Personalized Medicine, King Abdulaziz University, Jeddah, Saudi Arabia; 236 Centre of Excellence in Genomic Medicine Research, King Abdulaziz University, Jeddah, Saudi Arabia; 237 Faculty of Medical Sciences, King Abdulaziz University, Jeddah, Saudi Arabia; 238 Pediatric Cardiology Department, King Abdulaziz University, Jeddah, Saudi Arabia; 239 Children Hospital, Genetics Department, Ministry of Health, Taif, Saudi Arabia; 240 Cardiology Unit, King Abdulaziz University Hospital, Jeddah, Saudi Arabia; 241 Gene-Tox Laboratory, Division of Genetics, Department of Zoology, Aligarh Muslim University, Aligarh, UP India; 242 Center of Excellence in Genomic Medicine Research, King Abdulaziz University, Jeddah, Saudi Arabia; 243 Diagnostic Genomic Medicine Unit, Center of Excellence in Genomic Medicine Research, King Abdulaziz University, Jeddah, Saudi Arabia; 244 Center of Excellence in Genomic Medicine Research, King Abdulaziz University, Jeddah, Saudi Arabia; 245 Center of Innovation in Personalized Medicine, King Abdulaziz University, Jeddah, Saudi Arabia; 246 Center of Excellence in Genomic Medicine Research, King Abdulaziz University, Jeddah, Kingdom of Saudi Arabia; 247 Centre of Excellence in Genomic Medicine Research (CEGMR), King Abdulaziz University, Jeddah, Saudi Arabia; 248 Department of Biochemistry, Faculty of Science, King Abdulaziz University, Jeddah, Kingdom of Saudi Arabia; 249 Department of Obstetrics and Gynaecology, Faculty of Medicine, King Abdulaziz University, Jeddah, Kingdom of Saudi Arabia; 250 Center of Excellence in Genomic Medicine Research, King Abdulaziz University, Jeddah, Saudi Arabia; 251 King Fahd Medical Research Center, King Abdulaziz University, Jeddah, Saudi Arabia; 252 Center of Excellence in Genomic Medicine Research, King Abdulaziz University, Jeddah, Saudi Arabia; 253 King Fahd Medical Research Center, King Abdulaziz University, Jeddah, Saudi Arabia; 254 Department of Medical Genetics, King Fahad General Hospital, Jeddah, Saudi Arabia; 255 Children Hospital, Taif, Saudi Arabia; 256 Center of Excellence in Genomic Medicine Research, King Abdulaziz University, Jeddah, Saudi Arabia; 257 King Fahd Medical Research Center, King Abdulaziz University, Jeddah, Saudi Arabia; 258 King Abdulaziz University Hospital, Jeddah, Saudi Arabia; 259 Mohammad Ali Jauhar University, Rampur, UP India; 260 Center for Interdisciplinary Research in Basic Sciences, Jamia Millia Islamia, Jamia Nagar, New Delhi India; 261 Department of Computer Science, Jamia Millia Islamia, Jamia Nagar, New Delhi India; 262 King Fahd Medical Research Center, King Abdulaziz University, Jeddah, Saudi Arabia; 263 Center of Excellence in Genomic Medicine Research, King Abdulaziz University, Jeddah, Saudi Arabia; 264 King Fahd Medical Research Center, King Abdulaziz University, Jeddah, Saudi Arabia; 265 Center of Excellence in Genomic Medicine Research, King Abdulaziz University, Jeddah, Saudi Arabia; 266 Jamia Millia Islamia, New Delhi, India; 267 Department of Biotechnology, Kadi Sarva Vishwavidhyalaya, Gandhinagar, Gujarat India; 268 King Fahd Medical Research Center, King Abdulaziz University, Jeddah, Saudi Arabia; 269 Clinical Biochemistry Department, College of Medicine, King Abdulaziz University, Jeddah, Saudi Arabia; 270 Center of Excellence in Genomic Medicine Research, King Abdulaziz University, Jeddah, Saudi Arabia; 271 Center of Excellence in Genomic Medicine Research, King Abdulaziz University, Jeddah, Saudi Arabia; 272 Center of Innovation in Personalized Medicine, King Abdulaziz University, Jeddah, Saudi Arabia; 273 Obstetrics and Gynecology Department, King AbdulAziz University Hospital, Jeddah, Saudi Arabia; 274 Faculty of Applied Medical Sciences, King Abdulaziz University, Jeddah, Saudi Arabia; 275 King Fahd Medical Research Center, King Abdulaziz University, Jeddah, Saudi Arabia; 276 Applied Medical Science Laboratory Department, King Abdulaziz University, Jeddah, Saudi Arabia; 277 Center of Excellence in Genomic Medicine Research, King Abdulaziz University, Jeddah, Saudi Arabia; 278 Center of Excellence in Genomic Medicine Research, King Abdulaziz University, 21589 Jeddah, Kingdom of Saudi Arabia; 279 Department of Medical Genetics, King Fahad General Hospital, Jeddah, 21196 Saudi Arabia; 280 Department of Pediatric Neurologist, Maternity and Children Hospital, Jeddah, Saudi Arabia; 281 The Centre for Applied Genomics, The Hospital for Sick Children, Toronto, Ontario M5G 1L7 Canada; 282 McLaughlin Centre and Department of Molecular Genetics, University of Toronto, Toronto, Ontario M5G 1L7 Canada; 283 Center of Excellence in Genomic Medicine Research, King Abdulaziz University, Jeddah, Saudi Arabia; 284 Department of Biochemistry, Faculty of Science, King Abdulaziz University, Jeddah, Kingdom of Saudi Arabia; 285 Department of Pediatrics, Faculty of Medicine, King Abdulaziz University, Jeddah, Kingdom of Saudi Arabia; 286 Biochemistry Department, King Abdulaziz University, Jeddah, Saudi Arabia; 287 Center of Excellence in Genomic Medicine Research, King Abdulaziz University, Jeddah, Saudi Arabia; 288 Department of Medicine, Faculty of Medicine, King Abdulaziz University, Jeddah, Saudi Arabia; 289 Department of Pathology, Faculty of Medicine, King Abdulaziz University, Jeddah, Saudi Arabia; 290 Department of Genetic Medicine and Development, Medical School, University of Geneva, Geneva, Switzerland; 291 Medical Biochemistry Departments, Faculty of Medicine, Menoufia University, Shibin Al Kawm, Egypt; 292 Departments of Cardiology, Faculty of Medicine, Menoufia University, Shibin Al Kawm, Egypt; 293 Departments of Anesthesia, Faculty of Medicine, Menoufia University, Shibin Al Kawm, Egypt; 294 Biochemistry Departments, Faculty of Medicine, Umm Al-Qura University, Makkha, Kingdom of Saudi Arabia; 295 Center of Innovations in Personalized Medicine, King Abdulaziz University, Jeddah, Kingdom of Saudi Arabia; 296 Center of Excellence in Genomic Medicine Research, King Abdulaziz University, Jeddah, Kingdom of Saudi Arabia; 297 Diabetes & Endocrinology Center of Excellence, Clinical Nutrition Department, International Medical Center, Jeddah, Kingdom of Saudi Arabia; 298 Faculty of Applied Medical Sciences, King Abdulaziz University, Jeddah, Kingdom of Saudi Arabia; 299 Special Infectious Agents Unit, King Fahd Medical Research Center, King Abdulaziz University, Jeddah, 21589 Kingdom of Saudi Arabia; 300 Department of Medical Laboratory Technology, Faculty of Applied Medical Sciences, King Abdulaziz University, Jeddah, Saudi Arabia; 301 Center of Excellence in Genomic Medicine Research (CEGMR), King Abdulaziz University, Jeddah, 21589 Saudi Arabia; 302 Department of Medical Microbiology and Parasitology, Faculty of Medicine, King Abdulaziz University, Jeddah, Saudi Arabia; 303 Department of Anatomical pathology, Faculty of Medicine, King Abdulaziz University, Jeddah, Saudi Arabia; 304 Department of Microbiology, King Abdulaziz University, Jeddah, Saudi Arabia; 305 Department of Computer Science, Faculty of computing and Information Technology Rabigh, King Abdulaziz University, Rabigh, 21911 Saudi Arabia; 306 Kusuma School of biological Sciences, Indian Institute of Technology, Delhi (IITD), Hauz Khas, New Delhi, 110016 India; 307 Mathematics Department, Faculty of Sciences, King Abdul Aziz University, P. O. Box 80003, Jeddah, 21589 Saudi Arabia; 308 Department of Information Technology, Faculty of computing and Information Technology Rabigh, King Abdulaziz University, Rabigh, 21911 Saudi Arabia; 309 Center of Excellence in Genomic Medicine Research, King Abdulaziz University, Jeddah, Saudi Arabia; 310 Center of Innovation in Personalized Medicine, King Fahd Medical Research Center, King Abdulaziz University, Jeddah, Saudi Arabia; 311 Biochemistry Department, Faculty of Science, Production of Bioproducts for Industrial Applications Research Group and Experimental Biochemistry Unit, King Fahd Medical Research Center King, Abdulaziz University, Jeddah, Saudi Arabia; 312 Department of Agriculture, Faculty of Agricultural and Food Sciences, American University of Beirut (AUB), Beirut, Lebanon; 313 Adjuncted to Biochemistry Department, King Abdulaziz University, Jeddah, Saudi Arabia; 314 Center of Excellence in Genomic Medicine Research, King Abdulaziz University, Jeddah, Saudi Arabia; 315 Department of Surgery, King Abdulaziz University, Jeddah, Saudi Arabia; 316 Center of Excellence in Genomic Medicine Research, King Abdulaziz University, Jeddah, Saudi Arabia; 317 Center of Innovation in Personalized Medicine, King Abdulaziz University, Jeddah, Saudi Arabia; 318 Deaprtment of Computer Science, Faculty of computing and Information Technology Rabigh, King Abdulaziz University, Rabigh, 21911 Saudi Arabia; 319 Department of Information Systems, Faculty of computing and Information Technology Rabigh, King Abdulaziz University, Rabigh, 21911 Saudi Arabia; 320 Department of Medical Laboratory Technology and Haematology, Faculty of Applied Medical Sciences, King Abdulaziz University, Jeddah, Saudi Arabia; 321 Centre of Excellence in Genomic Medicine Research, King Abdulaziz University, Jeddah, Saudi Arabia; 322 Sheikh Salem Bin Mahfouz Scientific Chair for Treatment of Osteoarthritis by Stem Cells, King Abdulaziz University, Jeddah, Saudi Arabia; 323 Center of Innovation in Personalized Medicine, King Abdulaziz University, Jeddah, Saudi Arabia; 324 Department of Orthopaedic Surgery, Faculty of Medicine, King Abdulaziz University Hospital, Jeddah, Saudi Arabia; 325 Center of Excellence in Genomic Medicine Research, Faculty of Applied Medical Sciences, King Abdulaziz University, Jeddah, Saudi Arabia; 326 Center of Innovation in Personalized Medicine, King Abdulaziz University, Jeddah, Saudi Arabia; 327 Biochemistry Department, Faculty of Science, Production of Bioproducts for Industrial Applications Research Group and Experimental Biochemistry Unit, King Fahd Medical Research Center King, Abdulaziz University, Jeddah, Saudi Arabia; 328 King Fahd Medical Research Center, Faculty of Applied Medical Sciences, King Abdulaziz University, Jeddah, Saudi Arabia; 329 Center of Excellence in Genomic Medicine Research, King Abdulaziz University, Jeddah, Saudi Arabia; 330 Center of Innovation in Personalized Medicine, King Abdulaziz University, Jeddah, Saudi Arabia; 331 Department of Environmental sciences, Faculty of Meteorology, Environment and Arid Land Agriculture, King Abdulaziz University, Jeddah, Saudi Arabia; 332 New York University School of Medicine, Nelson Institute of Environmental Medicine, New York, USA; 333 Centre of Biotechnology of Sfax, Laboratory of Molecular and Cellular Screening Processes, Bioinformatics Group, Po. Box: 1177, 3018 Sfax, Tunisia; 334 Center of Excellence in Genomic Medicine Research, King Abdulaziz University, Jeddah, Saudi Arabia; 335 Center of Innovation in Personalized Medicine, King Abdulaziz University, Jeddah, Saudi Arabia; 336 King Fahd Medical Research Centre (KFMRC), King Abdulaziz University, Jeddah, Saudi Arabia; 337 Centre of Excellence in Genomic Medicine Research (CEGMR), King Abdulaziz University, Jeddah, Saudi Arabia; 338 Aziziah Maternity and Children Hospital, Jeddah, Saudi Arabia; 339 Department of Biochemistry, Faculty of Science, King Abdulaziz University, Jeddah, Saudi Arabia; 340 Center of Innovation in Personalized Medicine, King Abdulaziz University, Jeddah, Saudi Arabia; 341 Sheikh Salem Bin Mahfouz Scientific Chair for Treatment of Osteoarthritis by Stem Cells, King Abdulaziz University, Jeddah, Saudi Arabia; 342 Department of Orthopaedic Surgery, Faculty of Medicine, King Abdulaziz University Hospital, Jeddah, Saudi Arabia; 343 The D-BOARD European Consortium for Biomarker Discovery, The APPROACH Innovative Medicines Initiative (IMI) Consortium, Faculty of Health and Medical Sciences, University of Surrey, Guildford, Surrey GU2 7XH UK; 344 Arthritis Research UK Centre for Sport, Exercise and Osteoarthritis, Arthritis Research UK Pain Centre, Medical Research Council and Arthritis Research UK Centre for Musculoskeletal Ageing Research, University of Nottingham, Queen’s Medical Centre, Nottingham, NG7 2UH UK; 345 Department of Biochemistry, Faculty of Science, King Abdulaziz University, Jeddah, Saudi Arabia; 346 Center of Excellence in Genomic Medicine Research, King Abdulaziz University, Jeddah, Saudi Arabia; 347 Center of Excellence in Genomic Medicine Research, King Abdulaziz University, Jeddah, Kingdom of Saudi Arabia; 348 Department of Biochemistry, Faculty of Science, King Abdulaziz University, Jeddah, Kingdom of Saudi Arabia; 349 Department of Multimedia Technology, Karunya University, Coimbatore, India; 350 King Fahad Center for Medical Research, King Abdulaziz University, Jeddah, Saudi Arabia; 351 Center of Excellence in Genomic Medicine Research, King Abdulaziz University, Jeddah, Saudi Arabia; 352 Department of Biological Sciences, College of Science and Arts-Rabigh, King Abdulaziz University, Jeddah, Kingdom of Saudi Arabia; 353 Center of Emphasis in Neuroscience, Texas Tech University Health Science Center, El Paso, 79905 Texas USA; 354 Faculty of Computing and Information Technology, King Abdulaziz University, Rabigh, 21911 Kingdom of Saudi Arabia; 355 Department of Biological Sciences and Engineering, Maulana Azad National Institute of Technology, Bhopal, 462051 India; 356 Faculty of Computing and Information Technology, King Abdulaziz University, Rabigh, Saudi Arabia; 357 Department of Biotechnology, KadiSarvaVishwavidhyalaya, Gandhinagar, Gujarat India; 358 King Fahd Medical Research Center, King Abdulaziz University, Jeddah, Saudi Arabia; 359 King Fahd Medical Research Center, King Abdulaziz University, Jeddah, Kingdom of Saudi Arabia; 360 Center of Excellence in Genomic Medicine Research, King Abdulaziz University, Jeddah, Kingdom of Saudi Arabia; 361 Faculty of Computing and Information Technology, King Abdulaziz University, Rabigh, Kingdom of Saudi Arabia; 362 Department of Obstetrics and Gynecology, Faculty of Medicine, King Abdulaziz University, Jeddah, Kingdom of Saudi Arabia; 363 KACST Technology Innovation Center in Personalized Medicine, King Abdulaziz University, Jeddah, Kingdom of Saudi Arabia; 364 Department of Clinical and Chemical Pathology, Faculty of Medicine, Tanta University, Tanta, Egypt; 365 Cardiogenetic Team, Department of Pediatrics, College of Medicine, Taibah University, AL Madinah, Saudi Arabia; 366 Department of Human Genetics, Medical Research Institute, Alexandria University, Alexandria, Egypt; 367 Department of Cardiology, Faculty of Medicine, Tanta University, Tanta, Egypt; 368 Department of Biochemistry, Faculty of Science, King Abdulaziz University, Jeddah, Saudi Arabia; 369 Centre for Innovation in Personalized Medicine, King Abdulaziz University, Jeddah, Saudi Arabia; 370 Centre for Excellence in Genomic Medicine Research, King Abdulaziz University, Jeddah, Saudi Arabia; 371 Center of Excellence for Genomic Medicine Research, King Abdulaziz University, Jeddah, Saudi Arabia; 372 Biochemistry Department, Faculty of Science, King Abdulaziz University, Jeddah, Saudi Arabia; 373 Special Infectious Agents Unit, King Fahd Medical Research Center, King Abdulaziz University, Jeddah, Saudi Arabia; 374 Medical Laboratory Technology Department, Faculty of Applied Medical Sciences, King Abdulaziz University, Jeddah, Saudi Arabia; 375 Department of Obstetrics & Gynaecology, King Abdul Aziz University Hospital, Jeddah, Saudi Arabia; 376 Faculty of Agriculture, American University of Beirut, Beirut, Lebanon; 377 Adjunct to Biochemistry Department, King Abdulaziz University, Jeddah, Saudi Arabia; 378 Center of Excellence in Genomic Medicine Research, King Abdulaziz University, Jeddah, Saudi Arabia; 379 Center of Innovation in Personalized Medicine, King Fahd Medical Research Center, King Abdulaziz University, Jeddah, Saudi Arabia; 380 Biochemistry Department, Faculty of Science, Abdulaziz University, Jeddah, Saudi Arabia; 381 Production of Bioproducts for Industrial Applications Research Group, Abdulaziz University, Jeddah, Saudi Arabia; 382 Experimental Biochemistry Unit, King Fahd Medical Research Center King, Abdulaziz University, Jeddah, Saudi Arabia; 383 Department of Agriculture, Faculty of Agricultural and Food Sciences, American University of Beirut (AUB), Beirut, Lebanon; 384 adjuncted to Biochemistry Department, King Abdulaziz University, Jeddah, Saudi Arabia; 385 Clinical Pathology Department, Mansoura University, Faculty of Medicine, Mansoura, Egypt; 386 Rheumatology and Rehabilitation Department, Mansoura University, Faculty of Medicine, Mansoura, Egypt; 387 Center of Excellence in Genomic Medicine Research, King Abdulaziz University, Jeddah, Saudi Arabia; 388 KACST Technology Innovation Center in Personalized Medicine, King Abdulaziz University, Jeddah, Saudi Arabia; 389 Children Hospital, Genetics Department, Taif, Saudi Arabia; 390 Aziziah Maternity and Children Hospital, Jeddah, Saudi Arabia; 391 The Centre for Applied Genomics, The Hospital for Sick Children, Toronto, Canada; 392 King Fahd Medical Research Center, King Abdulaziz University, P.O. Box 80216, Jeddah, 21589 Saudi Arabia; 393 Department of Biophysics, All India Institute of Medical Sciences, New Delhi, 110029 India; 394 Children’s Hospital of Philadelphia, Philadelphia, PA 19104 USA; 395 Center of Excellence in Genomic Medicine Research, King Abdulaziz University, P.O. Box 80216, Jeddah, 21589 Saudi Arabia; 396 Center of Excellence in Genomic Medicine Research, King Abdulaziz University, Jeddah, Saudi Arabia; 397 Department of Biology, King Abdulaziz University, Jeddah, Saudi Arabia; 398 King Fahad Medical Research Center, King Abdulaziz University, Jeddah, Saudi Arabia; 399 KACST Technology Innovation Center in Personalized Medicine, King Abdulaziz University, Jeddah, Saudi Arabia; 400 Department of Pathology, Faculty of Medicine, King Abdulaziz University Hospital, Jeddah, Saudi Arabia; 401 Department of Pathology, King Faisal Specialist Hospital and Research Center, Jeddah, Saudi Arabia; 402 Division of Neurosurgery, Department of Surgery, King Abdulaziz University Hospital, Jeddah, Saudi Arabia; 403 Department of Biochemistry, Faculty of Science, King Abdulaziz University, Jeddah, Saudi Arabia; 404 Centre of Excellence in Genomic Medicine Research (CEGMR), King Abdulaziz University, Jeddah, Saudi Arabia; 405 Experimental Biochemistry Unit, King Fahd Medical Research Centre King, Abdulaziz University, Jeddah, Saudi Arabia; 406 Department of Medical Genetics, Umm Al-Qura University, Makkah, Saudi Arabia; 407 Science and Technology Unit, Umm Al-Qura University, Makkah, Saudi Arabia; 408 Department of Laboratory and Blood Bank, King Abdullah Medical City, Makkah, Saudi Arabia; 409 King Faisal Specialist Hospital and Research Centre, Riyadh, Saudi Arabia; 410 Center of Excellence in Genomic Medicine Research (CEGMR), King Abdulaziz University, Jeddah, Saudi Arabia; 411 Center of Innovation in Personalized Medicine, King Abdulaziz University, Jeddah, Saudi Arabia; 412 Gynecological Oncology Unit, Department of Obstetrics and Gynaecology, Faculty of Medicine, King Abdulaziz University Hospital, Jeddah, Saudi Arabia; 413 Laboratory of Biotechnology and Valorization of Bio/Geo Resources LR11ES31 High Institute of Biotechnology Sidi Thabet, University of Manouba, Biotechpole, Sidi Thabet, Tunisia; 414 Department of Gastroenterology, Hospital Mohamed Taher Maamouri, Nabeul, Tunisia; 415 Center of Excellence in Genomic Medicine Research, King Abdulaziz University, Jeddah, Saudi Arabia; 416 Center of Innovation in Personalized Medicine, King Fahd Medical Research Center, King Abdulaziz University, Jeddah, Saudi Arabia; 417 Biochemistry Department, Faculty of Science, King Fahd Medical Research Center, King Abdulaziz University, Jeddah, Saudi Arabia; 418 Production of Bioproducts for Industrial Applications Research Group, King Fahd Medical Research Center, King Abdulaziz University, Jeddah, Saudi Arabia; 419 Experimental Biochemistry Unit, King Fahd Medical Research Center, King Abdulaziz University, Jeddah, Saudi Arabia; 420 Center of Innovation in Personalized Medicine, King Abdulaziz University, Jeddah, Saudi Arabia; 421 Center of Excellence in Genomic medicine Research, King Abdulaziz University, Jeddah, Saudi Arabia; 422 Biology Department, Faculty of Science, King Abdulaziz University, Jeddah, Saudi Arabia; 423 Medical Laboratory Department, Faculty of Applied Medical Technology, King Abdulaziz University, Jeddah, Saudi Arabia; 424 Diagnostic Genomic Medicine Unit, Center of Excellence in Genomic Medicine Research, King Abdulaziz University, Jeddah, Saudi Arabia; 425 Center of Innovation in Personalized Medicine, King Abdulaziz University, Jeddah, Saudi Arabia; 426 Department of Biochemistry, King Abdulaziz University, Jeddah, Saudi Arabia; 427 Production of Bioproducts for Industrial Applications Research Group and Experimental Biochemistry Unit, King Fahd Medical Research Center, King Abdulaziz University, Jeddah, Saudi Arabia; 428 Microbial Biotechnology Department, National Research Center, Dokki, Cairo, Egypt; 429 Centre for Excellence in Genomic Medicine Research, King Abdulaziz University, Jeddah, Saudi Arabia; 430 Center of Excellence in Genomic Medicine, King Abdulaziz University, PO Box 80216, Jeddah, 21589 Saudi Arabia; 431 Institute for Genomics and Evolutionary Medicine, Temple University (SERC 602A), Philadelphia, Pennsylvania 19122 USA; 432 Center of Excellence in Genomic Medicine Research, King Abdulaziz University, Jeddah, Saudi Arabia; 433 Department of Biology, King Abdulaziz University, Jeddah, Saudi Arabia; 434 King Fahad Medical Research Center, King Abdulaziz University, Jeddah, Saudi Arabia; 435 Department of Pathology, Faculty of Medicine, King Abdulaziz University Hospital, Jeddah, Saudi Arabia; 436 Department of Pathology, King Faisal Specialist Hospital and Research Center, Jeddah, Saudi Arabia; 437 Division of Neurosurgery, Department of Surgery, King Abdulaziz University Hospital, Jeddah, Saudi Arabia; 438 KACST Technology Innovation Center in Personalized Medicine, King Abdulaziz University, Jeddah, Saudi Arabia; 439 Center of Excellence in Genomic Medicine Research, King Abdulaziz University, Jeddah, Saudi Arabia; 440 King Fahad Medical Research Center, King Abdulaziz University, Jeddah, Saudi Arabia; 441 KACST Technology Innovation Center in Personalized Medicine, King Abdulaziz University, Jeddah, Saudi Arabia; 442 Department of Pathology, Faculty of Medicine, King Abdulaziz University Hospital, Jeddah, Saudi Arabia; 443 Department of Pathology, King Faisal Specialist Hospital and Research Center, Jeddah, Saudi Arabia; 444 Division of Neurosurgery, Department of Surgery, King Abdulaziz University Hospital, Jeddah, Saudi Arabia; 445 Center of Excellence in Genomic Medicine Research, King Abdulaziz University, Jeddah, Saudi Arabia; 446 KACST Technology Innovation Center in Personalized Medicine, King Abdulaziz University, Jeddah, Saudi Arabia; 447 Department of Surgery, Faculty of Medicine, King Abdulaziz University, Jeddah, Saudi Arabia; 448 Department of Surgery, King Faisal Specialist Hospital and Research Center, Jeddah, Saudi Arabia; 449 Department of Pathology, King Faisal Specialist Hospital and Research Center, Jeddah, Saudi Arabia; 450 Department of Pathology, Faculty of Medicine, King Abdulaziz University, Jeddah, Saudi Arabia; 451 Center of Excellence in Genomic Medicine Research, King Abdulaziz University, Jeddah, Saudi Arabia; 452 KACST Technology Innovation Center in Personalized Medicine, King Abdulaziz University, Jeddah, Saudi Arabia; 453 Department of Surgery, Faculty of Medicine, King Abdulaziz University, Jeddah, Saudi Arabia; 454 Department of Surgery, King Faisal Specialist Hospital and Research Center, Jeddah, Saudi Arabia; 455 Department of Pathology, King Faisal Specialist Hospital and Research Center, Jeddah, Saudi Arabia; 456 Department of Pathology, Faculty of Medicine, King Abdulaziz University, Jeddah, Saudi Arabia; 457 Biology Department, Faculty of Science, King Abdulaziz University, Jeddah, Saudi Arabia; 458 Special Infectious Agents Unit, King Fahd Medical Research Center, King Abdulaziz University, Jeddah, Saudi Arabia; 459 Medical Laboratory Technology Department, Faculty of Applied Medical Sciences, King Abdulaziz University, Jeddah, Saudi Arabia; 460 Laboratory of Biotechnology and Valorization of Bio/Geo Resources LR11ES31 High Institute of Biotechnology Sidi Thabet, University of Manouba, Biotechpole Sidi Thabet, Sidi Thabet, Tunisia; 461 Department of Gastroenterology, Hospital Mohamed Taher Maamouri, Nabeul, Tunisia; 462 Laboratory of Soil Biology, University Neuchatel, Neuchatel, Switzerland; 463 Faculty of Medicine, King Abdulaziz University, Jeddah, Saudi Arabia; 464 Center of Innovation in Personalized Medicine, King Fahad Medical Research Center, King Abdulaziz University, Jeddah, Saudi Arabia; 465 Faculty of Science, King Abdulaziz University, Jeddah, Saudi Arabia; 466 Faculty of Applied Medical Sciences, King Abdulaziz University, Jeddah, Saudi Arabia; 467 Centre of Excellence in Genomic Medicine Research, King Abdulaziz University, Jeddah, Saudi Arabia; 468 Hôpital Necker-Enfants Malades-Université Paris descartes-Laboratoire de génétique, Paris, France; 469 Erfan & Bagedo Hospital, Jeddah, Saudi Arabia; 470 Aziziah Maternity and Children Hospital, Jeddah, Saudi Arabia; 471 Center of Excellence in Genomic Medicine Research (CEGMR), King Abdullaziz University, Jeddah, Saudi Arabia; 472 Department of Clinical Genetic and Metabolic Genetics, King Saud Medical Hospital, Riyadh, Saudi Arabia; 473 Department of Pathology, School of Medicine, Umm Al-Qura University, Mecca, Saudi Arabia; 474 King Fahd Medical Research Center, King Abdulaziz University, Jeddah, Saudi Arabia; 475 Faculty of Applied Medical Sciences, King Abdulaziz University, Jeddah, Saudi Arabia; 476 King Abdullah International Medical Research Center/King Saud Bin Abdulaziz University for Health Sciences, Ministry of National Guard Health Affairs, Riyad, Saudi Arabia; 477 Center of Excellence in Genomic Medicine Research, King Abdulaziz University, Jeddah, Saudi Arabia; 478 King Fahd Medical Research Center, King Abdulaziz University, Jeddah, Saudi Arabia; 479 Department of Biosciences, Jamia Milia Islamia, New Delhi, India; 480 Mohammad Ali Jauhar University, Rampur, UP India; 481 School of Medicine, University of Health Science, Lahore, Pakistan; 482 Center of Excellence in Genomic Medicine Research, King Abdulaziz University, Jeddah, Saudi Arabia; 483 Department of Biology, King Abdul-Aziz University, Jeddah, Saudi Arabia; 484 Center of Innovation in Personalized Medicine at King Abdulaziz University, Jeddah, Saudi Arabia; 485 King Fahd Medical Research Center, King Abdul Aziz University, Jeddah, Saudi Arabia; 486 King Fahd Medical Research Center, King Abdulaziz University, Jeddah, Kingdom of Saudi Arabia; 487 Centre of Excellence in Genomic Medicine Research, King Abdulaziz University, Jeddah, Kingdom of Saudi Arabia; 488 KACST Technology Innovation Center in Personalized Medicine, King Abdulaziz University, Jeddah, Kingdom of Saudi Arabia; 489 Department of Obstetrics and Gynecology, King Abdulaziz University Hospital, Jeddah, Kingdom of Saudi Arabia; 490 Medical Biotechnology and Translational Medicine Research, King Fahd Medical Research Center, King Abdulaziz University, PO Box 80216, Jeddah, 21589 Saudi Arabia; 491 Institute of Microbial Technology (IMTECH), Chandigarh, 160036 India; 492 Ummul Qura University, Makkah, Saudi Arabia; 493 Center of Excellence in Genomic Medicine Research, King Abdulaziz University, P.O. Box 80216, Jeddah, 21589 Saudi Arabia; 494 Department of Biochemistry, Faculty of Medicine, Umm Al-Qura University, Makkah, Kingdom of Saudi Arabia; 495 Department of Biochemistry, Faculty of Pharmacy, Al-Azhar University, 71524 Assiut, Egypt; 496 Institute of Pharmacy and Molecular Biotechnology, Heidelberg University, Im Neuenheimer Feld 364, 69120 Heidelberg, Germany; 497 Assisted Conception Unit, Obstetrics and Gynecology Department, College of Medicine, King Saud University, Riyadh, Kingdom of Saudi Arabia; 498 Urology Division, Surgery Department, College of Medicine, King Saud University, Riyadh, Kingdom of Saudi Arabia; 499 Toxicogenomics Laboratory, Division of Genetics, Department of Zoology, Aligarh Muslim University, Aligarh, India; 500 Centre of Excellence in Genomic and Medicine Research, King Abdulaziz University, Jeddah, Saudi Arabia

## Abstract

O1 Regulation of genes by telomere length over long distances

Jerry W. Shay

O2 The microtubule destabilizer KIF2A regulates the postnatal establishment of neuronal circuits in addition to prenatal cell survival, cell migration, and axon elongation, and its loss leading to malformation of cortical development and severe epilepsy

Noriko Homma, Ruyun Zhou, Muhammad Imran Naseer, Adeel G. Chaudhary, Mohammed Al-Qahtani, Nobutaka Hirokawa

O3 Integration of metagenomics and metabolomics in gut microbiome research

Maryam Goudarzi, Albert J. Fornace Jr.

O4 A unique integrated system to discern pathogenesis of central nervous system tumors

Saleh Baeesa, Deema Hussain, Mohammed Bangash, Fahad Alghamdi, Hans-Juergen Schulten, Angel Carracedo, Ishaq Khan, Hanadi Qashqari, Nawal Madkhali, Mohamad Saka, Kulvinder S. Saini, Awatif Jamal, Jaudah Al-Maghrabi, Adel Abuzenadah, Adeel Chaudhary, Mohammed Al Qahtani, Ghazi Damanhouri

O5 RPL27A is a target of miR-595 and deficiency contributes to ribosomal dysgenesis

Heba Alkhatabi

O6 Next generation DNA sequencing panels for haemostatic and platelet disorders and for Fanconi anaemia in routine diagnostic service

Anne Goodeve, Laura Crookes, Nikolas Niksic, Nicholas Beauchamp

O7 Targeted sequencing panels and their utilization in personalized medicine

Adel M. Abuzenadah

O8 International biobanking in the era of precision medicine

Jim Vaught

O9 Biobank and biodata for clinical and forensic applications

Bruce Budowle, Mourad Assidi, Abdelbaset Buhmeida

O10 Tissue microarray technique: a powerful adjunct tool for molecular profiling of solid tumors

Jaudah Al-Maghrabi

O11 The CEGMR biobanking unit: achievements, challenges and future plans

Abdelbaset Buhmeida, Mourad Assidi, Leena Merdad

O12 Phylomedicine of tumors

Sudhir Kumar, Sayaka Miura, Karen Gomez

O13 Clinical implementation of pharmacogenomics for colorectal cancer treatment

Angel Carracedo, Mahmood Rasool

O14 From association to causality: translation of GWAS findings for genomic medicine

Ahmed Rebai

O15 E-GRASP: an interactive database and web application for efficient analysis of disease-associated genetic information

Sajjad Karim, Hend F Nour Eldin, Heba Abusamra, Elham M Alhathli, Nada Salem, Mohammed H Al-Qahtani, Sudhir Kumar

O16 The supercomputer facility “AZIZ” at KAU: utility and future prospects

Hossam Faheem

O17 New research into the causes of male infertility

Ashok Agarwa

O18 The Klinefelter syndrome: recent progress in pathophysiology and management

Eberhard Nieschlag, Joachim Wistuba, Oliver S. Damm, Mohd A. Beg, Taha A. Abdel-Meguid, Hisham A. Mosli, Osama S. Bajouh, Adel M. Abuzenadah, Mohammed H. Al-Qahtani

O19 A new look to reproductive medicine in the era of genomics

Serdar Coskun

P1 Wnt signalling receptors expression in Saudi breast cancer patients

Muhammad Abu-Elmagd, Abdelbaset Buhmeida, Ashraf Dallol, Jaudah Al-Maghrabi, Sahar Hakamy, Wejdan Al-Qahtani, Asia Al-Harbi, Shireen Hussain, Mourad Assidi, Mohammed Al-Qahtani, Adel Abuzenadah

P2 Analysis of oxidative stress interactome during spermatogenesis: a systems biology approach to reproduction

Burak Ozkosem, Rick DuBois

P3 Interleukin-18 gene variants are strongly associated with idiopathic recurrent pregnancy loss.

Safia S Messaoudi, Maryam T Dandana, Touhami Mahjoub, Wassim Y Almawi

P4 Effect of environmental factors on gene-gene and gene-environment reactions: model and theoretical study applied to environmental interventions using genotype

S. Abdalla, M. Nabil Al-Aama

P5 Genomics and transcriptomic analysis of imatinib resistance in gastrointestinal stromal tumor

Asmaa Elzawahry, Tsuyoshi Takahashi, Sachiyo Mimaki, Eisaku Furukawa, Rie Nakatsuka, Isao Kurosaka, Takahiko Nishigaki, Hiromi Nakamura, Satoshi Serada, Tetsuji Naka, Seiichi Hirota, Tatsuhiro Shibata, Katsuya Tsuchihara, Toshirou Nishida, Mamoru Kato

P6 *In-Silico* analysis of putative HCV epitopes against Pakistani human leukocyte antigen background: an approach towards development of future vaccines for Pakistani population

Sajid Mehmood, Naeem Mahmood Ashraf, Awais Asif, Muhammad Bilal, Malik Siddique Mehmood, Aadil Hussain

P7 Inhibition of AChE and BuChE with the natural compounds of *Bacopa monerri* for the treatment of Alzheimer’s disease: a bioinformatics approach

Qazi Mohammad Sajid Jamal, Mughees Uddin Siddiqui, Mohammad A. Alzohairy, Mohammad A. Al Karaawi

P8 Her2 expression in urothelial cell carcinoma of the bladder in Saudi Arabia

Taoufik Nedjadi, Jaudah Al-Maghrabi, Mourad Assidi, Heba Al-Khattabi, Adel Al-Ammari, Ahmed Al-Sayyad, Abdelbaset Buhmeida, Mohammed Al-Qahtani

P9 Association of angiotensinogen single nucleotide polymorphisms with Preeclampsia in patients from North Africa

Hédia Zitouni, Nozha Raguema, Marwa Ben Ali, Wided Malah, Raja Lfalah, Wassim Almawi, Touhami Mahjoub

P10 Systems biology analysis reveals relations between normal skin, benign nevi and malignant melanoma

Mohammed Elanbari, Andrey Ptitsyn

P11 The apoptotic effect of thymoquinone in Jurkat cells

Sana Mahjoub, Rabeb El Ghali, Bechir Achour, Nidhal Ben Amor, Mourad Assidi, Brahim N'siri, Hamid Morjani

P12 Sonic hedgehog contributes in bladder cancer invasion in Saudi Arabia

Taoufik Nedjadi, Adel Al-Ammari, Ahmed Al-Sayyad, Nada Salem, Esam Azhar, Jaudah Al-Maghrabi

P13 Association of Interleukin 18 gene promoter polymorphisms - 607A/C and -137 G/C with colorectal cancer onset in a sample of Tunisian population

Vera Chayeb, Maryam Dendena, Hedia Zitouni, Khedija Zouari-Limayem, Touhami Mahjoub

P14 Pathological expression of interleukin-6, -11, leukemia inhibitory factor and their receptors in tubal gestation with and without tubal cytomegalovirus infection

Bassem Refaat, Ahmed M Ashshi, Sarah A Batwa

P15 Phenotypic and genetic profiling of avian pathogenic and human diarrhegenic *Escherichia coli* in Egypt

Hazem Ramadan, Amal Awad, Ahmed Ateya

P16 Cancer-targeting dual gene virotherapy as a promising therapeutic strategy for treatment of hepatocellular carcinoma

Adel Galal Ahmed El-Shemi, Ahmad Ashshi, Mohammed Basalamah, Youjin Na, Chae-Ok YUN

P17 Cancer dual gene therapy with oncolytic adenoviruses expressing TRAIL and IL-12 transgenes markedly eradicated human hepatocellular carcinoma both *in vitro* and *in vivo*

Adel Galal Ahmed El-Shemi, Ahmad Ashshi, Mohammed Basalamah, Youjin Na, Chae-Ok Yun

P18 Therapy with paricalcitol attenuates tumor growth and augments tumoricidal and anti-oncogenic effects of 5-fluorouracil on animal model of colon cancer

Adel Galal El-Shemi, Bassem Refaat, Osama Kensara, Amr Abdelfattah

P19 The effects of *Rubus idaeus* extract on normal human lymphocytes and cancer cell line

Batol Imran Dheeb, Mohammed M. F. Al-Halbosiy, Rghad Kadhim Al lihabi, Basim Mohammed Khashman

P20 Etanercept, a TNF-alpha inhibitor, alleviates mechanical hypersensitivity and spontaneous pain in a rat model of chemotherapy-induced neuropathic pain

Djouhri, Laiche, Chaudhary Adeel, Nedjadi, Taoufik

P21 Sleeping beauty mutagenesis system identified genes and neuronal transcription factor network involved in pediatric solid tumour (medulloblastoma)

Hani Al-Afghani, Maria Łastowska, Haya H Al-Balool, Harsh Sheth, Emma Mercer, Jonathan M Coxhead, Chris PF Redfern, Heiko Peters, Alastair D Burt, Mauro Santibanez-Koref, Chris M Bacon, Louis Chesler, Alistair G Rust, David J Adams, Daniel Williamson, Steven C Clifford, Michael S Jackson

P22 Involvement of interleukin-1 in vitiligo pathogenesis

Mala Singh, Mohmmad Shoab Mansuri, Shahnawaz D. Jadeja, Hima Patel, Yogesh S. Marfatia, Rasheedunnisa Begum

P23 Cytogenetics abnormalities in 12,884 referred population for chromosomal analysis and the role of FISH in refining the diagnosis (cytogenetic experience 2004-2013)

Amal M Mohamed, Alaa K Kamel, Nivin A Helmy, Sayda A Hammad, Hesham F Kayed, Marwa I Shehab, Assad El Gerzawy, Maha M. Ead, Ola M Ead, Mona Mekkawy, Innas Mazen, Mona El-Ruby

P24 Analysis of binding properties of angiotensin-converting enzyme 2 through *in silico* method

S. M. A. Shahid, Qazi Mohammad Sajid Jamal, J. M. Arif, Mohtashim Lohani

P25 Relationship of genetics markers *cis* and *trans* to the β-S globin gene with fetal hemoglobin expression in Tunisian sickle cell patients

Moumni Imen, Chaouch Leila, Ouragini Houyem, Douzi Kais, Chaouachi Dorra Mellouli Fethi, Bejaoui Mohamed, Abbes Salem

P26 Analysis of estrogen receptor alpha gene polymorphisms in breast cancer: link to genetic predisposition in Sudanese women

Areeg Faggad, Amanuel T Gebreslasie, Hani Y Zaki, Badreldin E Abdalla

P27 KCNQI gene polymorphism and its association with CVD and T2DM in the Saudi population

Maha S AlShammari, Rhaya Al-Ali, Nader Al-Balawi , Mansour Al-Enazi, Ali Al-Muraikhi, Fadi Busaleh, Ali Al-Sahwan, Francis Borgio, Abdulazeez Sayyed, Amein Al-Ali, Sadananda Acharya

P28 Clinical, neuroimaging and cytogenetic study of a patient with microcephaly capillary malformation syndrome

Maha S. Zaki, Hala T. El-Bassyouni, Marwa I. Shehab

P29 Altered expression of CD200R1 on dendritic cells of patients with inflammatory bowel diseases: *in silico* investigations and clinical evaluations

Mohammed F. Elshal, Kaleemuddin M., Alia M. Aldahlawi, Omar Saadah,

J. Philip McCoy

P30 Development of real time PCR diagnostic protocol specific for the Saudi Arabian H1N1 viral strains

Adel E El-Tarras, Nabil S Awad, Abdulla A Alharthi, Mohamed M M Ibrahim

P31 Identification of novel genetic variations affecting Osteoarthritis patients

Haneen S Alsehli, Ashraf Dallol, Abdullah M Gari, Mohammed M Abbas, Roaa A Kadam, Mazen M. Gari, Mohmmed H Alkaff, Adel M Abuzenadah, Mamdooh A Gari

P32 An integrated database of GWAS SNVs and their evolutionary properties

Heba Abusamra, Sajjad Karim, Hend F Nour eldin, Elham M Alhathli, Nada Salem, Sudhir Kumar, Mohammed H Al-Qahtani

P33 Familial hypercholesterolemia in Saudi Arabia: prime time for a national registry and genetic analysis

Fatima A. Moradi, Omran M. Rashidi, Zuhier A. Awan

P34 Comparative genomics and network-based analyses of early hepatocellular carcinoma

Ibrahim Hamza Kaya, Olfat Al-Harazi, Dilek Colak

P35 A TALEN-based oncolytic viral vector approach to knock out ABCB1 gene mediated chemoresistance in cancer stem cells

Nabila A Alkousi, Takis Athanasopoulos

P36 Cartilage differentiation and gene expression of synovial fluid mesenchymal stem cells derived from osteoarthritis patients

Afnan O Bahmaid, Etimad A Alhwait, Mamdooh A Gari, Haneen S Alsehli, Mohammed M Abbas, Mohammed H Alkaf, Roaa Kadam, Ashraf Dallol, Gauthaman Kalamegam

P37 E-GRASP: Adding an evolutionary component to the genome-wide repository of associations (GRASP) resource

Hend F Nour Eldin, Sajjad Karim, Heba Abusamra, Elham Alhathli, Nada Salem, Mohammed H Al-Qahtani, Sudhir Kumar

P38 Screening of *AGL* gene mutation in Saudi family with glycogen storage disease Type III

Salma N Alsayed, Fawziah H Aljohani, Samaher M Habeeb, Rawan A Almashali, Sulman Basit, Samia M Ahmed

P39 High throughput proteomic data suggest modulation of cAMP dependent protein kinase A and mitochondrial function in infertile patients with varicocele

Rakesh Sharma, Ashok Agarwal, Damayanthi Durairajanayagam, Luna Samanta, Muhammad Abu-Elmagd, Adel M. Abuzenadah, Edmund S. Sabanegh, Mourad Assidi, Mohammed Al-Qahtani

P40 Significant protein profile alterations in men with primary and secondary infertility

Ashok Agarwal, Rakesh Sharma, Luna Samanta, Damayanthi Durairajanayagam, Mourad Assidi, Muhammad Abu-Elmagd, Mohammed Al-Qahtani, Adel M. Abuzenadah, Edmund S. Sabanegh

P41 Spermatozoa maturation in infertile patients involves compromised expression of heat shock proteins

Luna Samanta, Ashok Agarwal, Rakesh Sharma, Zhihong Cui, Mourad Assidi, Adel M. Abuzenadah, Muhammad Abu-Elmagd, Mohammed Al-Qahtani

P42 Array comparative genomic hybridization approach to search genomic answers for spontaneous recurrent abortion in Saudi Arabia

Alaa A Alboogmi, Nuha A Alansari, Maha M Al-Quaiti, Fai T Ashgan, Afnan Bandah, Hasan S Jamal, Abdullraheem Rozi_,_ Zeenat Mirza, Adel M Abuzenadah, Sajjad Karim, Mohammed H Al-Qahtani

P43 Global gene expression profiling of Saudi kidney cancer patients

Sajjad Karim, Hans-Juergen Schulten, Ahmad J Al Sayyad, Hasan MA Farsi, Jaudah A Al-Maghrabi, Zeenat Mirza, Reem Alotibi, Alaa Al-Ahmadi, Nuha A Alansari, Alaa A Albogmi, Maha M Al-Quaiti, Fai T Ashgan, Afnan Bandah, Mohammed H Al-Qahtani

P44 Downregulated StAR gene and male reproductive dysfunction caused by nifedipine and ethosuximide

Rasha A Ebiya, Samia M Darwish, Metwally M. Montaser

P45 Clustering based gene expression feature selection method: A computational approach to enrich the classifier efficiency of differentially expressed genes

Heba Abusamra, Vladimir B. Bajic

P46 Prognostic significance of Osteopontin expression profile in colorectal carcinoma

Jaudah Al-Maghrabi, Wafaey Gomaa, Mehenaz Hanbazazh, Mahmoud Al-Ahwal, Asia Al-Harbi, Wejdan Al-Qahtani, Saher Hakamy, Ghali Baba, Abdelbaset Buhmeida, Mohammed Al-Qahtani

P47 High Glypican-3 expression pattern predicts longer disease-specific survival in colorectal carcinoma

Jaudah Al-Maghrabi, Abdullah Al-Harbi, Mahmoud Al-Ahwal, Asia Al-Harbi, Wejdan Al-Qahtani, Sahar Hakamy, Ghalia Baba, Abdelbaset Buhmeida, Mohammed Al-Qahtani

P48 An evolutionary re-assessment of GWAS single nucleotide variants implicated in the Cholesterol traits

Elham M Alhathli, Sajjad Karim, Nada Salem, Hend Nour Eldin, Heba Abusamra, Sudhir Kumar, Mohammed H Al-Qahtani

P49 Derivation and characterization of human Wharton’s jelly stem cells (hWJSCs) *in vitro* for future therapeutic applications

Aisha A Alyamani, Gauthaman Kalamegam, Etimad A Alhwait, Mamdooh A Gari, Mohammed M Abbas, Mohammed H Alkaf, Haneen S Alsehli, Roaa A Kadam, Mohammed Al-Qahtani

P50 Attitudes of healthcare students toward biomedical research in the post-genomic era

Rawan Gadi, Abdelbaset Buhmeida, Mourad Assidi , Adeel Chaudhary, Leena Merdad

P51 Evaluation of the immunomodulatory effects of thymoquinone on human bone marrow mesenchymal stem cells (BM-MSCs) from osteoarthritic patients

Saadiah M Alfakeeh, Etimad A Alhwait, Mamdooh A Gari, Mohammed M Abbas, Mohammed H Alkaf, Haneen S Alsehli, Roaa Kadam, Gauthaman Kalamegam

P52 Implication of IL-10 and IL-28 polymorphism with successful anti-HCV therapy and viral clearance

Rubi Ghazala, Shilu Mathew, M.Haroon Hamed, Mourad Assidi, Mohammed Al-Qahtani, Ishtiaq Qadri

P53 Selection of flavonoids against obesity protein (FTO) using *in silico* and *in vitro* approaches

Shilu Mathew, Lobna Mira, Manal Shaabad, Shireen Hussain, Mourad Assidi, Muhammad Abu-Elmagd, Mohammed Al-Qahtani

P54 Computational selection and *in vitro* validation of flavonoids as new antidepressant agents

Shilu Mathew, Manal Shaabad, Lobna Mira, Shireen Hussain, Mourad Assidi, Muhammad Abu-Elmagd, Mohammed Al-Qahtani

P55 *In Silico* prediction and prioritization of aging candidate genes associated with

progressive telomere shortening

Ahmed Rebai, Mourad Assidi, Abdelbaset Buhmeida, Muhammad Abu-Elmagd, Ashraf Dallol, Jerry W Shay

P56 Identification of new cancer testis antigen genes in diverse types of malignant human tumour cells

Mikhlid H Almutairi

P57 More comprehensive forensic genetic marker analyses for accurate human remains identification using massively parallel sequencing (MPS)

Angie Ambers, Jennifer Churchill, Jonathan King, Monika Stoljarova, Harrell Gill-King, Mourad Assidi, Muhammad Abu-Elmagd, Abdelbaset Buhmeida, Muhammad Al-Qatani, Bruce Budowle

P58 Flow cytometry approach towards treatment men infertility in Saudi Arabia

Muhammad Abu-Elmagd, Farid Ahmed, Ashraf Dallol, Mourad Assidi, Taha Abo Almagd, Sahar Hakamy, Ashok Agarwal, Muhammad Al-Qahtani, Adel Abuzenadah

P59 Tissue microarray based validation of CyclinD1 expression in renal cell carcinoma of Saudi kidney patients

Sajjad Karim, Hans-Juergen Schulten, Ahmad J Al Sayyad, Hasan MA Farsi, Jaudah A Al-Maghrabi, Abdelbaset Buhmaida, Zeenat Mirza, Reem Alotibi, Alaa Al-Ahmadi, Nuha A Alansari, Alaa A Albogmi, Maha M Al-Quaiti, Fai T Ashgan, Afnan Bandah, Mohammed H Al-Qahtani

P60 Assessment of gold nanoparticles in molecular diagnostics and DNA damage studies

Rukhsana Satar, Mahmood Rasool, Waseem Ahmad, Nazia Nazam, Mohamad I Lone, Muhammad I Naseer, Mohammad S Jamal, Syed K Zaidi, Peter N Pushparaj, Mohammad A Jafri, Shakeel A Ansari, Mohammed H Alqahtani

P61 Surfing the biospecimen management and processing workflow at CEGMR Biobank

Hanan Bashier, Abrar Al Qahtani, Shilu Mathew, Amal M. Nour, Heba Alkhatabi, Adel M. Abu Zenadah, Abdelbaset Buhmeida, Mourad Assidi, Muhammed Al Qahtani

P62 Autism Spectrum Disorder: knowledge, attitude and awareness in Jeddah, Kingdom of Saudi Arabia

Muhammad Faheem, Shilu Mathew, Shiny Mathew, Peter Natesan Pushparaj, Mohammad H. Al-Qahtani

P63 Simultaneous genetic screening of the coagulation pathway genes using the Thromboscan targeted sequencing panel

Hani A. Alhadrami, Ashraf Dallol, Adel Abuzenadah

P64 Genome wide array comparative genomic hybridization analysis in patients with syndromic congenital heart defects

Ibtessam R. Hussein, Adeel G. Chaudhary, Rima S Bader, Randa Bassiouni, Maha Alquaiti, Fai Ashgan, Hans Schulten, Mohamed Nabil Alama, Mohammad H. Al Qahtani

P65 Toxocogenetic evaluation of 1, 2-Dichloroethane in bone marrow, blood and cells of immune system using conventional, molecular and flowcytometric approaches

Mohammad I Lone, Nazia Nizam, Waseem Ahmad, Mohammad A Jafri, Mahmood Rasool, Shakeel A Ansari, Muhammed H Al-Qahtani

P66 Molecular cytogenetic diagnosis of sexual development disorders in newborn: A case of ambiguous genitalia

Eradah Alshihri, Muhammad Abu-Elmagd, Lina Alharbi, Mourad Assidi, Mohammed Al-Qahtani

P67 Identification of disease specific gene expression clusters and pathways in hepatocellular carcinoma using *In Silico* methodologies

Shilu Mathew, Peter Pushparaj Natesan, Muhammed Al Qahtani

P68 Human Wharton’s Jelly stem cell conditioned medium inhibits primary ovarian cancer cells *in vitro:* Identification of probable targets and mechanisms using systems biology

Gauthaman Kalamegam, Peter Natesan Pushparaj, Fazal Khan, Roaa Kadam, Farid Ahmed, Mourad Assidi, Khalid Hussain Wali Sait, Nisreen Anfinan, Mohammed Al Qahtani

P69 Mutation spectrum of ASPM (Abnormal Spindle-like, Microcephaly-associated) gene in Saudi Arabian population

Muhammad I Naseer, Adeel G Chaudhary, Mohammad S Jamal, Shilu Mathew, Lobna S Mira, Peter N Pushparaj, Shakeel A Ansari, Mahmood Rasool, Mohammed H AlQahtani

P70 Identification and characterization of novel genes and mutations of primary microcephaly in Saudi Arabian population

Muhammad I Naseer, Adeel G Chaudhary, Shilu Mathew, Lobna S Mira, Mohammad S Jamal, Sameera Sogaty, Randa I Bassiouni, Mahmood Rasool, Mohammed H AlQahtani

P71 Molecular genetic analysis of hereditary nonpolyposis colorectal cancer (Lynch Syndrome) in Saudi Arabian population

Mahmood Rasool, Shakeel A Ansari, Mohammad S Jamal, Peter N Pushparaj, Abdulrahman MS Sibiani, Waseem Ahmad, Abdelbaset Buhmeida, Mohammad A Jafri, Mohiuddin K Warsi, Muhammad I Naseer, Mohammed H Al-Qahtani

P72 Function predication of hypothetical proteins from genome database of chlamydia trachomatis

Rubi, Kundan Kumar, Ahmad AT Naqvi, Faizan Ahmad, Md I Hassan, Mohammad S Jamal, Mahmood Rasool, Mohammed H AlQahtani

P73 Transcription factors as novel molecular targets for skin cancer

Ashraf Ali, Jummanah Jarullah, Mahmood Rasool, Abdelbasit Buhmeida, Shahida Khan, Ghufrana Abdussami, Maryam Mahfooz, Mohammad A Kamal, Ghazi A Damanhouri, Mohammad S Jamal

P74 An *In Silico* analysis of Plumbagin binding to apoptosis executioner: Caspase-3 and Caspase-7

Bushra Jarullah, Jummanah Jarullah, Mohammad SS Jarullah, Ashraf Ali, Mahmood Rasool, Mohammad S Jamal

P75 Single cell genomics applications for preimplantation genetic screening optimization: Comparative analysis of whole genome amplification technologies

Mourad Assidi, Muhammad Abu-Elmagd, Osama Bajouh, Peter Natesan Pushparaj, Mohammed Al-Qahtani, Adel Abuzenadah

P76 ZFP36 regulates miRs-34a in anti-IgM triggered immature B cells

Mohammad S Jamal, Jummanah Jarullah, Abdulah EA Mathkoor, Hashim MA Alsalmi, Anas MM Oun, Ghazi A Damanhauri, Mahmood Rasool, Mohammed H AlQahtani

P77 Identification of a novel mutation in the *STAMBP* gene in a family with microcephaly-capillary malformation syndrome

Muhammad I. Naseer, Mahmood Rasool, Sameera Sogaty, Adeel G. Chudhary, Yousif A. Abutalib, Daniele Merico, Susan Walker, Christian R. Marshall, Mehdi Zarrei, Stephen W. Scherer, Mohammad H. Al-Qahtani

P78 Copy number variations in Saudi patients with intellectual disability and epilepsy

Muhammad I. Naseer, Muhammad Faheem, Adeel G. Chaudhary, Mahmood Rasool, Gauthaman Kalamegam, Fai Talal Ashgan, Mourad Assidi, Farid Ahmed, Syed Kashif Zaidi, Mohammed M. Jan, Mohammad H. Al-Qahtani

P79 Prognostic significance of CD44 expression profile in colorectal carcinoma

Maryam Al-Zahrani, Sahira Lary, Sahar Hakamy, Ashraf Dallol, Mahmoud Al-Ahwal, Jaudah Al-Maghrabi, Emmanuel Dermitzakis, Adel Abuzenadah, Abdelbaset Buhmeida, Mohammed Al-Qahtani

P80 Association of the endothelial nitric oxide synthase (eNOS) gene G894T polymorphism with hypertension risk and complications

Abeer A Al-refai, Mona Saleh, Rehab I Yassien, Mahmmoud Kamel, Rabab M Habeb

P81 SNPs array to screen genetic variation among diabetic patients

Najlaa Filimban, Ashraf Dallol, Nadia Ghannam, Mohammed Al-Qahtani, Adel Mohammed Abuzenadah

P82 Detection and genotyping of *Helicobacter pylori* among gastric cancer patients from Saudi Arabian population

Fehmida Bibi, Sana Akhtar, Esam I. Azhar, Muhammad Yasir, Muhammad I. Nasser, Asif A. Jiman-Fatani, Ali Sawan

P83 Antimicrobial drug resistance and molecular detection of susceptibility to Fluoroquinolones among clinical isolates of Salmonella species from Jeddah-Saudi Arabia

Ruaa A Lahzah, Asho Ali

P84 Identification of the toxic and virulence nature of MAP1138c protein of *Mycobacterium avium* subsp. *paratuberculosis*

Syed A Hassan, Seyed E Hasnain, Iftikhar A Tayubi, Hamza A Abujabal, Alaa O Magrabi

P85 *In vitro* and *in silico* evaluation of miR137 in human breast cancer

Fazal Khan, Gauthaman Kalamegam, Peter Natesan Pushparaj, Adel Abuzenada, Taha Abduallah Kumosani, Elie Barbour, Mohammed Al-Qahtani

P86 Auruka gene is over-expressed in Saudi breast cancer

Manal Shabaad, Shilu Mathew, Ashraf Dallol, Adnan Merdad, Abdelbaset Buhmeida, Mohammed Al-Qahtani

P87 The potential of immunogenomics in personalized healthcare

Mourad Assidi, Muhammad Abu-Elmagd, Kalamegam Gauthaman, Mamdooh Gari, Adeel Chaudhary, Adel Abuzenadah, Peter Natesan Pushparaj, Mohammed Al-Qahtani

P88 *In Silico* physiochemical and structural characterization of a putative ORF MAP0591 and its implication in the pathogenesis of *Mycobacterium paratuberculosis* in ruminants and humans

Syed A Hassan, Iftikhar A Tayubi, Hani MA Aljahdali

P89 Effects of heat shock on human bone marrow mesenchymal stem cells (BM-MSCs): Implications in regenerative medicine

Reham Al Nono, Mamdooh Gari, Haneen Alsehli, Farid Ahmed, Mohammed Abbas, Gauthaman Kalamegam, Mohammed Al-Qahtani

P90 *In Silico* analyses of the molecular targets of Resveratrol unravels its importance in mast cell mediated allergic responses

Shilu Mathew, Fazal Khan, Mahmood Rasool, Mohammed Sarwar Jamal, Muhammad Imran Naseer, Zeenat Mirza, Sajjad Karim, Shakeel Ansari, Mourad Assidi, Gauthaman Kalamegam, Mamdooh Gari, Adeel Chaudhary, Adel Abuzenadah, Peter Natesan Pushparaj, Mohammed Al-Qahtani

P91 Effects of environmental particulate matter on bone-marrow mesenchymal stem cells

Muhammad Abu-Elmagd, Gauthaman Kalamegam, Roaa Kadam, Mansour A Alghamdi, Magdy Shamy, Max Costa, Mamdouh I Khoder, Mourad Assidi, Peter Natesan Pushparaj, Mamdooh Gari, Mohammed Al-Qahtani

P92 Distinctive charge clusters in human virus proteomes

Najla Kharrat, Sabrine Belmabrouk, Rania Abdelhedi, Riadh Benmarzoug, Mourad Assidi, Mohammed H. Al Qahtani, Ahmed Rebai

P93 *In vitro* experimental model and approach in identification of new biomarkers of inflammatory forms of arthritis

Ghazi Dhamanhouri, Peter Natesan Pushparaj, Abdelwahab Noorwali, Mohammad Khalid Alwasiyah, Afnan Bahamaid, Saadiah Alfakeeh, Aisha Alyamani, Haneen Alsehli, Mohammed Abbas, Mamdooh Gari, Ali Mobasheri, Gauthaman Kalamegam, Mohammed Al-Qahtani

P94 Molecular docking of GABA_A_ receptor subunit γ-2 with novel anti-epileptic compounds

Muhammad Faheem, Shilu Mathew, Peter Natesan Pushparaj, Mohammad H. Al-Qahtani

P95 Breast cancer knowledge, awareness, and practices among Saudi females residing in Jeddah

Shilu Mathew, Muhammad Faheem, Shiny Mathew, Peter Natesan Pushparaj, Mohammad H. Al-Qahtani

P96 Anti-inflammatory role of Sesamin by Attenuation of Iba1/TNF-α/ICAM-1/iNOS signaling in Diabetic Retinopathy

Mohammad Sarwar Jamal, Syed Kashif Zaidi, Raziuddin Khan, Kanchan Bhatia, Mohammed H. Al-Qahtani, Saif Ahmad

P97 Identification of drug lead molecule against vp35 protein of Ebola virus: An *In-Silico* approach

Iftikhar AslamTayubi, Manish Tripathi, Syed Asif Hassan, Rahul Shrivastava

P98 An approach to personalized medicine from SNP-calling through disease analysis using whole exome-sequencing of three sub-continental populations

Iftikhar A Tayubi, Syed Hassan, Hamza A.S Abujabal

P99 Low versus high frequency of Glucose –6 – Phosphate Dehydrogenase (G6PD) deficiency in urban against tribal population of Gujarat – A signal to natural selection

Ishani Shah, Bushra Jarullah, Mohammad S Jamal, Jummanah Jarullah

P100 Spontaneous preterm birth and single nucleotide gene polymorphisms: a recent update

Ishfaq A Sheikh, Ejaz Ahmad, Mohammad S Jamal, Mohd Rehan, Muhammad Abu-Elmagd, Iftikhar A Tayubi, Samera F AlBasri, Osama S Bajouh, Rola F Turki, Adel M Abuzenadah, Ghazi A Damanhouri, Mohd A Beg, Mohammed Al-Qahtani

P101 Prevalence of congenital heart diseases among Down syndrome cases in Saudi Arabia: role of molecular genetics in the pathogenesis

Sahar AF Hammoudah, Khalid M AlHarbi, Lama M El-Attar, Ahmed MZ Darwish

P102 Combinatorial efficacy of specific pathway inhibitors in breast cancer cells

Sara M Ibrahim, Ashraf Dallol_,_ Hani Choudhry, Adel Abuzenadah, Jalaludden Awlia, Adeel Chaudhary, Farid Ahmed, Mohammed Al-Qahtani

P103 MiR-143 and miR-145 cluster as potential replacement medicine for the treatment of cancer

Mohammad A Jafri, Muhammad Abu-Elmagd, Mourad Assidi, Mohammed Al-Qahtani

P104 Metagenomic profile of gut microbiota during pregnancy in Saudi population

Imran khan, Muhammad Yasir, Esam I. Azhar, Sameera Al-basri, Elie Barbour, Taha Kumosani

P105 Exploration of anticancer targets of selected metabolites of *Phoenix dactylifera L.* using systems biological approaches

Fazal Khan, Gauthaman Kalamegam, Peter Natesan Pushparaj, Adel Abuzenada, Taha Abduallah Kumosani, Elie Barbour

P106 CD226 and CD40 gene polymorphism in susceptibility to Juvenile rheumatoid arthritis in Egyptian patients

Heba M. EL Sayed, Eman A. Hafez

P107 Paediatric exome sequencing in autism spectrum disorder ascertained in Saudi families

Hans-Juergen Schulten, Aisha Hassan Elaimi, Ibtessam R Hussein, Randa Ibrahim Bassiouni, Mohammad Khalid Alwasiyah, Richard F Wintle, Adeel Chaudhary, Stephen W Scherer, Mohammed Al-Qahtani

P108 Crystal structure of the complex formed between Phospholipase A_2_ and the central core hydrophobic fragment of Alzheimer’s β- amyloid peptide: a reductionist approach

Zeenat Mirza, Vikram Gopalakrishna Pillai, Sajjad Karim, Sujata Sharma, Punit Kaur, Alagiri Srinivasan, Tej P Singh, Mohammed Al-Qahtani

P109 Differential expression profiling between meningiomas from female and male patients

Reem Alotibi, Alaa Al-Ahmadi, Fatima Al-Adwani, Deema Hussein, Sajjad Karim, Mona Al-Sharif, Awatif Jamal, Fahad Al-Ghamdi, Jaudah Al-Maghrabi, Saleh S Baeesa, Mohammed Bangash, Adeel Chaudhary, Hans-Juergen Schulten, Mohammed Al-Qahtani

P110 Neurospheres as models of early brain development and therapeutics

Muhammad Faheem, Peter Natesan Pushparaj, Shilu Mathew, Taha Abdullah Kumosani, Gauthaman Kalamegam, Mohammed Al-Qahtani

P111 Identification of a recurrent causative missense mutation p.(W577C) at the LDLR exon 12 in familial hypercholesterolemia affected Saudi families

Faisal A Al-Allaf, Zainularifeen Abduljaleel, Abdullah Alashwal, Mohiuddin M. Taher, Abdellatif Bouazzaoui, Halah Abalkhail, Faisal A. Ba-Hammam, Mohammad Athar

P112 Epithelial ovarian carcinoma (EOC): Systems oncological approach to identify diagnostic, prognostic and therapeutic biomarkers

Gauthaman Kalamegam, Peter Natesan Pushparaj, Muhammad Abu-Elmagd, Farid Ahmed Khalid HussainWali Sait, Nisreen Anfinan, Mamdooh Gari, Adeel Chaudhary, Adel Abuzenadah, Mourad Assidi, Mohammed Al-Qahtani

P113 Crohn’s disease phenotype in northern Tunisian population

Naira Ben Mami, Yosr Z Haffani, Mouna Medhioub, Lamine Hamzaoui, Ameur Cherif, Msadok Azouz

P114 Establishment of *In Silico* approaches to decipher the potential toxicity and mechanism of action of drug candidates and environmental agents

Gauthaman Kalamegam, Fazal Khan, Shilu Mathew, Mohammed Imran Nasser, Mahmood Rasool, Farid Ahmed, Peter Natesan Pushparaj, Mohammed Al-Qahtani

P115 1q Gain predicts poor prognosis marker for young breast cancer patients

Shereen A Turkistany, Lina M Al-harbi, Ashraf Dallol, Jamal Sabir, Adeel Chaudhary, Adel Abuzenadah

P116 Disorders of sex chromosomes in a diagnostic genomic medicine unit in Saudi Arabia: Prevalence, diagnosis and future guidelines

Basmah Al-Madoudi, Bayan Al-Aslani, Khulud Al-Harbi, Rwan Al-Jahdali, Hanadi Qudaih, Emad Al Hamzy, Mourad Assidi, Mohammed Al Qahtani

P117 Combination of WYE354 and Sunitinib demonstrate synergistic inhibition of acute myeloid leukemia *in vitro*

Asad M Ilyas, Youssri Ahmed, Mamdooh Gari, Farid Ahmed, Mohammed Alqahtani

P118 Integrated use of evolutionary information in GWAS reveals important SNPs in Asthma

Nada Salem, Sajjad Karim, Elham M Alhathli, Heba Abusamra, Hend F Nour Eldin, Mohammed H Al-Qahtani, Sudhir Kumar

P119 Assessment of *BRAF*, *IDH1*, *IDH2*, and *EGFR* mutations in a series of primary brain tumors

Fatima Al-Adwani, Deema Hussein, Mona Al-Sharif, Awatif Jamal, Fahad Al-Ghamdi, Jaudah Al-Maghrabi, Saleh S Baeesa, Mohammed Bangash, Adeel Chaudhary, Mohammed Al-Qahtani, Hans-Juergen Schulten

P120 Expression profiles distinguish oligodendrogliomas from glioblastoma multiformes with or without oligodendroglioma component

Alaa Alamandi, Reem Alotibi, Deema Hussein, Sajjad Karim, Jaudah Al-Maghrabi, Fahad Al-Ghamdi, Awatif Jamal, Saleh S Baeesa, Mohammed Bangash, Adeel Chaudhary, Hans-Juergen Schulten, Mohammed Al-Qahtani

P121 Hierarchical clustering in thyroid goiters and hyperplastic lesions

Ohoud Subhi, Nadia Bagatian, Sajjad Karim, Adel Al-Johari, Osman Abdel Al-Hamour, Hosam Al-Aradati, Abdulmonem Al-Mutawa, Faisal Al-Mashat, Jaudah Al-Maghrabi, Hans-Juergen Schulten, Mohammad Al-Qahtani

P122 Differential expression analysis in thyroiditis and papillary thyroid carcinomas with or without coexisting thyroiditis

Nadia Bagatian, Ohoud Subhi, Sajjad Karim, Adel Al-Johari, Osman Abdel Al-Hamour, Abdulmonem Al-Mutawa, Hosam Al-Aradati, Faisal Al-Mashat, Mohammad Al-Qahtani, Hans-Juergen Schulten, Jaudah Al-Maghrabi

P123 Metagenomic analysis of waste water microbiome in Sausdi Arabia

Muhammad W shah, Muhammad Yasir, Esam I Azhar, Saad Al-Masoodi

P124 Molecular characterization of *Helicobacter pylori* from faecal samples of Tunisian patients with gastric cancer

Yosr Z Haffani, Msadok Azouz, Emna Khamla, Chaima Jlassi, Ahmed S. Masmoudi, Ameur Cherif, Lassaad Belbahri

P125 Diagnostic application of the oncoscan^©^ panel for the identification of hereditary cancer syndrome

Shadi Al-Khayyat, Roba Attas, Atlal Abu-Sanad, Mohammed Abuzinadah, Adnan MerdadAshraf Dallol, Adeel Chaudhary, Mohammed Al-Qahtani, Adel Abuzenadah

P126 Characterization of clinical and neurocognitive features in a family with a novel *OGT* gene missense mutation c. 1193G > A/ (p. Ala319Thr)

Habib Bouazzi, Carlos Trujillo, Mohammad Khalid Alwasiyah, Mohammed Al-Qahtani

P127 Case report: a rare homozygous deletion mutation of *TMEM70* gene associated with 3-Methylglutaconic Aciduria and cataract in a Saudi patient

Maha Alotaibi, Rami Nassir

P128 Isolation and purification of antimicrobial milk proteins

Ishfaq A Sheikh, Mohammad A Kamal, Essam H Jiffri, Ghulam M Ashraf, Mohd A Beg

P129 Integrated analysis reveals association of ATP8B1 gene with colorectal cancer

Mohammad A Aziz, Rizwan Ali, Mahmood Rasool, Mohammad S Jamal, Nusaibah samman, Ghufrana Abdussami, Sathish Periyasamy, Mohiuddin K Warsi, Mohammed Aldress, Majed Al Otaibi, Zeyad Al Yousef, Mohamed Boudjelal, Abdelbasit Buhmeida, Mohammed H Al-Qahtani, Ibrahim AlAbdulkarim

P130 Implication of IL-10 and IL-28 polymorphism with successful anti-HCV therapy and viral clearance

Rubi Ghazala, Shilu Mathew, M. Haroon Hamed, Mourad Assidi, Mohammed Al-Qahtani, Ishtiaq Qadri

P131 Interactions of endocrine disruptor di-(2-ethylhexyl) phthalate (DEHP) and its metabolite mono-2-ethylhexyl phthalate (MEHP) with progesterone receptor

Ishfaq A Sheikh, Muhammad Abu-Elmagd, Rola F Turki, Ghazi A Damanhouri, Mohd A. Beg

P132 Association of HCV nucleotide polymorphism in the development of hepatocellular carcinoma

Mohd Suhail, Abid Qureshi, Adil Jamal, Peter Natesan Pushparaj, Mohammad Al-Qahtani, Ishtiaq Qadri

P133 Gene expression profiling by DNA microarrays in colon cancer treated with chelidonine alkaloid

Mahmoud Z El-Readi, Safaa Y Eid, Michael Wink

P134 Successful *in vitro* fertilization after eight failed trials

Ahmed M. Isa, Lulu Alnuaim, Johara Almutawa, Basim Abu-Rafae, Saleh Alasiri, Saleh Binsaleh

P135 Genetic sensitivity analysis using SCGE, cell cycle and mitochondrial membrane potential in OPs stressed leukocytes in *Rattus norvegicus* through flow cytometric input

Nazia Nazam, Mohamad I Lone, Waseem Ahmad, Shakeel A Ansari, Mohamed H Alqahtani

## O1 Regulation of genes by telomere length over long distances

### Jerry W. Shay

#### University of Texas Southwestern Medical Center, Dallas, Texas, USA

The ends of linear chromosomes are called telomeres that resemble broken DNA. However, the telomeres are protected from the DNA damage responses due to the formation of a looping structure (T-loop) and by “capping” the telomeres with a series of shelterin proteins. It is generally thought the main, if not only, function of telomeres is protection from DNA damage responses. When telomeres get very short due to normal replicative aging, cells stop dividing due to loss of the telomere end protective functions. We have previously reported that telomeres may also have additional functions in human cells. Telomeres are characterized by a distinct chromatin structure with spreading of heterochromatin into the subtelomeric regions, but little is known about the chromatin conformation of human telomeres. We have previously shown, using a luciferase reporter introduced into telomeres, that there is a 10-fold decreased expression of the reporter compared to the reporter introduced into internal genomic loci. This phenomenon is known as Telomere Position Effects (TPE). We have also found that a human gene, interferon stimulating gene 15 (ISG15), (~1 M base pairs from the 1p36.33 telomere) is silenced in young cells with long telomerase, upregulated in cells with short telomeres and silenced again in old cells with experimentally hTERT (telomerase) elongated telomeres. However, we observed genes between ISG15 and the telomere are not regulated by classic telomere position effects (TPE). To distinguish this type of telomere length dependent regulation of gene expression from classic TPE, we have termed this type of regulation Telomere Position Effects Over Long Distances (TPE-OLD). We discovered using 3D co-FISH and a modification of Hi-C (chromosome capture followed by high-throughput sequencing), that there are a significant number of genes within the first 10 M bases distal to the telomere on many chromosomes regulated by telomere length with genes closer to the telomere not being regulated by TPE.

## O2 The microtubule destabilizer KIF2A regulates the postnatal establishment of neuronal circuits in addition to prenatal cell survival, cell migration, and axon elongation, and its loss leading to malformation of cortical development and severe epilepsy

### Noriko Homma^1^, Ruyun Zhou^1^, Muhammad Imran Naseer^2^, Adeel G. Chaudhary^2^, Mohammed Al-Qahtani^2^, Nobutaka Hirokawa^1,2^

#### ^1^Department of Cell Biology and Anatomy, Graduate School of Medicine, the University of Tokyo, Tokyo 113-0033, Japan; ^2^Center of Excellence in Genomic Medicine Research, King Abdulaziz University, Jeddah, 21589, Saudi Arabia

##### **Correspondence:** Nobutaka Hirokawa (hirokawa@m.u-tokyo.ac.jp) – Center of Excellence in Genomic Medicine Research, King Abdulaziz University, Jeddah, 21589, Saudi Arabia

Kinesin super family protein 2A (KIF2A) is an ATP-dependent microtubule destabilizer, that belongs to the kinesin-13 family. It is highly expressed in juvenile brains, but its postnatal function has not been determined due to mortality in KIF2A-deficient mice. In addition, KIF2A is a causal gene in human Malformation of Cortical Development (MCD) with epilepsy, but the contribution of KIF2A to its pathogenesis is not yet understood due to the small number of human patients. In this study, we first analyzed the prenatal function of KIF2A using kif2a-KO mice. These mice were pale, breathed irregularly, and exhibited frequent twitching in addition to malformations resembling those in kif2a-mutated human patients. To determine the post-migration function of KIF2A, we generated newly tamoxifen-inducible conditional knockout mice. Despite successful neuronal migration, all offspring displayed hyper-activity, weight loss, severe cortical/hippocampal-focus epilepsy, and died. Interestingly, KIF2A was highly expressed in excitatory axons, in cortical neurites in layers II/III/V and in dentate mossy fibers (MF). In the cortex, the loss of KIF2A generated aberrant axon sprouting in those layers. In the hippocampus, the loss of KIF2A resulted in the development of hippocampal sclerosis, MF sprouting, and recurrent excitatory circuits resembling but different from TLE. These phenotypes developed without excitation. cKO granule cells exhibited failed axon/dendrite determination, and developed multiple short axons throughout the entire molecular layer. These results suggest that KIF2A is crucial postnatally for establishing accurate neuronal circuits, in addition to its role in prenatal cell survival, migration, and axon elongation.

## O3 Integration of metagenomics and metabolomics in gut microbiome research

### Maryam Goudarzi^1^ and Albert J. Fornace Jr.^1,2,3,4^

#### ^1^Department of Biochemistry and Molecular & Cellular Biology, Lombardi Comprehensive Cancer Center. Georgetown University, Washington, DC, USA; ^2^Department of Oncology, Lombardi Comprehensive Cancer Center, Georgetown University, Washington, DC, USA; ^3^Department of Radiation Medicine, Lombardi Comprehensive Cancer Center, Georgetown University, Washington, DC, USA; ^4^Center of Excellence in Genomic Medicine Research (CEGMR), King Abdulaziz University, Jeddah, KSA

##### **Correspondence:** Albert J. Fornace Jr. (af294@georgetown.edu) – Center of Excellence in Genomic Medicine Research (CEGMR), King Abdulaziz University, Jeddah, KSA

The gut is the largest reservoir of microbes in the body, harboring more than 100 trillion microorganisms in a well-balanced communication with the host factors. The gut microbiota plays an essential role in a wide range of biological functions such as digestion and the development of immunological responses. The microbial community composition and diversity is important in maintaining the homeostatis in the host. Factors, which may influence the gut microbial composition include diet, environmental exposures, age, genetics, and many more. Recent advances in technology, particularly 16S rRNA sequencing and mass spectrometry have enabled us to survey these microbial communities at the phylogenetic level and assess the host/microbes co-metabolism. The metagenomic studies have shed light on the functional composition of the gut microbiota and have determined that in diseases such as inflammatory bowel disease (IBD) there is an increase in functions of the auxotrophic and pathobiont bacteria. A number of studies have also pointed to an increase in the sulfate-reducing bacteria, such as *Desulfovibrio,* in IBD. On the other hand, metabolomics studies have revealed that taurine-conjugated bile acids increase the availability of free sulfur causing an expansion of the sulfate-reducing pathobiont *Bilophila wadsworthia*, which drive colitis in genetically susceptible *IL10*^*−/−*^ but not wild-type mice. Furthermore, an increased in glutathione transport and riboflavin metabolism and a decrease in biosynthesis of amino acids have been reported in ulcerative colitis patients, which is indicative of increased predisposition for managing oxidative stress, a hallmark of an inflammatory environment. Our current studies have explored the correlative nature of the microbe/host co-metabolism to characterize the regulatory role of microbiota in maintaining host health. These correlative host/microbial studies have the potential to determine causality, response to treatment, risk prediction, and keystone species for use as probiotics in diseases such as IBD or colitis induced by immuno-radiotherapy.

## O4 A unique integrated system to discern pathogenesis of central nervous system tumors

### Saleh Baeesa, Deema Hussain, Mohammed Bangash, Fahad Alghamdi, Hans-Juergen Schulten, Angel Carracedo, Ishaq Khan, Hanadi Qashqari, Nawal Madkhali, Mohamad Saka, Kulvinder S. Saini, Awatif Jamal, Jaudah Al-Maghrabi, Adel Abuzenadah, Adeel Chaudhary, Mohammed Al Qahtani and Ghazi Damanhouri

#### King Fahd Medical Research Centre, King Abdulaziz University, Jeddah, Saudi Arabia

##### **Correspondence:** Deema Hussain (deemah@hotmail.com) – King Fahd Medical Research Centre, King Abdulaziz University, Jeddah, Saudi Arabia

Central Nervous System Tumors (CNST) are a collection of neoplasms that grow in the brain and the spinal cord. Long term survival for patients carrying these tumors can reach at best 14 % in countries that provide optimal health operating systems. Despite advancement in cancer targeted therapy, surgery and radiation remain typically the main methods of treatment, even though both are challenging and may affect the quality of life of survivors adversely. Inventive approaches and deeper comprehensions of tumor characteristics are needed to transform treatment options for CNST patients. We established a unique integrated system to enable analysis of multiple bio-parameters of individual CNST. Each sample recovered was prepared to preserve tissue and generate a corresponding cell line with a purpose to process for genetic, biochemical, cytological and pharmacological analysis. A collection of 84 tumors were recovered, with a wide range of tumor types including Meningiomas, Astrocytomas, Oligodendrogliomas, Medulloblastomas and Glioblastomas. Generating a tumor type combined outlook facilitated access to vital information, as exemplified by the analysis of sixteen primary Meningiomas. Investigating the gene expression profile of uncultured tumors samples while considering the live cell behavior in relation to morphology, growth, stemness and sensitivity to chemotherapy, enabled the identification of possible novel prognostic markers for Meningiomas, including the recently associated cancer stem cell marker *Anterior Gradient 2 Homolog (AGR2)*. This integrated analysis approach is critical for the development of safe, efficacious and potent targeted therapeutic modalities.

## O5 RPL27A is a target of miR-595 and deficiency contributes to ribosomal dysgenesis

### Heba Alkhatabi

#### Centre of Excellence in Genomic Medicine Research, King Abdulaziz University, Jeddah, Saudi Arabia

Myelodysplasia with monosomy 7 or deletion 7q has a dismal prognosis and high propensity for leukaemic transformation. The exact pathogenesis of these disorders remains elusive. We investigated functional consequences of deletion of microRNAs (miRNAs) residing on chromosome 7q, focusing on miR-595. Using a novel assay, several targets for miR-595 were identified, including a large ribosomal subunit RPL27A. RPL27A downregulation induced p53 activation, apoptosis and inhibited proliferation. Importantly, p53-independent effects were identified secondary to a reduction in the ribosome subunit 60s with associated ribosome dysgenesis. Of note, RPL27A overexpression showed no significant effects on p53 mRNA levels but did enhance proliferation. In normal CD34+ cells, RPL27A knockdown preferentially blocked erythroid proliferation and differentiation. Lastly, miR-595 appears significantly downregulated in MDS patient samples possessing -7/-7q anomalies compared to those with normal karyotype. We postulate that haploinsufficency of miR-595 in patients with -7/del 7q may contribute to disease pathogenesis via RPL27A modulation.

## O6 Next generation DNA sequencing panels for haemostatic and platelet disorders and for Fanconi anaemia in routine diagnostic service

### Anne Goodeve, Laura Crookes, Nikolas Niksic, Nicholas Beauchamp

#### Sheffield Diagnostic Genetic Service, Sheffield Children’s NHS Foundation Trust, Western Bank, Sheffield, UK

##### **Correspondence:** Anne Goodeve (a.goodeve@sheffield.ac.uk) – Sheffield Diagnostic Genetic Service, Sheffield Children’s NHS Foundation Trust, Western Bank, Sheffield, UK

Next Generation sequencing (NGS) is transforming delivery of diagnostic molecular genetics. Gene panels are being utilised to analyse groups of related disorders and to extend diagnostic capability in comparison with previous Sanger sequencing. The aim of this study is to examine the utility of gene panels for diagnostic/research NGS analysis of haemostatic and platelet disorders and for Fanconi anaemia. Requests were received for all but one (*F5*) genes on the haemostasis/platelet panel. Candidate pathogenic mutations were identified in 24 of 39 patients (62 %), including 15/16 males diagnosed with haemophilia A and 3/7 individuals with possible von Willebrand disease. Some analyses (e.g. *ADAMTS13*, *MYH9, VWF*) were requested to help exclude specific diagnoses. In addition to analysis of single genes, combinations were analysed simultaneously, e.g. *F8 & F9* (possible carrier relative of deceased haemophilia patient; unknown type)*; F8, F9, F13A1, F13B* and *VWF* (baby died of bleeding shortly after birth), *F8 & VWF* (low FVIII:C). In contrast, nearly all patients referred for FA analysis were seeking a diagnosis of the disorder and all 16 genes were analysed in most individuals investigated (15/19). Biallelic mutations were identified in 7 cases (33 %) in *BRCA2, FANCA, FANCC, FANCD2* and *FANCG.* A single heterozygous mutation was identified in 2 patients (13 %), 2 heterozygous mutations in different genes in 1 case (7 %), homozygous mutations in 2 (13 %) and no mutation in 5 (33 %). We can conclude that NGS provides a single laboratory workflow for analysis of gene panels for related disorders as well as for whole genomes/exomes. Data analysis can include a single gene, such as *ADAMTS13*, or ≥ 1 gene for disorders such as those affecting fibrinogen. For disorders potentially caused by one of the several genes in a pathway such as FA, all can be analysed simultaneously. Use of NGS provides a single laboratory workflow for analysis of gene panels for many different disorders and can dramatically reduce time to achieve a definitive diagnosis.

## O7 Targeted sequencing panels and their utilization in personalized medicine

### Adel M. Abuzenadah

#### Center of Innovations in Personalized Medicine, King Abdulaziz University, Jeddah, Saudi Arabia

Several milestones have to be achieved in order to introduce the concept of personalized medicine to the healthcare system in Saudi Arabia. There is a notable lack of publicly available information about the “normal” genetic makeup of this population which is hindering the complete elucidation of “disease” genomes. Therefore, it is important that considerable effort should be spent on the identification of the genetic and epigenetic risk factors that predispose to the common diseases in the Kingdom of Saudi Arabia. Knowledge gained will help in the elucidation of disease biomarkers and drug response modifiers. Furthermore, providers of personalized medicine should validate internationally-approved biomarkers and risk factor indicators for use in the Kingdom of Saudi Arabia. Most importantly, genetic tests offered to the public should be designed to be cost-effective without compromising sensitivity or specificity and with fast turnaround amenable to use in the clinic. Towards this end, we at the Center of Innovations in Personalized Medicine (CIPM) at King Abdulaziz University are utilizing the robustness of the Ion Torrent Personal Genome Machine to design genetic tests for afflictions common to the Kingdom of Saudi Arabia. We have designed and tested custom Ampliseq panels as well as used panels from the Life Technologies catalog. Our array of targeted sequencing panel now covers conditions such as deafness, beta-thalassemia, thrombophilia, inborn errors of metabolism, and cancer susceptibility. We will report on the progress achieved so far and discuss the future of such platform in the delivery of individualized diagnostics.

## O8 International biobanking in the era of precision medicine

### Jim Vaught

#### President, ISBER, Editor-in-Chief, *Biopreservation & Biobanking*, USA

Biospecimens are collected from patients and other donors in a variety of ways for basic, clinical and epidemiologic research studies. Methods for collecting, processing, storing and analyzing biospecimens have usually been developed locally for diagnostic and research purposes, with little consideration for standardization or long-term quality management. Biospecimen management has developed into a more scientifically-based endeavor, as researchers have realized that biospecimens and data of consistent quality are required as we enter the era of precision medicine.

Best practices have been developed by organizations such as the International Society for Biological and Environmental and Repositories (ISBER), the U.S. National Cancer Institute (NCI), the International Agency for Research on Cancer (IARC) and the European Organization for Economic Cooperation and Development (OECD). Biospecimen research programs have been developed to establish evidence-based standard procedures to control the collection and processing of biospecimens. Molecular data from the analysis of biospecimens contribute directly to the diagnosis of disease and treatment of patients. Various “pre-analytical” variables can affect the quality of biospecimens. Among these variables are: the amount of time that it takes for surgical removal of the biospecimen; the time the specimen spends at room temperature before it is frozen or fixed with formalin; and many other variables that can affect biospecimen quality. There is the potential for: incorrect diagnoses; incorrect treatment; irreproducible results; and overall the potential for misinterpretation of artifacts as new biomarkers. The adoption of best practices for biospecimen collection and processing is an important part of the strategy to advance translational research and precision medicine. These practices should include:

- Governance models with clearly stated technical standards, ethical guidelines, access policies and procedures, scientific rationale, and long-term custodianship plans.
- A strong quality management program with clearly defined standard operating procedures, and regular audits to assure compliance.
- A comprehensive business model that has a sustainable cost-recovery plan, or a plan to assure consistent long-term financial support.
- In general, adherence to a set of best practices governing both technical and ethical/legal issues.

The future development of international collaboration in biobanking will require cooperation among various nations to standardize and harmonize their biospecimen practices.

## O9 Biobank and biodata for clinical and forensic applications

### Bruce Budowle^1,2^, Mourad Assidi^2^, Abdelbaset Buhmeida^2^

#### ^1^Institute of Applied Genetics, Department of Molecular and Medical Genetics, University of North Texas Health Science Center, Fort Worth, TX 76107, USA; ^2^Center of Excellence in Genomic Medicine Research, King Abdulaziz University, Jeddah, Saudi Arabia

##### **Correspondence:** Bruce Budowle (bruce.budowle@unthsc.edu) – Center of Excellence in Genomic Medicine Research, King Abdulaziz University, Jeddah, Saudi Arabia

With advances in molecular biology (i.e., OMICs) and computational capabilities, society is on the verge of demystifying the causes of diseases with an expectation of better diagnostics, prognostics, and possible therapeutics for health and well-being. However, forensic science (for 30 years) embraced the field of genetics and built an entire infrastructure relying on DNA typing from biospecimen collection to final results integrated under strict and formal quality assurance guidelines. Both the medical and forensic disciplines rely on the accumulation of big data to perform precision, or personalized, analyses. These two disciplines have built biobanks and/or databanks with different rationales, designs and governance. Databanks and databases can be exploited to harness the power of emerging technologies and either enable detection of putative genes or potential suspects, and promote development of innovative biomarkers and treatments. Both resources must provide value, have proper ethical/conduct governance, develop capabilities to store and retrieve samples and annotated data, and be managed and maintained. Medical databases typically are governed by institutions (often not government agencies, although constrained by laws) and can be either free to those who donate samples or consumers pay for tests. Samples and metadata are provided on a voluntary consent basis, and the resource may be available to many interested parties. Forensic databases are controlled by the government, access is limited to law enforcement, and sample donation often is mandatory. Medical and forensic big data resources, views of data sharing, experiences of both systems users are discussed. Another resource, the human microbiome, should be considered. The composition of the microbiome can affect health and well-being. Indeed, the microbiome may end up being a more flexible and dynamic manner to address aspects of personalized medicine. Forensic genetics also may benefit as the microbes may serve as genetic signatures that could individualize humans and serve as another powerful identification tool. Forensic and medical biobanks should begin considering on how to sample, collect and store such samples in suitable core facilities, the microbiome banks. Such integration of the microbiome to either forensic and/or medical biobanks will revolutionize current approaches towards individualized medicine and precision forensics.

## O10 Tissue microarray technique: a powerful adjunct tool for molecular profiling of solid tumors

### Jaudah Al-Maghrabi

#### Department of Pathology, Faculty of Medicine, King Abdulaziz University, Jeddah, Saudi Arabia

Tissue microarray (TMA) is one of the most revolutionary technologies introduced into research and routine laboratories during the past decade. It is based on the idea of applying miniaturization and a high throughput approach for tissue analysis. TMA is a method of harvesting small disks of tissue from a range of standard histological sections and placing them in an array on a recipient paraffin block such that hundreds of cases can be analysed simultaneously saving money and time. In this presentation we will go through the guidelines for conducting TMA, Advantages and disadvantages of TMA and we will present our experience of TMA establishment in Saudi Arabia. In the Kingdom of Saudi Arabia remains the Tissue Microarray “TMA” technique almost absent in the laboratories of pathology, at least, to our knowledge, in the western region of the Kingdom, and therefore, this project will contribute in principle to establish this modern technology, to help reduce expenses for materials and reagents used in research conducted in the field of cancer. So far, we successfully transferred approximately 7723 FFPE blocks of different types of solid tumors which, already archived at department of pathology, KAUH, during the last two decades to approximately 180 TMA FFPE Blocks. In addition, we validated and estimated the concordance rate between conventional FFPE full section and TMA FFPE slides which, was realistic and of good quality. Moreover, we also validated the TMA technique in an integrative and comprehensive approach with immunohistochemistry (IHC) for protein profiling of different markers and Bright-field double in situ hybridization (BDISH) for gene profiling in different solid tumors. The TMA technique seems to us as feasible, reasonable, doable and multipurpose. However, hard work is considered necessary to procure the associated clinic-pathologic and follow up data of these samples to make the full package constructive and valuable for the scientists and research community for further downstream molecular profiling and characterization of solid tumors in order to initiate a basic platforms (infrastructure) for prognostic and predictive genomic models (molecular signatures) to facilitate the approach towards personalized oncology in Saudi Health System.

## O11 The CEGMR biobanking unit: achievements, challenges and future plans

### Abdelbaset Buhmeida^1^, Mourad Assidi^1^ and Leena Merdad^2^

#### ^1^Center of Excellence in Genomic Medicine Research, King Abdulaziz University, Jeddah, Saudi Arabia; ^2^Department of Dental Public Health, Faculty of Dentistry, King Abdulaziz University, Jeddah, Saudi Arabia

##### **Correspondence:** Abdelbaset Buhmeida (abuhme@utu.fi) – Center of Excellence in Genomic Medicine Research, King Abdulaziz University, Jeddah, Saudi Arabia

Since the achievement of the Human Genome Project in 2003, a significant increase in biobanks in terms of number, design, scale and governance has been noticed worldwide in order to support effective genomic research. Biobanks/Biorepositories are the main core around which OMICs scale research and advanced clinical applications have been performed to drive the Precision Medicine wave toward shaping the future of human welfare. Consequently, a biobanking unit has been developed at the Center of Excellence in Genomic Medicine (CEGMR) in 2008 to collect, store and release high quality biospecimens with fully annotated clinico-pathological data according to best practices established worldwide in order to deliver personalized diagnostics and tailor individualized therapeutics for the Saudi population. Despite promising milestones and over 150 publications in ISI journals using CEGMR Biobank Unit (CBU) biospecimens, the awareness about biobanking and biospecimen donation remains the foremost challenge. Reaching the benefits of biobanks rely on an educated public and well trained healthcare providers that will enable the establishment of perpetual and comprehensive partnerships between several stakeholders involved in healthcare service. A survey conducted by our CBU team targeted healthcare students at King Abdulaziz University and showed that only 46 % of them have heard about the “Human Genome Project”. Surprisingly, 72 % of these future healthcare providers have never heard of the term “biobank” which was significantly correlated with lower willingness to donate biospecimens. Interestingly, around 50 % of healthcare students were willing to donate biospecimens and believed that it will advance medical research and benefit the whole society. Better general health status, previous blood donation and higher scores of biobanking knowledge were significantly associated with the willingness to donate (*p*-value = 0.048, *p*-value = 0.043 and *p*-value < 0.001, respectively). These findings highlight the urgency of developing a multidisciplinary awareness strategy to integrate biobanking and OMICs scale approaches in the healthcare students’ curricula, building up therefore well qualified healthcare providers’ competencies. Simultaneously, a suitable and lifelong public outreach program about biobanking tailored to the diverse communities in Saudi Arabia must be launched to ensure their effective involvement in the biobanking and precision medicine trend with adequate awareness about benefits and risks.

## O12 Phylomedicine of tumors

### Sudhir Kumar, Sayaka Miura, and Karen Gomez

#### Institute for Genomics and Evolutionary Medicine, Temple University, Philadelphia, PA 19122, USA

##### **Correspondence:** Sudhir Kumar (s.kumar@temple.edu) – Institute for Genomics and Evolutionary Medicine, Temple University, Philadelphia, PA 19122, USA

Comparative analysis of sequences is routinely employed in tracing the origins, patterns, and evolutionary relationships of homologous sequences from strains, populations, and species. Now, these analyses are poised to become key in oncology owing to escalating sequencing of tumors. They will reveal evolutionary history of clones that comprise tumors, patterns of sequence diversity, and tempo and mode of clonal evolution that underlie the origin and adaptive proliferation of cancerous cells. I will present results from our evaluation of the performance of many existing computational methods in correctly inferring tumor clones from multi-region sequencing data. I will also present our new methods for inferring clone and tumor histories, which establish that our new method accurately infers (a) clusters of variants (clonotypes) that comprise a tumor, (b) evolutionary tree of clonotypes, and (c) estimates of their relative frequencies in tumors. These methods are based on fundamental molecular evolutionary principles and they will greatly facilitate cancer-related research pursued by basic biologists and clinicians.

## O13 Clinical implementation of pharmacogenomics for colorectal cancer treatment

### Angel Carracedo^1^, Mahmood Rasool^2^

#### ^1^Genomic Medicine Group- Galician Foundation of Genomic Medicine (SERGAS), CIBERER, University of Santiago de Compostela, Santiago de Compostela, Spain; ^2^Center of Excellence in Genomic Medicine, King Abdulaziz University, Jeddah, KSA

##### **Correspondence:** Angel Carracedo (angel.carracedo@usc.es) – Genomic Medicine Group- Galician Foundation of Genomic Medicine (SERGAS), CIBERER, University of Santiago de Compostela, Santiago de Compostela, Spain

Colorectal cancer (CRC) is the third most prevalent cancer in men and the second in women worldwide and although a great improvement in response rate and patient’s survival was recently achieved through the introduction of new-targeted agents in combination with standard fluoropyrimidines-based chemotherapeutic regimens, still adverse effects and development of chemoresistance are important limitations to pharmacological therapy. Some biomarkers to guide CRC treatment have been developed and some of them are considered valid by the regulatory agencies and included in the technical sheet of the drugs but many others are still “probable valid” needed of additional validation and cost-efficiency studies. Some of the most promising biomarkers are still not translated to clinical practice nor approved by the agencies. In addition, there is a lack of integration in a common framework of biomarkers at different levels (germline, somatic, epigenetic) limiting translation and cost-efficiency analysis. Here we present a strategy for the translation to clinical practice of pharmacogenomic biomarkers to predict response (both efficacy and ADRs) and to guide treatment in colorectal cancer including previous valid biomarkers approved by EMA, probable biomarkers at germline and somatic level. The selected markers include variants at DNA level (sequence level and methylation markers) and RNA level (including miRNA). The use of specific CTC genomic alterations associated with treatment resistance and recurrence to guide the selection of target therapies in mCRC patients will be also discussed. The final goal is an integrative approach for a practical translation to clinical practice of CRC pharmacogenomics. To achieve this goal cost efficiency studies will be performed and a pilot project has been to organize workflow and optimize decision-making processes.

## O14 From association to causality: translation of GWAS findings for genomic medicine

### Ahmed Rebai

#### Laboratory of Molecular and Cellular Screening Processes (Bioinformatics Group), Centre of Biotechnology of Sfax, P.O. Box “1177”, Sfax, Tunisia

With the decrease of the cost of high throughput genotyping and sequencing, Genome wide Association Studies (GWAS) have become a good approach to identify sequence variations and mainly Single nucleotide polymorphisms (SNP) that are related to complex and common diseases. However, GWAS data allow only associational inference and is only able to identify sequence variants that are statistically associated with the disease status. The last seven years have seen an exponential growth in the number of GWAS which resulted in over 15 thousand associated Single Nucleotide polymorphisms (SNPs) identified in more than 2100 published studies. Although many statistical methods and algorithms have been developed to increase the power to detect association, testing causal relationship between the associated SNPs and the disease risk was not given much attention. A causal variant is a ‘mutation’ that contributes to an increase in risk to disease. Establishing causality from association is thus a challenging task, hindered by many complicating factors and remains a major concern in identifying genetic causes of common diseases, particularly for clinical translation of GWAS findings. In order to establish causality, we generally need intervention, which means not only observing the genotypes of some individuals but taking actions that can manipulate these genotypes, which can only be done in animal models. However, recent studies showed that, although we cannot estimate causal effects using observations alone, it is possible to use the classical framework of causality inference to get estimate of lower bounds for these effects. Here I build on recent works within the Bayesian networks modeling framework, to present an original approach to test causality based on observation data only. Based on standard GWAS data alone, this approach provides a measure for ranking causal effects of associated variants. The accuracy and stability of this measure is assessed and compared to other approaches. Selecting the most likely causal variants from GWAS results will allow the prioritization and designs of targeted experiments to unravel causal mechanisms underlying association and effective clinical translation of GWAS findings for genomic medicine, including individual risk prediction, advanced clinical strategies and personalized treatments.

## O15 E-GRASP: an interactive database and web application for efficient analysis of disease-associated genetic information

### Sajjad Karim^1^, Hend F Nour Eldin^1^, Heba Abusamra^1^, Elham M Alhathli^1^, Nada Salem^1^, Mohammed H Al-Qahtani^1^, and Sudhir Kumar^1,2^

#### ^1^Center of Excellence in Genomic Medicine Research, King Abdulaziz University, PO Box 80216, Jeddah 21589, Saudi Arabia; ^2^Institute for Genomics and Evolutionary Medicine, Temple University (SERC 602A), Philadelphia, PA 19122, USA

##### **Correspondence:** Sajjad Karim (skarim1@kau.edu.sa) – Center of Excellence in Genomic Medicine Research, King Abdulaziz University, PO Box 80216, Jeddah 21589, Saudi Arabia

Genome-wide association study data is used widely to identify the genetic variants associated with complex traits or common disease. Genome-Wide Repository of Associations between SNPs and Phenotypes (GRASP) is a refined database, containing ∼ 8.87 million SNP associations reported in 2044 studies and ~178 thousand phenotypes, derived from GWAS data. GRASP v2.0 users face difficulties in access and correlation of SNPs with traits. Thus we aimed to design an interactive database providing detailed information of SNPs required for better data interpretation, communication between possible collaborators and new hypotheses to be generated. To develop a web application, we used ASP.net as front-end tool; SQL server, MatLab, D3.js, D3Plus.js and jQuery data table as back-end tools; JavaScript and Ajax for client-side purpose. MATLAB was used to analyse the statistical replication and evolutionary information of each SNPs and all SNPs were mapped to genome position of human genome build 38 (hg38) using LiftOver. We developed a new web application called E-GRASP using an advance tool and different filter code to retrieve and represent data in better way leading to fast and easy data analysis. We retrieved and retained all information of GRASP in E-GRASP and added new information including statistical replication and evolutionary information of each SNPs. E-GRASP provides information under following categories: (i) SNPs view- provides information about SNPid, PMID, P-value, chromosome, position, number of studies and phenotypes for each SNPs; (ii) Study view- explains about unique SNPs replication in each studies and phenotypes, and (iii) Evolutionary view- describes evolutionary information including E-value for SNP phenotype association, E-rank of the evolutionary rate and time span of the position retrieved from the “E-rank Web Server” for each SNP. Web application allows the users to computed P-rank and E-rank using P-value and E-value respectively for each phenotype of different studies. In conclusion, E-GRASP is more representative database with additional information of SNPs replication and evolutionary scores, facilitating the comprehensive qualitative and quantitative analysis. In future, we aim add complete data sets of studies available in GRASP and provide more filters to get refined information.

## O16 The supercomputer facility “AZIZ” at KAU: utility and future prospects

### Hossam Faheem

#### Fujitsu Technology Solutions International

Aziz is ranked No. 360 among the world’s Top 500 supercomputers. Aziz is also one of the top 10 supercomputers in Kingdom of Saudi Arabia. Primary objective of Aziz is to support growing number of researchers and scientists in King Abdul Aziz University and its partners across different geographical locations. The compute facility consists of high-end compute nodes; interconnect infrastructure, and storage capabilities. Compute nodes consist of a total of 496 nodes (11,904 cores) for running large parallel jobs as well as a large number of small parallel or serial jobs. 380 nodes are standard compute nodes (9120 cores) with 96 GB (4GB per core) for running the majority of mixed HPC applications. The remaining 112 high memory compute nodes (2688 cores) with 256 GB (10.6GB per core) are intended for applications that require large memory for their execution. 2 NVidia Tesla K20 GPGPU equipped compute nodes (2496 CUDA cores) with 96GB for running applications with the ability to use GPU-based accelerators. 2 Intel Phi 5110P Co-processor equipped compute nodes (120 Xeon phi cores) with 96GB for running applications with the ability to use MIC based accelerators. Storage space contains Fujitsu FEFS high-speed parallel file system of 2 PB acts as scratch space as well as storage for current running jobs. A total of 28 I/O servers and Infiniband as interconnect enable high speed non-blocking storage access to compute nodes. To address the needs of longer term storage capacity at KAU, a total of 6 PB of usable permanent storage solution was provided with automatic archival system. A NAS storage unit of total 22 TB usable space acts as home directory and application space for users. Infiniband network consists of Intel QDR which is used as interconnect connecting computing nodes and FEFS high-speed storage system. It Features like non-blocking configuration and RDMA enables application use full potential of underlying interconnect and reach maximum performance, delivering results in least possible time. We believe that Aziz will have a great effect on accelerating scientific research and solving computationally intensive problems at KAU.

## O17 New research into the causes of male infertility

### Ashok Agarwal

#### Case Western Reserve University, School of Medicine and Lerner College of Medicine, Cleveland Clinic Foundation; American Center for Reproductive Medicine, Andrology Center, Cleveland, Ohio, USA

Twenty-five percent of male-factor cases present with no identifiable cause of male infertility. Conventional semen analysis provides important but limited information in the laboratory diagnosis of male infertility. While advanced sperm function tests provide additional information at the cellular level, understanding the underlying molecular mechanism of sperm dysfunction is important. The talk will mainly focus on new research into the causes of male infertility. Proteomics is the new frontier of research in male infertility as it allows the identification of numerous sperm-specific proteins. In addition, it provides a greater understanding of protein functions involved in sperm processes such as motility, capacitation, acrosome reaction & fertilization. Identification of alterations in major proteins associated with dysfunctional sperm will help in our understanding of the pathology of male infertility. The speaker will examine, 1) the current methodology and techniques used in the global proteomic analysis of sperm and seminal plasma samples from infertile men with various clinical diagnosis, 2) examine the bioinformatics tools and search engines employed in analyzing the data; 3) discuss the key findings of recent proteomics research published from his center. Finally, the speaker will review the future of proteomics in male infertility and list the major challenges in introducing proteomics in the laboratory diagnosis of male infertility.

## O18 The Klinefelter syndrome: recent progress in pathophysiology and management

### Eberhard Nieschlag^1,2^, Joachim Wistuba^1^, Oliver S. Damm^1^, Mohd A. Beg^2^, Taha A. Abdel-Meguid^3^, Hisham A. Mosli^3^, Osama S. Bajouh^4^, Adel M. Abuzenadah^2,5^, Mohammed H. Al-Qahtani^2^

#### ^1^Center for Reproductive Medicine and Andrology, University of Münster, Münster, Germany; ^2^Center of Excellence in Genomic Medicine Research, King Abdulaziz University, Jeddah, Saudi Arabia; ^3^Department of Urology, King Abdulaziz University Hospital, Jeddah, Saudi Arabia; ^4^Department of Obstetrics and Gynecology, King Abdulaziz University Hospital, Jeddah, Kingdom of Saudi Arabia; ^5^KACST Technology Innovation Center in Personalized Medicine, King Abdulaziz University, Jeddah, Saudi Arabia

##### **Correspondence:** Eberhard Nieschlag (Eberhard.Nieschlag@ukmuenster.de) – Center of Excellence in Genomic Medicine Research, King Abdulaziz University, Jeddah, Saudi Arabia

**Background**

The Klinefelter syndrome (KS), 47, XXY karyotype, is the most common chromosome aneuploidy in men characterized by hypogonadism, infertility, and other comorbidities. The incidence of KS in the general male population is 1:500, whereas in our ongoing project in Saudi Arabia the incidence is 1:11 in selected patients with azoospermia or low sperm counts.

**Fertility**

Until recently KS men were considered infertile as about 90 % of KS men are azoospermic. Recently, sperm were obtained by testicular sperm extraction for intracytoplasmic sperm injection into oocytes, resulting in live-born children. Most of these children have a euploid karyotype as these sperm derive from tubuli with euploid spermatogonial foci. As shown in a KS mouse model these spermatogonia get lost during early development so that cryopreservation of testicular biopsies from prepubertal boys for later gamete maturation *in vitro* is discussed.

**Comorbidities**

KS is often associated with gynecomastia, metabolic syndrome, diabetes type II, cardiopathies, thrombosis, embolism, osteoporosis, and epilepsy. Androgen receptor polymorphism influences the incidence and together with smaller diameter of arteries in KS may aggravate associated disorders. Paternal compared to maternal origin of X chromosome is associated with a more pronounced risk of insulin resistance, metabolic syndrome, and cardiac disorders.

**Psycho-neurological function**

Half of KS patients suffer from disturbed verbalization and legasthenia causing difficulties in communication and learning, many develop autism spectrum disorders. Neuro-imaging showed anomalies in the brain of KS patients. KS mice also exhibit cognition, memory, and learning difficulties and help to elucidate the psycho-neurological shortcomings.

**Testosterone treatment**

All KS men develop a lack of testosterone sooner or later. Although the capacity for biosynthesis of testosterone of the hyperplastic Leydig cells is normal, the hormone seems to be trapped in the testis due to impaired blood flow as shown in the KS mouse. Therefore, testosterone substitution remains the prime option in treating KS men. As current modalities of testosterone substitution cannot remedy all symptoms, recent discussion focuses on when the treatment should be initiated (prepubertal, pubertal, or adult?) and what the optimal serum levels should be.

**Acknowledgements**

This project was funded by the Deanship of Scientific Research (DSR), King Abdulaziz University, Jeddah, under HiCi grant no. (1434-141-453). The authors, therefore, acknowledge with thanks DSR technical and financial support.

## O19 A new look to reproductive medicine in the era of genomics

### Serdar Coskun

#### Assisted Reproductive Technology Laboratory, Department of Pathology and Laboratory Medicine, King Faisal Specialist Hospital and Research Center, Riyadh, KSA

Recent advances in the molecular biology made the genomic technologies widely available to the medical community. Reproductive medicine is also adapting these changes into its practice. The impact is more profound in the field of preimplantation genetic diagnosis (PGD). The main limitation in PGD has been the availability of a small quantity of genetic material hampering the use of genomic technologies. In recent years, improved whole genome amplification techniques with more sensitive detection methods made possible to use microarrays and next generation sequencing in PGD. We now can identify single gene disorders in embryos along with screening all 23 chromosomal abnormalities. Currently, there are studies evaluating the effect of chromosomal screening in improving the in vitro fertilization outcomes. Another impact of genomic technologies will be on understanding the mechanism of infertility. There is about 20 % of the infertility cases considered as “unexplained” with no known causes according to the current tests available. Whole genome sequencing might help to uncover the genetic bases of these cases. We have recently shown TLE6 mutation the underlying cause of repeated fertilization failure. The third impact of these technologies will be on treatment where drugs and dose could be individualized according to the genetic background of a person. Some of the genomic technologies are also being used in prenatal diagnosis (PND) in the clinical settings. Namely, the noninvasive PND is now available to the patients as a routine test. In this presentation, the newly adapted genomic methodologies and their applications to the reproductive medicine will be reviewed.

## P1 Wnt signalling receptors expression in Saudi breast cancer patients

### Muhammad Abu-Elmagd^1,2^, Abdelbaset Buhmeida^1,2^, Ashraf Dallol^1,2^, Jaudah Al-Maghrabi^3^, Sahar Hakamy^1^, Wejdan Al-Qahtani^1^, Asia Al-Harbi^1^, Shireen Hussain^1^, Mourad Assidi^1,2^, Mohammed Al-Qahtani^1,2^, Adel Abuzenadah^1,2^

#### ^1^Center of Excellence in Genomic Medicine Research, King Abdulaziz University, Jeddah, Saudi Arabia; ^2^Center of Innovation in Personalized Medicine, King Abdulaziz University, Jeddah, Saudi Arabia; ^3^Department of Pathology, Faculty of medicine, King Abdulaziz University, Jeddah, Saudi Arabia

##### **Correspondence:** Muhammad Abu-Elmagd (mabuelmagd@kau.edu.sa) – Center of Innovation in Personalized Medicine, King Abdulaziz University, Jeddah, Saudi Arabia

**Background**

Wnt ligands and their receptors “Frizzleds’ constitute a pivotal signalling that mediates a considerable number of cellular events during development, in adulthood and cancer including fate determination, polarity, tissue patterning, and proliferation. At least ten members of frizzled transcription factors have been characterised each of which is known as seven transmembrane G protein-coupled receptor. In cancer, frizzled receptors expression is associated with tumour development and patient’s outcomes including recurrence and survival [1]. High level of Fizzled6 expression in particular was reported during leukemogenesis [2]. Our current study aimed to analyse the expression pattern of a number of frizzled receptors in Saudi Arabia breast cancer (BC) tissue and here we present only FRIZZLED6 (FZD6) analysis.

**Subjects and methods**

BC tissue samples from more than 615 patients aged between 25-80 years who were reported for BC complications at King Abdulaziz University Hospital, Jeddah, Saudi Arabia were used. All samples, processed for paraffin sectioning, were subjected to tissue array technology and automated immunohistochemistry staining for Frizzled genes according to the protocol described in detail in [3].

**Results**

Expression pattern analysis of Frizzled6 revealed that its expression is mainly cytoplasmic while few cases showed, in addition, nuclear expression reflecting the heterogeneity of the tumour. The majority of BC tissues (60 % of the samples) showed no/weak expression patterns while around 40 % of the samples showed moderate to high level of the expression.

**Conclusions**

We have analysed the expression pattern of a number of Wnt signalling receptors (Frizzleds) in Saudi BC patients. Among these receptors is FZD6 which revealed in general weak expression. FZD6 expression is currently further analysed and being correlated with patients clinico-pathological features in order to evaluate its prognostic significance in BC.

**Acknowledgements**

This project was funded by the National Plan for Science, Technology and Innovation (MAARIFAH) – King Abdulaziz City for Science and Technology, Saudi Arabia – Award number (13-CIPM-01).

**References**

1. Ueno K, Hirata H, Hinoda Y, Dahiya R: **Frizzled homolog proteins, microRNAs and Wnt signaling in cancer**. *International journal of cancer Journal international du cancer* 2013, **132**(8):1731-1740.

2. Wu QL, Zierold C, Ranheim EA: **Dysregulation of Frizzled 6 is a critical component of B-cell leukemogenesis in a mouse model of chronic lymphocytic leukemia**. *Blood* 2009, **113**(13):3031-3039.

3. Al-Khattabi H, Kelany A, Buhmeida A, Al-Maghrabi J, Lari S, Chaudhary A, Gari M, Abuzenadah A, Al-Qahtani M: **Evaluation of HER-2/neu gene amplification by fluorescence in situ hybridization and immunohistochemistry in Saudi female breast cancer**. *Anticancer research* 2010, **30**(10):4081-4088.

## P2 Analysis of oxidative stress interactome during spermatogenesis: a systems biology approach to reproduction

### Burak Ozkosem^1^, Rick DuBois^2^

#### ^1^Biodesign Institute, Arizona State University, Tempe, AZ 85281, USA; ^2^Department of Chemistry and Biochemistry, Arizona State University, Tempe, AZ 85287, USA

##### **Correspondence:** Burak Ozkosem (burak.ozkosem@mail.mcgill.ca) – Biodesign Institute, Arizona State University, Tempe, AZ 85281, USA

**Background**

Daily production of spermatozoa is a complex process and severely affected by oxidative stress. Spermatogenesis is one of the most gainful cell-producing systems in animals, generating 100 million spermatozoa each day forming high levels of protein-protein interactions (PPIs). Interactions depend on cell type, cell cycle phase, developmental stage, oxidative stress conditions, redox mediated protein modifications, presence of cofactors, and other binding partners. To better understand how sperm production is regulated, we performed network analysis of redox mediated PPIs during spermatogenesis. Interactions between the major signaling pathways in germ cells yet to be investigated [1]. Furthermore, interactions between antioxidant defense proteins and sperm signaling pathways (e.g., energy production, sperm-egg recognition, and fertilization) are not known.

**Results**

Our interactome analysis combined experimental and predicted PPIs [2,3]. PPIs that were coming from different databases showed a small overlap, and also male infertility proteins are not well known and knowledge of their interactors are based on low throughput studies. Only 26 proteins were found to be annotated in the ReactomeDB [4]. Our curated network representing Antioxidant Response pathway proteins and their interactors consists of 101 nodes and 235 edges.

**Conclusions**

Almost 40 % of human genes do not have a PfamA or GO annotation, no current studies provide a list of potential functions [3,4]. We found that only two proteins, superoxide dismutase 1 and 2 (SOD1 and SOD2) had genetic interactions, while SOD3 had only one physical interaction which was inferred from mouse, also nitric oxidase 4 (NOX4) and glutathione peroxidase 5 (GPX5) didn’t show any interactions Other proteins showed physical interactions and more than 90 % those proteins had minimum 4 unique interactors. Oxidative stress response proteins with high number of interactions are involved in biological processes in sperm, and by manipulating this PPI network we simulated sperm specific conditions. Catalase (CAT) is not present in mature sperm, in the absence of CAT, the interactome would shift to new PPIs involving other antioxidants or alternative pathways. Combining computational and experimental tools for identifying PPIs will enrich the maps of PPIs in germ cells, and help understanding molecular basis of the increase in infertile population.

**References**

1. Ozkosem, B., & O'Flaherty, C: **Detrimental Effects of Oxidative Stress on Spermatozoa Lacking Peroxiredoxin 6**. *Free Radic Biol and Med* 2012, **53**: S86.

2. Orchard S, Kerrien S, Abbani S, Aranda B, Bhate J, Bidwell S, Bridge A, Briganti L, Brinkman Fiona SL, Cesareni G, Chatr-aryamontri A, Chautard E, Chen C, Dumousseau M, Goll J, Hancock Robert EW, Hannick LI, Jurisica I, Khadake J, Lynn DJ, Mahadevan U, Perfetto L, Raghunath A, Ricard-Blum S, Roechert B, Salwinski L, Stümpflen V, Tyers M, Uetz P, Xenarios I, Hermjakob H: **Protein interaction data curation: the International Molecular Exchange (IMEx) consortium**. *Nat Methods* 2012, **9**:345–350.

3. Barabási AL, Gulbahce N, Loscalzo J: **Network medicine: a network-based approach to human disease.***Nat Rev Genet* 2011, **12**: 56–68.

4. Vidal, M: **Interactome Modelling**. *FEBS Letters* 2005, 579, **8**: 1834-1838

**Fig. 1 (abstract P2):**
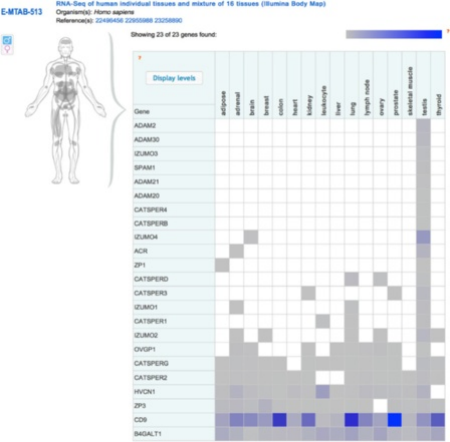
Expression details of known male fertility related proteins and functional Interactions

**Table 1 (abstract P2):** Major antioxidant response/defense proteins in testes and their interaction statistics

	High Throughput	Low Throughput	Total Interactions	Unique Interactors	Publications
CAT	13 (50 %)	13 (50 %)	26	13	15
GPX1	1 (10 %)	9 (90 %)	10	4	3
GPX2	3 (100 %)	0 (0 %)	3	3	1
GPX3	0 (0 %)	1 (100 %)	1	1	1
GPX4	5 (100 %)	0 (0 %)	5	4	4
GPX5	0 (0 %)	0 (0 %)	0	0	0
GPX6	2 (100 %)	0 (0 %)	2	2	2
GPX7	2 (100 %)	0 (0 %)	2	2	2
NOS2	139 (79 %)	36 (21 %)	175	148	17
NOX4	0 (0 %)	0 (0 %)	0	0	0
NOX5	0 (0 %)	5 (100 %)	5	4	2
PRDX1	92 (78 %)	26 (22 %)	118	98	52
PRDX2	45 (75 %)	15 (25 %)	60	53	23
PRDX3	33 (73 %)	12 (27 %)	45	33	24
PRDX4	42 (78 %)	12 (22 %)	54	42	27
PRDX5	35 (100 %)	0 (0 %)	35	32	11
PRDX6	41 (75 %)	14 (25 %)	55	45	29
SOD1	18 (24 %)	58 (76 %)	76	44	31
SOD2	18 (78 %)	5 (22 %)	23	19	13
SOD3	0 (0 %)	1 (100 %)	1	1	1
SRXN1	35 (100 %)	0 (0 %)	35	35	2
TXN	52 (60 %)	35 (40 %)	87	61	39

## P3 Interleukin-18 gene variants are strongly associated with idiopathic recurrent pregnancy loss

### Safia S Messaoudi^1^, Maryam T Dandana^2^, Touhami Mahjoub^2^, Wassim Y Almawi^3^

#### ^1^Forensic Biology Department, College of Forensic Sciences, Naif Arab University for Security Sciences, Riyadh, Kingdom of Saudi Arabia; ^2^College of Pharmacy of Monastir, Monastir, Tunisia; ^3^Department of Medical Biochemistry, Arabian Gulf University, Manama, Bahrain

##### **Correspondence:** Safia S Messaoudi (safsafsophie@gmail.com) – Forensic Biology Department, College of Forensic Sciences, Naif Arab University for Security Sciences, Riyadh, Kingdom of Saudi Arabia

**Background**

Interleukin-18 (IL-18) is an important regulator of innate and acquired immune responses with a proposed role in a variety of early inflammatory responses [1]. This cytokine is actively involved in the regulation of immune responses in order to have a successful pregnancy and enhances either T helper 1 (Th1) or Th2 differentiation depending on the immunologic state [2]. Alterations of IL-18 expression and secretion were linked with the pathogenesis of pregnancy complications, such as recurrent pregnancy loss (RPL) [3].

Furthermore, genetic variants in IL-18 gene were identified to have an impact on the level of IL-18 protein secretion [4].

In this study, we investigate the possible associations of Interleukin-18 (IL-18) promoter single nucleotide polymorphisms (SNPs) with idiopathic recurrent pregnancy loss (RPL).

**Materials and methods**

We evaluated −656C/A (rs1946519), −137G/C (rs187238), −119A/C (rs360718), and −105G/A (rs360717) IL-18 promoter polymorphisms (SNPs) by Taqman assays in 470 Tunisian women comprising 235 RPL cases and 235 age-matched multiparous control women. The association of IL-18 alleles, and genotypes with RPL was evaluated by Fisher’s exact test and regression analysis. A value of P < 0.05 will be considered statistically significant.

**Results**

Genotype distribution of −656C/A, −137G/C, −119A/C, and −105G/A was in Hardy–Weinberg equilibrium. The A allele of −105G/A (P < 0.001) and the A allele of −656C/A (P < 0.01), but not the C allele of −119A/C (P = 0.93) or C allele of −137G/C (P = 0.32), were significantly associated with RPL. Significant differences in −656C/A (P < 0.001) and −105G/A (P < 0.001), but not −119A/C (P = 0.78) or −137G/C (P = 0.12) regarding the distribution of genotypes were noted between RPL cases and control women. Since both variants were linked with reduced IL-18 availability [4] our findings underscore the significance of reduced IL-18 in the maintenance of normal pregnancy [5], and in the pathogenesis of pregnancy complications [6], including RPL [4]. Our results confirm the lack of association between rs187238and RPL in southern Iranian women [7].

**Conclusions**

We demonstrated that the IL-18 promoter variants −656C/A and −105G/A are significantly associated with RPL among Tunisian women.

**References**

1. Dinarello CA, Novick D, Kim S, Kaplanski G. **Interleukin-18 and IL-18 binding protein**. *Front Immunol.* 2013 Oct **8**;4:289.

2. Chaouat, G., Ledee-Bataille, N., Dubanchet, S., Zourbas, S., Sandra, O., Martal, J., 2004. **TH1/TH2 paradigm in pregnancy: paradigm lost? Cytokines in pregnancy/early abortion: reexamining the TH1/TH2 paradigm**. *Int. Arch. Allergy Immunol.***134**, 93–119.

3. Ostojić, S., Volk, M., Medica, I., Kapović, M., Meden-Vrtovec, H., Peterlin, B., 2007. **Polymorphisms in the interleukin-12/18 genes and recurrent spontaneous abortion.***Am. J. Reprod. Immunol.***58**, 403–408.

4. Barbaux, S., Poirier, O., Godefroy, T., Kleinert, H., Blankenberg, S., Cambien, F., Tiret, L., 2007. **Differential haplotypic expression of the interleukin-18 gene***. Eur. J. Hum. Genet*. **15**, 856–863.

5. Wilson, R., Moor, J., Jenkins, C., Miller, H., Walker, J.J., McLean, M.A., et al., 2004. **Abnormal first trimester serum interleukin 18 levels are asso-ciated with a poor outcome in women with a history of recurrent miscarriage**. *Am. J. Reprod. Immunol.***51**, 156–159.

6. Roland, L., Gagné, A., Bélanger, M.C., Boutet, M., Julien, P., Bilodeau, J.F., 2010. **Plasma interleukin-18 (IL-18) levels are correlated with antioxidant vitamin coenzyme Q(10) in preeclampsia. Acta Obstet**. *Gynecol. Scand*. **89**, 360–366.

7. Naeimi, S., Ghiam, A.F., Mojtahedi, Z., Dehaghani, A.S., Amani, D., Ghaderi, A., 2006. **Interleukin-18 gene promoter polymorphisms and recurrent spontaneous abortion.***Eur. J. Obstet. Gynecol. Reprod. Biol*. **128**, 5–9.

**Table 2 (abstract P3):** *IL-18* SNPs analyzed

Name	HWE *P*	Minor allele	Cases MAF^a^	Controls MAF	*P* ^b^	OR (95% CI)
rs1946519	0.58	A	0.46	0.31	<0.001	1.91 (1.48–2.45)
rs187238	0.65	A	0.31	0.28	0.32	1.15 (0.89–1.50)
rs360718	0.45	C	0.27	0.27	0.93	0.98 (0.75–1.28)
rs360717	0.12	A	0.49	0.30	<0.001	2.18 (1.70–2.78)

MAF, minor allele frequency; HWE, Hardy–Weinberg equilibrium

^a^Minor allele defined based on control frequency

^b^Observed *P* value

**Table 3 (abstract P3):** *IL-18* genotype frequencies

SNP	Genotype	Cases^a^	Controls^a^	*P* ^b^	OR (95% CI)
rs1946519	C/C	66 (0.28)^c^	114 (0.48)	<0.001	1.00 (reference)
	C/A	120 (0.51)	97 (0.41)		2.11 (1.42–3.15)
	A/A	49 (0.21)	24 (0.10)		3.19 (1.82–5.60)
rs187238	G/G	122 (0.52)	126 (0.53)	0.12	1.00 (reference)
	G/C	82 (0.35)	92 (0.39)		0.86 (0.59–1.25)
	C/C	31 (0.13)	19 (0.08)		1.61 (0.90–2.90)
rs360718	A/A	132 (0.56)	127 (0.54)	0.61	1.00 (reference)
	A/C	82 (0.35)	89 (0.38)		0.84 (0.58–1.22)
	C/C	21 (0.09)	19 (0.08)		1.05 (0.56–1.96)
rs360717	G/G	82 (0.35)^c^	120 (0.51)	<0.001	1.00 (reference)
	G/A	78 (0.33)	89 (0.38)		1.22 (0.83–1.80)
	A/A	75 (0.32)	26 (0.11)		4.08 (2.51–6.64)

^a^A total of 235 RPL cases and 235 control subjects were included

^b^Pearson’s chi squared test

^c^Number of subjects (frequency)

## P4 Effect of environmental factors on gene-gene and gene-environment reactions: model and theoretical study applied to environmental interventions using genotype

### S. Abdalla^1^, M. Nabil Al-Aama^2^

#### ^1^Department of Physics, Faculty of Science, King Abdulaziz University Jeddah, P.O. Box 80203, Jeddah 21589, Saudi Arabia; ^2^Internal Medicine and Cardiology, King Abdulaziz University Medical School, Jeddah, Saudi Arabia

##### **Correspondence:** S. Abdalla (smabdullah@kau.edu.sa) – Department of Physics, Faculty of Science, King Abdulaziz University Jeddah, P.O. Box 80203, Jeddah 21589, Saudi Arabia

**Background**

Genotype is affected by both the ecological [1, 2] and genetic factors [3] to some known and conjoint diseases of contrast where the size of which genes and environment interact. Neglecting the presence of risk factors, ecological factors will lead to assess the probabilities of some benefits in a definite population sample. Here, there must be a general methodology for the analysis of twin data as the gene interactions (epistasis), the possible weightiness of the gene, and genetic interactions of the environment, and the conditions which stop the presumption of “equal environments” for mono monozygotic and dizygotic twins.

**Materials and methods**

A model to study the genes and gene to gene environment interactions [1, 2] is presented. The classic twin study assumptions, including Fisher’s hypothesis which presumes that genes act as hazard points for combined features in a style needs dominate polygenic added: long way. Provided there will be no confusion when applying: Environmental-, twin- and family- data; results show an issue for every trait or common disease [4]. Every fine-detail that is present through the solution space will match with a different system of the potential hazard and the action of environment and genes. Every fine-detail that is present through the solution space will match with a different system of the potential hazard and the action of environment- [5] and genes [6].

**Conclusions**

The present data showed the possibility of limiting the spread of conjoint diseases when applying environmental-interactions to certain gene. Our results emphasize the significance of the genetic makeup of the individual when estimating the possible hazards of complex diseases is overstated [3, 4]. Moreover, when the phenomena are a powerful, genetically, because of epistasis, environmental explanation for the large risk of common brotherhood is reasonable, even when considering high heritage typical cases. Thus, the results confirm the potential the previously rejected on the light of the data of the case twin. Thus, genetic models of family assembly may be incorrect and chase additional genes can be counter-productive to a large extent.

**References**

1. Abdalla S, Al-Hadeethi Y., **Gene’s alternations with exposure time of environmental factors**, *Gene*. 2013 Oct 10; **528**(2):256-60. doi:10.1016/j.gene.2013.06.065. Epub 2013 Jul 13.

2. Abdalla S, Al-Hadeethi Y and El-Shewy E K, **Effect of environmental factors on gene alterations in complex diseases: simulation study using Gene-Environment iNteraction Simulator 2**, *BMC Genomics* 2014, **15**(Suppl. 2):P47 doi:10.1186/1471-2164-15-S2-P47

3. Abdalla S, M Letarte, **Hereditary haemorrhagic telangiectasia: current views on genetics and mechanisms of disease**, *J Med Genet*. 2006 February; **43**(2): 97–110. doi:10.1136/jmg.2005.030833, PMCID: PMC2603035

4. Veronique MM Vorselaars, Sebastiaan Velthuis, Repke J Snijder, Jan Albert Vos, Johannes J Mager, Martijn C Post, **Pulmonary hypertension in hereditary haemorrhagic telangiectasia**, *World J Cardiol*. 2015 May 26; **7**(5): 230–237. Published online 2015 May 26. doi:10.4330/wjc.v7.i5.230, PMCID: PMC4438464

5. Ferdos Alaa el Din, Sylvie Patri, Vincent Thoreau, Montserrat Rodriguez-Ballesteros, Eva Hamade, Sabine Bailly, Brigitte Gilbert-Dussardier, Raghida Abou Merhi, Alain Kitzis, **Functional and splicing defect analysis of 23 ACVRL1 mutations in a cohort of patients affected by Hereditary Hemorrhagic Telangiectasia**, PMCID: PMC4503601

6. Abdalla S, Cymerman U, Rushlow D, Chen N, Stoeber GP, Lemire EG, Letarte M., **Novel mutations and polymorphisms in genes causing hereditary hemorrhagic telangiectasia**, *Hum Mutat.* 2005 Mar;**25**(3):320-1.

## P5 Genomics and transcriptomic analysis of imatinib resistance in gastrointestinal stromal tumor

### Asmaa Elzawahry^1^, Tsuyoshi Takahashi^2^, Sachiyo Mimaki^3^, Eisaku Furukawa^1^, Rie Nakatsuka^2^, Isao Kurosaka^1^, Takahiko Nishigaki^2^, Hiromi Nakamura^4^, Satoshi Serada^4^, Tetsuji Naka^5^, Seiichi Hirota^6^, Tatsuhiro Shibata^5^, Katsuya Tsuchihara^3^, Toshirou Nishida^7^, Mamoru Kato^1^

#### ^1^Department of Bioinformatics, National Cancer Center Research Institute, 5-1-1 Tsukiji, Chuuoo-ku, Tokyo 104-0045, Japan; ^2^Department of Gastroenterological Surgery, Osaka University Graduate School of Medicine, 2-2 E2, Yamadaoka, Suita City, Osaka, 565-0871, Japan; ^3^Division of Translational Research, Exploratory Oncology Research and Clinical Trial Center, National Cancer Center, 6-5-1 Kashiwanoha, Kashiwa, Chiba 277-8577, Japan; ^4^Division of Cancer Genomics, National Cancer Center Research Institute, 5-1-1 Tsukiji, Chuuoo-ku, Tokyo 104-0045, Japan; ^5^Laboratory for Immune Signal, National Institute of Biomedical Innovation, 7-6-8 Saito-Asagi, Ibaraki City, Osaka, 567-0085, Japan; ^6^Department of Surgical Pathology, Hyogo Medical College, 1-1, Mukogawa-cho, Nishinomiya City, Hyogo, 663-8501, Japan; ^7^Department of Surgery, National Cancer Center Hospital East, 6-5-1 Kashiwanoha, Kashiwa, Chiba, 277-8577, Japan

##### **Correspondence:** Mamoru Kato (mamkato@ncc.go.jp) – Department of Bioinformatics, National Cancer Center Research Institute, 5-1-1 Tsukiji, Chuuoo-ku, Tokyo 104-0045, Japan

**Background**

The gastrointestinal stromal tumor (GIST) is the most common frequent mesenchymal tumor of the digestive tract. GIST proliferation is driven by gain-of-function mutations in *KIT*. These characteristics have facilitated the targeted therapies development that based on with tyrosine kinase inhibitors, such as imatinib. Although many clinical studies have demonstrated revolutionized effects of imatinib, more than 80 % of patients lastly develop disease progression driven by secondary resistance mutations in *KIT* kinase domains. However, the full spectrum of genomic and transcriptomic changes behind the resistance remains unknown.

**Results**

This study analyzed genomic and transcriptomic changes in drug-sensitive and -resistant cell lines against imatinib. We also looked at an “intermediate” cell-line before reaching the full resistance. We identified SNVs and CNAs from the next-generation sequencing and also the transcriptome from microarrays. For clinical insights, we conducted exome sequencing for two clinical samples with the resistance. Notably, the cell line briefly exposed to imatinib exhibited drastic transcriptional changes, but few genomic changes.

**Conclusions**

We suggest that pre-existing cell death-resistant subpopulations are the main cause for full resistance via secondary KIT mutations. The combination of chemotherapy with imatinib and apoptosis pathway-targeting drugs, could limit the emergence of drug-resistant cancer.

## P6 *In-Silico* analysis of putative HCV epitopes against Pakistani human leukocyte antigen background: an approach towards development of future vaccines for Pakistani population

### Sajid Mehmood, Naeem Mahmood Ashraf, Awais Asif, Muhammad Bilal, Malik Siddique Mehmood, Aadil Hussain

#### Clinical Biochemistry Group, Department of Biochemistry & Molecular Biology, University of Gujrat, Punjab, Pakistan

##### **Correspondence:** Sajid Mehmood (Sajid.mehmood@uog.edu.pk) – Clinical Biochemistry Group, Department of Biochemistry & Molecular Biology, University of Gujrat, Punjab, Pakistan

**Background**

Mounting burden of HCV infected individuals and soaring cost of treatment is serious source of unease for developing countries. Numbers of various approaches have been anticipated to develop vaccine against HCV but majority of them proved ineffective. Development of vaccine by considering geographical distribution of HCV genotypes and host genetics shows potential. In this research article, we have tried to predict most putative HCV epitopes which are efficiently restricted by most common HLA alleles in Pakistani population through different computational algorithms.

**Results**

Thirteen selected, experimentally identified epitopes sequences were used to derived consensus sequences in all genotypes of HCV. Obtained consensus sequences were used to predict their binding affinities with most prevalent HLA alleles in Pakistani population. Three Class-I from core, NS5B and NS4B region and one class-II epitopes from NS3 region showed effective binding and proved to be highly putative to boast immune response. Cocktail of these four have been checked for population coverage and they gave 81.59 % for Pakistani Asian and 76.63 % for Pakistani Mixed populations.

**Conclusions**

Computational algorithems are robust way to shortlist potentail candidate epitopes for vaccine development but further, in vivo and in vitro studies are required to confirm their immunogenic properties.

## P7 Inhibition of AChE and BuChE with the natural compounds of *Bacopa monerri* for the treatment of Alzheimer’s disease: a bioinformatics approach

### Qazi Mohammad Sajid Jamal^1^, Mughees Uddin Siddiqui^1^, Mohammad A. Alzohairy^2^, Mohammad A. Al Karaawi^2^

#### ^1^Department of Health Information Management, College of Applied Medical Sciences, Buraydah Colleges, Al Qassim, Saudi Arabia; ^2^Department of Medical Laboratory, College of Applied Medical Sciences, Buraydah Colleges, Al Qassim, Saudi Arabia

##### **Correspondence:** Qazi Mohammad Sajid Jamal (qazi.bioinformatics@gmail.com) – Department of Health Information Management, College of Applied Medical Sciences, Buraydah Colleges, Al Qassim, Saudi Arabia

**Background**

Alzheimer’s disease (AD) is the neurodegenerative diseases and there are no perfect treatments. The pathogenesis of AD is directly linked to the presence of two deposits, senile plaques (SPs), neurofibrillary tangles (NFTs), and cholinergic abnormalities. Acetylcholine receptors stimulation is essential for the cure of AD. In this perspective, the role of AChE inhibitors, which get better cognitive functions, are presently accepted for its treatment. Cholinesterase inhibitors a compound that works as a neurotransmitter in the Central Nervous System avert the metabolism of acetylcholine (a-SEA-til-KOH-lean). Individuals with dementia generally have lower levels of this compound, which is essential for the processes of remembrance, thinking, and interpretation. Cholinesterase inhibitors slow the breakdown of acetylcholine. Therefore, in the current study the natural compounds of Bacopa *monerri* have been used as an acetyl cholinesterase inhibitor which could play an important role in the treatment of AD after further *in vitro* or *in vivo*, and other related investigations.

**Results**

The molecular interaction analysis to explore the binding pattern of *Bacopa monneiri* compounds with the 3-D structures of AChE and BuChE indicates that bacoside X, bacoside A, 3-beta-D-glucosylstigmasterol and Daucosterol could be good inhibitors on the basis of the obtained binding energies (i.e. with AChE -15.44, **-16.22**, and -13.96 Kcal/mol respectively, and with BuChE -16.23, -15.37 and -15.27 kcal/mol respectively) and inhibition constants (i.e. with AChE 3.32 *nM*, 11.13 *nM* and 24.63 *nM* and with BuChE 879.51 *pM*, 2.29 *nM* and 4.43 *nM* respectively).These results would be useful for further designing of the AChE and BuChE inhibitors with high activity in the treatment of the AD.

**Conclusions**

Therefore, our study indicates that the inhibitory constants of the aforesaid natural compounds of *Bacopa* can be utilized for the development of inhibitors.

**Acknowledgements**

The authors are thankful to Buraydah Colleges for providing all the necessary facilities to carry out this work.

**References**

1. T. Kihara: **Alzeimer’s Disease and Acetycholine Receptors.***Acta Neurobiol Exp* 2004, 64: 99-105.

2. Pam, Jennifer L: **Active Site Gating and Substrate Specificity of Butyrylcholineasterase and Acetylcholinsterase: Insights from Molecular Dynamics Simulation.***J Phys Chem B.* 2011, **115(27)**:8797-8805.

3. Morris, G.M: **Automated Docking using Lamarkian Genetic Algorithm and an Empirical Binding Free Energy Function.** J Comp Chem 1998, **19**:1639-1662.

## P8 Her2 expression in urothelial cell carcinoma of the bladder in Saudi Arabia

### Taoufik Nedjadi^1^, Jaudah Al-Maghrabi^2^, Mourad Assidi^3^, Heba Al-Khattabi^3^, Adel Al-Ammari^4^, Ahmed Al-Sayyad^5^, Abdelbaset Buhmeida^3^, Mohammed Al-Qahtani^3^

#### ^1^King Fahd Medical Research Centre, KAU, Jeddah, KSA; ^2^Department of Pathology, Faculty of Medicine, KAU, Jeddah, KSA; ^3^Centre for Excellence in Genomic Medicine Research, Jeddah, KSA; ^4^Department of Urology, King Faisal Specialist Hospital and research Centre, Jeddah, KSA; ^5^Department of Urology, KAUH, Jeddah, KSA

##### **Correspondence:** Abdelbaset Buhmeida (abuhme@utu.fi) – Centre for Excellence in Genomic Medicine Research, Jeddah, KSA

**Background**

High levels of Human epidermal growth factor receptor (Her2) have been associated with cancer development and poor prognosis in many cancer types. In bladder carcinoma (BC), the clinical significance of her2 status remains poorly understood. The aim of the current study was to analyze the status of her2 expression and its clinical value as a prognostic marker in BC.

**Subjects and methods**

Bladder cancer samples were collected from patients who underwent surgical resection at King Abdulaziz University Hospital-KSA. One hundred and sixty samples were arranged on a tissue microarray (TMA) and stained using immunohistochemistry (IHC) and bright-field dual in situ hybridization (BDISH) methods. Correlation between the levels of her2 protein expression/gene amplification and patients’ clinical parameters were evaluated.

**Results**

Using Immunohistochemistry, Her2 staining was expressed in the cytoplasm and cell membrane. Her2 was detected in 85 % of patients and over-expressed (+3) in 24 % of BC cohort. Overexpression of her2 protein was significantly associated with tumour staging (*p* = 0.002), lymph node invasion (*p* = 0.04) and vascular invasion (*p* = 0.01). BDISH analysis revealed that *her2* gene is amplified (score of 2+) in 25 % of patients. Significant correlation between *her2* gene amplification and tumour grade was reported (*p* = 0.03). Interestingly, amplification of *her2* gene was significantly associated with poor cancer-specific survival in bladder cancer (p < 0.04, log-rank test).

**Conclusions**

High concordance between the IHC and BDISH data was shown. Her2 might serve as a potential marker for cancer metastasis and poor prognosis. This finding also indicates that high proportion of bladder cancer patient could potentially benefit from anti-her2 targeted therapies.

**Acknowledgements**

This work was financially supported by King Abdulaziz City for Science and Technology (KACST) under research no (Bio-10-1260-03) and (ات-33-26.).

## P9 Association of angiotensinogen single nucleotide polymorphisms with Preeclampsia in patients from North Africa

### Hédia Zitouni^1,2^, Nozha Raguema^1,2^, Marwa Ben Ali^1,2^, Wided Malah^3^, Raja Lfalah^3^, Wassim Almawi^4^, Touhami Mahjoub^1^

#### ^1^Research’s Laboratory of Human Genome and Multi-factorial diseases LR12ES07, College of Pharmacy, Monastir, Tunisia 5000; ^2^Faculty of Sciences, Bizerte, Tunisia 7021; ^3^Maternity and Neonatal Centre, Monastir, Tunisia 5000; ^4^Department of Medical Biochemistry, College of Medicine and Medical Sciences; Arabian Gulf University, P.O. Box 22979, Manama, Bahrain

##### **Correspondence:** Hédia Zitouni (hediaztn@gmail.com) – Faculty of Sciences, Bizerte, Tunisia 7021

**Background**

Preeclampsia (PE) is a major pregnancy complication, associated with maternal and fetal morbidity and mortality, and affects about 2-8 % of pregnancies worldwide (1, 2). PE is characterized by onset of proteinuria and hypertension after 20^th^ week of gestation. PE is a documented risk factor for preterm birth and intrauterine growth retardation (IUGR), and is a multi-factorial disorder involving both modifiable and non-modifiable factors. The latter include specific mutations in gene implicated in blood pressure control. These include, angiotensinogen (*AGT*) gene, which play a key role in blood pressure regulation. This study examined the association between PE and the *AGT* polymorphismsM235T and T174M in (North African) Tunisian population.

**Materials and methods**

**Fig. 2 (abstract P9):**
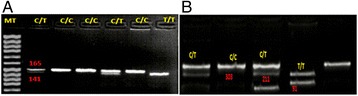
Agarose gel electrophoresis, (**a**): M235T-AGT genotypes, Lane C/T: heterozygotes M235/235 T, Lane C/C: homozygous M235/M235, Lane T/T: homozygous 235 T/235 T, MT:DNA molecular marker (**b**): T174M-AGT genotypes, Lane C/T: heterozygotes T174/174 M, Lane C/C:homozygous T174/T174, Lane T/T: homozygous 174 M/174 M

This case-control study included 300 unrelated Tunisian women with PE, and 300 unrelated age- and ethnically-matched control women. M235T and T174M genotyping was done by RFLP-PCR (Fig. 2). Haplotype analysis was investigated using SNPstats software.

**Results**

Significant association between PE and M235T [*P* = 0.0034; OR (95 %) = 2.77(1.35 -5.68)], and T174M [*P* = 0.01; OR (95 %) = 0.52 (0.32-0.98)] was seen in the studied population (Tab1e 1). Two-locus haplotype analysis demonstrated significant association with the 235 T and 174Mhaplotypes [*P* = 0.0051; OR (95 %) = 1.56 (1.15-2.13)].

**Conclusions**

M235T and T174M variants, especially the T235 allele, contribute to an increased risk of developing PE in (North African) Tunisians.

**Table 4 (abstract P9):** Association of *AGT* genotypes with PE

SNP	Genotype	Patients	Controls	*P*	OR (95 % CI)	*P*	aOR (95 % CI)
rs699	C/C	137 (0.50)^*1*^	176 (0.63)	0.003	1.00 (Reference)	0.006	1.00 (Reference)
	C/T	109 (0.40)	90 (0.32)		1.56 (1.09 – 2.22)		1.79 (1.18 – 2.71)
	T/T	26 (0.10)	12 (0.04)		2.78 (1.36 – 5.72)		2.43 (1.05 – 5.63)
rs4762	C/C	196 (0.89)	167 (0.82)	0.015	1.00 (Reference)	0.031	1.00 (Reference)
	C/T	22 (0.10)	37 (0.18)		0.51 (0.29 – 0.89)		0.49 (0.25 – 0.96)
	T/T	2 (0.01)	0 (0.00)		NA		NA

1 Number (frequency)

2 aOR = adjusted OR, adjusted for BMI, gestation, and baby weight

**Trial registration**

Current Controlled Trials

**References**

1. Roberts JM, Redman CW. **Preeclampsia: more than pregnancy-induced hypertension**. *Lancet*. 1993; 341:1447–1451.

2. Eiland E, Nzerue C, Faulkner M. **Preeclampsia:***J Pregnancy*. **2012**:586578.

## P10 Systems biology analysis reveals relations between normal skin, benign nevi and malignant melanoma

### Mohammed Elanbari, Andrey Ptitsyn

#### Sidra Medical and Research Center, PO Box 26999, Doha, Qatar

##### **Correspondence:** Andrey Ptitsyn (aptitsyn@sidra.org) – Sidra Medical and Research Center, PO Box 26999, Doha, Qatar

**Background**

Melanoma is among the most prevalent types of cancer worldwide. Malignant melanoma is aggressive, hard to treat and presents a significant challenge for early diagnostics. Better understanding of molecular mechanisms underlying the development and function of malignant melanoma is essential for improvement of diagnostic technology.

**Results**

In this study we used previously published and publicly available data on microarray patterns of gene expression in normal skin, benign nevi and malignant melanoma samples. We have applied an alternative analysis strategy designed to highlight molecular functions and regulatory pathways rather than separate biomarkers characteristic of melanoma. As anticipated, we report the three classes of samples are separated from each other by specific molecular mechanisms. However, the relation between normal skin samples, nevi and melanoma show striking similarity to previously described relation between normal, primary tumor and metastatic tumor samples in other types of cancer. We also present the evidence that in gene expression space some benign nevi samples are located closer to the cluster of melanoma samples, which may be indicative of their high metastatic potential detectable on the early stages of development.

**Conclusions**

Our analysis points at similarity of functional patterns of gene expression between nevi and malignant melanoma. We hypothesize that the pathways differentially activated between nevi and melanoma are responsible for metastatic progression.

## P11 The apoptotic effect of thymoquinone in Jurkat cells

### Sana Mahjoub^1^, Rabeb El Ghali^1^, Bechir Achour^1^, Nidhal Ben Amor^1^, Mourad Assidi^2^, Brahim N’siri^1,^ Hamid Morjani^3^

#### ^1^Laboratory of Human Genome and Multifactorials diseases, Faculty of Pharmacy, Monastir, Tunisia; ^2^Center of Excellence in Genomic Medicine Research (CEGMR), King Abdulaziz University, Jeddah, Kingdom of Saudi Arabia; ^3^Research Unit Extracellular Matrix and Cellular Dynamic, Faculty of Pharmacy, Monastir, Tunisia

##### **Correspondence:** Sana Mahjoub (sanamahjoub0903@gmail.com) – Laboratory of Human Genome and Multifactorials diseases, Faculty of Pharmacy, Monastir, Tunisia

**Background**

Leukemia is a cancer of blood cells. This malignant disease is a heavy burden for patients and the health system worldwide. According to the American Cancer Society estimates, about 48,610 new cases and about 23,720 deaths occurred in the United States in 2013 [1, 2]. It is the most common pediatric cancer, representing approximately 25 % of cancers diagnosed in children aged <20 years [3]. There have been dramatic improvements in blood cancer treatment using chemotherapy, ionizing radiation, radio immunotherapy, immunotherapy, gene therapy, and stem cell transplantation [4]. However, most of these therapies are plagued with side effects, and almost all cause cytotoxicity in healthy cells [5]. These findings make discovery of new treatments measures for leukemia very imperative. Natural compounds have been an important source of drugs since ancient times. Thymoquinone (TQ) is the major bioactive compound of the essential oil of *Nigella sativa* which have many anticancer effects. The aim of this study is to analyze the potential of TQ to induce apoptosis in Jurkat cells. The cytotoxicity was evaluated by MTT assay at different concentration of TQ to determinate the IC50 (half maximal inhibitory concentration). Apoptosis effect was analyzed by annexin V and caspase3/7 assays at both IC50 and 1 μM.

**Results**

Our results reported an IC50 of 210 nM for TQ. We showed also that the number of labeled cells by annexin V and caspase 3/7 activity are proportional to TQ concentration.

**Conclusions**

TQ has an apoptotic activity which is concentration dependent. It is an interesting anticancer agent extracted from natural compounds. However, further *in vitro* investigations are required to optimize its effect and assess possible side effects.

References

1. Udensi UK, Tchounwou PB: **Dual effect of oxidative stress on leukemia cancer induction and treatment**. *Journal of experimental & clinical cancer research : CR* 2014, **33**:106.

2. Siegel R, Naishadham D, Jemal A: **Cancer statistics, 2013**. *CA: a cancer journal for clinicians* 2013, **63**(1):11-30.

3. Sun W, Gaynon PS, Sposto R, Wayne AS: **Improving access to novel agents for childhood leukemia**. *Cancer* 2015, **121**(12):1927-1936.

4. Nabhan C, Rosen ST: **Chronic lymphocytic leukemia: a clinical review**. *JAMA : the journal of the American Medical Association* 2014, **312**(21):2265-2276.

5. Rahman HS, Rasedee A, How CW, Zeenathul NA, Chartrand MS, Yeap SK, Abdul AB, Tan SW, Othman HH, Ajdari Z *et al*: **Antileukemic effect of zerumbone-loaded nanostructured lipid carrier in WEHI-3B cell-induced murine leukemia model**. *International journal of nanomedicine* 2015, **10**:1649-1666.

**Fig. 3 (abstract P11):**
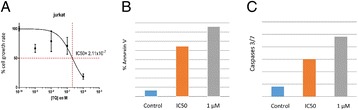
Assessment of apoptotic effect of Thymoquinone (TQ) by MTT assay using different concentrations (Control, IC50 and 1 μM). **a**: The IC_50_ of TQ in Jurkat cells; **b**: Percentage of Annexin V positive cells; **c**: Percentage of caspase 3/7 positive cells

## P12 Sonic hedgehog contributes in bladder cancer invasion in Saudi Arabia

### Taoufik Nedjadi^1^, Adel Al-Ammari^2^, Ahmed Al-Sayyad^3^, Nada Salem^4^, Esam Azhar^1^, Jaudah Al-Maghrabi^5^

#### ^1^King Fahd Medical Research Centre, KAU, Jeddah, Saudi Arabia; ^2^Department of Urology, King Faisal Specialist Hospital and research Centre, Jeddah, Saudi Arabia; ^3^Department of Urology, KAUH, Jeddah, Saudi Arabia; ^4^Centre for Excellence in Genomic Medicine Research, Jeddah, Saudi Arabia; ^5^Department of Pathology, Faculty of Medicine, KAU, Jeddah, Saudi Arabia

##### **Correspondence:** Taoufik Nedjadi (tnedjadi@kau.edu.sa) – King Fahd Medical Research Centre, KAU, Jeddah, Saudi Arabia

**Background**

Sonic hedgehog gene (SHH) is a key regulator at an early embryonic development. Recent findings revealed a constitutive activation of the SHH pathway in several malignancies including bladder cancer. The role of SHH in bladder pathogenesis may be related to increased stemness and EMT. We here investigated the clinical and pathological significance of SHH in bladder cancer.

**Materials and methods**

The expression pattern of SHH protein was examined in 160 patients with bladder cancer using immunohistochemistry (IHC). Correlation analysis of the SHH status with clinicopathological parameters was performed using SPSS.

**Results**

SHH protein was overexpressed in 46 % of bladder cancer patients. The expression of SHH was significantly associated with lymph node invasion (p = 0.02) and with distant metastasis (p = 0.034). In univariate analysis, there was no relationship between SHH expression with other parameters including tumor grade, stage, age, gender, cancer type and survival.

**Conclusions**

Our data support previous findings and revealed that SHH contribute in bladder tumorigenesis. Further research is needed to investigate the functional significance of SHH in bladder cancer.

**Acknowledgements**

This work was financially supported by King Abdulaziz City for Science and Technology (KACST) under research no (ات-33-26.).

## P13 Association of Interleukin 18 gene promoter polymorphisms - 607A/C and -137 G/C with colorectal cancer onset in a sample of Tunisian population

### Vera Chayeb^1,2^, Maryam Dendena ^1^, Hedia Zitouni ^1,2^, Khedija Zouari-Limayem ^3,4^, Touhami Mahjoub^1^

#### ^1^Human Genome and Multifactorial Diseases Laboratory, Faculty of Pharmacy, University of Monastir, Monastir 5000, Tunisia; ^2^Faculty of Science of Bizerte, University of Carthage, Jarzouna 7021, Tunisia; ^3^Research Unit: Colorectal cancer in Young Subjects - Faculty of Medecine, University of Monastir, Monastir 5000, Tunisia; ^4^Surgery Department, University Hospital Fattouma Bourguiba, University of Monastir, Monastir 5000, Tunisia.

##### **Correspondence:** Vera Chayeb (vchaieb@yahoo.fr) – Faculty of Science of Bizerte, University of Carthage, Jarzouna 7021, Tunisia

**Background**

Inflammation is considered both as a cause and consequence of cancer. Litterature has shown that the imbalance between pro-inflammatory and anti-inflammatory cytokines could modulate colorectal carcinogenesis [1]. Interleukin 18 is a pro-inflammatory cytokin, which level was reported to be increased in cancer disease [2,3] .We hypothesised that it can be genetically controlled by polymorphisms located in the promoter region .

**Materials and methods**

We had conducted a case-control study of 148 subjects: 74 patients and 74 healthy volunteers of Tunisian origin. DNA isolation was made by Miller’s method. Genetic profiling was performed of the RFLP-PCR assay of the -607 A/C and -137 G/C polymorphism of Interleukin 18 gene. Statistical Analysis was conducted by the EPIINFO v.7.1.2.0 statistical software.

**Results**

Our work has demonstrated a significant association of the A/A genotype of the -607 A/C polymorphism under the additive and recessive models of genetic transmission by the onset colorectal cancer (Table 5). In contrast, no association has been observed betwwen the -137 G/C (Table 6) promoter polymorphism and colorectal cancer.

**Conclusions**

This paperwork has demonstrated that promoter polymorphism -607 A/C seems to play a role in the colorectal cancer development in the studied Tunisian sample population.

**Acknowledgements**

We would like to thank Ms. Saad Hanen and Prof.Abdelaziz Hamdi for their collaborations.

**References**

1. Duluc D, Corvaisier M, Blanchard S, Catala L: **Interferon-gamma reverses the immunosuppressive and protumoral properties and prevents the generation of human tumor-associated macrophages**. *Int J Cancer.* 2009, **25(2)**: 367-73.

2. Pages F, Vives V, Sautès-Fridman C: **Control of tumor development by intratumoral cytokines.***Immunol Lett.* 1999, **68 (1)** :135–139.

3. Giedraitis V, He B, Xin Huang W: **Cloning and mutation analysis of the human IL-18 promoter: a possible role of polymorphisms in expression regulation.***J Neuroimmunol.* 2001, **112(1-2)**:146–152.

**Table 5 (abstract P13):** Genotypic distributions of the -607A/C polymorphism under the three models of genetic transmission

Model	Genotype	Controls	Cases	OR [CI _95 % _]*	p-value**
Additive	A/A	7 (9.4 %)	17(22.9 %)	2.87 [0.94-9.38]	0.038
	A/C	35 (47.2 %)	30(40.5 %)	1.01 [0.47 -2.18]	0.891
	C/C	32 (43,2 %)	27 (36.4 %)	1	-
Dominant	A/A - A/C	42(56.6 %)	47(63.4 %)	1.32 [0.65-2.707]	0.5
	C/C	32(43.2 %)	27 (22.9 %)	1	-
Recessive	A/A	7(9.4 %)	17(22.9 %)	2.85 [1.02-8.68]	0.02
	A/C - C/C	67(90.4 %)	57(76.9 %)	1	-

*Fisher exact Odds Ratio

**Fisher exact p-value

**Table 6 (abstract P13):** Genotypic distributions of the -137G/C polymorphism under the three models of genetic transmission

Model	Genotype	Control	Cases	OR [CI_95%_]*	p-value**
Additive	G/G	20 (27 %)	21 (28.3 %)	1	-
	G/C	36 (48.6 %)	30 (40.5 %)	0.79[0.33-1.86]	0.69
	C/C	18 (24.3 %)	23 (31 %)	1.21[0.46-3.17]	0.824
Dominant	G/G	20 (27 %)	21 (28 %)	1	-
	G/C- C/C	54(72.9 %)	53(71.6 %)	0.93[0.42-2.04]	0.85
Recessive	G/G- G/C	56(75.6 %)	51(68.9 %)	1	-
	C/C	18 (24.3 %)	23 (31 %)	1.40(0.64-3.09)	0.36

*Fisher exact Odds Ratio **Fisher exact p-value

## P14 Pathological expression of interleukin-6, -11, leukemia inhibitory factor and their receptors in tubal gestation with and without tubal cytomegalovirus infection

### Bassem Refaat^1^, Ahmed M Ashshi^1^, Sarah A Batwa^2^

#### ^1^Laboratory Medicine Department, Faculty of Applied Medical Sciences, Umm Al-Qura University, Makkah, KSA; ^2^Obstertics and Gynaecology Department, Maternity and Children Hospital, Jeddah, KSA

##### **Correspondence:** Bassem Refaat (bassem.refaat@yahoo.co.uk) – Laboratory Medicine Department, Faculty of Applied Medical Sciences, Umm Al-Qura University, Makkah, KSA

**Background**

Little is known about the pathogenic mechanisms underlying cytomegalovirus (CMV) induced fallopian tube (FT) damage and ectopic pregnancy (EP) [1, 2]. This study measured the prevalence of CMV infection in EP and its impact on the production of interleukin (IL)-6 family members and their corresponding receptors (IL6R, IL11R & LIFR) in Fallopian tubes (FT) with and without EP.

**Materials and methods**

Fresh FTs were obtained from 84 women with EP, 20 total abdominal hysterectomy (TAH) during the midluteal phase and 31 tubal ligations. Tubal infection with CMV was detected by an IVD CE PCR kit. The participants were then categorised according to their CMV results and he candidate molecules were measured by realtime RT-PCR and immunohistochemistry in tubes with EP and were CMV-positive (n = 15) and the results were compared with those obtained from EP (n = 15) and TAH (n = 15) and were negative for the virus.

**Results**

**Fig. 4 (abstract P14):**
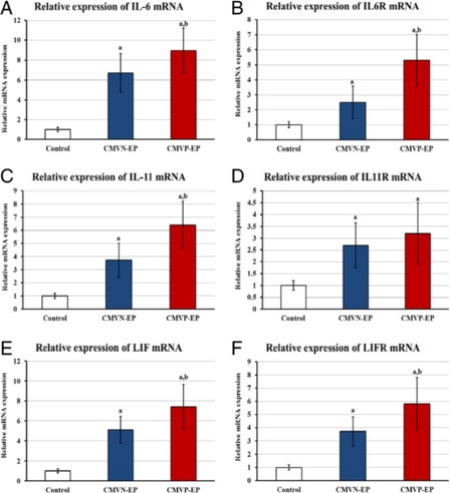
Mean ± SD of messenger RNA relative expression of **a** IL-6, **b** IL6RA, **c** IL-11, **d** IL11RA, **e** LIF and **f** LIFR, in Fallopian tube collected from control, EP negative for CMV (CMVN-EP) and EP positive for CMV (CMVP-EP) groups. (^a^ P < 0.05 compared with control & ^b^ P < 0.05 compared with CMVN-EP group)

The frequency of viral infection was higher (P = 0.01) in EP (21.4 %) than controls (5.9 %) and EP samples simultaneously positive for CMV had higher expression of all candidate cytokines and their receptor, except for IL11R, at the gene (Fig. 4) and protein (Table 7) levels compared with negative EP and TAH.

**Conclusions**

CMV infection of FT appears to be involved in the pathogenesis of EP by increasing the production of members of IL-6 family in the FT. Further studies are needed to explorer the function of CMV and candidate cytokines in the pathogenesis of tubal pregnancy.

**Acknowledgements**

This project was funded by the National Science, Technology and Innovation Plan (MARRIFAH) - King Abdul Aziz City for Science and Technology (KACST), the Kingdom of Saudi Arabia, Award Number (11-MED2067-10).

**References**

1. Qian HL, Cai T, and Jin HM: **Human cytomegalovirus glycoprotein genotypes in the genital tract tissue of tubal pregnancy patients***. J Int Med Res.* 2009; **37**: 385-91.

2. Refaat B, Ashshi AM, Batwa SA, and El-Shemi AG: **Seroprevalence of Chlamydia trachomatis, cytomegalovirus, herpes simplex virus 1 and 2 in Saudi women with normal and abnormal early pregnancy: A case control study***. African Journal of Microbiology Research*. 2014; **8**: 3565-3569.

**Table 7 (abstract P14):** Mean ± SD of age, gestational age, serum β-hCG and immunohistochemistry for IL-6, IL-11, LIF and their receptors in Fallopian tubes collected from control, CMV negative (CMVN-EP) and positive (CMVP-EP) ectopic pregnancies

	Control	CMVN-EP	CMVP-EP
Age (years)	37.2 ± 5.1	33.3 ± 6.3	31.5 ± 5.4^a^
Gestational age (weeks)	N/A	7.3 ± 1.5	7.6 ± 1.8
β-hCG (IU/L)	ND	2,317 ± 1,964	2,861 ± 2,016
IL-6	188.3 ± 33.1	301.7 ± 34.2^a^	367 ± 41.2^a,b^
IL6Rα	104.4 ± 22.7	179.5 ± 35.8^a^	301.3 ± 67.3^a,b^
IL-11	182.3 ± 36.4	251.9 ± 36.4	367.5 ± 41.1^a,b^
IL11Rα	149.7 ± 22.6	230.6 ± 42.8^a^	241.2 ± 51.4^a^
LIF	205.5 ± 31.2	277.8 ± 49.8^a^	344.4 ± 58.6^a,b^
LIFR	188.1 ± 33.6	264.9 ± 47.2^a^	327.4 ± 53.1^a,b^

^N/A^not applicable; ^ND^ not done; ^a^P < 0.05 compared with control & ^b^P < 0.05 compared with CMVN-EP group)

## P15 Phenotypic and genetic profiling of avian pathogenic and human diarrhegenic *Escherichia coli* in Egypt

### Hazem Ramadan^1^, Amal Awad^2^ and Ahmed Ateya^3^

#### ^1^Hygiene and Zoonoses Department, Faculty of Veterinary Medicine, Mansoura University, Mansoura 35516, Egypt; ^2^Bacteriology, Mycology and Immunology Department, Faculty of Veterinary Medicine, Mansoura University, Mansoura 35516, Egypt; ^3^Animal Husbandry and Animal Wealth Development Department, Faculty of Veterinary Medicine, Mansoura University, Mansoura 35516, Egypt

##### **Correspondence:** Hazem Ramadan (hazemhassan84@yahoo.com) – Hygiene and Zoonoses Department, Faculty of Veterinary Medicine, Mansoura University, Mansoura 35516, Egypt

**Background**

This study was carried out to identify the coexistence of phenotypes, intestinal virulent genes and phylotypes between human diarrhegenic *E. coli* (DEC) and avian pathogenic *E. coli* (APEC).

**Materials and methods**

A total of 36 diseased chickens (3 visceral organs; liver, spleen and heart per each) were collected from different poultry farms located in the district of Mansoura, Egypt, during the first quarter of 2015. Seventy-eight human stool samples (50 diarrheic patients and 28 healthy persons) were taken from a small charity hospital laboratory in the district of Mansoura, Egypt during the second quarter of 2015. Culturing, phenotyping, PCR screening of intestinal virulent markers (intimin and shiga toxin genes) and phylogrouping were performed.

**Results**

From 186 chicken visceral (108) and human stool (78) samples, sixty five (35.3 %) biochemically identified *E.coli* isolates were detected from chicken (29/108; 26.9 %) and human samples (36/78; 46.2 %). Seventeen phenotypes were determined from human and chicken isolates and the most common serotypes distinguished from APEC isolates were O78, O2 and O1. Importantly, only 2 similar serotypes (O119:H4 and O26:H11) were identified from both APEC and human DEC isolates. Molecularly, the respective percentages of 100 and 35 with intimin (*eae*) and Shiga toxin genes were detected from APEC isolates while 50 %, 27.8 % and 19.4 % of human DEC isolates harbored *eae*, *stx1* and *stx2* genes respectively. Phylogrouping revealed the significant (P < 0.05) higher occurrence of pathogenic phylogroups (D and B2) in APEC (19/29, 65.5 %) than human DEC isolates (8/36, 22.2 %).

**Conclusions**

Our results verified that APEC isolates shared serotypes, virulent genes and phylotypes with DEC isolates with a subsequent potential public health concern. Also, the implementation of PCR based methods along with the conventional cultural methods is beneficial. To the best of our knowledge, this is the first report in Egypt that determines virulent genes and phylogroups concomitance between APEC and DEC isolates.

## P16 Cancer-targeting dual gene virotherapy as a promising therapeutic strategy for treatment of hepatocellular carcinoma

### Adel Galal Ahmed El-Shemi^1,2^, Ahmad Ashshi^1^, Mohammed Basalamah1, Youjin Na^3^, Chae-Ok YUN^3^

#### ^1^Department of Laboratory Medicine, Faculty of Applied Medical Sciences, Umm Al-Qura University, PO Box 7607, Holy Makkah, Kingdom of Saudi Arabia; ^2^Department of Pharmacology, Faculty of Medicine, Assiut University, Asyut, Egypt; ^3^Department of Bioengineering, College of Engineering, Hanyang University, 222 Wangsinmi-ro, Seongdong-gu, Seoul 133-791, Republic of Korea

##### **Correspondence:** Adel Galal Ahmed El-Shemi (dr_adel_elshemy2006@yahoo.com) – Department of Pharmacology, Faculty of Medicine, Assiut University, Asyut, Egypt

**Background**

Hepatocellular carcinoma (HCC) is a highly fatal cancer without effective therapy. Recently, a novel strategy known as cancer-targeting dual gene virotherapy (CTDGVT), in which an oncolytic adenoviral vectors (oncolytic Ads) encodes two therapeutic genes, has shown efficient anticancer therapy, whereby, an excellent triplex antitumor effect can be achieved by the viral oncolytic effect on cancer cells and the additive or synergetic interaction between the two expressed anti-tumor genes. Therefore, the present research work is designed to investigate the therapeutic potential, possible synergy and safety profile of this CTDGVT strategy in treatment of human HCC.

**Materials and methods**

Herein, we constructed two oncolytic Ads: one expressing human TRAIL (Ad-ΔB/TRAIL); an apoptotic ligand induces apoptosis in tumor cells; and other expressing human ING4 (Ad-ΔB/ING4), a novel tumour suppressor gene, and then investigated their individual and combined therapeutic efficacy and safety against HCC both in vitro on human HCC cells and in vivo on xenogarfted model of human HCC in nude mice.

**Results**

**Fig. 5 (abstract P16):**
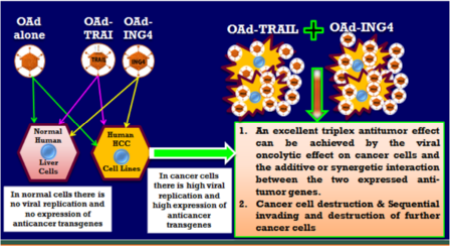
Treatment of hepatocellular carcinoma by using cancer gene-viro-therapy strategy.

Co-therapy with Ad-ΔB/TRAIL and Ad-ΔB/ING4 elicited significant killing effect on HCC cells and growth suppression on the xenogarfted tumor, without overlapping toxicity (Fig. 5). Mechanistically, Ad-ΔB/TRAIL and Ad-ΔB/ING4 had co-operated together to induce apoptosis; stimulate anti-tumor immune response reflected by excess interferon gamma (IFN-γ) production and deep infiltration of natural killer cells (NK1.1 + ve cells) and antigen presenting cells (Cd11 + ve cells); and to inhibit vascular endothelial growth factor (VEGF) and CD31 expression as well as microvessel density in xenografted tumors.

**Conclusions**

Combination therapy with oncolytic Ads expressing human TRAIL and human ING4 genes additively suppressed human HCC both *in vitro* and *in vivo*, via inhibition of tumor angiogenesis and vasculature as well as induction of anti-tumor immunity and apoptosis.

**Acknowledgements**

This project was funded by the National Science, Technology and Innovation Plan (MARRIFAH) - King Abdul Aziz City for Science and Technology (KACST), the Kingdom of Saudi Arabia, Award Number (11-MED2065-10).

## P17 Cancer dual gene therapy with oncolytic adenoviruses expressing TRAIL and IL-12 transgenes markedly eradicated human hepatocellular carcinoma both *in vitro* and *in vivo*

### Adel Galal Ahmed El-Shemi, Ahmad Ashshi, Mohammed Basalamah, Youjin Na, Chae-Ok Yun

#### Department of Laboratory Medicine Department, Faculty of Applied Medical Sciences, Umm Al-Qura University, Makkah, PO Box 7607, Saudi Arabia

##### **Correspondence:** Adel Galal Ahmed El-Shemi (dr_adel_elshemy2006@yahoo.com) – Department of Laboratory Medicine Department, Faculty of Applied Medical Sciences, Umm Al-Qura University, Makkah, PO Box 7607, Saudi Arabia

**Background**

Cancer gene therapy-mediated by oncolytic adenoviruses (Ads) encoding immunostimulatory interleukin-12 (IL-12) gene, or pro-apoptotic tumor necrosis factor-related apoptosis-inducing ligand (TRAIL) gene, have been recently emerged as a novel strategy in cancer therapy. In this study, we generated, and the investigated the single and combination therapy of two oncolytic Ad encoding human TRAIL gene (Ad-ΔB/TRAIL) and oncolytic Ad encoding human IL-12 gene (Ad-ΔB/IL-12) on human hepatocellular carcinoma (human HCC) cell lines and on orthotopic human HCC (Hep3B) model in athymic nude mice.

**Results**

Ad-ΔB/TRAIL and Ad-ΔB/IL-12 combination therapy elicited a more significant cytopathic effect on HCC cells and additive growth suppression of the xenogarfted tumor compared with their single therapy, without overlapping toxicity. Additionally, the augmented anti-HCC activity of Ad-ΔB/TRAIL and Ad-ΔB/IL-12 combination therapy had also resulted in a more activation of the apoptotic-caspase-3 and-8 pathway, overproduction of interferon gamma (IFN-γ), high infiltration rate of natural killer cells and antigen presenting cells. Moreover, Ad-ΔB/TRAIL and Ad-ΔB/IL-12 combination treatment additively reduced vascular endothelial growth factor (VEGF) and CD31 expression as well as the microvessel density in the tumor tissues.

**Conclusions**

Cancer dual gene therapy with Ad-ΔB/TRAIL and Ad-ΔB/IL-12 was synergistically interacted in suppressing human HCC in vitro and in vivo with enhanced activation of anti-tumor immunity and apoptosis, and also enhanced inhibition of tumor angiogenesis and vasculature.

**Acknowledgements**

This project was funded by the National Science, Technology and Innovation Plan (MARRIFAH) - King Abdul Aziz City for Science and Technology (KACST), the Kingdom of Saudi Arabia, Award Number (11-MED2065-10).

**Fig. 6 (abstract P17):**
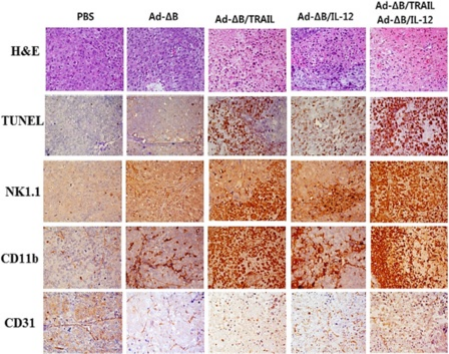
Representative photographs of histological, immunohistochemical and TUNEL analyses of the tumor tissues. H&E: hematoxylin and eosin staining for histopathology; TUNEL: staining of apoptotic cells in tumor tissue; NK1.1: to detect the infiltrated natural killer (NK) cells, the tumor tissues were treated with purified anti-mouse NK1.1 monoclonal antibody; CD11b: to detect the recruited antigen-presenting cells (dendritic cells and macrophage), the tumor tissues were treated with purified anti-mouse CD11b monoclonal antibody; and CD31: to detect tumor microvessels endothelial cells and vascular density, the tumor tissues were treated with anti-CD31 antibody. Brown staining indicates positive staining cells.

## P18 Therapy with paricalcitol attenuates tumor growth and augments tumoricidal and anti-oncogenic effects of 5-fluorouracil on animal model of colon cancer

### Adel Galal El-Shemi^1,2^, Bassem Refaat^1^, Osama Kensara^3^, and Amr Abdelfattah^1^

#### ^1^Department of Laboratory Medicine, Faculty of Applied Medical Sciences, Umm Al-Qura University, PO Box 7607, Holy Makkah, Saudi Arabia; ^2^Department of Pharmacology, Faculty of Medicine, Assiut University, Assiut, Egypt; ^3^Department of Clinical Nutrition, Faculty of Applied Medical Sciences, Umm Al-Qura University, PO Box 7607, Holy Makkah, Saudi Arabia

##### **Correspondence:** Adel Galal El-Shemi (dr_adel_elshemy2006@yahoo.com) – Department of Pharmacology, Faculty of Medicine, Assiut University, Assiut, Egypt

**Background**

The limited efficacy and safety of the current therapies of human colorectal carcinoma (CRC) represent a major obstacle. Thus, development of more effective treatment option is a paramount medical demand. In this regard, Paricalcitol (PCAL), a new vitamin D analogue with less calcemic side effects than vitamin D, has been recently identified as a pluripotent anticancer agent. Therefore, the current study was designed to investigate the therapeutic effect of Pcal against CRC and determine its underlying mechanism.

**Materials and methods**

CRC was developed in rats by using azoxymethane (AOM) model. Five groups of male Wistar rats were assigned as follow: normal controls, AOM alone, AOM-treated with 5–Fluorouracil (5-FU) which is the standard chemotherapeutic agent in the treatment of human CRC, AOM-treated with PCAL, and AOM-treated with PCAL + 5-FU. All groups were examined at week 15 after AOM injection, and their colons were and assessed at gross, histopathological, immunohistochemical, and molecular levels.

**Results**

**Fig. 7 (abstract P18):**
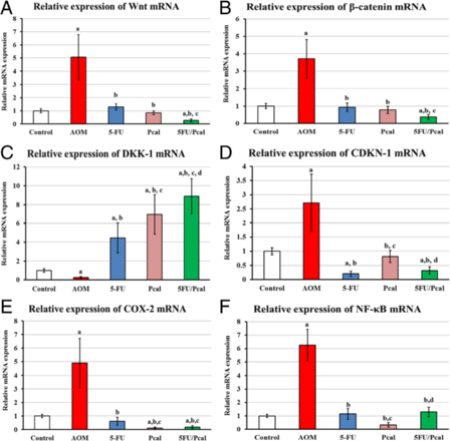
Paricalcitol (Pcal) and 5-Flurouracil (5-FU) had cooperated together to repress the expression of pro-cancerous genes of Wnt, β-catenin, CDNK-1A, NF-kB, COX-2, and to upregulate the expression of anti-tumorigenesis gene of DKK-1 compared with their monotherapies.

Therapy with PCAL not only reduced the growth of CRC but also augmented the tumoricidal effect of 5-FU, and they were significantly co-operated in inhibition of the colonic expression of well-known pro-oncogenic, angiogenesis- and metastasis inducing genes and molecules; such as Wnt/*β*-catenin signalling pathway, CDNK-1A, NF-*k*B, iNOS, Smads, HSP90, COX-2, Caspase-3, TGF-β1, and VEGF, that collectively have crucial roles in CRC development, promotion, invasion and metastasis. As shown in Fig. 7 Pcal and 5-FU had cooperated together to more repress the expression of pro-cancerous Wnt (Fig. 7a), β-catenin (Fig. 7b), CDNK-1A (Fig. 7d), COX-2 (Fig. 7e) NF-Kb (Fig. 7f); and to upregulate the expression of anti-tumorigenesis DKK-1 (Fig. 7c) compared with their monotherapies.

**Conclusions**

This study suggests that therapy with PCAL not only inhibit CRC development and promotion, but also has beneficial synergistic tumoricidal effect with 5-FU, and thereby could act as a potent additive anticancer agent for treatment of human colon cancer.

## P19 The effects of *Rubus idaeus* extract on normal human lymphocytes and cancer cell line

### Batol Imran Dheeb^1^, Mohammed M. F. Al-Halbosiy ^2^, Rghad Kadhim Al lihabi ^2^, Basim Mohammed Khashman^3^

#### ^1^Biology Department, College of Education, Iraqia University, Baghdad, Iraq; ^2^Biotechnology Research Center, Al- Nahrain University, Baghdad, Iraq; ^3^College of dentistry, Baghdad University, Baghdad, Iraq.

##### **Correspondence:** Batol Imran Dheeb (batoolomran@yahoo.com) – Biology Department, College of Education, Iraqia University, Baghdad, Iraq

**Background**

*Rubus idaeus* bioactive consider as cancer prevention according to Stoner *et al* [1]. Researches show berry bioactive protect against oxidative DNA damage), also a first line of defense against the multistage process of carcinogenesis. [2]. Active compounds play a major role in the induction of apoptosis (1). The aim of this study was to determine the cytotoxic activity of *Rubus idaeus* extract towards human lymphocyte and cancerous cell line also employed the pathway by which extract work and determine the cytokine level and CD markers in lymphocyte and cancerous cultured cell**.**

**Materials and methods**

Determination of Cytotoxicity effect of *Rubus idaeus* extract according to selected parameters including MTT assay [3] and *In vitro* Immunomodulation Determination.

**Results**

Extracted components from *Rubus idaeus* showed cytotoxic effects on the primary cell culture of normal hepatic cells (WRL-68), and cancer hepatic cell lines (HepG-2) at 120 μg/ml concentration. The most significant reduction (p ≤ 0.05) in cell viable count was at the concentration of 100 μg/ml which appears to cause induction of cell death via mitochondrial pathway for HepG-2 cell line after 24 hours’ exposure. The extract suppresses lymphocytes proliferation and caused increase in IL-2 and IL-4 level estimated by ELISA at concentrations of 250 and 500 μg/ml, while opposite results were shown after 4 hours of exposure.

**Conclusions**

*Rubus idaeus* extract having cytotoxic effects on HepG-2 and normal hepatic cells WRL68 line in a dose dependent manner.

**References**

1. Stoner GD, Wang LS, Casto BC (2008). **Laboratory and Clinical Studies of Cancer Chemoprevention by Antioxidants in Berries**. *Carcinogenesis*. **29**, 1665 – 1674.

2. García-Lafuente A, Guillamón E, Villares A, Rostagno M, Martínez J (2009). **Flavonoids as Anti-inflammatory Agents: Implications in Cancer and Cardiovascular Disease**. *Inflammatory Research*. **58**, 537 – 552.

3. Freshney, R.I. (2012). **Culture of Animal Cell. Sixth Edition**. Wily-Liss, New York.

## P20 Etanercept, a TNF-alpha inhibitor, alleviates mechanical hypersensitivity and spontaneous pain in a rat model of chemotherapy-induced neuropathic pain

### Djouhri, Laiche^1^, Chaudhary Adeel ^2^, Nedjadi, Taoufik^3^

#### ^1^Department of Physiology, College of Medicine, King Saud University, Riyadh, KSA; ^2^Centre for Excellence in Genomic Medicine Research, King Abdulaziz University, Jeddah, KSA; ^3^King Fahd Medical Research Centre, King Abdulaziz University, Jeddah, KSA.

##### **Correspondence:** Nedjadi, Taoufik (tnedjadi@kau.edu.sa) – King Fahd Medical Research Centre, King Abdulaziz University, Jeddah, KSA.

**Background**

Cancer is a major health problem and a leading cause of death worldwide. Many cancer patients receiving chemotherapy are unable to complete full or optimal treatment schedules because many potentially curative cancer chemotherapeutic agents cause severe toxic damage to the peripheral nervous system. This neurotoxicity is often accompanied by chronic peripheral neuropathic pain (NP). This chemotherapy-induced NP (CINP) is extremely debilitating and severely affects the life quality of 10 % to 100 % of cancer patients and survivors (depending on the dose and drug type). The underlying mechanisms of CINP, which is resistant to currently available drugs, are poorly understood, but pro-inflammatory cytokines may play a role. The aim of the current study was to examine the hypothesis that the pro-inflammatory TNF-α is involved in the pathophysiology of CINP, and that its blockade would alleviate pain hypersensitivity in an *in vivo* rat model of CINP.

**Materials and methods**

To induce CINP, 18 male Sprague Dawley rats were injected with the anti-cancer drug, Paclitaxel (2 mg/kg, i.p.) on four alternate days. All the rats were assessed for behavioral signs of mechanical and heat hypersensitivity, and spontaneous pain 4 weeks after treatment. To examine the effects of blocking TNF-α on these pain behaviours, two groups of CINP rats were used: one group (n = 12) was treated with the anti-TNF-α, etanercept (6 mg/kg, i.p.) and the other with vehicle (n = 12).

**Results**

All paclitaxel treated rats showed significant decreases (P < 0.001) in paw withdrawal threshold to a mechanical stimulus, but not in paw withdrawal latency to a noxious heat stimulus 4 weeks post treatment indicating development of mechanical hypersensitivity (allodynia), but not heat hypersensitivity (hyperalgesia). The rats also exhibited significant spontaneous foot lifting, a behavioural sign of spontaneous pain (see Djouhri et al., 2006; 2012). Interestingly our data also show that, compared with vehicle, etanercept significantly reduced mechanical hypersensitivity and spontaneous pain at both 24 h (P < 0.01) and 48-h (P < 0.01) post-drug treatment.

**Conclusions**

The findings suggest that TNF-α is involved in the pathophysiology of CINP, and that strategies that target TNF-α inhibition may be effective in treating CINP.

**Acknowledgements**

This work was financially supported by King Abdulaziz City for Science and Technology (KACST) under research grant no. (12-MED3091-03).

## P21 Sleeping beauty mutagenesis system identified genes and neuronal transcription factor network involved in pediatric solid tumour (medulloblastoma)

### Hani Al-Afghani^1,2^, Maria Łastowska^1^, Haya H Al-Balool^1^, Harsh Sheth^1^, Emma Mercer^1^, Jonathan M Coxhead^1^, Chris PF Redfern^1^, Heiko Peters^1^, Alastair D Burt^1^, Mauro Santibanez-Koref^1^, Chris M Bacon^1^, Louis Chesler^1^, Alistair G Rust^1^, David J Adams^1^, Daniel Williamson^1^, Steven C Clifford^1^, Michael S Jackson^1^

#### ^1^Institute of Genetic Medicine, Newcastle University, Newcastle upon Tyne, UK; ^2^Security Forces Hospital, General Directorate of Medical Services, Ministry of Interior, Makkah, KSA

##### **Correspondence:** Hani Al-Afghani (hani940@hotmail.com) – Security Forces Hospital, General Directorate of Medical Services, Ministry of Interior, Makkah, KSA.

**Background**

Medulloblastoma (MB) is a paediatric tumour of the cerebellum which is responsible for 15-20 % of all childhood brain tumours. Mortality due to this disease is high (~40 %) and successful treatment is associated with significant neurological and cognitive consequences, making new therapies desirable. Disruption of the Sonic Hedgehog (SHH) signaling pathway, including mutations in PTCH1, define a major subset of human MB. Mice heterozygous for the Ptch1 ortholog develop MBs at low frequency.

**Materials and methods**

To identity genes that co-operate with Ptch1 in MB development, we have performed a Sleeping Beauty insertional mutagenesis screen in this murine model. To identify the genes responsible for this enhanced tumour formation we have used Splinkerette-PCR and 454-FLX sequencing to define SB insertion sites within 40 tumours. Monte Carlo and Kernel Convolution statistical methods were applied on these data to identify statistically significant candidate MB genes defined by common insertion sites (CISs). Sophisticated bioinformatic analysis were conducted in human gene expression datasets to identify a plausible neuronal network. Illumina beads microarray was carried out on mutagenised mice primary tumour samples.

**Results**

We find that mutagenesis significantly increases the frequency of MB formation in Ptch heterozygote mice from ~3 % to ~25 % after 8 months (p < 0.001). Statistical analysis identified 18 (CISs). Many of these are gene known to be involved in neuronal development and cell fate determination. Subsequent ARACNe network analysis has established that seven (CISs) lie within a single network (with Myt1l, was highly networked gene), which is enriched for both neuronal genes and transcription factors, and includes genes known to interact with the SHH pathway. Furthermore, the disrupted network genes work synergistically to upregulate Igf2 (involved in MB formation).

**Conclusions**

Functional analyses of this network should both improve our understanding of how this tumour develops, and define potential targets for therapeutic and diagnostic intervention.

## P22 Involvement of interleukin-1 in vitiligo pathogenesis

### Mala Singh^1^, Mohmmad Shoab Mansuri^1^, Shahnawaz D. Jadeja^1^, Hima Patel^1^, Yogesh S. Marfatia^2^, Rasheedunnisa Begum^1^

#### ^1^Department of Biochemistry, Faculty of Science, The M.S. University of Baroda, Vadodara- 390 002, Gujarat, India; ^2^Department of Skin andVD, Sir Sayajirao Gaikwad Medical College, The Maharaja Sayajirao University of Baroda, Vadodara, Gujarat, India.

##### **Correspondence:** Rasheedunnisa Begum (rasheedunnisab@yahoo.co.in) – Department of Biochemistry, Faculty of Science, The M.S. University of Baroda, Vadodara- 390 002, Gujarat, India

**Background**

Vitiligo is a hypomelanotic autoimmune skin disorder. High serum and transcript levels of IL1 have been reported in vitiligo patients [1, 2]. *IL1RN* intron 2 VNTR (rs1794068) has been found to be associated with autoimmune disorders including vitiligo [3]. We have explored the role of Interleukin (IL)-1 in vitiligo by monitoring the expression of *IL1A, IL1B, IL1Receptor1 (R1)* and *IL1 Receptor Antagonist (RN)* in skin samples, effect of IL1-α on melanocyte viability, IL1R1 membrane expression and genotyping of *IL1RN* VNTR in Gujarat population.

**Materials and methods**

Twelve skin *biopsies* from vitiligo patient’s lesional, non-lesional skin and controls were obtained. RNA was isolated and relative gene expression was performed using Real-time-PCR. Genomic DNA was extracted from whole blood of 226 vitiligo patients and controls for *IL1RN* VNTR genotyping. PrimaryNormal Human Melanocytes (NHM) were isolated and cultured from human skin, cell viability was monitored by MTT assay. Membrane expression of IL1R1was monitored using Flow-cytometry.

**Results**

**Fig. 8 (abstract P22):**
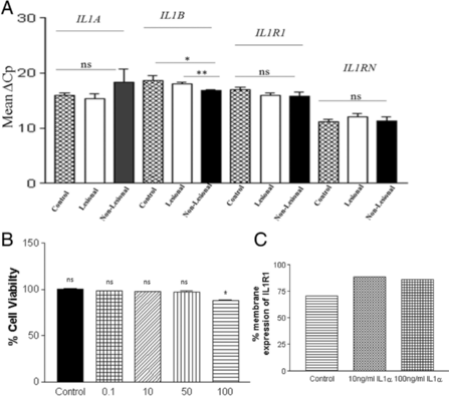
**a** *IL1A, IL1B, IL1R1 and IL1RN* transcript levels in skin of vitiligo patients (n = 12) and controls (n = 12). Significant increase of *IL1B* transcript levels in non lesional skin compared to lesional skin of vitiligo patients and control skin. (*p* = 0.0021 and *p* = 0.0290 respectively) was observed. However, no significant difference in the transcript levels of *IL1A, IL1R1 and IL1RN* was observed. **b** MTT cell viability assay: IL1-α (100 ng/ml) showed significant decrease in cell viability of NHM at 48 hrs (n = 3, *p < 0.0210). However, at lower concentrations (0.1-50 ng/ml) IL1-α did not show any significant change in the % cell viability. **c** The NHM were treated with 10 ng/ml IL1α and 100 ng/ml IL1α and subsequently IL1R1 levels were measured by Flow cytometry after 48 hrs of treatment. Results showed significant increase (~22 %) in the membrane expression of IL1R1 upon exogenous IL1-α stimulation to NHM

We investigated the expression of *IL1A, IL1B, IL1R* and *IL1RN* in control and vitiliginous human skin, and found unaltered levels of *IL1A, IL1R1* and *IL1RN* (*p* = 0.6000, *p* = 0.8186, *p* = 0.2147 respectively). Interestingly, significant increase in *IL1B* levels was seen in non- lesional compared to lesional vitiliginous skin (*p* = 0.0021), suggesting its important role in disease progression (Fig. 8a). *In vitro* studies were performed on NHM to monitor the dose dependent effect of IL1-α on melanocyte death. IL1-α (100 ng/ml) showed ~12 % melanocyte death upon 48 hrs. treatment (Fig. 8b). Further, transcript levels as well as membrane expression of IL1R1 in NHM upon IL1-α treatment was studied.1.24-fold increase in *IL1R1* and ~22 % increase in membrane expression of IL1R1 as observed (Fig. 8c). Additionally, genotyping of *IL1RN* VNTR in 226 patients and controls was carried out and no significant difference was found in genotype and allele frequency (Table 8).

**Conclusions**

Significant increase of *IL1B* in non-lesional skin indicates its important role in vitiligo progression. IL1-α decreases the NHM viability via upregulation of IL1R1, suggesting the important role of IL1 in immune homeostasis and melanocyte biology. Lack of genetic association of *IL1RN*VNTR polymorphism suggests dysregulation of IL-1 signaling may not be due to *IL-1RN* studied polymorphism i.e., rs1794068.

**Acknowledgements**

This work was supported by grant to RB (BMS/Adhoc/122/11-2012) Indian Council of Medical Research, New Delhi, India.

**References**

1. Laddha NC, Dwivedi M, Mansuri MS, Singh M, Patel HH, Agarwal N, Shah AM, **Begum R: Association of Neuropeptide Y (NPY), Interleukin-1β (IL1B) Genetic Variants and Correlation of IL1B Transcript Levels with Vitiligo Susceptibility.***PLoS ONE 2014,***9**:e107020.

2. Birol A, Kisa U, Kurtipek GS, Kara F, Kocak M, Erkek E, Caglayan O:**Increased tumor necrosis factor alpha (TNF-alpha) and interleukin 1 alpha (IL1-alpha) levels in the lesional skin of patients with nonsegmental vitiligo.***Int J Dermatol.* 2006, **8**:992-3.

3. Pehlivan S, Ozkinay F, Alper S, Onay H, Yuksel E, Pehlivan M, Ozkinay C**: Association between IL4 (-590), ACE (I)/(D), CCR5 (Delta32), CTLA4 (+49) and IL1-RN (VNTR in intron 2)gene polymorphisms and vitiligo.***Eur J Dermatol.* 2009,**2**:126-8.

**Table 8 (abstract P22):** Distribution of Genotypes and Alleles for *IL1RN* intron 2 VNTR (rs1794068) in vitiligo patients and controls from Gujarat. ‘n’ represents number of Patients/Controls

SNP	Genotype or allele	Vitiligo Patients (Freq.)	Controls (Freq.)	*p* for Association	Odds ratio	CI (95%)
*IL1RN* intron 2 VNTR (rs1794068)	Genotype	n = 226	n = 226			
	(A 1/1)	85 (37.61)	93 (41.15)	R	1^a^	-
	(A 1/2)	89 (39.38)	84 (37.16)	0.4891^a^	0.8626^a^	0.5674 to 1.311^a^
	(A 2/2)	40 (17.69)	33 (14.6)	0.3109^a^	0.7540^a^	0.4364 to 1.303^a^
	(A 3/2)	3 (1.32)	4 (1.76)	0.7992^a^	1.219^a^	0.2650 to 5.605^a^
	(A 3/1)	8 (3.53)	9 (3.98)	0.9564^a^	1.028^a^	0.3794 to 2.787^a^
	(A 4/2)	1 (0.44)	00 (0.00)	0.2970^a^	0.3048^a^	0.01224 to 7.589^a^
	(A 1/4)	00 (0.00)	1 (0.44)	0.3403^a^	2.743^a^	0.1102 to 68.30^a^
	(A 5/2)	00 (0.00)	2 (0.88)	0.1786^a^	4.572^a^	0.2163 to 96.66^a^
	Allele					
		182(55.6)				
	A1	133(40.6)	187(57.3)	R^b^	1^b^	-
		11(3.36)				
	A2		123(37.7)	0.5177^b^	0.9001^b^	0.6542 to 1.238^b^
		1(0.30)				
	A3		13(3.98)	0.7404^b^	1.150^b^	0.5022 to 2.634^b^
		0(0)				
	A4		1(0.30)	0.9848^b^	0.9733^b^	0.06038 to 15.69^b^
	A5		2(0.61)	0.1641^b^	4.867^b^	0.2319 to 102.1^b^

‘R’ represents reference group

HWE refers to Hardy-Weinberg Equilibrium

CI refers to Confidence Interval

(P) refers to Patients and (C) refers to Controls

^a^Vitiligo Patients vs. Controls (genotype) using chi-squared test with 2 × 2 contingency table

^b^Vitiligo Patients vs. Controls (allele) using chi-squared test with 2 × 2 contingency table

A are different alleles of *IL1RN* intron 2 VNTR (rs1794068)

## P23 Cytogenetics abnormalities in 12,884 referred population for chromosomal analysis and the role of FISH in refining the diagnosis (cytogenetic experience 2004-2013)

### Amal M Mohamed^1^, Alaa K Kamel^1^, Nivin A Helmy^1^, Sayda A Hammad^1^, Hesham F Kayed^1^, Marwa I Shehab^1^, Assad El Gerzawy^1^, Maha M. Ead^1^, Ola M Ead^1^, Mona Mekkawy^1^, Innas Mazen^2^, Mona El-Ruby^2^

#### ^1^Human Cytogenetics Department, National Research Centre, Dokki, Egypt; ^2^Clinical Genetics Department, National Research Centre, Dokki, Egypt

##### Amal M Mohamed (amalmahmoud15@yahoo.com) – Human Cytogenetics Department, National Research Centre, Dokki, Egypt; ^2^Clinical Genetics Department, National Research Centre, Dokki, Egypt

**Background**

This study represents the frequency of chromosomal abnormalities in 12,844 patients referred to the Human Cytogenetics Department from the outpatient’s clinics of Clinical Genetics Department, National Research Centre. The aim of this study is to assess the frequency and types of chromosome abnormalities and identify the role of FISH in refine the clinical diagnosis and compare our findings to results reported in similar studies.

**Results**

According to the cause of referrals the patients were categorized into six groups, intellectual disability/multiple congenital abnormalities represented 31.4 % of all patients, disorders of sex development represent 24 %, genetic counseling for previous history of affected child 10 %, repeated abortions 10 %, premarital counseling 9.2 %, miscellaneous group like obesity, Congenital heart defects, limb anomalies, chromosome breakage syndrome, etc 15.4 %. Males represent 46.2 %, females represented 52.8 % and unidentified sex 1 %. The total rate of chromosomal abnormalities was 19.3 %. The highest anomaly rate was among Down syndrome. Autosomal anomalies represent 78.5 %, numerical anomalies were 50.5 %, and structure anomalies were 28 %. Sex chromosome anomalies were 21.5 %, numerical 15.7 %, and structural 5.8 %. FISH analysis was performed on 12 % of patients referred for cytogenetic analysis, it diagnosed patients with microdeletion syndromes, confirmed the sex chromosome anomalies in DSD and identified the break sites in chromosomal translocation and the nature of add chromosomal materials. Through the detection of microdeletion syndromes FISH raised the chromosomal abnormalities in this referred population by 1 %.

**Conclusions**

Cytogenetics and FISH could diagnose 20.3 % of referred population for cytogenetics, still a large number of these patients needs genetic diagnosis especially patients with ID. We recommend the application of multiple ligation probe amplification (MLPA) and array CGH which will help for more accurate genetic testing.

**Acknowledgements**

Authors would like to thank the National Research Centre, Egypt for supporting this work.

**References**

1. Wellesley D, Dolk H, Boyed PA, Greenlees R, Haeuusler M, Nelen V, Garne E, khoshnood B, Doray B, Rissmann A, Mullaney C, Calzolari E, Bakker M, Salvador J, Addor M, Draper E, Rankin J, Tucker D: **Rare chromosomal abnormalities, prevalence and prenatal diagnosis rates from population-based congenital anomaly registers in Europe.** European Journal of Human Genetics 2012, **20**:521-526.

2. Ghazaey S, Mirzaei F, Ahadian M, keifi F, Tootian S, Abbasadegan R. **Pattern of chromosomal aberrations in patients from north east Iran. CELL Journal** 2013, **3**: 258-265.

## P24 Analysis of binding properties of angiotensin-converting enzyme 2 through *in silico* method

### S. M. A. Shahid^1^, Qazi Mohammad Sajid Jamal^2^, J. M. Arif^1^, Mohtashim Lohani^3^

#### ^1^Department of Biochemistry, College of Medicine, University of Hail, Hail, Saudi Arabia; ^2^Department of Health Information Management, College of Applied Medical Sciences, Buraydah Colleges, Al Qassim, Saudi Arabia; ^3^Department of Biosciences, Faculty of Applied Science, Integral University, Lucknow, India.

##### **Correspondence:** S. M. A. Shahid (2007.sma@gmail.com) – Department of Biochemistry, College of Medicine, University of Hail, Hail, Saudi Arabia

**Background**

Angiotensin-converting enzyme 2 (ACE 2) belongs to the rennin-angiotensin system (RAS) and is a potential therapeutic target for the control of cardiovascular diseases and hypertension. Inhibitors of ACE2 are effective drugs for the treatment of cardiovascular diseases and associated pathophysiology. In this study, we have taken the human Angiotensin-Converting Enzyme 2 and their known inhibitors, to identify the catalytic site residues of ACE2. The predicted catalytic traid of ACE2 is ALA 354 - GLN 281 - LYS 511 – GLU 376 – THR 282. Our results would be more useful in designing and development new inhibitors of human ACE2 to control cardiovascular diseases and hypertension.

**Results**

We used a novel, rapid, and economical structure-based approach to predict the catalytic site of human Angiotensin-Converting Enzyme 2 (ACE2). We docked all 7 known inhibitors with ACE2 using AutoDock program and evaluated the binding compatibility with receptor based on docked energy (in kcal/mol). The docking tool generated 30 conformations for each docked inhibitor in approximately 25 minutes of CPU time. Based on docking energy it was predicted that the inhibitors Candesartan (-7.55 kcal/mol), losartan (-12.07 kcal/mol), Quinapril (-11.7 kcal/mol), Ramipril (-11.57 kcal/mol), Valsartan (-6.75 kcal/mol), Perindopril (-10.04 kcal/mol) and Enalapril (-10.36 kcal/mol) have good binding affinities towards the ACE2 and their Root mean square deviation from a reference.

**Conclusions**

The protein-ligand interaction plays a significant role in structure based drug designing and is extensively used to reduce cost and time in drug discovery. In this present work, we have taken 7 known inhibitors of ACE2 namely Candesartan, Losartan, Quinapril, Ramipril, Valsartan, Perindopril and Enalapril were taken for *in silico* prediction of catalytic site (ALA 354 - GLN 281 - LYS 511 – GLU 376 – THR 282) residues of the human Angiotensin-converting enzyme 2 (ACE2). Our reports can be used to design and develop new inhibitors with better binding affinities towards the ACE2 to recuperate cardiovascular disease.

**Acknowledgements**

The authors are thankful to the University of Hail for providing all the necessary facilities to carry out this work.

## P25 Relationship of genetics markers *cis* and *trans* to the β-S globin gene with fetal hemoglobin expression in Tunisian sickle cell patients

### Moumni Imen^1^, Chaouch Leila^1^, Ouragini Houyem^1^, Douzi Kais^1^, Chaouachi Dorra Mellouli Fethi^2^, Bejaoui Mohamed^2^ and Abbes Salem^1^

#### ^1^Laboratory of molecular and cellular Hematology. Pasteur Institute of Tunis, University of Tunis El-Manar, 74 PO Box. 1002 Belvedere, Tunis, Tunisia; ^2^Service d’Immuno-Hématologie pédiatrique, Centre National de Greffe de Moelle Osseuse, Tunis, Tunisia

##### **Correspondence:** Moumni Imen (moumniimen@yahoo.fr) – Laboratory of molecular and cellular Hematology. Pasteur Institute of Tunis, University of Tunis El-Manar, 74 PO Box. 1002 Belvedere, Tunis, Tunisia

**Background**

Sickle cell anemia is the first monogenic disease described in human, and it became the paradigm for a disease traceable to a single mutation in a single gene. Spectacular results are certainly being obtained useful for understanding the phenotypic variability of the disease. However, Fetal hemoglobin (HbF) plays a dominant role in ameliorating morbidity and mortality of hemoglobinopathies. The aim of this study is to evaluate the effect of polymorphic markers located *in cis* and *trans* of β^S^-globin gene on the variation of HbF expression among Tunisian sickle cell patients. After formal consent, we performed the Haplotype analysis of the β-globin gene cluster by (PCR-RFLP), the framework polymorphism was established by PCR-sequencing, four independent regions of interest were investigated: the 5′ region of β-LCR-HS2 site, the intervening sequence II (IVSII) region of the two fetal genes (Gγ and Aγ) and the 5′ region of β-Globin gene. In *trans* of the βS-globin gene we studied two polymorphisms rs1005589 and rs11095629 on neuronal membrane glycoprotein gene (GPM6B) in chromosome X by SSP-PCR.

**Results**

The Correlation of these various Haplotypes and SNPs with HbF expression was studied. Our data showed that among the various polymorphic markers analyzed in cis of β^S^-globin gene, only the sequence (AT)xN12(AT)y in LCR HS2 region was significantly associated (p < 0.05) with increased HbF levels, suggesting that the β-globin gene cluster exerts a significant effect on HbF in sickle cell patients. In GPM6B gene, our results indicate that the rs11095629 and rs1005589 are associated with HbF level variation.

**Conclusions**

This study suggests that different genes might modulate the rate of HbF in sickle cell anemia, which can improve understanding of the physiopathology of the disease and aid to increase our ability to predict clinical severity.

**Acknowledgements**

This work has been supported by «la direction de la recherche scientifique, ministere de l’enseignement supérieur et de la recherche scientifique de Tunisie », Laboratory of Molecular and Cellular Hematology, Pasteur Institute of Tunis: LR 11 IPT 07».

## P26 Analysis of estrogen receptor alpha gene polymorphisms in breast cancer: link to genetic predisposition in Sudanese women

### Areeg Faggad^1,2^, Amanuel T Gebreslasie^2,3^, Hani Y Zaki^2^, Badreldin E Abdalla^2,4^

#### ^1^Department of Molecular Biology, National Cancer Institute (NCI-UG), University of Gezira, Wad Madani, Sudan; ^2^Department of Biochemistry and Nutrition, Faculty of Medicine, University of Gezira, Wad Madani, Sudan; ^3^Department of Biochemistry, Orotta School of Medicine and Dentistry, Eritrea; ^4^Department of Biochemistry, Faculty of Science, King Abdulaziz University, Jeddah, Saudi Arabia

##### **Correspondence:** Areeg Faggad (areegfaggad@hotmail.com) – Department of Biochemistry and Nutrition, Faculty of Medicine, University of Gezira, Wad Madani, Sudan

**Background**

Breast cancer (BC) is the most frequently diagnosed cancer and the leading cause of cancer death among women worldwide [1]. The incidence rates are increasing alarmingly among Sudanese women. Estrogen receptor alpha, encoded by *ESR1* gene, is a ligand-activated transcription factor that mediates estrogen action in target tissues. Estrogen is essential in breast tissue proliferation and differentiation. Estrogen exposure is a central risk factor in the development of BC [2]; therefore, genetic variants in *ESR1* are likely to affect BC susceptibility. Most genetic associations on BC have arisen from studies investigating European and American patients. However, possibility of specific changes among cancer patients of different ethnic groups remains unexplored. The aim of this study was to evaluate the association of two single nucleotide polymorphisms (SNPs) in *ESR1* with BC among Sudanese women.

**Subjects and methods**

This case-control study included 71 BC women diagnosed at National Cancer Institute (NCI-UG), University of Gezira, Sudan from 2012 to 2014 (patient group); and 73 women having no evidence of personal or family history of BC were recruited as a control group. DNA was extracted from peripheral blood. Genotyping of the *ESR1* polymorphisms were performed using polymerase chain reaction-restriction fragment length polymorphism (PCR-RFLP). Amplification products were digested by BsmAI and Hpy188III endonucleases for rs3020314 and rs1643821 respectively, the DNA fragments were electrophoresed and the resulting bands were visualized using automated gel documentation system. Information on demographic data, personal and family medical history, in addition to reproductive history was obtained. Anthropometric measurements and clinicopathological characteristics were assessed.

**Results**

The findings showed significant differences in genotype frequencies between cases and controls. For rs3020314, women carrying the heterozygous genotype CT had a significantly increased risk of BC compared to controls (OR: 2.67; 95 % CI: 1.19-6.01; p = 0.014). Conversely, for rs1514348 women with the heterozygous genotype CT showed a significantly decreased BC risk (OR: 0.41, 95 % CI: 0.19-0.89; p = 0.046). There were no differences in allele frequencies between the two groups. For rs3020314, cases with BMI > = 25 kg/m^2^ carrying CT genotype had 7 times BC risk than controls, likewise postmenopausal BC patients with CT genotype had 6 times BC risk than controls. No association was found between genotype frequencies of either SNP with clinicopathologic features.

**Conclusions**

Our data suggest that the *ESR1*polymorphism rs3020314 might contribute to increased BC risk, and rs1643821 to decreased risk in Sudanese women. However, these findings need to be further tested in a larger number of Sudanese women in a population-based approach.

**References**

1. Ferlay J, Soerjomataram I, Ervik M, Dikshit R, Eser S, Mathers C, Rebelo M, Parkin DM, Forman D, Bray, F. **GLOBOCAN 2012 v1.0, Cancer Incidence and Mortality Worldwide: IARC CancerBase No. 11** [Internet]. Lyon, France: International Agency for Research on Cancer; 2013. Available from: http://globocan.iarc.fr, accessed 14 Oct 2015.

2. Dumitrescu RG, Cotarla I. **Understanding breast cancer risk - where do we stand in 2005?***J Cell Mol Med* 2005; **9**(1): 208-221.

## P27 KCNQI gene polymorphism and its association with CVD and T2DM in the Saudi population

### Maha S AlShammari, Rhaya Al-Ali, Nader Al-Balawi , Mansour Al-Enazi, Ali Al-Muraikhi, Fadi Busaleh, Ali Al-Sahwan, Francis Borgio, Abdulazeez Sayyed, Amein Al-Ali, Sadananda Acharya

#### College of Medicine, Institute for Research and Medical Consultations, University of Dammam, Dammam, Saudi Arabia

##### **Correspondence:** Maha S AlShammari (Alshammari.maha.s@gmail.com) – College of Medicine, Institute for Research and Medical Consultations, University of Dammam, Dammam, Saudi Arabia

**Background**

Genome Wide Association studies have identified several loci associated with an increased risk of developing CVD and T2DM, this was confirmed by replication studies. Polymorphisms within KCNQ1gene are consistently associated with T2DM in a number of populations. Recent reports indicated that both T2DM and CVD are increasing in the Saudi population, with T2DM reaching alarming levels in the Eastern Province. This study was undertaken to shed light on the possible association of three polymorphisms (rs2237892, rs151290 and rs2237895) with T2DM and/or CVD in this population.

**Subjects and methods**

Patients clinically diagnosed with either T2DM (320 patients), CVD (250 patients) or both (60 patients) and 516 healthy controls were included in the study. Genotyping was performed by TaqMan assay run on a real time PCR thermocycler.

**Results**

A statistically significant association was found for SNPs rs151290 (OR = 1.63; p = <0.00001) and rs2237895 (OR = 1.70; p = <0.00001) with CVD. Moreover, SNPs rs151290 (OR = 2.20; p = 0.0029) and rs2237895 (OR = 1.56; p = 0.031514) showed a strong association in patients with both T2DM and CVD. However, none of the SDNPs tested showed any significant association with T2DM. Haploview analysis showed that CCC (rs151290 “C”; rs2237892 “C”; rs2237895 “C”) and ACC (rs151290 “A”; rs2237892 “C”; rs2237895 “C”) haplotypes are the most significant risk (p < 0.00001) allele combinations for CVD, while CCA (rs151290 “C”; rs2237892 “C”; rs2237895 “A”) and ACA (rs151290 “A”; rs2237892 “C”; rs2237895 “A”) are co-morbidity risk haplotypes for T2DM and CVD.

**Conclusions**

KCNQ1 polymorphism at SNPs rs151290 and rs2237895 is strongly associated with CVD in this population, but reflected no association with T2DM.

## P28 Clinical, neuroimaging and cytogenetic study of a patient with microcephaly capillary malformation syndrome

### Maha S. Zaki^1^, Hala T. El-Bassyouni^1^, Marwa I. Shehab^2^

#### ^1^Clinical Genetics Department, National Research Centre, Cairo, 12622, Egypt; ^2^Cytogenetics Department, National Research Centre, Cairo, 12622, Egypt.

##### **Correspondence:** Hala T. El-Bassyouni (halabassyouni@yahoo.com) – Clinical Genetics Department, National Research Centre, Cairo, 12622, Egypt

**Background**

\Microcephaly-capillary malformation syndrome (MICCAP syndrome) is a newly recognized congenital neurocutaneous central nervous system disorder. To date the diagnosis has been confirmed in 14 affected individuals.

**Materials and methods**

Meticulous clinical examination, brain MRI, echocardiogram, abdominal ultrasound, ophthalmological examination, electroencephalogram (EEG), thyroid profile, intelligent quotient, karyotyping and array-CGH were done.

**Results**

We report a 5 years old girl of a non-consanguineous marriage with microcephaly, early onset seizures that started at 7 months of age, craniofacial dysmorphism, recurrent infection and profound intellectual disability. Delayed mile stones, craniofacial dysmorphism included narrow forehead, anterior recession of hair line, scanty eye brows, depressed nasal bridge, squint, tented upper lip, and protruded tongue. Limited extension of right wrist, brittle dystrophic nails, soft tissue syndactly of fingers and areas of cutaneous vascular malformation. Neurologically, she had hypertonia and hyperreflexia. The anthropometric measurements were weight 9 Kg (-3.5 SD), head circumference 39.5 cm (-8.8 SD) and height 90.5 cm (-3.7 SD). Thyroid profile showed hypothyroidism and Echo heart displayed patent foramen oval, right ventricular dysplasia. DEXA revealed osteopenia at the spine and femur. The Intelligent Quotient (IQ) at the age of 5 years based on the Wechsler Intelligence Scale was 56 (profound intellectual disability). The MRI brain showed severe microcephaly with marked cerebral atrophic changes, incomplete demyelination, vascular malformation and hemorrhage of the brain. Dental examination revealed high arched palate, delayed teeth eruption, abnormal small teeth, premature shedding of upper incisors and high caries index. Karyotype revealed normal female karyotype 46,XX and array-comparative genomic hybridization analysis (array CGH) was normal.

**Conclusions**

To our knowledge this work presents the first patient of Microcephaly capillary malformation syndrome reported in Egypt. Our study displays previously unreported findings of dental abnormalities and osteopenia.

**Consent to publish**

Written informed consent for publication of the clinical details was obtained from the parents of the patient. A copy of the consent form is available for review by the Editor of *BMC Genomics*.

## P29 Altered expression of CD200R1 on dendritic cells of patients with inflammatory bowel diseases: *in silico* investigations and clinical evaluations

### Mohammed F. Elshal^1^, Kaleemuddin M.^1^, Alia M. Aldahlawi^2^, Omar Saadah^3^, J. Philip McCoy^4^

#### ^1^Biochemistry Department, Faculty of Sciences, King Abdulaziz University, Jeddah, Saudi Arabia; ^2^Biological Sciences Department - Faculty of Sciences, King Abdulaziz University, Jeddah, Saudi Arabia; ^3^Department of Pediatrics, Faculty of Medicine, King Abdulaziz University, Jeddah, Saudi Arabia; ^4^Flow Cytometry Core Facility, National Heart, Lung, and Blood Institute, National Institutes of Health, Bethesda, MD, USA

##### **Correspondence:** Mohammed F. Elshal (melshal@kau.edu.sa) – Biochemistry Department, Faculty of Sciences, King Abdulaziz University, Jeddah, Saudi Arabia

**Background**

Inflammatory bowel diseases (IBD) are chronic disease that are thought to occur due to loss of tolerance toward commensal flora and food antigens in the gut. The CD200 receptor (CD200R) cell surface protein is an inhibitory receptor mainly expressed on myeloid cells, whose known functions are immunosuppression and modulation of inflammation [2]. We aimed to analyze the structural deviation of clinically significant mutant forms of CD200R by computational modeling and to investigate the expression of CD200R1 by dendritic cells (DCs) using flow cytometry to determine whether there are any abnormalities in their expression at the cellular level.

**Materials and methods**

The *In-silico* analysis of clinically potential missense mutations of human CD200R1 gene was performed by algorithms based on sequence and structure homology (SIFT, PolyPhen and ConSurf). Molecular modeling and secondary structural analysis was done to confirm their impact on the stability and secondary properties of wild-type and mutated CD200R1 protein. For *in vivo* validation, 37 IBD patients (14 UC and 23 CD) were included. Fourteen age-matched healthy volunteers (5 males and 9 females) served as a healthy control group (HC). Peripheral blood mononuclear cells were collected and analyzed for expression of CD200R1 on DCs multi-parametric flow cytometry as described elsewhere [4]. The study protocol was approved by the Ethics Committee of the KAUH.

**Results**

**Fig. 9 (abstract P29):**
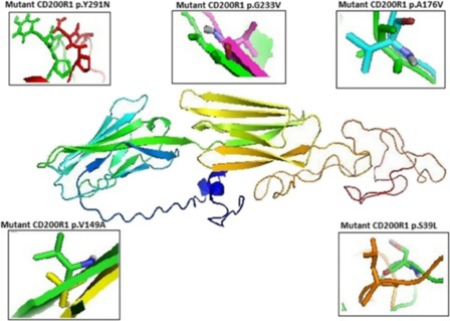
The central figure demonstrates the *ab initio* 3D model of CD200R1 developed from 1 Tasser server. Surrounding figures show the structural deviation between wild-type model (green coloures) and mutant type model (non-green coloured) of selected mutations of CD200R1 protein which is related to IBD. (Visualized by PyMol-Molecular graphic system)

**Fig. 10 (abstract 29):**
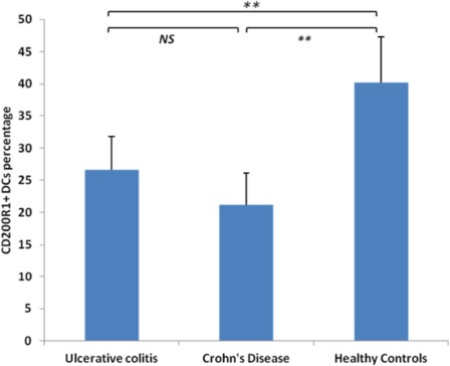
Boxplot shows the frequency of CD200R+ on DCs of patients with Ulcerative colitis (UC), Crohn’s Disease (CD) as compared with (healthy Controls) HC: NS: not significant; *P < 0.05; **P < 0.001

By using *in-silico* algorithms, we were able to identify that the deleterious mutations corresponding to p.Y291N, p.G223V, p.A176V, p.V149A and p.S39L have potential role in structural and functional deviations of CD200R1 activity (RMSD ≥ 2 Å) (Fig. 9). Multi-parametric flow-cytometry results revealed significant decrease (P < 0.001) in CD200R expressing DCs of IBD patients when compared with that of HC (Fig. 10).

**Conclusions**

In-silico finding suggests that mutations in CD200R1 influence IBD rate, which we confirmed this hypothesis by demonstrating that CD200R1 expressing DCs are significantly lower in IBD patients compared with healthy controls. Collectively, these data present evidence for dysregulated expression of the inhibitory molecule CD200R1 on DCs that may contribute to the immunologic abnormalities occurring in this disease and provide insight into the pathophysiology of IBD. Further functional studies are mandatory to explore the molecular mechanism underlying the down-regulation of CD200R in IBD.

**Acknowledgements**

The authors would like to express their sincere appreciation to the Deanship of Scientific Research at the King Abdulaziz University, Jeddah, Saudi Arabia for funding this Research Group project no. RG/32/02.

**References**

1. J.E. Lennard-Jones, **Classification of inflammatory bowel disease, Scandinavian journal of gastroenterology**. *Supplement*, **170** (1989) 2-6; discussion 16-19.

2. D. Holmannova, M. Kolackova, K. Kondelkova, P. Kunes, J. Krejsek, C. Andrys**, CD200/CD200R paired potent inhibitory molecules regulating immune and inflammatory responses; Part I: CD200/CD200R structure, activation, and function**, *Acta medica*, **55** (2012) 12-17.

3. A. Levine, A. Griffiths, J. Markowitz, D.C. Wilson, D. Turner, R.K. Russell, J. Fell, F.M. Ruemmele, T. Walters, M. Sherlock, **Pediatric modification of the Montreal classification for inflammatory bowel disease: the Paris classification,***Inflammatory bowel diseases*, **17** (2011) 1314-1321.

4. Slukvin, II, M.A. Vodyanik, J.A. Thomson, M.E. Gumenyuk, K.D. Choi, **Directed differentiation of human embryonic stem cells into functional dendritic cells through the myeloid pathway,***Journal of immunology*, **176** (2006) 2924-2932.

## P30 Development of real time PCR diagnostic protocol specific for the Saudi Arabian H1N1 viral strains

### Adel E El-Tarras^1^, Nabil S Awad^2,3^, Abdulla A Alharthi^1^, Mohamed M M Ibrahim^3^

#### ^1^Biotechnology and Genetic Engineering Research Unit, Deanship of Scientific Research, Taif University, Taif, KSA; ^2^Department of Genetics, Faculty of Agriculture and Natural Resources, Aswan University, Aswan, Egypt; ^3^College of Biotechnology, Misr University for Science and Technology, Sixth of October City, Egypt

##### **Correspondence:** Mohamed M M Ibrahim (mohamed.ibrahim@must.edu.eg) – College of Biotechnology, Misr University for Science and Technology, Sixth of October City, Egypt

**Background**

Periodically, complete novel antigenic subtypes of influenza viruses have been introduced in human population, causing large-scale global outbreaks with high death tolls. As a consequence of all this, pandemic preparedness has become an important issue worldwide and specially for Kingdom of Saudi Arabia. Theses Pandemic plans should include proper approaches that allow early recognition of novel influenza viruses infecting humans in the future.

**Results**

In silico PCr analysis of the designed oligonucleotide primers showed a strong matching with the corresponding nucleotide sequences of the KSA H1N1 viral strains. Moreover, real time PCR data generated from this study produced a variable range of Cycle threshold (C.t.) values of 16.61 – 31.7. Furthermore, sensitivity test of the optimized real time PCR protocol showed that recommended protocol is efficient and specific to the H1N1 KSA strains, especially the oligonucleotide primers H1N1 KSA F2R2 which produced an earlier C.t. value of 16.61.

**Conclusions**

Results of this study confirm the importance of design specific diagnostic protocols against local infectious agents in order to increase the accuracy and efficiency of the detection process and, hence, increases the chances to control the epidemic spread of H1N1 viruses. Finally, this scientific article represents the first research to design and test specific oligonucleotide primers for the detection of local H1N1 viral strains in Kingdom of Saudi Arabia.

## P31 Identification of novel genetic variations affecting Osteoarthritis patients

### Haneen S Alsehli^1,2^, Ashraf Dallol^2^, Abdullah M Gari^3^, Mohammed M Abbas^1,4^, Roaa A Kadam^5^, Mazen M. Gari^5^, Mohmmed H Alkaff ^1,4^, Adel M Abuzenadah^2,6^, Mamdooh A Gari^1,2,6^

#### ^1^Sheikh Salem Bin Mahfouz Scientific Chair for Treatment of Osteoarthritis by Stem Cells, King Abdulaziz University, Jeddah, Saudi Arabia; ^2^Center of Innovation in Personalized Medicine, King Abdulaziz University, Jeddah, Saudi Arabia; ^3^Department of Hematology, Faculty of Medicine, King Abdulaziz University Hospital, King Abdulaziz University, Jeddah, Saudi Arabia; ^4^Department of Orthopedic Surgery, Faculty of Medicine, King Abdulaziz University, Jeddah, Saudi Arabia; ^5^Stem Cell Unit, Centre of Excellence in Genomic Medicine Research, King Abdulaziz University, Jeddah, Saudi Arabia; ^6^Department of Medical Laboratory Technology, Faculty of Applied Medical Sciences, King Abdulaziz University, Jeddah, Saudi Arabia

##### **Correspondence:** Mamdooh A Gari (mgari@kau.edu.sa) – Sheikh Salem Bin Mahfouz Scientific Chair for Treatment of Osteoarthritis by Stem Cells, King Abdulaziz University, Jeddah, Saudi Arabia

**Background**

Osteoarthritis (OA) is a progressive joint disease characterized by gradual degradation of extracellular matrix (ECM) components in the cartilage and bone. The ECM of cartilage is highly specified structure that is mainly composed of type II collagen that provides tensile strength to the tissue, aggrecan and proteoglycans. However, changes in the ECM composition and structure can lead to collagen type II loss, and network integrity. Several risk factors have been correlated with OA including age, genetic predisposition, hereditary factors, obesity, mechanical injuries, and joint trauma. Certain genetic association studies have been identified several genes associated with OA using genome-wide association studies (GWASs).

**Materials and methods**

To understand the pathology of OA and changes in the ECM that could be caused by genetic mutation, we performed a pilot whole-exome sequencing study on blood samples obtained from five end-stage OA patients with an age range of 46 –70 years old.

**Results**

Although no common genetic factors have been found, we identified several novel genetic variants affecting genes that function in several candidate causative pathways including immune response, inflammatory and cartilage degradation such as SELP, SPN, COL6A6 and COL7A1.

**Conclusions**

The approach of exome sequencing can be a promising method to identify gene mutations that influence the OA disease.

**Acknowledgements**

We would like to acknowledge the financial support provided by the Sheikh Salem Bin Mahfouz Scientific Chair for Treatment of Osteoarthritis by Stem Cells. The Center of Innovation in Personalized Medicine (CIPM), Stem Cell Lab at Center of Excellence for Genomic Medicine Research and the Orthopedics Stem Cell Research Lab (CEGMR) at King Abdulaziz University Hospital for supporting this study.

## P32 An integrated database of GWAS SNVs and their evolutionary properties

### Heba Abusamra^1^, Sajjad Karim^1^, Hend F Nour eldin^1^, Elham M Alhathli^1^, Nada Salem^1^, Sudhir Kumar^1,2^, Mohammed H Al-Qahtani^1^

#### ^1^Center of Excellence in Genomic Medicine Research, King Abdulaziz University, PO Box 80216, Jeddah 21589, Saudi Arabia; ^2^Institute for Genomics and Evolutionary Medicine, Temple University, Philadelphia, PA 19122, USA

##### **Correspondence:** Sajjad Karim (skarim1@kau.edu.sa) – Center of Excellence in Genomic Medicine Research, King Abdulaziz University, PO Box 80216, Jeddah 21589, Saudi Arabia

**Background**

Genome-wide repository of associations between SNPs and phenotypes (GRASP) is a centralized repository of publically available genome-wide association study (GWAS) results. GRASP v2.0 contains ∼ 8.87 million SNP associations reported in 2044 studies and ~178 thousand phenotypes [1]. However, this database does not contain evolutionary information about individual SNVs, particularly the prioritization of SNVs by using evolutionary conservation of SNVs.

**Materials and methods**

We built a new relational database that contains information from GRASP v.2.0 as well as evolutionary information. The resulting SQL database was subjected to MATLAB analysis in order to compute secondary fields, including the statistical replication of SNVs over studies and evolutionary information for each SNV. All SNVs were mapped to genome position of human genome build 38 (hg38) using the LiftOver resource.

**Results**

We developed an integrated E-GRASP-DB that provides detailed information of SNPs, an efficient examination of past GWAS results for diverse sets of researchers to complete various qualitative and quantitative analyses. Statistical SNV replication category provides information about replication of SNVs in studies, replication of SNVs in phenotype, SNVs replication in each study of each phenotype, and number of unique studies for each SNV. Another replications based on studies include number of SNVs in each study, studies replication in phenotype, and number of unique SNVs in each study (Table 9). Further, evolutionary information includes E-value for SNP phenotype association, rank of the evolutionary rate of the position, and rank of the evolutionary time span of the position that have been retrieved from the E-rank web server for each SNP [2].

**Conclusions**

Our E-GRASP database has additional information related to SNPs replication and evolutionary scores, facilitating better data interpretation and new hypotheses to be generated.

**Acknowledgements**

Authors would like to acknowledge the Deanship of Scientific Research, King Abdulaziz University*,* Jeddah*,* Saudi Arabia for funding the research *(*HiCi-1434-117-2)*.*

**References**

1. Eicher JD, Landowski C, Stackhouse B, Sloan A, Chen W, Jensen N, Lien J-P, Leslie R, Johnson AD: **GRASP v2. 0: an update on the Genome-Wide Repository of Associations between SNPs and phenotypes.***Nucleic acids research* 2015, **43:**D799-D804.

2. Dudley JT, Chen R, Sanderford M, Butte AJ, Kumar S: **Evolutionary meta-analysis of association studies reveals ancient constraints affecting disease marker discovery.***Molecular biology and evolution* 2012, **29:**2087-2094.

**Table 9 (abstract P32):** New statistical and evolutionary information included in E-GRASP

SNP ID	Statistical Replication		Evolutionary Information	
*rs4719450*	RepInPMID	3	Evalue	7.06E-12
	RepInPhenotype	3	Rate(x1000)	7558
	RepInEachPMID	1	FetchTime	265
	RepInEachPMIDinPhentotype	1	Prank	93828
	UniqueStudyInSNP	3	Erank	103468
	NumofSNPsinPMID	546944	E-P	9640
	RepPhenotypeinPMID	546944		
	UniqueSNPinPMID	355701		

## P33 Familial hypercholesterolemia in Saudi Arabia: prime time for a national registry and genetic analysis

### Fatima A. Moradi^1,2^, Omran M. Rashidi ^2^, Zuhier A. Awan^3^

#### ^1^Department of Biology, Genomic and Biotechnology Section, King Abdulaziz University, Jeddah, Saudi Arabia; ^2^Princess Al-Jawhara Al-Ibrahim Centre of Excellence in Research of Hereditary Disorders, Faculty of Medicine, King Abdul-Aziz University, Jeddah, Saudi Arabia; ^3^Department of Clinical Biochemistry, Faculty of Medicine, King Abdulaziz University, Jeddah, Saudi Arabia

##### **Correspondence:** Zuhier A. Awan (zawan@kau.due.sa) – Department of Clinical Biochemistry, Faculty of Medicine, King Abdulaziz University, Jeddah, Saudi Arabia

**Background**

The story of Familial Hypercholesterolemia (FH) in the Middle East started fifty years ago with the first description of Essential Familial Hypercholesterolemia in Lebanese families by Khachadurian in 1962. Later the field of FH and atherosclerosis expanded exponentially with the discovery of the LDL-receptor (LDLR) defect in fibroblast of homozygote FH individuals leading the way for Goldstein and Brown to receive the Nobel prize. Later numerous mutations in the *LDLR* gene where reported.

**Results**

Through record keeping of FH individuals we exposed the global under reporting, poor management and premature death in undiagnosed FH cases in Saudi Arabia. In addition, mapping private mutations and the emergence of the third culprit (*PCSK9* gene) associated with FH has flourished the concept of the heterogeneity in FH and the gene-gene interaction. Furthermore, the advance imaging of atherosclerosis in FH had aided in the understanding of the phenomena of aortic calcification and the essential role of inflammation as a neglected marker and target for intensive therapy in these individuals.

**Conclusions**

Efforts to maintain a registry and a cascade screening program for FH are undergoing to improve the recognition of FH in Saudi Arabia. Increase awareness of the number one genetic risk for CVD, which is FH, and the preventive strategies to reduce premature atherosclerosis are expected to improve FH life expectancy.

## P34 Comparative genomics and network-based analyses of early hepatocellular carcinoma

### Ibrahim Hamza Kaya^1^, Olfat Al-Harazi^2^, Dilek Colak^2^

#### ^1^Future Scientist Program Trainee at Genetics Department, King Faisal Specialist Hospital and Research Centre, Riyadh, Saudi Arabia; ^2^Department of Biostatistics, Epidemiology and Scientific Computing, King Faisal Specialist Hospital and Research Centre, Riyadh, Saudi Arabia

##### **Correspondence:** Dilek Colak (dcolakkaya@kfshrc.edu.sa) – Department of Biostatistics, Epidemiology and Scientific Computing, King Faisal Specialist Hospital and Research Centre, Riyadh, Saudi Arabia

**Background**

Hepatocellular carcinoma (HCC) is the fourth most common cancer type, and has one of the highest mortality rates among cancer patients. The disease is more common in Eastern Mediterranean nations, probably due to a significant contribution of hepatitis C virus (HCV) and hepatitis B infection. The disease is mostly diagnosed at advanced stages, and therefore has poor prognosis. Previously, we have developed a rat model of liver cancer for the identification distinct molecular mechanisms for early HCC. In this study, we gathered recent human HCC genomic data sets and performed cross-species comparative genomic analyses and identified a gene list that may be most relevant to early HCC. Moreover, we validated our gene-list on the gene expression profiling of peripheral blood mononuclear cells (PBMC) from patients with HCC. Furthermore, using our gene list we performed functional annotation, gene networks and pathway analyses.

**Results**

We identified potential gene signature that is conserved across rat and human HCCs, and validated its diagnostic value on the PBMC from patients with HCC dataset as well as independently performed HCC datasets. Our results indicate alterations in a number of cancer related pathways and critical for early HCC transformation.

**Conclusions**

The results suggest that network analysis coupled with cross-species genomic analysis may provide a robust method to identify key biological programs associated with early HCC and may lead to improved diagnosis and therapeutic options.

## P35 A TALEN-based oncolytic viral vector approach to knock out ABCB1 gene mediated chemoresistance in cancer stem cells

### Nabila A Alkousi, Takis Athanasopoulos

#### University of Wolverhampton, Wolverhampton, West Midlands, WV1 1LY, UK

##### **Correspondence:** Nabila A Alkousi (n.alkousi@wlv.ac.uk) – University of Wolverhampton, Wolverhampton, West Midlands, WV1 1LY, UK

**Background**

Finding a treatment for various types of cancer is a key challenge with very poor clinical outcomes. Chemoresistance induced by cancer stem cells (CSCs) and subsequent tumour relapses are major obstacles in cancer chemotherapy [4,5]. ABCB1 is a gene which is highly expressed in CSCs that encodes for the P-glycoprotein (P-pg)/ multidrug resistance protein (MDR), protecting the cancer cell from anticancer drugs [2]. To overcome the chemoresistance ability in cancer cells and/or CSCs, we designed a TALEN-based oncolytic viral vector approach as a genome editing tool to knockout the ABCB1 gene and inhibit over expression of P-gp in CSCs [1,3].

**Materials and methods**

Several bioinformatics tools (Table 10) were utilized to determine structure/function relationships of ABCB1 gene and to design TALENs.

**Results**

E-TALEN/De-novo software shows 12 designs depicting nucleotide sequence, transcript and exon, RVD sequence score and percent of total transcripts hit. TALEN designs with highest scores were selected based on i.e. length of binding site / (DNA composition Variation + RVD Composition). ABCB1_14_59842 had the higher score of the successful designs and no off-target theoretical crossmatches with a score of 1.379 whereas RVD recognized the nucleotide sequence of ABCB1 gene in ENST00000265724 at exon 5. The RVD sequence of the TALEN design ABCB1_14_59842 was evaluated by using E- TALEN/Evaluation software. In order to obtain functional TALENs, repeats were generated into the Sangamo plasmid model and forward and reverse TALEN plasmids map were constructed by using ApE plasmid editing tool.

**Conclusions**

Genetically engineered TALENs with repeat variable diresidues (RVD) which specifically target and cleave the ABCB1 gene via DSB/NHEJ mechanisms at exon 5, were designed. These can be delivered by recombinant oncolytic viral vectors that specifically target CSCs. ABCB1 knockouts and reversion of chemoresistance in CSCs could potentially improve the outcome of chemotherapy in cancer patients.

**References**

1. Cripe T, Wang P, Marcato P, Mahller Y and Lee P: **Targeting Cancer initiating Cells With Oncolytic Viruses**. *Mol Ther* 2009, **17**(10):1677-1682.

2. Gottesman M, Fojo T and Bates **S: Multidrug resistance in cancer: role of ATP-dependent transporters**. *Nature ReviewsCancer* 2002, **2**(1):48-58.

3. Joung J and Sander J: **TALENs: a widely applicable technology for targeted genome editing**. *Nature Reviews Molecular Cell Biology* 2012, **14**(1):49-55.

4. Matchett K and Lappin T: **Concise Reviews: Cancer Stem Cells: From Concept to Cure.***Stem Cells* 2013, **32**(10): 2563-2570.

5. Nguyen L, Vanner R, Dirks P and Eaves C: **Cancer stem cells: an evolving concept.***Nature Reviews Cancer* 2012, **12**(2):133-143

**Table 10 (abstract P35):** Software/databases used for design a TALENs plasmid knockout system for the ABCB1 gene

Software/Database	Description	URL (Uniform Resource Locator)
The Ensembl	Produces available online genome databases.	http://www.ensembl.org/index.html
E-TALEN/De-novo	Design a TALEN for specific single targeted gene.	http://www.e-talen.org/E-TALENdesigntalens.html
E-TALEN/Evaluation	Evaluate existing designs of TALEN.	http://www.e-talen.org/E-TALEN/reannotate_talens.html
The TAL Plasmids Assembly Tool	Analyze the TALEN and confirmed the RVD sequences.	http://bao.rice.edu/Research/BioinformaticTools/assembleTALSequences.html
ApE software	Plasmid editor.	http://biologylabs.utah.edu/jorgensen/wayned/ape/

**Fig. 11 (abstract P35):**
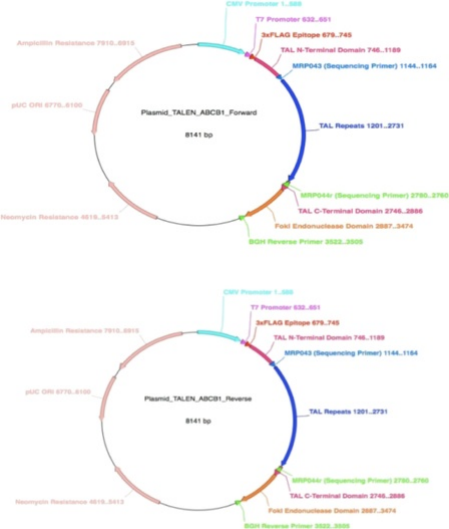
The model of the final forward and reverse plasmids for the ABCB1 TALEN

## P36 Cartilage differentiation and gene expression of synovial fluid mesenchymal stem cells derived from osteoarthritis patients

### Afnan O Bahmaid^1^, Etimad A Alhwait^1^, Mamdooh A Gari^2,3,4^, Haneen S Alsehli^5^, Mohammed M Abbas^3,6^, Mohammed H Alkaf^3,6^, Roaa Kadam^4^, Ashraf Dallol^5^, Gauthaman Kalamegam^3,4^

#### ^1^Department of Biochemistry, Faculty of Science, King Abdulaziz University, Jeddah, Saudi Arabia; ^2^Department of Medical Laboratory Technology, Faculty of Applied Medical Sciences, King Abdulaziz University, Jeddah, Saudi Arabia; ^3^Sheikh Salem Bin Mahfouz Scientific Chair for Treatment of Osteoarthritis by Stem Cells, King Abdulaziz University, Jeddah, Saudi Arabia; ^4^Stem Cells Unit, Center of Excellence in Genomic Medicine Research (CEGMR), King Abdulaziz University, Jeddah, Saudi Arabia; ^5^Center of Innovation in Personalized Medicine, King Abdulaziz University, Jeddah, Saudi Arabia; ^6^Department of Orthopaedic Surgery, Faculty of Medicine, King Abdulaziz University Hospital, King Abdulaziz University, Jeddah, Saudi Arabia

##### **Correspondence:** Gauthaman Kalamegam (kgauthaman@kau.edu.sa) – Sheikh Salem Bin Mahfouz Scientific Chair for Treatment of Osteoarthritis by Stem Cells, King Abdulaziz University, Jeddah, Saudi Arabia

**Background**

Osteoarthritis (OA), a progressive disease of synovial joints represents failed repair of joint damage that results from stresses that may be initiated by an abnormality in any of the synovial joint tissues [1]. Synovial fluid contains mesenchymal stem cells (MSCs) that contribute to cartilage regeneration [2]. The aim of this study is to derive the MSCs from the synovial fluid (SF-MSCs), characterize them for stemness and evaluate their differentiation potential into chondrocytes and related gene expression.

**Materials and methods**

Synovial fluid samples were collected from patients following institutional ethical approval and informed patient consent. Derivation SF-MSCs were done using earlier established protocol and were assessed for their morphology (Phase contrast microscopy), cell viability and proliferation (MTT assay), CD marker expression (flow cytometry) and cartilage related gene expression (qRT-PCR).

**Results**

**Fig. 12 (abstract P36):**
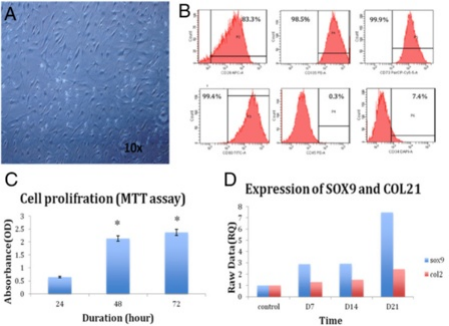
**a** Phase contrast microscopic image of synovial fluid-MSC (SF-MSC) showing the spindle-shape morphology (magnification 10x); **b** -Flow cytometry analysis of surface-marker expression on SF-MSC showing positive expression for CD73, CD90, CD105, CD29 and negative expression for CD34 and CD 45; **c** - Cell proliferation (MTT assay) of SF-MSCs at 24 h, 48 h and 72 h showing increase in cell proliferation with time; **d** - Real time gene expression analysis (qRT-PCR) showing increased expression of SOX9 and COL2A1

SF-MSCs were derived with greater efficiency and propagated to produce primary cell lines. Initial passages showed short fibroblastic morphology and increased proliferation (Fig. 12a) and were positive for MSCs related CD markers namely, CD73, CD105, CD90, CD29 and negative for CD34, CD45 (Fig. 12b). Cell proliferation by 229.42 % and 264.71 % at 48 h and 72 h compared to the 24 h respectively and these increases in values were statistically significant (Fig. 12c). qRT-PCR analysis showed increased expression of collagen II and SOX9 in the differentiated samples compared to the controls (Fig. 12d).

**Conclusions**

SF-MSCs were derived, expanded in culture and were also differentiated into chondrocytes that highly expressed cartilage related genes. Presence of MSCs from within the joint indicate the self healing mechanisms. Thus these cells might be a right choice of cell type for cartilage regeneration. In addition they may be able to provide closer insight into actual disease status enabling development of targeted therapies.

**Acknowledgements**

We acknowledge the fund provided by KACST(AT-35-438) and “Sheikh Salem Bin Mahfouz Scientific Chair for Treatment of Osteoarthritis by Stem Cells”, King Abdulaziz University and the Orthopaedics department at KAUH for providing the clinical material. We also greatly acknowledge the logistics provided by the Stem Cells Lab and the technical support by the flow cytometry unit at the Center of Excellence in Genomic Medicine Research.

**References**

1. Lane NE, Brandt K, Hawker G, Peeva E, Schreyer E, Tsuji W and Hochberg MC:**OARSI-FDA initiative: defining the disease state of osteoarthritis. Osteoarthritis Cartilage.** 2011,19:478–82.

2. Beekhuizen M, van Osch G, Bot A, Hoekstra M, Saris1D, Dhert W and Creemers L: **Inhibition of oncostatatin m in osteoarthritic synovial fluid enhances GAG production in osteoarthritic cartilage repair**. 2013,26:1473-2262.

## P37 E-GRASP: Adding an evolutionary component to the genome-wide repository of associations (GRASP) resource

### Hend F Nour Eldin^1^, Sajjad Karim^1^, Heba Abusamra^1^, Elham Alhathli^1^, Nada Salem^1^, Mohammed H Al-Qahtani^1^, Sudhir Kumar^1,2^

#### ^1^Center of Excellence in Genomic Medicine Research, King Abdulaziz University, Jeddah, Saudi Arabia; ^2^Institute for Genomics and Evolutionary Medicine, Temple University, Philadelphia, PA 19122, USA

##### **Correspondence:** Sajjad Karim (skarim1@kau.edu.sa) – Center of Excellence in Genomic Medicine Research, King Abdulaziz University, Jeddah, Saudi Arabia

**Background**

Genome-Wide Repository of Associations between SNPs and Phenotypes (GRASP) is an extensive database of publicly available genome-wide association study (GWAS) results, which are the genetic variants (single nucleotide variants, SNVs) associated with complex traits and diseases [1]. This resource is primarily a population genetics resource, which currently ignores the evolutionary information available for the positions harboring SNVs. We have developed a web application to make the information available in the GRASP2 resource along with the evolutionary information.

**Materials and methods**

In building the web application, we used JavaScript, Ajax and ASP.net to develop the client-side web interface which is served on the back-end by a SQL server, Matlab, D3.js, D3Plus.js and jQuery datatable. Popular dbSNP, dbSNP-Q and hgLiftOver resources were used for synchronizing and populating the database, in addition to the contect available at GRASP2.

**Results**

**Fig. 13 (abstract P37):**
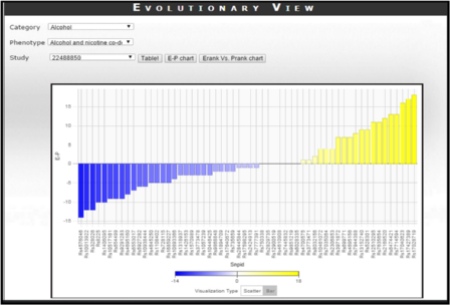
Erank–Prank (E-P) chart indicates evolutionary conservation information of SNPs

We developed a new web application and database named “E-GRASP” to enable easy and fast data retrieval of SNVs in GRASP and associated evolutionary information. E-GRASP presents three different views. (a) SNV View that contains information about each SNV such as SNPid, PMID, P value, chromosome number, position, number of studies reporting the SNV, and number of phenotypes associated with each SNV. (b) Study View that presents information on individual studies and basic information, including PMID, list of SNVs, their replication, phenotypes. (c) Evolutionary View that contains evolutionary conservation information as well as the E-rank [2] of SNVs in individual studies (plus P value, E value, P-rank, E-rank, and MAF) (Fig. 13).

**Conclusions**

E-GRASP will enable users to examine the GWAS data along with evolutionary information. In future, we plan to add more features and filters to improve it further.

**Acknowledgements**

Authors would like to acknowledge the Deanship of Scientific Studies, King Abdulaziz University*,* Jeddah*,* Saudi Arabia for funding the research *(*HiCi-1434-117-2*)*.

**References**

1. Eicher JD, Landowski C, Stackhouse B, Sloan A, Chen W, Jensen N, Lien J-P, Leslie R, Johnson AD: **GRASP v2. 0: an update on the Genome-Wide Repository of Associations between SNPs and phenotypes.***Nucleic acids research* 2015, **43:**D799-D804.

23. Dudley JT, Chen R, Sanderford M, Butte AJ, Kumar S: **Evolutionary meta-analysis of association studies reveals ancient constraints affecting disease marker discovery.***Molecular biology and evolution* 2012, **29:**2087-2094.

## P38 **Screening of*AGL*gene mutation in Saudi family with glycogen storage disease Type III**

### Salma N Alsayed^1^, Fawziah H Aljohani^1^, Samaher M Habeeb^1^, Rawan A Almashali^1^, Sulman Basit^2^, Samia M Ahmed^1^

#### ^1^Medical Laboratories Technology Department, Applied Medical Science College, Taibah University, Al Madinah Al Munawarah, Saudi Arabia; ^2^Center for Genetics and Inherited Diseases, Taibah University, Al Madinah Al Munawarah, Saudi Arabia

##### **Correspondence:** Salma N Alsayed (mt91.salma@gmail.com) – Medical Laboratories Technology Department, Applied Medical Science College, Taibah University, Al Madinah Al Munawarah, Saudi Arabia

**Background**

Glycogen storage disease (GSD) type III is autosomal recessive disease affects several organs as liver, heart and skeletal muscles. It is due to glycogen debranching enzyme deficiency that is responsible for glycogenolysis and is encoded by amylo-1, 6-glucosidase, 4-α-glucanotransferase (*AGL*) gene. We aim in this study to screen *AGL* gene mutation in a Saudi family with GSD type III from Al-Madinah Al-Munawwarah.

**Materials and methods**

Blood samples from 6 members of a family were collected including two affected with GSD III. DNA was extracted using QIAGEN Mini Kit. Quality of DNA was tested using gel electrophoresis and UV visualization. Polymerase chain reaction (PCR) was done to amplify all exons of *AGL* gene. PCR products were purified by QIAquick Purification Kit. The products of cycle sequencing were purified using X-terminator PCR Purification kit. The targeted exons were sequenced by ABI 3500 genetic analyzer. The data were analyzed using BioEdit Sequence Alignment Editor.

**Results**

Clinical manifestations of hepatomegaly was observed in two affected individuals. Laboratory investigations revealed an increase in creatinine kinase and transaminases. No potential sequence variants were found when patients DNA sequence was compared with reference sequence of exons and splice junctions of *AGL* gene.

**Conclusions**

Sequence analysis revealed no mutation in the patients DNA, while the possibility of presence of mutation in the regulatory region (promoter, enhancer) of this gene cannot be excluded.

## P39 High throughput proteomic data suggest modulation of cAMP dependent protein kinase A and mitochondrial function in infertile patients with varicocele

### Rakesh Sharma^1^, Ashok Agarwal^1^, Damayanthi Durairajanayagam^1,2^, Luna Samanta^1,3^, Muhammad Abu-Elmagd^4,5^, Adel M. Abuzenadah^4,5^, Edmund S. Sabanegh^6^, Mourad Assidi^4,5^, Mohammed Al-Qahtani^4^

#### ^1^American Center for Reproductive Medicine, Cleveland Clinic, Cleveland, OH, USA; ^2^Physiology, Universiti Teknologi, Sungai Buloh, MARA, Malaysia; ^3^Redox Biology Laboratory, School of Life Science, Ravenshaw University, Orissa, India; ^4^Center of Excellence in Genomic Medicine Research, Jeddah, Saudi Arabia; ^5^KACST Center of Innovation in Personalized Medicine at King Abdulaziz University, Jeddah, Saudi Arabia; ^6^Urology Department, Cleveland Clinic, Cleveland, OH, USA

##### **Correspondence:** Ashok Agarwal (agarwaa@ccf.org) – American Center for Reproductive Medicine, Cleveland Clinic, Cleveland, OH, USA

**Background**

Varicocele is diagnosed in about 15 % of the adult male population and ~40 % of infertile males. The prevalence of varicocele is 35 % in men with primary infertility, but increases to 81 % in men with secondary infertility, implicating varicocele as a cause of progressive decline in fertility. Approximately 90 % of varicoceles occur unilaterally on the left side, while 10 % occur bilaterally. Despite extensive research, the exact cause of infertility in men having varicocele remains unknown. We utilized proteomic analyses to identify proteins and pathways that may be affected and responsible for infertility in men with varicocele.

**Materials and methods**

An *in-silico* analysis of proteomics data targeting proteins in spermatozoa from infertile men with varicocele (n = 5) both unilateral and bilateral and men of proven fertility (n = 5) were separated on 1-D gel electrophoresis. Proteins were digested with trypsin in gel and identified on a LTQ-Orbitrap Elite hybrid mass spectrometer system. The differentially expressed proteins identified were subjected to bioinformatics pathway analysis using STRING, IPA and Metacore software to find out the putative pathways involved in development of infertility in these patients.

**Results**

Ninety nine proteins were differentially expressed in the varicocele groups, of which nine were uniquely expressed in the fertile group and 2 proteins in the varicocele groups. Integrins (ITGM and ITGB2) are uniquely expressed in varicocele group. The underexpressed proteins in varicocele group include proteins involved in stress response and energy metabolism, molecular chaperones, vesicular transport, proteins necessary for chromatin compaction and epigenetic regulation and sperm function (Acrosin, AK7, SPA17, CACNA2D). The regulatory subunit of protein kinase A (PKA) was underexpressed in the varicocele group.

**Conclusions**

The *in silico* proteomic profiling reveals that mitochondrial dysfunction may lead to oxidative stress mediated anomalies in sperm function. Underexpression of PRKAR1A may lead to activation of Protein kinase A, a tissue specific extinguisher leading to dismantling of signaling in the spermatozoa of varicocele patients resulting in reduced fertilizing ability.

**Acknowledgements**

Dr. Muhammad Abu-Elmagd and Dr Mourad Assidi projects are funded by the National Plan for Science, Technology and Innovation (MAARIFAH) – King Abdulaziz City for Science and Technology (KACST) - the Kingdom of Saudi Arabia – awards number (13-CIPM-01) and (13-MED2190-03) respectively.

## P40 Significant protein profile alterations in men with primary and secondary infertility

### Ashok Agarwal^1^, Rakesh Sharma^1^, Luna Samanta^1,2^, Damayanthi Durairajanayagam^1,3^, Mourad Assidi^4,5^, Muhammad Abu-Elmagd^4,5^, Mohammed Al-Qahtani^4^, Adel M. Abuzenadah^4,5^, Edmund S. Sabanegh^6^

#### ^1^American Center for Reproductive Medicine, Cleveland Clinic, Cleveland, Ohio, USA; ^2^Redox Biology Laboratory, School of Life Science, Ravenshaw University, Orissa, India; ^3^Physiology, Universiti Teknologi MARA, Sungai Buloh, Malaysia; ^4^Center of Excellence in Genomic Medicine Research, King Abdulaziz University, Jeddah, Saudi Arabia; ^5^KACST Center of Innovation in Personalized Medicine, King Abdulaziz University, Jeddah, Saudi Arabia; ^6^Urology Department, Cleveland Clinic, Cleveland, OH, USA

##### **Correspondence:** Ashok Agarwal (agarwaa@ccf.org) – American Center for Reproductive Medicine, Cleveland Clinic, Cleveland, Ohio, USA

**Background**

Both primary and secondary infertility are due to an impairment of sperm function which is associated to some proteomic profile alterations. The objective of the present study is to compare the proteome profile of spermatozoa of fertile and infertile men irrespective of primary or secondary causes. The specific aim was to determine the differentially expressed proteins (DEPs) in infertile group involved in sperm function linked to failure of successful pregnancy in their partners.

**Materials and methods**

This prospective proteomic study analyzed proteins in spermatozoa from proven fertile (n = 5) and infertile men (n = 5). Proteins were extracted and separated by 1-D gel. Bands were digested with trypsin and analyzed on a LTQ-Orbitrap Elite hybrid mass spectrometer system. Protein identification was done using Mascot (Matrix Science, London, UK; version 2.3.02), SEQUEST (Thermo Fisher Scientific, San Jose, CA, USA; version 1.4.0.288) and X! Tandem (TheGPM, thegpm.org; version CYCLONE (2010.12.01.1). Mascot, Sequest and X! Tandem were set up to search the human reference with database assuming trypsin as the digestion enzyme. Functional annotations of proteins were obtained using bioinformatics tools and pathway databases.

**Results**

Proteins associated with functional annotations related to one or more of the search terms, namely, sperm function, motility, fertilization, stress response, acrosome reaction, and reproduction revealed 40 DEPs in infertility group, when compared with fertile group. The 35 underexpressed DEPs in the infertile men revealed dysregulation of post-translational modification, protein folding, heat-stress response, cell motility, DNA damage mediated apoptosis and mitochondrial inner membrane assembly. On the other hand, HIST1H2BA, a variant histone specifically required to direct the transformation of dissociating nucleosomes to protamine in male germ cells was overexpressed in infertile group.

**Conclusions**

Pathway analysis in our current study suggests that the dysregulated post-translational modification, protein synthesis, ubiquitination and proteosomal break down may lead to accumulation of defective proteins in the spermatozoa, which consequently could lead to compromised sperm function. This may result in unsuccessful pregnancy.

**Acknowledgements**

Dr. Muhammad Abu-Elmagd and Dr Mourad Assidi projects are funded by the National Plan for Science, Technology and Innovation (MAARIFAH) – King Abdulaziz City for Science and Technology (KACST) - the Kingdom of Saudi Arabia – awards number (13-CIPM-01) and (13-MED2190-03) respectively.

## P41 Spermatozoa maturation in infertile patients involves compromised expression of heat shock proteins

### Luna Samanta^1,2^, Ashok Agarwal^1^, Rakesh Sharma^1^, Zhihong Cui^1,3^, Mourad Assidi^4,5^, Adel M. Abuzenadah^4,5^, Muhammad Abu-Elmagd^4,5^, Mohammed Al-Qahtani^4^

#### ^1^American Center For Reproductive Medicine, Cleveland Clinic, Cleveland, OH, USA; ^2^Redox Biology Laboratory, School of Life Science, Ravenshaw University, Orissa, India; ^3^Institute of Toxicology, Third Military Medical University, Chongqing, China; ^4^Center of Excellence in Genomic Medicine Research, King Abdulaziz University, Jeddah, Saudi Arabia; ^5^KACST Center of Innovation in Personalized Medicine, King Abdulaziz University, Jeddah, Saudi Arabia

##### **Correspondence:** Ashok Agarwal (agarwaa@ccf.org) – American Center For Reproductive Medicine, Cleveland Clinic, Cleveland, OH, USA

**Background**

Reduced expression of heat shock proteins particularly, HSPA2 is associated with oligozoospermia, increased frequency of chromosomal aneuploidies, DNA fragmentation, apoptosis, abnormal morphology, cytoplasmic retention, reduced fertility potential, and even pregnancy failure following in vitro fertilization (IVF). Despite the importance of HSPA2 in spermatogenesis and fertility, there is a lack of reports on the role of HSPA2 in human sperm maturation. The present study was designed to identify the pathways involved in protein turn-over, particularly heat shock proteins in spermatozoal function from infertile patients; and subsequently validate the role of testis-specific HSPs in sperm function.

**Materials and methods**

This prospective proteomic study analyzed proteins in spermatozoa from infertile men. Briefly, spermatozoa from infertile patients (n = 5) were fractionated on density gradient (80, 60 and 40 %) layers. Fraction 1 (F1) refers to the least mature stage having lowest density whereas the fraction 4 (F4) comprises of the most dense and morphologically mature motile spermatozoa. Fraction 2 (F2) and fraction 3 (F3) are the intermediate stages. Proteins were extracted and separated by 1-D gel. Bands were digested with trypsin and analyzed on a LTQ-Orbitrap Elite hybrid mass spectrometer system. Protein identification was done using Mascot, SEQUEST and X! Tandem which were set up to search the human reference with database assuming trypsin as the digestion enzyme. Functional analyses of proteins were performed using gene ontology analyses. Selected candidate proteins were validated by Western blot.

**Results**

A significant decreasing trend in spectral counts of molecular chaperones was observed from F1 to F4 fractions. Among the chaperones, HSPA2 was selected as it is testis specific and its average spectral count decreased from 707.7 in F1 to 276 in F4. This was validated by western blot. Another important HSP 70 family member HSPA1L was also validated and correlated with the pathway analysis data. Beta-Actin was taken as internal control. The results showed similar trend as per the proteomic analysis.

**Conclusions**

HSPA2 expression correlates significantly with sperm maturation process. Aberrant HSPA2 expression and downregulation of protein modification and folding may play an important role in sperm function and fertilization processes.

**Acknowledgements**

Dr. Muhammad Abu-Elmagd project is funded by the National Plan for Science, Technology and Innovation (MAARIFAH) – King Abdulaziz City for Science and Technology (KACST) - the Kingdom of Saudi Arabia – award number (13-CIPM-01).

## P42 Array comparative genomic hybridization approach to search genomic answers for spontaneous recurrent abortion in Saudi Arabia

### Alaa A Alboogmi^1^, Nuha A Alansari^1^, Maha M Al-Quaiti^1^, Fai T Ashgan^1^, Afnan Bandah^1^, Hasan S Jamal^2^, Abdullraheem Rozi^2^_,_ Zeenat Mirza^3^, Adel M Abuzenadah^1^, Sajjad Karim^1^, Mohammed H Al-Qahtani^1^

#### ^1^Center of Excellence in Genomic Medicine Research, King Abdulaziz University, Jeddah-21589, Saudi Arabia; ^2^Department of Obstetrics and Gynecology, King Abdulaziz University Hospital, Faculty of Medicine, King Abdulaziz University, Jeddah-21589, Saudi Arabia; ^3^King Fahd Medical Research Center, King Abdulaziz University, Jeddah-21589, Saudi Arabia

##### **Correspondence:** Sajjad Karim (skarim1@kau.edu.sa) – Center of Excellence in Genomic Medicine Research, King Abdulaziz University, Jeddah-21589, Saudi Arabia

**Background**

Continuously increasing world population is major concern today, however many couples are praying for a child as their desire to have baby results in combination of frustration, inadequacy and hopelessness, because of recurrent pregnancy loss or spontaneous recurrent abortion (SRA) [1]. Chromosome abnormalities has been reported as major reason for SRA and cytogenetics techniques are routinely used to detect chromosomal techniques, however, it has many limitations [2].

**Materials and methods**

In the present study, we applied high-density whole genome array- comparative genomic hybridization technique to identify all chromosome abnormalities in forty one SRA patients where classical cytogenetics G-band analysis technique fails to detect such abnormalities [3]. The array-CGH analysis was performed by Agilent sure print G3 Hmn CGH 2x 400 K arrays (Agilent Technologies, USA) following manufacturer’s protocol.

**Results**

**Fig. 14 (abstract P42):**
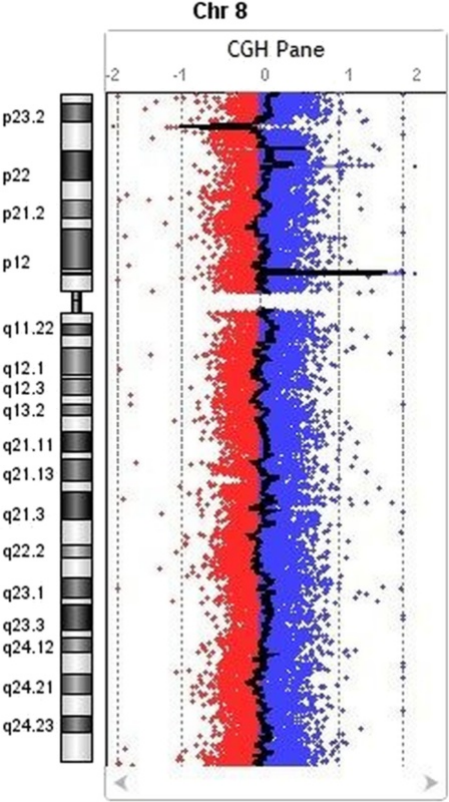
CGH array detected alteration in Chromosome 8 (8p23.1, 8p22, 8p11.23) of recurrent abortion patient

Array-CGH results showed ~800 losses and gains in different genomic regions of 41 SRA patients with cut off values of -1.0 for microdeletion and 0.8 for microduplication. We reported 35 frequent alterations that were present in >10 % of patients, including three macro-alteration (8p23.1, 10q11.21-q11.22, 15q11.2) and multiple micro-deletions/amplifications: 1p21.1 (AMY2A, AMY2B, AMY1A/B/C), 1q21.3 (LCE3C), 1q24.2 (NME7), 3p22.2 (CTDSPL), 4q13.2 (UGT2B17), 6p21.32 (HLA-DRB5,HLA-DRB6), 7p15.2 (SKAP2), 7p14.1(TARP), 7q34 (MGAM, PRSS1, PRSS2, MTRNR2L6, TRY6), 8p23.2(CSMD1), 8p22 (MSR1), 8p11.23 - p11.22 (ADAM5P,ADAM3A), 10q11.22(PPYR1, GPRIN2), 11q11 (OR4C11, OR4P4, OR4S2, OR4C6), 12p13.2 (PRH1, TAS2R46, TAS2R43, PRR4), 14q11.1-q11.2 (OR11H12, POTEG, POTEM, OR4Q3, OR4M1, OR4N2, OR4K2, OR4K5, OR4K1), 14q32.33 (KIAA0125,ADAM6,NCRNA00226), 20p13 (SIRPB1), 22q11.22 (MIR650, IGLL5), 22q11.23 (LOC391322,GSTT1, GSTTP2) (Fig. 14).

**Conclusions**

The study shows that whole genome array-CGH can be used in the identification of potential genes and chromosomal abnormalities underlying RSA problem. We have reported some of the novel CNVs/genes involved in the Saudi RSA patients. A more comprehensive procedure is required to validation the possible causative CNVs and genes in these regions to improve the diagnoses and treatment of RSA.

**Acknowledgements**

This project was funded by the National Plan for Science, Technology and Innovation (MAARIFAH) – King Abdulaziz City for Science and Technology, Saudi Arabia – award number (08-MED120-03).

**References**

1. Rai R, Regan L. **Recurrent miscarriage.***Lancet* 2006; **68**:601–11.

2. Goddijn M, Leschot NJ. **Genetic aspects of miscarriage**. *Baillieres Best Pract Res Clin Obstet Gynaecol* 2000; **14**(5):855–65.

3. Azmanov DN, Milachich TV, Zaharieva BM, et al. **Profile of chromosomal aberrations in different gestational age spontaneous abortions detected by comparative genomic hybridization**. *Eur J Obst Gynecol Reprod Biol* 2007; **131**:127–31.

## P43 Global gene expression profiling of Saudi kidney cancer patients

### Sajjad Karim^1^, Hans-Juergen Schulten^1^, Ahmad J Al Sayyad^2^, Hasan MA Farsi^2^, Jaudah A Al-Maghrabi^3,4^, Zeenat Mirza^5^, Reem Alotibi^1^, Alaa Al-Ahmadi^1^, Nuha A Alansari^1^, Alaa A Albogmi^1^, Maha M Al-Quaiti^1^, Fai T Ashgan^1^, Afnan Bandah^1^, Mohammed H Al-Qahtani^1^

#### ^1^Center of Excellence in Genomic Medicine Research, King Abdulaziz University, PO BOX 80216, Jeddah 21589, Saudi Arabia; ^2^Department of Urology, Faculty of Medicine, King Abdulaziz University, Jeddah, Saudi Arabia; ^3^Department of Pathology, Faculty of Medicine, King Abdulaziz University, Jeddah, Saudi Arabia; ^4^Department of Pathology, King Faisal Specialist Hospital and Research Center, Jeddah, Saudi Arabia; ^5^King Fahd Medical Research Center, King Abdulaziz University, PO BOX 80216, Jeddah 21589, Saudi Arabia

##### **Correspondence:** Sajjad Karim (skarim1@kau.edu.sa) – Center of Excellence in Genomic Medicine Research, King Abdulaziz University, PO BOX 80216, Jeddah 21589, Saudi Arabia

**Background**

Kidney cancer (KC) is the sixth-leading cause of cancer death worldwide [1]. KC in its metastatic stage is least responsive to treatment, however, early detection opens survival window by surgical resection. Therefore we need to investigate the exact molecular events leading to disease onset and progression of KC.

**Materials and methods**

We performed whole gene expression profiling of seven KC against five control using Affymetrix HuGene 1.0 ST arrays and Partek genomic suite v 6.6. IPA, a genome-wide biological pathway analysis package, was used to find significantly molecular networks and pathways associated with KC.

**Results**

We identified 1596 differentially expressed significant genes, 542 up and 1054 down regulated, with cutoff P value ≤ 0.05 and a fold change > 2; comparing KC with normal kidney tissues. The most significantly upregulated genes were small nucleolar RNA, C/D box 29 (SNORD29), hypoxia inducible lipid droplet-associated (HILPDA), caveolin 1 (CAV1), early B-cell factor 2 (EBF2), transforming growth factor, beta-induced (TGFBI), mitochondrially encoded tRNA cysteine (MT-TC), enolase 2 (ENO2), neuropilin (NRP) and tolloid (TLL)-like 2 (NETO2), anoctamin 4 (ANO4), versican (VCAN) and matrix metallopeptidase 16 (MMP-16) whereas downregulated genes were aldolase B, fructose-bisphosphate (ALDOB), solute carrier family12 (SLC12A3), calbindin1 (CALB1), uromodulin (UMOD), plasminogen (PLG), kininogen 1 (KNG1), nephrosis2 idiopathic, steroid-resistant (NPHS2), and potassium inwardly-rectifying channel subfamilyJ1 (KCNJ1). IPA based canonical pathway analysis shown LPS/IL-1 Mediated Inhibition of RXR Function, Valine Degradation I, Tryptophan Degradation X (Mammalian, via Tryptamine), Noradrenaline and Adrenaline Degradation, Serotonin Degradation and FXR/RXR Activation Signaling pathway to be significantly associated with our kidney cancer cases and this finding is in accordance with other finding [2, 3, 4].

**Conclusions**

Present study identified differentially expressed genes in kidney cancer of Saudi Arabian patients using whole transcript, high-density expression arrays. Our dataset is small but has a potential source for novel biomarker and may offer unique biological insights for kidney cancer.

**Acknowledgements**

This project was funded by the National Plan for Science, Technology and Innovation (MAARIFAH) – King Abdulaziz City for Science and Technology, Saudi Arabia – award number (10-BIO1258-03, 10-BIO1073-03 and 08-MED120-03).

**References**

1. Jemal A, Siegel R, Xu J, Ward E: **Cancer statistics, 2010**. *CA Cancer J Clin.* 2010, **60(5):** 277-300.

2. Young AN, Amin MB, Moreno CS, Lim SD, Cohen C, Petros JA, Marshall FF, Neish AS: **Expression profiling of renal epithelial neoplasms: a method for tumor classification and discovery of diagnostic molecular markers.***Am J Pathol* 2001, **158:** 1639-1651.

3. Gumz ML, Zou H, Kreinest PA, Childs AC, Belmonte LS, LeGrand SN, Wu KJ, Luxon BA, Sinha M, Parker AS, Sun LZ, Ahlquist DA, Wood CG, Copland JA. **Secreted frizzled-related protein 1 loss contributes to tumor phenotype of clear cell renal cell carcinoma*.****Clin Cancer Res.* 2007, **13(16)**: 4740-9.

4. Ross JS, Stagliano NE, Donovan MJ, Breitbart RE, and Ginsburg GS **Atherosclerosis: A Cancer of the Blood Vessels?** Am J Clin Pathol 2001, **116 (Suppl 1):** S97-S107.

## P44 Downregulated StAR gene and male reproductive dysfunction caused by nifedipine and ethosuximide

### Rasha A Ebiya^1,2^, Samia M Darwish^1,2^, Metwally M. Montaser^3,4^

#### ^1^Zoology Department, Women’s College for Arts, Science & Education, Ain Shams University, Cairo, Egypt; ^2^Biology Department, Faculty of Applied Science, Umm Al- Qura University, Makka Saudi Arabia; ^3^Zoology Department, Faculty of Science, Al-Azhar University, Cairo, Egypt; ^4^Biotechnology Department, Faculty of Science, Taif University, Taif, Saudi Arabia

##### **Correspondence:** Rasha A Ebiya (Rasha_ali_511@yahoo.com) – Zoology Department, Women’s College for Arts, Science & Education, Ain Shams University, Cairo, Egypt

**Background**

Calcium is important for male fertility in vasodilation, sperm development and several enzymatic reactions. It also serves as a second messenger to control acrosome reaction and sperm motility. Calcium channel-blockers (CCB) as nifedipine and ethosuximide are used in hypertension and epilepsy treatment can affect the male reproductive system. However, little is known about their side effects on chromosomes, StAR-gene, histology of testis and the underlying mechanism of the male reproductive dysfunction. The present study utilized various analyses including genotoxicity, histology and sperm analysis to address the involvement. of CCB in inducing male infertility.

**Materials and methods**

Thirty-six albino male mice were orally treated by 50 or 100 mg/kg body weight nifedipine and ethosuximide respectively for 20 days followed by another 10 days without treatment for drug withdrawal and assayed for chromosome aberrations; epididymal sperm count, motility, abnormal shape; and the testicular expressions of biomarkers gene including steroidogenic acute regulatory protein (StAR) gene were measured. In addition, the histologic structure of the testis was investigated to the process of spermatogenesis which indicating partly absent and atrophy and malformation.

**Results**

Mice administrated CCB showed a significant increase in the percentage of chromosome aberration and sperm shape change. In addition, expressions of StAR-mRNA was significantly down regulated. Sperm count and motility were significantly decreased. However, a slight improvement was observed in all tested parameters after drug withdrawal. All seminiferous tubules displayed total atrophy, more disruption, severe damage and more elongation of the tubules with disorganization of germinal epithelium that detached from the basement membrane. In addition, the lumen of seminiferous tubules showed the completely absence of sperm cells.

**Conclusions**

There is evidence that both nifedipine and ethosuximide cause a significant increase in chromosome abnormalities, decrease in sperm structure and function, and downregulation of StAR-mRNA expression. All these side effects may lead to irreversible male sterility.

## P45 Clustering based gene expression feature selection method: A computational approach to enrich the classifier efficiency of differentially expressed genes

### Heba Abusamra^1,2^, Vladimir B. Bajic^3^

#### ^1^King Abdullah University of Science and Technology (KAUST), Computer, Electrical and Mathematical Sciences and Engineering Division, Thuwal, 23955-6900, Saudi Arabia; ^2^Center of Excellence in Genomic Medicine Research, King Abdulaziz University, PO Box 80216, Jeddah 21589, Saudi Arabia; ^3^King Abdullah University of Science and Technology (KAUST), Computational Bioscience Research Center, Thuwal, Jeddah 23955-6900, Saudi Arabia

##### **Correspondence:** Vladimir B. Bajic (vladimir.bajic@kaust.edu.sa) – King Abdullah University of Science and Technology (KAUST), Computational Bioscience Research Center, Thuwal, Jeddah 23955-6900, Saudi Arabia

**Background**

The native nature of high dimension low sample size of gene expression data make the classification task more challenging. Therefore, feature (gene) selection become an apparent need. Selecting a meaningful and relevant genes for classifier not only decrease the computational time and cost, but also improve the classification performance. Among different approaches of feature selection methods, however most of them suffer from several problems such as lack of robustness, validation issues etc. Here, we present a new feature selection technique that takes advantage of clustering both samples and genes.

**Materials and methods**

We used leukemia gene expression dataset [1]. The effectiveness of the selected features were evaluated by four different classification methods; support vector machines, k-nearest neighbor, random forest, and linear discriminate analysis. The method evaluate the importance and relevance of each gene cluster by summing the expression level for each gene belongs to this cluster. The gene cluster consider important, if it satisfies conditions depend on thresholds and percentage otherwise eliminated.

**Results**

**Fig. 15 (abstract P45):**
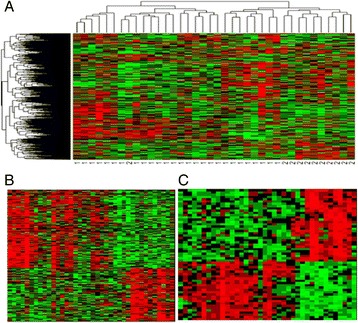
**a** Heat-map of differential expressed genes of leukemia. Row represents genes, and column represents samples, **b** Heat-map of refined differential expressed genes of leukemia for 1117 genes and **c** 58 genes

Initial analysis identified 7120 differentially expressed genes of leukemia (Fig. 15a), after applying our feature selection methodology we end up with specific 1117 genes discriminating two classes of leukemia (Fig. 15b). Further applying the same method with more stringent higher positive and lower negative threshold condition, number reduced to 58 genes have be tested to evaluate the effectiveness of the method (Fig. 15c). The results of the four classification methods are summarized in Table 11.

**Conclusions**

The feature selection method gave good results with minimum classification error. Our heat-map result shows distinct pattern of refines genes discriminating between two classes of leukemia.

**References**

1. Golub TR, Slonim DK, Tamayo P, Huard C, Gaasenbeek M, Mesirov JP, Coller H, Loh ML, Downing JR, Caligiuri MA, Bloomfield CD, Lander ES: **Molecular classification of cancer: class discovery and class prediction by gene expression monitoring.***Science*, 1999, **286**(5439):531-537.

**Table 11 (abstract P45):** Results of different classification methods on the test set

Leukimia Dataset			
Method	Accurecy	Sensitivity	Specificity
SVM			
linear	97.14%	100%	92.85%
polynomial	88.57%	100%	71.24%
radial	97.14%	100%	92.85%
sigmoid	97.14%	100%	92.85%
k-NN			
K = 1	94.28%	100%	85.71%
K = 3	97.14%	100%	92.85%
K = 5	94.28%	100%	85.71%
K = 7	91.42%	100%	78.85%
K = 9	85.71%	100%	64.29%
K = 11	77.14%	100%	42.86%
Random Forest	93.42%	99.52%	84.28%
LDA	91.42%	100%	78.57%

## P46 Prognostic significance of Osteopontin expression profile in colorectal carcinoma

### Jaudah Al-Maghrabi^1^, Wafaey Gomaa^1,2^, Mehenaz Hanbazazh^1^, Mahmoud Al-Ahwal^3^, Asia Al-Harbi^4^, Wejdan Al-Qahtani^4^, Saher Hakamy^4^, Ghali Baba^4^, Abdelbaset Buhmeida^4^, Mohammed Al-Qahtani^4^

#### ^1^Department of Pathology, King Abdulaziz University, Jeddah, Saudi Arabia; ^2^Department of Pathology, Faculty of Medicine, Minia University, Al Minia, Egypt; ^3^Department of Medicine, King Abdulaziz University, Jeddah, Saudi Arabia; ^4^Center of Excellence in Genomic Medicine Research, King Abdulaziz University, Jeddah, Saudi Arabia

##### **Correspondence:** Jaudah Al-Maghrabi (jalmaghrabi@hotmail.com) – Department of Pathology, King Abdulaziz University, Jeddah, Saudi Arabia.

**Background**

Osteopontin (OPN) is an extracellular matrix protein possess central role in many physiological and pathological processes including, tumorigenesis. It is over-expressed in a variety of solid tumors, including lung, breast, colorectal, stomach and ovarian cancers. It is believed that manipulation of OPN levels may be useful in the treatment of cancer metastasis. The purpose of this work is to study the association of OPN expression profile with several clinic-pathological variables and patient outcome in colorectal carcinoma (CRC).

**Patients and methods**

Hundred and Thirty-Four archival FFPE samples of CRC were collected from King Abdulaziz University Hospital, Saudi Arabia. Tissue microarrays were constructed and automated immunohistochemistry was done to evaluate the impact of expression patterns of OPN protein in CRC.

**Results**

OPN is expressed in both cytoplasm and nuclei. About 20 % and 23 % of the tumor samples showed high cytoplasmic and nuclear OPN expression patterns, respectively. There was no association between OPN expression patterns and gender, age, tumor size and location. However, borderline significant correlation was observed with lymph nodes status (p < 0.06) while significant correlations were observed with tumor grade (0.008), tumor invasion (0.01) and distant metastasis (0.04). Interestingly, the disease free survival (DFS) outcome of CRC patients, by using Kaplan-Meier analysis, showed that there was a significant (p < 0.05) variation in DFS between patients with high expression tumors as compared to those with low expression tumors in that patients with tumors of high expression stay alive longer.

**Conclusions**

The data imply that OPN expression might have an essential function in tumor invasion and distant metastasis and provides additional information in predicting patient outcome in CRC.

## P47 High Glypican-3 expression pattern predicts longer disease-specific survival in colorectal carcinoma

### Jaudah Al-Maghrabi^1^, Abdullah Al-Harbi^1^, Mahmoud Al-Ahwal^2^, Asia Al-Harbi^3^, Wejdan Al-Qahtani^3^, Sahar Hakamy^3^, Ghalia Baba^3^, Abdelbaset Buhmeida^3^, Mohammed Al-Qahtani^3^

#### ^1^Department of Pathology, King Abdulaziz University, Jeddah, Saudi Arabia; ^2^Department of Medicine, King Abdulaziz University, Jeddah, Saudi Arabia; ^3^Center of Excellence in Genomic Medicine Research, King Abdulaziz University, Jeddah, Saudi Arabia

##### **Correspondence:** Jaudah Al-Maghrabi (jalmaghrabi@hotmail.com) – Department of Pathology, King Abdulaziz University, Jeddah, Saudi Arabia

**Background**

Glypicans (GPC) are engaged in developmental morphogenesis, and have been involved in regulatory processes of several cell signaling pathways. Abnormal expression of glypicans has been observed in different cancer types, including ovarian, pancreatic, and breast cancers. The present work was designed to investigate the expression profiling of Glypican-3 (GPC-3) and its association with clinico-pathological features as well as prognostic significance in colorectal carcinoma (CRC).

**Patients and methods**

Hundred and Forty-Two archival FFPE samples of CRC were collected from King Abdulaziz University Hospital, Saudi Arabia. Tissue microarrays were constructed and automated immunohistochemistry was done in order to detect and evaluate the impact of expression patterns of GPC-3 protein in CRC.

**Results**

About 70 % of the tumor samples showed high cytoplasmic GPC-3expression, whereas 30 % of cases showed low expression patterns. There was no correlation between GPC-3 expression and age, gender, tumor grade, and lymph node status. However, borderline significant correlation was observed with tumor invasion (p < 0.06). Interestingly, in Kaplan-Meier survival analysis, there was a significant (p < 0.015) difference in disease-specific survival (DSS) between patients with high expression tumors (living significantly longer) and those with low expression patterns tumors.

**Conclusions**

GPC-3 is significantly associated to disease survival outcome, holding some prognostic significance. However, large cohort study is recommended in order to explore the molecular value of GPC-3 in CRC**.**

## P48 An evolutionary re-assessment of GWAS single nucleotide variants implicated in the Cholesterol traits

### Elham M Alhathli^1^, Sajjad Karim^1^, Nada Salem^1^, Hend Nour Eldin^1^, Heba Abusamra^1^, Sudhir Kumar^1,2^, Mohammed H Al-Qahtani^1^

#### ^1^Center of Excellence in Genomic Medicine Research, P.O. Box 80216, King Abdulaziz University, Jeddah 21589, Saudi Arabia; ^2^Institute for Genomics and Evolutionary Medicine, Temple University, Philadelphia, PA 19122, USA

##### **Correspondence:** Sajjad Karim (skarim1@kau.edu.sa) – Center of Excellence in Genomic Medicine Research, P.O. Box 80216, King Abdulaziz University, Jeddah 21589, Saudi Arabia.

**Background**

A number of loci associated with total cholesterol concentration and cardiovascular diseases are identified by genome-wide association studies (GWAS). The Genome-Wide Repository of association between SNPs and phenotype (GRASP v2.0) database has most of the publically available data for diverse phenotypes including total cholesterol [1]. These direct association results do not account for difference in evolutionary conservation of positions and the power of the test, which may not identify all SNPs with significant associations [2]. We applied the E-ranking approach, based on P value, allele frequency, and evolutionary conservation score, on total cholesterol data in GRASP to reassess the significant and reproducible genetic disease associations [2].

**Materials and methods**

We retrieved 232,051 single nucleotide variants (SNVs) for a total cholesterol GWAS. We generated ranks of all SNVs using the E-value produced by the E-rank web server [2]. We assessed the quality of the highest ranking SNVs based on the number of times they were reported in 79 other cholesterol studies available in the GRASP database.

**Results**

**Fig. 16 (abstract P48):**
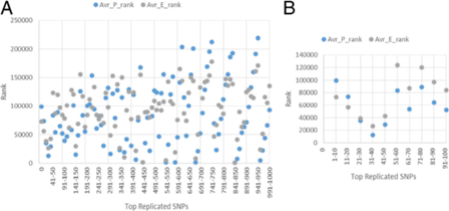
The average P-rank *vs*. E-rank of the top highly replicated SNPs. **a** top 1000 SNPs. **b** a zoom view of the top 100 SNPs

We found 3,173 of the top-5000 E-rank SNPs shared with the top 5000 P-rank SNPs. Overall, we indicated an improved average replication for top E-rank SNPs (average = 4) after removing bad P-rank SNPs (average = 3.5). Of the missense mutation causing SNPs, 15 out of 21 SNPs were identified by using combined E-rank/P-rank approach were underestimated using p-value alone. Two SNPs (rs1919127-C2orf16 and rs2266788-APOA5) of coding region were identified as highly associated with total cholesterol (Table 12). In contrast, we also found few top replicated SNPs in total cholesterol category with lower E-rank than Prank (Fig. 16).

**Conclusions**

Evolutionary ranking of genomic positions to filter disease associated SNPs can improve the reproducibility of SNPs at faster evolving sites and can enhance SNP discovery in published GWAS. These results are based on replication in studies with a limited number of significant SNPs, therefore, using full dataset can contribute significantly for improvement of the discovery of genetic variants in future work.

**Acknowledgements**

Authors would like to acknowledge Deanship of Scientific Research, Jeddah, Saudi Arabia (HiCi-1434-117-2) for funding the research.

**Reference**

1. Either JD, Landowska C, Stackhouse B, Sloan A, Chen B, Jensen N, Lien JP, Leslie R, and Johnson AD. **GRASP v2.0: an update on the Genome-Wide Repository of Associations between SNPs and phenotypes**. *Nucleic Acids Res.* 2015; 43(D1): D799-804.

2. Dudley JT, Chen R, Sander ford M, Butte AJ, Kumar S. **Evolutionary meta-analysis of association studies reveals ancient constraints affecting disease marker discovery**. *Mol Bio Evol.* 2012, 29(9): 2087-2094.

**Table 12 (abstract P48):** Example of SNPs with high replication and better E-rank than P-rank

SNP	P-value	P-rank	E-rank	In Gene	NoStudy	dbSNPfxn
rs1919127	2.01E-07	49	23	Homo sapiens chromosome 2 open reading frame 16 (C2orf16)	8	Missense
rs2266788	2.14E-07	52	40	Apolipoprotein A-V (APOA5)	26	5′ of gene within 2000bp

## P49 Derivation and characterization of human Wharton’s jelly stem cells (hWJSCs) *in vitro* for future therapeutic applications

### Aisha A Alyamani^1^, Gauthaman Kalamegam^2,3^, Etimad A Alhwait^1^, Mamdooh A Gari ^2,3,4^, Mohammed M Abbas ^3,5^, Mohammed H Alkaf ^3,5^, Haneen S Alsehli^6^, Roaa A Kadam^2^, Mohammed Al-Qahtani^2^

#### ^1^Department of Biochemistry, Faculty of Science, King Abdulaziz University, Jeddah, Saudi Arabia; ^2^Stem Cells Unit, Centre of Excellence in Genomic Medicine Research (CEGMR), King Abdulaziz University, Jeddah, Saudi Arabia; ^3^Sheikh Salem Bin Mahfouz Scientific Chair for Treatment of Osteoarthritis by Stem Cells, King Abdulaziz University, Jeddah, Saudi Arabia; ^4^Department of Medical Laboratory Technology, Faculty of Applied Medical Sciences, King Abdulaziz University, Jeddah, Saudi Arabia; ^5^Department of Orthopaedic Surgery, Faculty of Medicine, King Abdulaziz University, Jeddah, Saudi Arabia; ^6^Center of Innovation in Personalized Medicine, King Abdulaziz University, Saudi Arabia

##### **Correspondence:** Gauthaman Kalamegam (kgauthaman@kau.edu.sa) – Stem Cells Unit, Centre of Excellence in Genomic Medicine Research (CEGMR), King Abdulaziz University, Jeddah, Saudi Arabia

**Background**

Adult cartilage has limited intrinsic self-repair capacity and poor regeneration. New hope come from using stem cells for cartilage tissue engineering [1]. Mesenchymal stem cells especially those from the Wharton’s Jelly of the human umbilical cord (hWJSCs) represent an attractive source for chondrogenic differentiation, given their capacity for self-renewal, ease of access and lack of immunogenic or tumorigenic activities [2]. In the present study we aim to derive hWJSCs from the umbilical cords, characterize them for the mesenchymal stem cell markers, evaluate their morphology, proliferation and differentiate them into chondrocytes *in vitro.* Differentiation efficiency was evaluated using histology as well as cartilage related gene expression.

**Materials and methods**

Human umbilical cords were collected following ethical approval from the King Abdulaziz University (KAU) and informed patient consent. hWJSCs were derived using earlier established protocol and were assessed for their morphology (Phase contrast microscopy), cell proliferation (MTT assay), surface markers analysis (FACS), chondrogenic differentiation capacity (Toluidine Blue histology) and related gene expression (qRT-PCR).

**Results**

**Fig. 17 (abstract P49):**
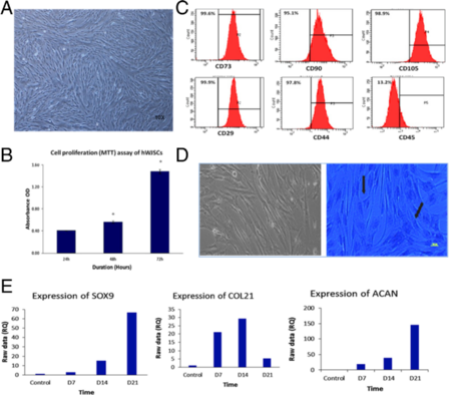
**a** Phase contrast micrograph of human umbilical cord Wharton’s Jelly stem cells (hWJSCs, 4x magnification); **b** Cell proliferation (MTT assay) of hWJSCs; **c** Flow cytometry analysis of surface-marker expression on hWJSCs showing positive staining for CD73 (top panel, left) , CD90 (top panel, middle), CD105 (top panel, right), CD29 (bottom panel, left), CD44 (bottom panel, middle), and negative staining CD 45 (bottom panel, right); **d** Toludine Blue histology of hWJSCs showing both control (left) and chondrocyte differentiation (right) following 21 days of culture in chondrocytic differentiation medium; **e** Gene expression analysis (qRT-PCR) showing increased expression of three markers SOX9 (left), COL2A1 (middle) and Aggrecan (right) respectively following chondrocytic differentiation of hWJSCs

hWJSCs were derived and propagated to produce primary cell lines. Early passages showed short fibroblastic morphology (Fig. 17a) and increased proliferation by 38.22 % and 263.80 % at 48 h and 72 h compared to the 24 h respectively (Fig. 17b). MSCs related surface markers namely CD73 (99.6 %), CD105 (98.9 %), CD90 (95.1 %), CD44 (97.8 %) and CD29 (99.9 %) were highly expressed (Fig. 17c). hWJSCs were successfully differentiated into chondrocytes (Fig. 17d) which showed increased expression of collagen II (COL2A1), aggrecan (ACAN) and SOX9 compared to the control (Fig. 17e).

**Conclusions**

hWJSCs was successfully derived with greater efficiency and expanded in culture. They were also differentiated into chondrocytes *in vitro*, that highly expressed cartilage related genes. Unlike BM-MSCs, hWJSCs can be harvested in abundance with no risk of donor site morbidity, are relatively young in nature, rich in proteoglycans, hypoimmunogeneic and non-tumorigeneic [2]. hWJSCs therefore can be used either alone or with biological nanoscaffolds for cartilage regeneration.

**Acknowledgements**

The financial support provided by King Abdulaziz City for Science and Technology (KACST) (AT-35-437), the “Sheikh Salem Bin Mahfouz Scientific Chair for Treatment of Osteoarthritis by Stem Cells” (11-557, DSR, KAU) as well as the logistics provided by the Stem Cells Lab at CEGMR; the Obstetrics & Gynaecology Department of the King Abdulaziz university Hospital and technical support by the flow cytometry unit are greatly acknowledged.

**References**

1. Kim DW, Staples M, Shinozuka K, Pantcheva P, Kang SD and Borlongan CV. **Wharton’s Jelly-Derived Mesenchymal Stem Cells: Phenotypic Characterization and Optimizing Their Therapeutic Potential for Clinical Applications.***Int J Mol Sci,*2013. **14** (6), 11692–11712.

2. Weiss ML, Anderson C, Medicetty S, Seshareddy KB, Weiss RJ, VanderWerff I, Troyer D, McIntosh KR. **Immune properties of human umbilical cord Wharton’s jelly-derived cells.***Stem Cells,* 2008. **26**, 2865–2874.

## P50 Attitudes of healthcare students toward biomedical research in the post-genomic era

### Rawan Gadi^1^, Abdelbaset Buhmeida^2^, Mourad Assidi ^2,3^, Adeel Chaudhary^2^, Leena Merdad^4^

#### ^1^Faculty of Dentistry, King Abdulaziz University, Jeddah, Saudi Arabia; ^2^Center of Excellence in Genomic Medicine Research, King Abdulaziz University, Jeddah, Saudi Arabia; ^3^KACST Technology Innovation Center in Personalized Medicine at King AbdulAziz University, Jeddah, Saudi Arabia; ^4^Dental public health department, Faculty of Dentistry, King Abdulaziz University, Jeddah, Saudi Arabia

##### **Correspondence:** Leena Merdad (lmerdad@kau.edu.sa) – Dental public health department, Faculty of Dentistry, King Abdulaziz University, Jeddah, Saudi Arabia

**Background**

Over the past decade, the field of genomics has witnessed important advances in terms of scope and used technologies [1]. The integration of genomics into biomedical research and clinical care has tremendous impacts on research, health and society [2]. Understanding the attitudes of healthcare students is a prerequisite to design appropriate research programs integrated into their educational curriculum (Double Helix Curriculum) in order to ensure perpetual development and innovation in biomedical technologies and applications[3, 4]. In Saudi Arabia, previous studies have focused only on medical students and used questionnaires that were not validated. The objectives of this study were (1) to assess the attitudes of healthcare students towards biomedical research; and (2) to investigate the factors affecting their attitudes.

**Materials and methods**

This cross-sectional study was conducted among all senior healthcare students (Faculties of Medicine, Dentistry, Pharmacology and Applied Medical Sciences), at King Abdulaziz University, Jeddah, Saudi Arabia using a validated Research Attitudes Questionnaire [5]. The questionnaire contained 11 items covering different aspects of biomedical research. Each item was rated on a 5-point Likert scale. A higher score indicates more positive attitudes towards biomedical research. Data were described using means and standard deviations. The t-test and one-way ANOVA were used to test the association between predictors and attitude scores.

**Results**

Out of 512 consent students, the general percentage attitude score was 69 %. The majority of students showed positive attitudes towards biomedical research although some showed negative attitudes towards certain aspects such as participant safety. The relationship between students’ attitudes and type of faculty and previous involvement in medical research was significant (*p-*value = 0.04 and 0.01, respectively.) In addition, higher knowledge of biobanking was correlated with positive attitudes towards biomedical research (Pearson’s r = 0.30, *p*-value < 0.001).

**Conclusions**

In general, health care students showed favorable attitude towards biomedical research. A noticeable effect of the students’ faculty and previous involvement in research on their attitudes towards biomedical research was observed. These findings suggest that healthcare schools should consider including teaching of advanced research methodology and OMICs-based approaches in their curricula as well as motivate students to participate in biomedical research activities.

**References**

1. Macaulay, I.C. and T. Voet, **Single cell genomics: advances and future perspectives**. *PLoS Genet*, 2014. **10**(1): p. e1004126.

2. Guttmacher, A.E. and F.S. Collins, **Realizing the promise of genomics in biomedical research**. *JAMA*, 2005. **294**(11): p. 1399-1402.

3. Abu-Zaid, A. and K. Alkattan, **Integration of scientific research training into undergraduate medical education: a reminder call**. *Med Educ Online*, 2013. **18**: p. 22832.

4. Solomon, S.S., et al., **Impact of medical student research in the development of physician-scientists**. *J Investig Med*, 2003. **51**(3): p. 149-56.

5. Rubright, J.D., et al., **Measuring how people view biomedical research: Reliability and validity analysis of the Research Attitudes Questionnaire**. *J Empir Res Hum Res Ethics*, 2011. **6**(1): p. 63-8.

## P51 Evaluation of the immunomodulatory effects of thymoquinone on human bone marrow mesenchymal stem cells (BM-MSCs) from osteoarthritic patients

### Saadiah M Alfakeeh^1^, Etimad A Alhwait^1^, Mamdooh A Gari^2,3,4^, Mohammed M Abbas^3,5^, Mohammed H Alkaf^3,5^, Haneen S Alsehli^6^, Roaa Kadam^4^, Gauthaman Kalamegam^3,4^

#### ^1^Department of Biochemistry, Faculty of Science, King Abdulaziz University, Jeddah, Saudi Arabia; ^2^Department of Medical Laboratory Technology, Faculty of Applied Medical Sciences, King Abdulaziz University, Jeddah, Saudi Arabia; ^3^Sheikh Salem Bin Mahfouz Scientific Chair for Treatment of Osteoarthritis by Stem Cells, King Abdulaziz University, Jeddah, Saudi Arabia; ^4^Stem Cells Unit, Centre of Excellence in Genomic Medicine Research (CEGMR), King Abdulaziz University, Jeddah, Saudi Arabia; ^5^Department of Orthopaedic Surgery, Faculty of Medicine, King Abdulaziz University Hospital, Jeddah, Saudi Arabia; ^6^Center of Innovation in Personalized Medicine, King Abdulaziz University, Saudi Arabia

##### **Correspondence:** Gauthaman Kalamegam (kgauthaman@kau.edu.sa) – Sheikh Salem Bin Mahfouz Scientific Chair for Treatment of Osteoarthritis by Stem Cells, King Abdulaziz University, Jeddah, Saudi Arabia

**Background**

Inflammatory events lead to altered immunological profile and acceleration of the disease pathology in osteoarthritis (OA). Thymoquinone (TQ) which is a major active chemical component of *Nigella sativa* is reported to have immunomodulatory properties which impart beneficial effects on bone and joint diseases [1]. We in the present study aim to evaluate the effect TQ on inflammatory and cell death related gene expression.

**Materials and methods**

Bone marrow aspirate from OA patients were obtained following institutional ethical approval (11-557). Primary cultures of BM-MSCs were established and basic characterization including cell morphology (phase contrast microscopy), MSC related CD marker expression (FACS) were done. BM-MSCs were treated with TQ and their effects on cell proliferation (MTT assay) and gene expression for IL-6, TNF-α, COX 2, BAX and BCL-2 were done using real-time PCR.

**Results**

**Fig. 18 (abstract P51):**
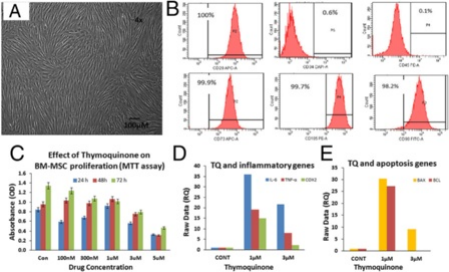
**a** Phase contrast microscopic image of Bone Marrow-MSCs showing the spindle-shaped morphology (Magnification 4x); **b** FACS image showing positive expression for CD29, CD73, CD90, CD105 and negative expression for CD 34, CD45; **c** Effect of Thymoquinone on BM-MSCs showing decrease in proliferation with increase in concentration of TQ; **d**, **e** Gene expression analysis of IL-6, TNF-α, COX2,BAX and BCL2 by real-time PCR following treatment of BM-MSCs ith (1 μM and 3 μM of TQ. GAPDH was the internal control. Data analysis and relative quantitation was done using comparative Ct method (ΔΔCt)

Derived BM-MSCs demonstrated characteristic fibroblastic morphology (Fig. 18a) and were highly positive for CD105, CD73, CD29, CD44 and CD90 and negative for CD34 & CD45 (Fig. 18b). BM-MSCs treated TQ showed mean maximal decrease in cell proliferation by 61.08 %, 66.84 % and 65.67 % for 24 h, 48 h and 72 h respectively compared to the control (Fig. 18c). Inflammation related genes namely IL-6, TNF-α and COX2 were decreased by 14.41, 10.92 and 12.55 fold respectively compared to the control (Fig. 18d). BAX was increased by 29.48 (1 μM) and 8.18 fold (3 μM) respectively, while BCl2 decreased by3.23 fold (1 μM) and were not detectable at 3 μM (Fig. 18e).

**Conclusions**

BM-MSCs were isolated with greater efficiency and expanded in culture. Treatment with TQ showed dose dependent decrease in inflammation related genes. TQ has been shown to have immunoregualtory effects on pancreatic ductal adenocarcinoma cells earlier [2]. Inhibitory effect on cell proliferation at higher concentrations indicate that TQ have also a cytotoxic effect. Hence, an optimal dose of TQ ranging between 1 μm and 3 μm may be useful in decreasing the inflammatory events without compromising required stem cell numbers for cartilage regeneration.

**Acknowledgements**

The financial support provided by KACST (AT-35-436), the “Sheikh Salem Bin Mahfouz Scientific Chair for Treatment of Osteoarthritis by Stem Cells (11-557, DSR, KAU)”, the training and support by the Stem Cells Unit and the flow cytometry unit at CEGMR and the clinical material provided by the Department of Orthopaedics, King Abdul Aziz University Hospital are greatly acknowledged.

**References**

1. Ahmad Nazrun Shuid, Norazlina Mohamed, Isa Naina Mohamed, Faizah Othman, Farihah Suhaimi, Elvy Suhana Mohd Ramli, Norliza Muhammad, and Ima Nirwana Soelaiman. **Nigella sativa: A Potential Antiosteoporotic Agent.***Evidence-Based Complementary and Alternative Medicine.* 2012. Article ID 696230, 1-6

2. Navdeep Chehl, Galina Chipitsyna, Qiaoke Gong, Charles J Yeo, Hwyda A Arafat. **Anti-inflammatory effects of the Nigella sativa seed extract, thymoquinone, in pancreatic cancer cells.***HPB (Oxford)*. 2009. **11**(5): 373–381.

## P52 Implication of IL-10 and IL-28 polymorphism with successful anti-HCV therapy and viral clearance

### Rubi Ghazala^1^, Shilu Mathew^2^, M.Haroon Hamed^3^, Mourad Assidi^2,4^, Mohammed Al-Qahtani^2^, Ishtiaq Qadri^5^

#### ^1^Department of Medicine, University of Health Science, Lahore, Pakistan; ^2^Center of Excellence in Genomic Medicine Research, King Abdulaziz University, Jeddah, Saudi Arabia; ^3^Department of Biology, King Abdul-Aziz University, Jeddah, Saudi Arabia; ^4^Center of Innovation in Personalized Medicine at King Abdulaziz University, Jeddah, Saudi Arabia; ^5^King Fahd Medical Research Center, King Abdul Aziz University, Jeddah, Saudi Arabia

##### **Correspondence:** Ishtiaq Qadri (ishtiaq80262@yahoo.com) – King Fahd Medical Research Center, King Abdul Aziz University, Jeddah, Saudi Arabia

**Background**

Hepatitis C virus the main reason of chronic liver ailment and liver cancer globally and is distributed into six discrete genotypes through the world with numerous subtypes in each genotype (1-6). The response and extent of interferon treatment is genotype specific and host restriction. Number of cellular genes are involved in this process including TBXA2R (G-protein coupled receptor), TRAF2 (adapter protein), LTbeta which is a membrane protein, NFkappaB2 and RelA (transcriptional factors), SNARK and MKK7 (protein kinases) and two diligently associated TNF/lymphotoxin pathway. A reported polymorphisms (SNPs) in these and other genetic factor are involved in viral clearance and chronicity. Genetic studies have also identified several SNPs round the interferon λ3 interleukin-28B, which are sturdily related with SVR to PEG-IFN and RBV cure for chronicity. In this study we examined SNP in IL10 and IL28B in HCV-GT3 infected individuals.

**Materials and methods**

A total of 349 patients of chronic hepatitis have been included in this genetic susceptibility study. The infected individuals were diagnosed anti-HCV positive and then confirmed by HCV Polymerized Chain Reaction (PCR) qualitative test. All these selected patients had been diagnosed for HCV and taken the standard treatment of interferon and ribavirin from 20 weeks to 36 weeks. The end product of PCR was confirmed by gel electrophoresis. The following restriction enzymes Ear I/, Rsa 1 and Mae III were used to digest the PCR product. This study focused only on patients who were non-responsive and had relapsed from conventional therapy.

**Results**

Our study proposes that both the interleukin genes interleukin 28B and IL-10 are found mutual in 2 foremost castes of the province Punjab. We determined that HCV GT-3a is precisely communal (84.0 %) amongst group of responders whereas GT-1a is more acquainted in relapser (66.2 %) as well as resistant (54.0 %). Genotype 4was excluded, the remaining 5 major genotypes, known as 1a (61.40 %), 2a (0.50 %), 2b (20.00 %), 3a (13.70 %) and an unidentified (4.40 %) were reported amongst the twelve various groups of Pakistan. Our findings designate that IL-10 and IL-28 genes may be intricate in the clearance of HCV GT3 in Asian population.

**References**

1. Mathew, S. *et al.***In silico studies of medicinal compounds against hepatitis C capsid protein from north Indi**a. *Bioinform Biol Insights***8**, 159-68 (2014).

2. http://www.hepatitiscentral.com/hepatitis-c/hepatitis-c-genotypes/.

3. Mathew, S. *et al.***Biomarkers for virus-induced hepatocellular carcinoma (HCC)**. *Infect Genet Evol***26**, 327-39 (2014).

4. Fatima, K. *et al.***Docking studies of Pakistani HCV NS3 helicase: a possible antiviral drug target**. *PLoS One***9**, e106339 (2014).

5. Mathew, S. *et al.***Computational Docking Study of p7 Ion Channel from HCV Genotype 3 and Genotype 4 and Its Interaction with Natural Compounds**. *PLoS One***10**, e0126510 (2015).

## P53 Selection of flavonoids against obesity protein (FTO) using *in silico* and *in vitro* approaches

### Shilu Mathew^1^, Lobna Mira^1^, Manal Shaabad^1^, Shireen Hussain^1^, Mourad Assidi^1,2^, Muhammad Abu-Elmagd^1^, Mohammed Al-Qahtani^1^

#### ^1^Center of Excellence in Genomic Medicine Research, King Abdulaziz University, Jeddah, Saudi Arabia; ^2^Center of Innovation in Personalized Medicine, King Abdulaziz University, Jeddah, Saudi Arabia

##### **Correspondence:** Mourad Assidi (mourad.assidi@gmail.com) – Center of Excellence in Genomic Medicine Research, King Abdulaziz University, Jeddah, Saudi Arabia

**Background**

Fat mass and obesity associated (FTO) is gene involved in obesity which affects more than 1/6 of the population worldwide [1]. FTO is located in the ‘p’ arm of the human chromosome 16 [2]. Certain variants of this gene are associated with obesity [3]. Research studies in humans and mice showed a role in cardiovascular and nervous systems and a robust association with obesity risk [4]. Natural products may play a effective role to prevent obesity especially those containing fibers, polyphenols, sterols, and alkaloids [5]. The aim of this study is to assess the use of selected flavonoids as potential obesity preventing agents targeting the three dimensional structure of FTO protein followed by an *in vitro* validation.

**Materials and methods**

An *in silico* approach based on well-established computational methods in quantitative structure-activity relationship, pharmacophore identification and computational docking software named CLC drug discovery Workbench were used [6]. Protein Data Bank was used to download the protein structure of FTO (3LFM). Selected natural compounds known to suppress the FTO action involved in obesity and lipid metabolism such as (a)Luteolin (*Reseda luteola)*,(b)Quercetin(*Emblica officinalis),*(c) Capsaicin(*Capsicum)*,(d)Abscisic acid(*Abscisin II*), (e)Ajoene(*Allium sativum*), and(f) Diosgenin(*Dioscorea villosa*) were subjected to comparative docking analysis. Subsequent *in vitro* validation of the selected compounds using cell culture will be envisioned.

**Results**

Flavonoid luteolin showed maximum affinity with a highest docking score of -25.632Kcal/mol, while Ajoene, Diosgenin and Quercetin showed less affinity towards FTO. The empathy of the selected natural compounds was in the order of Luteolin > Abscisic acid > Capsaicin > Ajoene > Quercetin > Diosgenin. Luteolin formed five H-Bonds in the active site of FTO protein at GLU161, ASP299, and ASP189 (3). Abscisic acid formed three H-Bonds at specific active sites such as ASP189, ARG196 and ASP299 with docking score of -28.636 Kcal/mol.

**Conclusions**

Flavonoids (particularly Luteolin) may act as an effective drug against FTO protein and could be therapeutically used for prevention of obesity. Further *in vitro* and *in vivo* validation will be necessary to also understand the underlying pathways.

**References**

1. https://www.ucl.ac.uk/news/news-articles/0713/15072013-How-obesity-gene-triggers-weight-gain-Batterham.

2. Jia G FY, Zhao X, Dai Q, Zheng G, Yang Y, Yi C, Lindahl T, Pan T, Yang YG, He C.: **N6-methyladenosine in nuclear RNA is a major substrate of the obesity-associated FTO**. *Nat Chem Biol* 2011; 7(12):885-887.

3. Loos RJ, Yeo GS: **The bigger picture of FTO: the first GWAS-identified obesity gene**. *Nat Rev Endocrinol* 2014; 10(1):51-61.

4. Alharbi KK, Syed R, Khan IA: **Computational study on the interaction of flavonoids with fat mass and obesity associated protein**. *J Environ Biol* 2015; 36(2):419-424.

5. Gamal A. Mohameda SRMI, Ehab S. Elkhayata, Riham Salah El Dine: **Natural anti-obesity agents**. *Bulletin of Faculty of Pharmacy* 2014; 52(2):269-284.

6. http://www.clcbio.com/products/clc-drug-discovery-workbench/.

**Fig. 19 (abstract P53):**
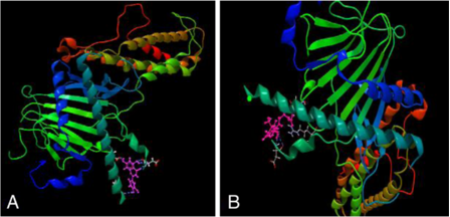
Interaction of **a** Luteolin forming five HBonds with active site of FTO protein and **b** Abscisic acid which formed three HBonds with docking score of -28.636 Kcal/mol

## P54 Computational selection and *in vitro* validation of flavonoids as new antidepressant agents

### Shilu Mathew^1^, Manal Shaabad^1^, Lobna Mira^1^, Shireen Hussain^1^, Mourad Assidi^1,2^, Muhammad Abu-Elmagd^1^, Mohammed Al-Qahtani^1^

#### ^1^Center of Excellence in Genomic Medicine Research, King Abdulaziz University, Jeddah, Saudi Arabia; ^2^Center of Innovation in Personalized Medicine at King Abdulaziz University, Jeddah, Saudi Arabia

##### **Correspondence:** Mourad Assidi (mourad.assidi@gmail.com) – Center of Excellence in Genomic Medicine Research, King Abdulaziz University, Jeddah, Saudi Arabia

**Background**

HTR_1A_ (5-Hydroxytryptamine Receptor 1A) is a protein associated with some diseases mainly depression [1], which is considered as a serious healthcare concern worldwide [2]. HTR1A gene encodes a GPCR for serotonin, which is implicated in several physiologic and pathologic conditions [3]. 5-HT_1A_ receptor is the dominant receptor of HTR1A and found to be responsible for depression and plays a role in the mechanism of action of several antidepressant drugs [4]. Studies indicate that with inactivation of HTR1A in mice resulted in increased stress and anxiety [5].Flavonoids are considered as one of the promising safer alternatives to treat depression [6]. The objective of this study is to screen various flavonoids which could potentially target the 5-HT_1A_R protein using in *silico* docking study. These flavonoids with potential antidepressant effect will be subjected to subsequent *in vitro* validation.

**Materials and methods**

Selected natural anti-depressant compounds known for anti-depressive effects such as (a) hypericin (*Hypericum perforatum*), (b) Saffron (*Crocus sativus*)*,* (c) Omega-3 fatty acid (α-Linolenic acid ), (d) Inositol (Vitamin B8),(e) Kave kave (*Piper methysticum*), (f) Tryptophan (*Tryptophan* synthase), (g) Vitamin B, and (h) Ginkgo (*Ginkgo biloba*) were screened against the active domain sites of residues in 5HT_1A_R using computational structural biology tools CLC drug discovery workbench. To evaluate the inhibitory activity of selected medicinal flavonoids, a quantitative structure-activity relationship (QSAR) was conducted.

**Results**

These studies confirm an inhibitory activity of the recruited medicinal drugs on 5-HT1AR. The docking scores were highest for vitamin B with -15.632Kcal/mol and showed a interactions at active site ALA349 (2), ASP352, and PHE353. Furthermore, selected medicinal drugs such as ginkgo, hypericin and omega-3 fatty acid also formed two H-bond interactions whereas hypericin interacted with active site region at SER45 and SER43 with high affinity.

**Conclusions**

Docking studies of the vitamin B showed that this compound is good molecule which docks well with 5-HT1AR. These results indicated that vitamin B could be one of the potential compound to treat depression, which need further validation, and assessment of their pharmacological activities using *in vitro* and *in vivo* models.

**References**

1. http://www.ncbi.nlm.nih.gov/gene/3350.

2. Ootsuka Y, Blessing WW: **Activation of 5-HT1A receptors in rostral medullary raphe inhibits cutaneous vasoconstriction elicited by cold exposure in rabbits**. *Brain research* 2006, **1073-1074**:252-261.

3. Yu Y, Ramage AG, Koss MC: **Pharmacological studies of 8-OH-DPAT-induced pupillary dilation in anesthetized rats**. *Eur J Pharmacol* 2004, **489**(3):207-213.

4. Prow MR, Martin KF, Heal DJ: **8-OH-DPAT-induced mydriasis in mice: a pharmacological characterisation**. *Eur J Pharmacol* 1996, **317**(1):21-28.

5. http://www.clcbio.com/products/clc-drug-discovery-workbench/.

6. www.zeiss.com/…/60-3-0003_e.pdf.

**Fig. 20 (abstract P54):**
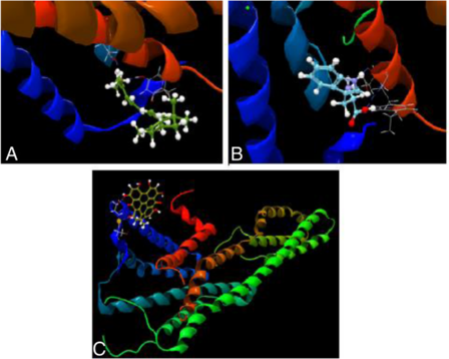
Shows components with high affinities to 5-HT1A **a** Vitamin B, **b** Tryptophan **c** hypericin

## P55 *In Silico* prediction and prioritization of aging candidate genes associated wit progressive telomere shortening

### Ahmed Rebai^1^, Mourad Assidi^2,3^, Abdelbaset Buhmeida^2^, Muhammad Abu-Elmagd^2,3^ Ashraf Dallol^2,3^, Jerry W Shay^2,4^

#### ^1^Bioinformatics Group, Centre of Biotechnology of Sfax, Sfax, Tunisia; ^2^Center of Excellence in Genomic Medicine Research, King Abdulaziz University, Jeddah, Saudi Arabia; ^3^Center of Innovation in Personalized Medicine, King Abdulaziz University, Jeddah, Saudi Arabia; ^4^Department of Cell Biology, The University of Texas Southwestern Medical Center, Dallas, TX, USA

##### **Correspondence:** Ahmed Rebai (ahmed.rebai@cbs.rnrt.tn) – Bioinformatics Group, Centre of Biotechnology of Sfax, Sfax, Tunisia.

**Background**

Very recently a new mechanism of gene regulation, called telomere position effect over long distances (TPE-OLD) has been discovered. As telomeres shorten during normal aging, certain genes nearby telomeres showed altered expression with increased age. It is thought that this off/on phenomenon could explain certain aging-associated diseases. However, only few TPE-OLD regulated genes have been functionally validated. Here we perform a preliminary *in silico* screening for these genes.

**Materials and methods**

Two different *in silico* approaches were used; the first (forward) started from a list of genes which expression is changed during aging, that are available in the GenAge database (http://genomics.senescence.info/genes/) or described in the literature as having their expression affected by telomere shortening. These genes were then filtered based on their proximity to telomere, their involvement in age-related diseases, and other attributes (gene size, signaling and regulation pathways, expression profile, etc.) using a weighted score. A set of 24 genes (with a standardized cut-off score of 0.5) was identified as genes of high interest for further experimental validation. The second (backward) approach consisted of a genome scan of all the genes located within 10 Mb from telomere using a machine-learning based procedure (Bayesian networks).

**Results**

Preliminary results showed modest sensitivity of the approach, which is expected given the reduced size of the training dataset and the non-availability of well characterized distinctive features of this class of genes and a deep understanding of TPE-OLD mechanisms. In fact, the main challenge in the backward approach is to define a training set with ‘positive’ and ‘negative’ genes, that is large enough to ensure high precision of the model learning process. The second challenge in this approach is choosing the gene features that are relevant and highly predictive of the TPE-OLD regulation status.

**Conclusions**

This study is an initial attempt to identify comprehensive and bi-directional approaches to identify aging-associated genes that are affected telomere length changes for subsequent analysis and functional validation. Once validated using larger gene sets, the overlap between these two approaches will help clarify the genes and mechanisms underlying aging-related human diseases.

## P56 Identification of new cancer testis antigen genes in diverse types of malignant human tumour cells

### Mikhlid H Almutairi

#### Zoology Department, College of Sciences, King Saud University, Riyadh, Saudi Arabia

**Background**

Humans possess a class of genes that are normally expressed in the testes of adult males, and are also characteristic of several types of cancer cells [1]. These genes are known as cancer-testis (CT) antigen genes and they might be helpful for both diagnosis and immunotherapy drug targeting [2]. For this reason, identifying new CTA genes has significant clinical importance. We postulated that meiosis-specific genes may provide a good source for identifying potential novel CTA genes. The overall purpose of this investigation was to identify new CTA candidate genes via RT-PCR analysis. A bioinformatic screening program, which included microarray analysis [3] and an expressed sequence tag (EST) analysis pipeline [4], indicated potential meiotic genes which could serve this purpose.

**Materials and methods**

16 and 11 genes were chosen at random from the candidate genes identified via the EST and the Microarray analyses, respectively. The RT-PCR validation employed RNA from 21 normal tissues, including adult testis. The genes that were expressed only in the testis were further examined by RT-PCR using 33 different cancer tissues.

**Results**

*CCNA1, C2orf69, C11orf70, C20orf195, HORMAD1, NOL4, ZNF558, SSX2, UBL4B, GAGE1 and FSCN3* were identified from the gene expression Microarray data analysis pipeline, which predicted they are testis-specific. The expression of these genes was investigated in 21 human normal tissues using RT-PCR technique. *SSX2*, *UBL4B* and *GAGE1* were expressed only in the testis (Table 13), while the remaining 8 genes were expressed in different normal tissues. Therefore, *SSX2*, *UBL4B* and *GAGE1* were further validated in 33 human cancer cell lines and tissues. *SSX2* and *GAGE1* genes were expressed in different types of cancer cells (Table 14).

**Conclusions**

The validation of the 27 genes using RT-PCR analysis determined that four genes showed a cancer testis-restricted expression patterns and four genes displayed meiosis-specific expression patterns. Therefore, these CT genes represent promising candidates due to their expression patterns in distinct types of cancer. However, further investigation is needed to establish the protein products of these excellent CT candidate genes in a range of normal and cancer tissues.

**References:**

1. Whitehurst A. **Cause and Consequence of Cancer/Testis Antigen Activation in Cancer**: *Annual Review of Pharmacology and Toxicology* 2014, **54:**251-272.

2. Caballero O, Chen Y: **Cancer/testis (CT) antigens: potential targets for immunotherapy**. *Cancer Science* 2009, **100(11):**2014-2021.

3. FEICHTINGER J, MCFARLANE R, LARCOMBE L: **CancerMA: a web-based tool for automatic meta-analysis of public cancer microarray data.***Database: The Journal of Biological Databases and Curation* 2012, **2012:** bas055.

4. **Meta‐analysis of expression of l (3) mbt tumor‐associated germline genes supports the model that a soma‐to‐germline transition is a hallmark of human cancers.***International Journal of Cancer* 2014*,***134**: 2359-2365.

**Table 13 (abstract P56):**
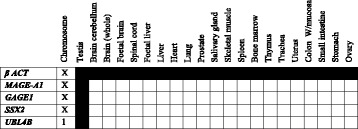
RT-PCR analysis of the mRNA from normal human tissues for *SSX2, UBL4B* and *GAGE1* genes identified from the Microarray analysis

**Table 14 (abstract P56):**
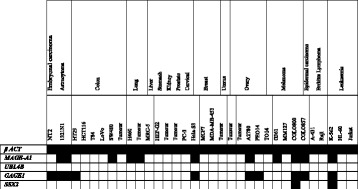
RT-PCR analysis of the mRNA from cancer tissues for *SSX2, UBL4B* and *GAGE1* genes identified from the Microarray analysis

## P57 More comprehensive forensic genetic marker analyses for accurate human remains identification using massively parallel sequencing (MPS)

### Angie Ambers^1^, Jennifer Churchill^1^, Jonathan King^1^, Monika Stoljarova^1,2^, Harrell Gill-King^3^, Mourad Assidi^4^, Muhammad Abu-Elmagd^4^, Abdelbaset Buhmeida^4^, Muhammad Al-Qatani^4^, Bruce Budowle^1,4^

#### ^1^Institute of Applied Genetics, Department of Molecular and Medical Genetics, University of North Texas Health Science Center, Fort Worth, TX 76107, USA; ^2^Institute of Gene Technology, Department of Molecular Diagnostics, Tallinn University of Technology, Akadeemia tee 15A-604, Tallinn 12618, Estonia; ^3^Laboratory of Forensic Anthropology, Center for Human Identification, Department of Biological Sciences, University of North Texas, 1511 West Sycamore, Denton, Texas USA; ^4^Center of Excellence in Genomic Medicine Research, King Abdulaziz University, Jeddah, Saudi Arabia

##### **Correspondence:** Bruce Budowle (bruce.budowle@unthsc.edu) – Center of Excellence in Genomic Medicine Research, King Abdulaziz University, Jeddah, Saudi Arabia

**Background**

Although the primary objective of forensic DNA analyses of unidentified human remains is positive identification, cases involving historical or archaeological skeletal remains often lack reference samples for comparison [1]. Massively parallel sequencing (MPS) offers an opportunity to provide biometric data in such cases, and these cases provide valuable data on the feasibility of applying MPS for characterization of modern forensic casework [2, 3]. In this study, MPS was used to characterize 140-year-old human skeletal remains discovered in a historical site in Deadwood, South Dakota, United States. The remains were discovered in an unmarked grave and there were no records or other meta data to identity of the individual. Due to the high throughput of MPS a variety of biometric markers could be typed using a single sample.

**Results**

Using MPS and suitable forensic genetic markers, more relevant information could be obtained from a limited quantity and quality sample. Results were obtained for 25/26 Y-STRs, 34/34 Y SNPs, 165/165 ancestry-informative SNPs, 28/28 phenotype-informative SNPs, 102/102 human identity SNPs, 27/29 autosomal STRs (plus amelogenin), and 4/8 X-STRs (as well as nine regions of the mitochondrial genome). The Y-chromosome (Y-STR, Y-SNP) and mtDNA profiles of the unidentified skeletal remains are consistent with the R1b and H1 haplogroups, respectively. Both of these haplogroups are the most common haplogroups in Western Europe. Ancestry-informative SNP analysis also supported a European background. The genetic results are consistent with anthropological findings that the remains belong to a male of European ancestry (Caucasian). Phenotype-informative SNP data provided strong support that the individual had light red hair and brown eyes.

**Conclusions**

This study is one of the first to genetically characterize historical human remains with forensic genetic marker kits specifically designed for MPS. The outcome demonstrates that substantially more genetic information can be obtained from the same initial quantities of DNA as that of current-based analyses.

**References**

1. Lorente JA, Entrala C, Alvarez JC, Lorente M, Villanueva E, Carrasco F, Budowle B: **Missing persons identification: genetics at work for society**. *Science* 2000, **290**(5500):2257-2258.

2. Seo SB, King JL, Warshauer DH, Davis CP, Ge J, Budowle B: **Single nucleotide polymorphism typing with massively parallel sequencing for human identification**. *International journal of legal medicine* 2013, **127**(6):1079-1086.

3. Seo S, Zeng X, Assidi M, LaRue B, King J, Sajantila A, Budowle B: **High throughput whole mitochondrial genome sequencing by two platforms of massively parallel sequencing**. *BMC genomics* 2014, **15**(Suppl 2):P7.

## P58 Flow cytometry approach towards treatment men infertility in Saudi Arabia

### Muhammad Abu-Elmagd^1,2^, Farid Ahmed^1^, Ashraf Dallol^1,2^, Mourad Assidi^1,2^, Taha Abo Almagd^3^, Sahar Hakamy^1^, Ashok Agarwal^4^, Muhammad Al-Qahtani^1^, Adel Abuzenadah^1,2^

#### ^1^Center of Excellence in Genomic Medicine Research, King Abdulaziz University Jeddah, Saudi Arabia; ^2^Center of Innovation in Personalized Medicine, King Abdulaziz University, Jeddah, Saudi Arabia; ^3^Urology Department, College of Medicine, King Abdulaziz University, Jeddah, Saudi Arabia; ^4^Center for Reproductive Medicine, Cleveland Clinic, Cleveland, OH, USA

##### **Correspondence:** Muhammad Abu-Elmagd (mabuelmagd@kau.edu.sa) – Center of Innovation in Personalized Medicine, King Abdulaziz University, Jeddah, Saudi Arabia

**Background**

Infertility has becoming an increasing social problem that reached to an alarming level of up to 12 %. In the Middle East countries, a newly married couple usually become very eager to get their first child as soon as they start their marriage life. This is due historic and cultural traditions that the father should get a child who will carry the family’s name and continue the heritage of the family’s tribe. If for some reasons that the pregnancy delayed or did not happen then the newly married couple, especially in the Gulf countries including Saudi Arabia, starts to get pressurized and stressed from other members of their families. Serious social impacts of infertility on the married couple has been recently reviewed. Among these are economic difficulties, improper integration with the rest of the society and family violence. These serious impacts highlight the real need for having real measurements and screening for the causes of the infertility which will directly and positively reflect on the families and the whole society in general. The aim of the current study is to establish a robust molecular assay to assess the quality of the sperms in both fertile and infertile men.

**Subjects and methods**

Ethical approval was granted from the centre of innovation and personalized medicine (CIPM) by the ethical committee. Consent forms were signed by the patients. Four assays are being established including measuring DNA fragmentation using TUNEL assay, mitochondrial membrane potential, sperm vitality using Promidium Iodide and sperm reactive oxygen stress (ROS).

**Results**

**Fig. 21 (abstract P58):**
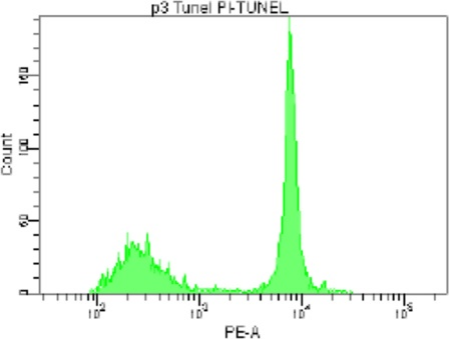
Flow cytometry analysis of patient #3 diagnosis with oligospermia. Sperms were stained with Promidium Iodide and the stain discriminates between two populations of sperms, one as viabe (right peak) and non-viable (left peak)

We have just started assessing the sperm viability of an infertile patient and the flow cytometry analysis showed that the sperms can be categorized to two separate population (viable and noviable) (Fig. 21).

**Conclusions**

Preliminary results showed that using flow cytometry approach shows that this approach could be a used as a robust quick analysis to discriminate between viable and non-viable sperms. Also, the technology could help in perfect selection of quality sperms that could be used in IVF clinics.

**Acknowledgments**

This project is funded by the National Plan for Science, Technology and Innovation (MAARIFAH) – King Abdulaziz City for Science and Technology (KACST) - the Kingdom of Saudi Arabia – Award number (13-CIPM-01).

## P59 Tissue microarray based validation of CyclinD1 expression in renal cell carcinoma of Saudi kidney patients

### Sajjad Karim^1^, Hans-Juergen Schulten^1^, Ahmad J Al Sayyad^2^, Hasan MA Farsi^2^, Jaudah A Al-Maghrabi^3,4^, Abdelbaset Buhmaida^1^, Zeenat Mirza^5^, Reem Alotibi^1^, Alaa Al-Ahmadi^1^, Nuha A Alansari^1^, Alaa A Albogmi^1^, Maha M Al-Quaiti^1^, Fai T Ashgan^1^, Afnan Bandah^1^, Mohammed H Al-Qahtani^1^

#### ^1^Center of Excellence in Genomic Medicine Research, King Abdulaziz University, PO Box 80216, Jeddah 21589, Saudi Arabia; ^2^Department of Urology, Faculty of Medicine, King Abdulaziz University, Jeddah, Saudi Arabia; ^3^Department of Pathology, Faculty of Medicine, King Abdulaziz University, Jeddah, Saudi Arabia; ^4^Department of Pathology, King Faisal Specialist Hospital and Research Center, Jeddah, Saudi Arabia; ^5^King Fahd Medical Research Center, King Abdulaziz University, PO Box 80216, Jeddah 21589, Saudi Arabia

##### **Correspondence:** Sajjad Karim (skarim1@kau.edu.sa) – Center of Excellence in Genomic Medicine Research, King Abdulaziz University, PO Box 80216, Jeddah 21589, Saudi Arabia

**Background**

Tissue microarrays (TMAs) is a new high-throughput tool for the study of protein expression patterns in tissues and are increasingly used to evaluate the diagnostic and prognostic importance of biomarkers. Renal cell carcinoma (RCC) is a seventh ranked malignancy with a poor prognosis (1). The aim of this study was to identify protein signatures that would predict clinical outcomes in a large cohort of patients with RCC based on data from previous gene expression microarray studies (2).

**Materials and methods**

We conducted microarray to identify differentially expressed genes associated with RCC and TMA based immunohistochemical analysis of CyclinD1 (CCND1) to validate our microarray finding over 139 cases of RCC patients. Statistical analysis was used to determine the association of CCND1 expression with RCC and cases were evaluated based on the absence or presence of staining intensity in the tumor cells.

**Results**

The result showed the positive percentage of CCND1 expression in 53 % (73/139) of RCC cases. CCND1 was one of the important upregulated gene identified in microarray and validated by TMA. Studies revealed that it is frequently deregulated in cancer and is a biomarker of cancer phenotype and disease progression (3, 4).

**Conclusions**

Our microarray and TMA based finding confirm the high expression of CCND1 in RCC. It is hoped that CCND1 may be potential therapeutic targets and its inhibition could target the migratory, invasive, and metastatic potential of RCC.

**Acknowledgements**

This project was funded by the National Plan for Science, Technology and Innovation (MAARIFAH) – King Abdulaziz City for Science and Technology, Saudi Arabia – award number (10-BIO1258-03, 10-BIO1073-03 and 08-MED120-03).

**References**

1. Siegel RL, Miller KD, Jemal A: **Cancer statistics**, 2015. *CA Cancer J Clin*. 2015, **66**: 5-29.

2. Mirza Z, Schulten HJ, Farsi HM, Al-Maghrabi JA, Gari M, Chaudhary AGA, Abuzenadah AM, Al-Qahtani MH, Karim S: **Molecular interaction of a kinase inhibitor midostaurin with anticancer drug targets, S100A8 and EGFR: transcriptional profiling and molecular docking study for kidney cancer therapeutics.***PLOS ONE* 2015, **10(3):**e0119765, 1-17.

3. Young AN, Amin MB, Moreno CS, Lim SD, Cohen C, Petros JA, Marshall FF, Neish AS: **Expression profiling of renal epithelial neoplasms: a method for tumor classification and discovery of diagnostic molecular markers.***Am J Pathol* 2001, **158:** 1639-165.

4. Musgrove EA, Caldon CE, Barraclough J, Stone A, Sutherland RL: **Cyclin D as a therapeutic target in cancer.***Nature Reviews Cancer* 2011, **11:**558-572.

## P60 Assessment of gold nanoparticles in molecular diagnostics and DNA damage studies

### Rukhsana Satar^1^, Mahmood Rasool^2^, Waseem Ahmad^2^, Nazia Nazam^3^, Mohamad I Lone^3^, Muhammad I Naseer^2,4^, Mohammad S Jamal^4^, Syed K Zaidi^2^, Peter N Pushparaj^2^, Mohammad A Jafri^2^, Shakeel A Ansari^2^, Mohammed H Alqahtani^2^

#### ^1^Department of Biochemistry, Ibn Sina National College for Medical Sciences, Jeddah-21418, Kingdom of Saudi Arabia; ^2^Center of Excellence in Genomic Medicine Research, King Abdulaziz University, Jeddah-21589, Kingdom of Saudi Arabia; ^3^Toxicogemics Laboratory, Division of Genetics, Department of Zoology, Aligarh Muslim University, Aligarh, India; ^4^King Fahd Medical Research Center, King Abdulaziz University, Jeddah-21589, Kingdom of Saudi Arabia

##### **Correspondence:** Shakeel A Ansari (shakeel.cegmr@gmail.com) – Center of Excellence in Genomic Medicine Research, King Abdulaziz University, Jeddah-21589, Kingdom of Saudi Arabia

**Background**

Since the inception of nanotechnology, noble metal nanoparticles (NPs) have shown greater impact in genomic medicine, cancer studies and targeted drug delivery. They are used in diagnostics and prognostic, DNA and RNA detection, SNP screening for various carcinomas and cardiovascular disorders [1,2]. Several researchers have employed these NPs in describing human sequence variations, multiplex SNP, genetic polymorphism of base excision repair, polymorphism at the level of single gene such as ERGG2 gene (for thyroid cancer risk), CLTA-4 gene (for susceptibility to type 1 diabetes) etc.

**Material and methods**

Gold NPs were synthesized and characterized by transmission electron microscopy (TEM), followed by their toxicity analysis was performed by comet assay. The surface was modified with glutaraldehyde as biocompatible coating for enhancing their potential application in drug delivery for concurrent therapy and other diagnostic based applications.

**Results:**

TEM results showed that the synthesized NPs were of 30 nm. Comet result showed that as a result of surface functionalization of gold NPs by glutaraldehyde, reduction in DNA damage was reduced from 7 μm to 2 μm.

**Conclusions**

These NPs can be used in numerous techniques involving NPs based enhancement in electrochemical DNA hybridization signals, electronucleation and ultra-sensitive electrical detection of nucleic acids with increased specificity and sensitivity apart from multiplexing capability and short turnaround times.

**References**

1. Rasool M, Malik A, Manan A, Ansari SA, Naseer MI, Qazi MH, Asif M, Gan SH, Kamal MA. **Nanoparticle based therapy in genomics**. *Curr Drug Metab* 2015, **16**: 354-361.

2. Mieszawska AJ, Mulder WJM, Fayad ZA, Cormode DP. **Multifunctional gold nanoparticles for diagnosis and therapy of disease**. *Mol Pharm* 2013, **10**: 831-847.

## P61 Surfing the biospecimen management and processing workflow at CEGMR Biobank

### Hanan Bashier, Abrar Al Qahtani, Shilu Mathew, Amal M. Nour, Heba Alkhatabi, Adel M. Abu Zenadah, Abdelbaset Buhmeida, Mourad Assidi, Muhammed Al Qahtani

#### Center of Excellence in Genomic Medicine (CEGMR), King Abdulaziz University, Jeddah, Saudi Arabia

##### **Correspondence:** Mourad Assidi (mourad.assidi@gmail.com) – Center of Excellence in Genomic Medicine (CEGMR), King Abdulaziz University, Jeddah, Saudi Arabia

**Background**

In the post-genomic Era, biobanks are the main core facility where high quality biospecimens with their fully annotated clinico-pathological data are processed according to best Standard Operating Procedures (SOPs) [**1**, **2**][**3**]. Since biospecimen is the main driver towards precision medicine, its management and processing along with their biodata remain therefore the crucial step that will significantly impact their subsequent use in research and/or diagnostics. In this context, the objective of this study is to underline the biospecimen management and processing at the CEGMR Biobank Unit (CBU), CEGMR, King Abdulaziz University, Jeddah, Saudi Arabia; and to suggest future guidelines to upgrade this facility.

**Materials and methods**

A summary of biospecimen transition steps within the CBU (collection, transport, reception, labelling, extraction, storage and release) with a special focus on the main improvement milestones since its establishment in 2008, current challenges and future plans are presented. Additionally, the main achievements of the CBU in terms of the biospecimens’ collection and its role in bridging the gap between clinicians and scientists are discussed.

**Results**

Biospecimen management at the CBU started in 2008 with basic freezing infrastructure and manual recording. A home-made Laboratory Information Management System (LIMS) “Biosearch” was implemented as user-friendly platform for biospecimen biodata entry, follow-up and retrieve [4]. Progressive introduction of SOPs and biospecimen quality control at different steps were implemented. A particular effort was given to staff training and public awareness mainly in healthcare providers explaining significant increase of CBU biospecimen providers over the past few years. Therefore, around 15000 high quality and fully annotated biospecimens (50 % genetic disorders; 40 % of mainly solid cancers) have been collected and part of them supplied to healthcare scientists. More than 150 studies published in ISI journals were carried out using CBU biospecimens.

**Conclusions**

The continual improvement of biospecimen management and processing is vital to keep CBU updated with the rapid evolution in biobanking concept, rules and applications worldwide. Additional efforts are needed to improve the CBU governance and biospecimen management including the introduction of robotic-based cryopreservation, the increase of awareness, and the implementation of the latest international SOPs and rules toward international accreditation.

**Acknowledgements**

Authors would like to thank King Abdulaziz City for Science and Technology (KACST) for their financial support to this study (Grant number: 11-BIO1512-03).

**References**

1. Wichmann E: **Need for guidelines for standardized biobanking**. *Biopreservation and biobanking* 2010, **8**(1):1.

2. Vaught JB, Henderson MK, Compton CC: **Biospecimens and Biorepositories: From Afterthought to Science**. *Cancer epidemiology, biomarkers & prevention : a publication of the American Association for Cancer Research, cosponsored by the American Society of Preventive Oncology* 2012, **21**(2):253-255.

3. Vegvari A, Welinder C, Lindberg H, Fehniger TE, Marko-Varga G: **Biobank resources for future patient care: developments, principles and concepts**. *Journal of clinical bioinformatics* 2011, **1**(1):24.

4. Karim S, Al-Kharraz M, Buhmeida A, Gari M, Chaudhary A, Abuzenadah A, Al-Qahtani M: **BioSearch: an in-house developed lab information management system for center of excellence in genomic medicine research**. *BMC genomics* 2014, **15**(Suppl 2):P41.

**Fig. 22 (abstract P61):**
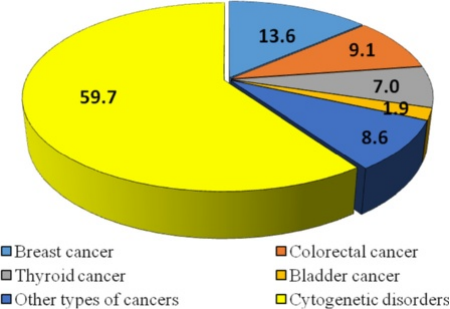
Types of biospecimens collected at CBU (2008-2015) (%)

## P62 Autism Spectrum Disorder: knowledge, attitude and awareness in Jeddah, Kingdom of Saudi Arabia

### Muhammad Faheem^1^, Shilu Mathew^2^, Shiny Mathew^3^, Peter Natesan Pushparaj^2^, Mohammad H. Al-Qahtani^2^

#### ^1^Department of Biochemistry, Faculty of Science, King Abdulaziz University, Jeddah, KSA; ^2^Center of Excellence in Genomic Medicine Research, King Abdulaziz University, Jeddah, KSA; ^3^Department of Multimedia Technology, Karunya University, Coimbatore, India

##### **Correspondence:** Peter Natesan Pushparaj (Peter.n.pushparaj@gmail.com) – Center of Excellence in Genomic Medicine Research, King Abdulaziz University, Jeddah, KSA

**Background**

Autism Spectrum Disorder (ASD) is a neurological disorder that is characterized by an impaired social interaction, communication, restricted, and repetitive behaviors. It starts at the time of birth or within the first three years of life, and is a big challenge not only for the affected child but also for the whole family. About 67-million individuals are suffering with autism worldwide whereas its prevalence ranges from 1.4 to 29 per 10,000 persons in Saudi Arabia [1, 2, 3]. Here, we study the general knowledge, awareness, and attitude of public towards autism patients in Jeddah, KSA.

**Materials and methods**

A survey comprises of thirty questions regarding ASD has been used to collect the data from 300 residents of Jeddah, KSA. Questionnaires were distributed hand-to-hand and through the creation of an online survey.

**Results**

In this study, 300 people, both males and females, have successfully completed the questionnaire. The results have shown that 89 % people were aware of autism, 83 % believed that the autistic child has difficulty in social interaction, and 53 % has linked autism with an emotional or psychological disorder. 30 % predicted that the autism patients do not want friends, 72 % were with a point of view that a person with autism may exhibit ritualistic or repetitive behavior, 67 % were convinced that autism can be cured or children with autism will eventually grow out of it, 70 % were with a thought that autistic patients may have very limited interests (i.e. preoccupation with one toy, movie, game etc). Furthermore, 25 % answered that autism is associated with mental retardation, 59 % think that autism affect the intelligence level, 52 % believed that the child with autism get married in the future. 58 % favored that autism child should attend special school, and 45 % accepted that there is a discrimination in society against the autistic child.

**Conclusions**

In conclusion, there is a need to increase the public awareness about ASD. Several educational awareness programs, seminars, and campaigns are required to build an autism friendly society. It is expected that the society will be more sympathetic and responsible towards ASD patients as a result of this informative study.

**References**

**1**. Dominick KC, Davis NO, Lainhart J, Tager-Flusberg H, Folstein S: **Atypical behaviors in children with autism and children with a history of language impairment**. *Research in developmental disabilities* 2007, **28 (2)**: 145-62.

**2**. Salhia HO, Al-Nasser LA, Taher LS, Al-Khathaami AM, El-Metwally AA: **systemic review of the epidemiology of autism in Arab Gulf countries**. *Neurosciences (Riyadh)* 2014, **19(4)**: 291-6.

**3**. World Health Organization (WHO), Fourth Conference, 2013.

## P63 Simultaneous genetic screening of the coagulation pathway genes using the Thromboscan targeted sequencing panel

### Hani A. Alhadrami^1,2^, Ashraf Dallol^2^, Adel Abuzenadah^1,2^

#### ^1^Faculy of Applied Medical Sciences, King Abdulaziz University, Jeddah, Saudi Arabia; ^2^Centre of Innovation in Personalized Medicine, King Abdulaziz University, Jeddah, Saudi Arabia

##### **Correspondence:** Hani A. Alhadrami (hanialhadrami@kau.edu.sa) – Centre of Innovation in Personalized Medicine, King Abdulaziz University, Jeddah, Saudi Arabia

**Background**

Thrombophilia is a condition where the blood has an increased tendency to clot. It can be acquired or inherited. Inherited thrombophilia is a result of DNA mutation in genes responsible for the production of blood clotting proteins. While inherited thrombophilia can be caused by a number of mutations, the most common ones are factor V Leiden (FVL) and prothrombin (factor II). Factor V Leiden mutation is a single nucleotide point mutation (SNP) located at position number 506 and alters amino acid arginine to glutamine in FV gene. Prothrombin (factor II) is the precursor to thrombin and located on chromosome 11p11-q12. Prothrombin G20210A mutation (factor II mutation) is a SNP located at position 20210 and changes amino acid guanine to adenine in the prothrombin gene. This mutation is associated with high levels of prothrombin and was reported to increase the risk of thrombosis almost three fold. Patients with high levels of other procoagulants such as factors VIII, IX, XI, VII, fibrinogen, and Von Willebrand factor (VWF) are also at high risk of thrombosis.

**Materials and methods**

Next Generation Sequencing allows high throughput DNA sequencing and mutation detection at a low cost and high turnover. This in turns has a major influence in both clinical care and understanding susceptibility to thrombophilia. Therefore, we have designed the Thromboscan panel which will allow the simultaneous screening of 23 coagulation genes using the Ampliseq™ technology.

**Results**

In this study, a screening panel of 23 coagulation genes has been developed, optimized and tested for the early diagnosis of thrombophilia using the cutting edge technology of next generation sequencing. The results confirmed 99.26 % coverage of the targeted genes with 430 amplicons with sizes ranges between 125-275 bp generating 81.56 kb of DNA sequence. We have demonstrated that this panel can be used on DNA extracted from peripheral blood or saliva.

**Conclusions**

The availability of this panel will help increase our understanding of genetic susceptibility to thrombophilia and other aberrant thrombotic events.

## P64 Genome wide array comparative genomic hybridization analysis in patients with syndromic congenital heart defects

### Ibtessam R. Hussein^1^, Adeel G. Chaudhary^1,2^, Rima S Bader^3^, Randa Bassiouni^4^, Maha Alquaiti^1^, Fai Ashgan^1^, Hans Schulten^1^, Mohamed Nabil Alama^5^, Mohammad H. Al Qahtani^1,2,3^

#### ^1^Centre of Excellence in Genomic Medicine Research, King Abdulaziz University, Jeddah, Saudi Arabia; ^2^Faculty of Medical Sciences, King Abdulaziz University, Jeddah, Saudi Arabia; ^3^Pediatric Cardiology Department, King Abdulaziz University, Jeddah, Saudi Arabia; ^4^Children Hospital, Genetics Department, Ministry of Health, Taif, Saudi Arabia; ^5^Cardiology Unit, King Abdulaziz University Hospital, Jeddah, Saudi Arabia

##### **Correspondence:** Ibtessam R. Hussein (irhussein@gmail.com) – Centre of Excellence in Genomic Medicine Research, King Abdulaziz University, Jeddah, Saudi Arabia

**Background**

Congenital heart defects (CHDs) are the most common birth defects leading to increased morbidity and mortality in neonatal life**.** CHDs are usually presented associated with developmental delay (DD) dysmorphic features and/or other congenital malformations**.** Genetic causes such as chromosome anomalies and syndromic CHDs contribute to a small proportion of CHDs, however, a large proportion of cases no genetic diagnosis could be achieved by clinical examination and conventional cytogenetic analysis [1].The development of genome wide array-Comparative Genomic Hybridization technique (array-CGH) allowed for the detection of cryptic chromosomal imbalances and pathogenic CNVs not detected by conventional techniques [2]. We investigated 94 patients having CHDs associated with other malformations and/or DD. Clinical examination and Echocardiography was done to all patients to evaluate the type of CHD and any associated malformations. To investigate for genome defects we applied high density array-CGH 2X400K (33 patients) and CGH/ SNP microarray 2X400K (Agilent) for 25 patients. Confirmation of results was done using Fluorescent in situ Hybridization (FISH) and qPCR techniques.

**Results**

Chromosomal abnormalities such as trisomy 18, 13, 21, 9p and microdeletions: del22q11.2, del7q11.23, del18 (p11.32; p11.21), tetrasomy 18p, and der 9, 15 (q34.2; q11.2) were detected in 15/94 patients (16 %) using conventional cytogenetics methods and array-CGH. Pathogenic variants were detected in 12/58 (20.7 %) samples, CNVs were observed in a large proportion of the studied samples. CGH/SNP array could detect loss of heterozygosity (LOH) in different chromosomal loci in 10/25 patients.

**Conclusions**

Array-CGH technique allowed for detection of cryptic chromosomal imbalances that could not be detected by conventional cytogenetic methods. Clustering of CNVs in certain genome loci needs further analysis to identify causal variants from those of unknown significance, and to identify candidate genes that may provide clues for understanding the molecular pathway of cardiac development. Detection of loci of LOH might reflect regions of homozygosity that can aid in diagnosis of autosomal recessive diseases through selection of candidate genes for sequence analysis.

**Acknowledgements**

The authors would like to thank the King Abdulaziz City for Science and Technology (KACST) for funding this work as part of the project No. (P-L-11-0556).

**References**

1. Meberg A, Hals J, Thaulow E. 2007. **Congenital heart defects, chromosomal anomalies, syndromes and extra cardiac malformations**. *Acta Pediatr*, **96**:1142-5.

2. Breckpot J., Thienpont B, Peeters H, de Ravel T, Singer A, Rayyan M., Allegaert K., et al. 2010. **Array Comparative Genomic Hybridization as a Diagnostic Tool for Syndromic Heart Defects**. *J Pediatr*. **156**:810-7.

## P65 Toxocogenetic evaluation of 1, 2-Dichloroethane in bone marrow, blood and cells of immune system using conventional, molecular and flowcytometric approaches

### Mohammad I Lone^1^, Nazia Nizam^1^, Waseem Ahmad^2^, Mohammad A Jafri^2^, Mahmood Rasool^2^, Shakeel A Ansari^2^, Muhammed H Al-Qahtani^2^

#### ^1^Gene-Tox Laboratory, Division of Genetics, Department of Zoology, Aligarh Muslim University, Aligarh, UP, India; ^2^Center of Excellence in Genomic Medicine Research, King Abdulaziz University, Jeddah, Saudi Arabia

##### **Correspondence:** Mohammad I Lone (iqbalzoo84@gmail.com) – Gene-Tox Laboratory, Division of Genetics, Department of Zoology, Aligarh Muslim University, Aligarh, UP, India

**Background**

Organochlorine pesticides induce extensive genotoxicity but dichloroethane is still being used in industry. The genotoxic profile of this compound has not been clearly identified. Current study evaluated genotoxic potential of dichloroethane using a battery of mutagenicity and genotoxicity assays.

**Materials and methods**

Adult Wistar rats (8 week old, both sexes, 5 rats/dose) were intraperitoneally injected with three doses [10 %, 20 %, 30 % of dichloroethane LD_50_ (807 mg/kg)].The cyclophosphamide was used as positive control. Bone marrow flushes and other cell types were harvested after completion of specified duration. The samples were tested in standard assays for genotoxicity (CA, MNT and MI), DNA damage (comet assay), mitochondrial membrane potential [MMP] estimation and cell cycle alteration analysis by flowcytometry.

**Results**

Dichloroethane treated rats showed a significant increase in micronucleated polychromatic erythrocytes and extensive chromosomal aberrations (CA value of 6.34 ± 1.69 at highest dose of 242.1 mg/kg). The treated rats also displayed high level DNA damage compared to the untreated control group (p < 0.05) as indicated by the value of olive tail moment (19.87 ± 1.4 at dose 242.1 mg/kg after 24 hour) in the comet assay. The flowcytometric analysis following PI staining of dichloroethane exposed cells after 24, 48 and 72 hour showed enhanced apoptosis which increased with the dose and exposure duration. The appearance of SubG1 apoptotic peak in cell cycle was also noticed. The cyclophosphamide treated group (positive control) showed 100 % cell population in the apoptotic phase. The MMP analysis (flowcytometry examination following rhodamine 123 staining) demonstrated a significant decrease in MMP of all WBCs (neutrophils, eosinophils, lymphocytes and monocytes) in the treated groups.

**Conclusions**

The dichloroethane exposure in Wistar rats induced excessive apoptosis in normal cells by direct DNA damage. The DNA damage resulted in decreased mitochondrial membrane potential which in turn altered membrane permeability leading to the release of pro-apoptotic signals and activation of caspase pathway. Thus, driving cells to undergo apoptosis. Therefore, dichloroethane has a potential for inducing severe cell injury and genotoxicity.

## P66 Molecular cytogenetic diagnosis of sexual development disorders in newborn: A case of ambiguous genitalia

### Eradah Alshihri^1^, Muhammad Abu-Elmagd^2,3^, Lina Alharbi^1^, Mourad Assidi^2,3^, Mohammed Al-Qahtani^1,2^

#### ^1^Diagnostic Genomic Medicine Unit, Center of Excellence in Genomic Medicine Research, King Abdulaziz University, Jeddah, Saudi Arabia; ^2^Center of Excellence in Genomic Medicine Research, King Abdulaziz University, Jeddah, Saudi Arabia; ^3^Center of Innovation in Personalized Medicine, King Abdulaziz University, Jeddah, Saudi Arabia

##### **Correspondence:** Muhammad Abu-Elmagd (mabuelmagd@kau.edu.sa) – Center of Innovation in Personalized Medicine, King Abdulaziz University, Jeddah, Saudi Arabia

**Background**

A considerable number of phenotypes relating to disorders of sexual development (DSD) were reported and characterized mainly by atypical development of the external genitalia. This ambiguous genitalia is one of the mixed gonadal dysgenesis that affects 1 in 4,500 of newborns worldwide [1, 2]. The main karyotype associated with this DSD is 45,X/46,XY mosaicism with normal or abnormal Y [3]. The phenotype is affected by the distribution of 45,X cell among the body cells and the Y chromosome aberration. Neonatal diagnosis is critical due to the high risk of developing dysgerminomas and gonadoblastomas. We report here both chromosomal and molecular cytogenetic analysis of a case diagnosed with an ambiguous genitalia in Jeddah, Saudi Arabia. The case has been admitted at King Abdulaziz University Hospital (KAUH) and has been referred to the Diagnostic Genomic Medicine Unit (DGMU) at the Centre of Excellence in Genomic Medicine Research for subsequent molecular and cytogenetic analyses.

**Subjects and methods**

Blood sample was taken from a 21-day newborn diagnosed with clinical indication of ambiguous genitalia and suspect of phenotypic female gender. The case was referred from the Pediatrics Unit at KAUH to the DGMU for molecular and cytogenetic investigations to determine the newborn sex.

**Results**

Based on 50 metaphase stages examined, The karyotype was 46,XY,del(y)(q11.2)[32]/45,X[18]. This male karyotype was marked by the presence of two different cell lines: 64 % of the examined cells showed deletion in the long arm of the chromosome Y at breakpoint q11.2; and 36 % of the cells showed monosomy of chromosome X. Fluorescent in situ hybridization using centromeric probes for chromosomes X and Y and whole chromosome confirmed sex chromosomes and the deleted part of Y chromosome didn’t inserted in another chromosome.

**Conclusions**

The gender of our current case of ambiguous genitalia was male with Y chromosome long arm deletion. This case of DSD shows the importance of the chromosomal and molecular cytognetic analysis for early and accurate determination the genetic gender in order to ensure that patients with similar conditions will receive proper diagnosis, management and both medical and psychological follow up in adolescence and adulthood.

**References**

1. Anderson S: **Disorders of Sexual Differentiation: Ethical Considerations Surrounding Early Cosmetic Genital Surgery**. *Pediatric nursing* 2015, **41**(4):176-186.

2. Telles-Silveira M, Knobloch F, Kater CE: **Management framework paradigms for disorders of sex development**. *Archives of endocrinology and metabolism* 2015.

3. Knudtzon J, Aarskog D: **45,X/46,XY mosaicism. A clinical review and report of ten cases**. *Eur J Pediatr* 1987, **146**(3):266-271.

## P67 Identification of disease specific gene expression clusters and pathways in hepatocellular carcinoma using *In Silico* methodologies

### Shilu Mathew, Peter Pushparaj Natesan, Muhammed Al Qahtani

#### Center of Excellence in Genomic Medicine Research, King Abdulaziz University, Jeddah, KSA

##### **Correspondence:** Peter Pushparaj Natesan (Peter.n.pushparaj@gmail.com) – Center of Excellence in Genomic Medicine Research, King Abdulaziz University, Jeddah, KSA

**Background**

Hepatocellular carcinoma is third most common cause of death and one of the most common cancers worldwide [1, 2]. HCC ascends in cirrhotic livers secondary to numerous environmental condition [3, 4]. However, HCC may also progress in both normal liver, and abnormal non-cirrhotic liver. HCV and HBV infections are one of the major cause resulting in cirrhosis and finally HCC [5]. Each of above conditions comprises altered epigenetic and genetic alterations, differential activation/inhibition of molecular pathways, gene mutations and chromosomal aberrations [3]. This study was done to achieve a comprehensive analysis of gene expression profiling using *in silico* methodologies to identify novel pathways and disease specific gene clusters in HCC.

**Materials and methods**

We have obtained Affymetrix Microarray CEL files from Gene Expression Omnibus (GEO) (GSE49515). The CEL files were then analyzed using Genespring™ GX 13.1 software (Agilent, USA). Statistical analysis was done using unpaired Student’s t-test to obtain differentially expressed genes (P < 0.05) with 2-fold cut-off between normal liver tissue and HCC samples. The differentially expressed genes were then subjected to Hierarchical Clustering to obtain HCC specific gene clusters. Furthermore, we have used Ingenuity Pathway Analysis (IPA) software (Ingenuity Systems, USA) to obtain differentially expressed canonical pathways and novel gene networks in HCC.

**Results**

**Fig. 23 (abstract P67):**
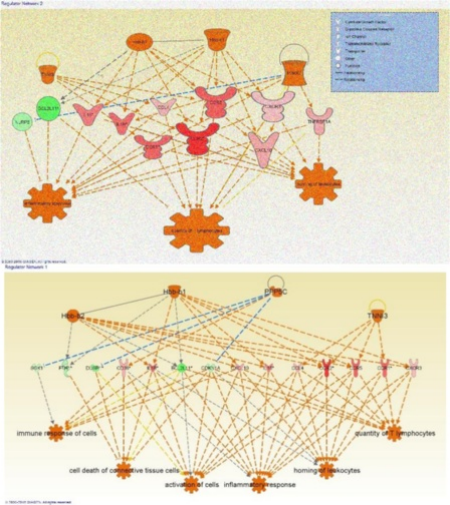
Novel immune and inflammatory gene clusters in HCC

Gene expression profiling between non-tumor tissues and HCC resulted in the identification of deregulated pathways and potential genetic networks in the context of HCC (Fig. 23). Pathway analysis defined evidence that various biological pathways, such as TREM1 Signaling, pathogenesis of multiple sclerosis, communication between innate and adaptive immune cells, toll-like receptor signaling and HER-2 signaling in breast cancer. Few differentially expressed classes of genes in HCC are related to cellular assembly, organization, cell cycle, DNA replication, cellular development, recombination and repair, cell morphology and RNA post-transcriptional modification, post-translational modification, RNA damage and repair, and liver toxicity.

**Conclusions**

Our gene expression profile analysis unraveled a complete cluster of genes and several dysregulated signaling pathways that are different in HCC. These observations may deliver the root for developing novel diagnostic, and prognostic biomarkers of HCC to design effective therapeutics in clinics.

**References**

1. McGlynn KA, London WT: **Epidemiology and natural history of hepatocellular carcinoma.***Best Pract Res Clin Gastroenterol* 2005, 19(1):3-23.

2. Parkin DM, Bray F, Ferlay J, Pisani P: **Global cancer statistics**, 2002. *CA Cancer J Clin* 2005, 55(2):74-108.

3. Lemmer ER, Friedman SL, Llovet JM: **Molecular diagnosis of chronic liver disease and hepatocellular carcinoma: the potential of gene expression profiling.***Semin Liver Dis* 2006, 26(4):373-384.

4. Mathew S, Ali A, Abdel-Hafiz H, Fatima K, Suhail M, Archunan G, Begum N, Jahangir S, Ilyas M, Chaudhary AG *et al*: **Biomarkers for virus-induced hepatocellular carcinoma (HCC)**. *Infect Genet Evol* 2014, 26:327-339.

5. Mathew S, Fatima K, Fatmi MQ, Archunan G, Ilyas M, Begum N, Azhar E, Damanhouri G, Qadri I: **Computational Docking Study of p7 Ion Channel from HCV Genotype 3 and Genotype 4 and Its Interaction with Natural Compounds.***PLoS One* 2015, 10(6):e0126510.

## P68 Human Wharton’s Jelly stem cell conditioned medium inhibits primary ovarian cancer cells *in vitro:* Identification of probable targets and mechanisms using systems biology

### Gauthaman Kalamegam^1^, Peter Natesan Pushparaj^1^, Fazal Khan^2^, Roaa Kadam^1^, Farid Ahmed^1^, Mourad Assidi^1^, Khalid Hussain Wali Sait^3^, Nisreen Anfinan^3^, Mohammed Al Qahtani^1^

#### ^1^Centre of Excellence in Genomic Medicine Research (CEGMR), King Abdulaziz University, Jeddah, Saudi Arabia; ^2^Department of Biochemistry, Faculty of Science, King Abdulaziz University, Jeddah, Kingdom of Saudi Arabia; ^3^Department of Obstetrics and Gynaecology, Faculty of Medicine, King Abdulaziz University, Jeddah, Kingdom of Saudi Arabia

##### **Correspondence:** Gauthaman Kalamegam (kgauthaman@kau.edu.sa) – Centre of Excellence in Genomic Medicine Research (CEGMR), King Abdulaziz University, Jeddah, Saudi Arabia

**Background:**

Most cases of ovarian cancer are only detected at advanced stages which carries poor prognosis and have approximately 30 % 5 year survival rate [1]. Mesenchymal stem cells (MSCs) are identified to exert anti-cancer properties [2,3]. The anticancer properties human Wharton’s jelly stem cells conditioned medium (hWJSCs-CM) on primary ovarian carcinoma cells *in vitro* and also identification of possible novel targets using systems biological approaches was evaluated.

**Materials and methods:**

Primary cultures of epithelial ovarian cancer cells (EOCs) and hWJSCs were derived following Ethical Committee approval [33-15/KAU] and cultured using their recommended media. EOCs were exposed to hWJSC-CM (100 %) for 24 h, 48 h and 72 h. Mophological changes (Phase-contrast imaging) and cell proliferation assay (MTT) were evaluated. Ingenuity Pathway Analysis (IPA, Igenuity Systems, USA) was used to identify targets and mechanisms in ovarian cancer.

**Results:**

**Fig. 24 (abstract P68):**
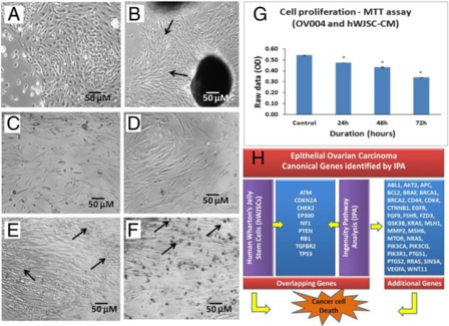
Primary cultures of EOCs and hWJSCs (a, b); hWJSCs induced morphological changes (c-f) and inhibition of cell proliferation (g); and canconical genes in EOCs by IPA (h)

EOCs showed growth as small clusters of epithelial cells with cobblestone appearance (Fig. 24a) while hWJSCs showed a monolayer of short fibroblasts (Fig. 24b). Treatment with hWJSC-CM led to varied morphological changes that resulted in death of EOCs (Fig. 24c-f). Time dependent inhibition in EOCs proliferation (MTT assay) were observed. Mean decreases in proliferations were 12.23 %, 19.71 % and 37.07 % at 24 h, 48 h and 72 h respectively compared to untreated control (Fig. 24g). IPA of EOC genes implicated in canonical pathways led to the identification of important molecular pathways and signaling networks associated with cancer cell death (Fig. 24h). Earlier transcriptome analysis of hWJSCs showed high expression of tumour suppressors and apoptosis inducing genes, which significantly overlap with IPA predictive results. Additional targets and mechanisms of EOCs death/inhibition identified using IPA needs validation by further *in vitro/in vivo* studies.

**Conclusions:**

hWJSC-CM induce primary EOCs inhibition *in vitro* and cause cell death probably via apoptosis. Our findings are in line with earlier reports of cancer inhibition by various other MSCs [2,3]. IPA predictive results indicating the genes/targets invovled in EOCs that overlap with hWJSCs tumour suppressors further support our findings. Additional *in vitro* and *in vivo* studies are necessary to ascertain EOCs inhibition with hWJSC-extracts and identify its possible mechaisms.

**Acknowledgements:**

The financial support provided by King Abdulaziz City for Science and Technology (KACST) grant [AT-34-330] is greatly acknowledged.

**References:**

1. Shuo Chen, Jin-Wen Jiao, Kai-Xuan Sun, Zhi-Hong Zong and Yang Zhao: **MicroRNA-133b targets glutathione S-transferase π expression to increase ovarian cancer cell sensitivity to chemotherapy drugs.***Drug Des Devel Ther*, 2015, **9**: 5225–5235

2. Gauthaman Kalamegam, Fong Chui-Yee, Suganya Cheyyatraivendran, Biswas Arijit, Choolani Mahesh, Bongso Ariff: **Human umbilical cord Wharton’s jelly stem cell (hWJSC) extracts inhibit cancer cell growth in vitro**. *J Cell Biochem* 2012, **113**:2027–2039.

3. Ayuzawa R, Doi C, Rachakatla RS, Pyle MM, Maurya DK, Troyer D, Tamura M: **Naıve human umbilical cord matrix derived stem cells significantly attenuate growth of human breast cancer cells in vitro and in vivo**. *Cancer Lett* 2009, **280**:31–37.

## P69 Mutation spectrum of ASPM (Abnormal Spindle-like, Microcephaly-associated) gene in Saudi Arabian population

### Muhammad I Naseer^1^, Adeel G Chaudhary^1^, Mohammad S Jamal^2^, Shilu Mathew^1^, Lobna S Mira^1^, Peter N Pushparaj^1^, Shakeel A Ansari^1^, Mahmood Rasool^1^, Mohammed H AlQahtani^1^

#### ^1^Center of Excellence in Genomic Medicine Research, King Abdulaziz University, Jeddah, Saudi Arabia; ^2^King Fahd Medical Research Center, King Abdulaziz University, Jeddah, Saudi Arabia

##### **Correspondence:** Mahmood Rasool (mahmoodrasool@yahoo.com) – Center of Excellence in Genomic Medicine Research, King Abdulaziz University, Jeddah, Saudi Arabia

**Background**

Autosomal recessive primary microcephaly (MCPH) is a neurodevelopmental disorder that is characterized by smaller head circumference present at birth with mental retardation [1]. Abnormal Spindle-like, Microcephaly-associated (ASPM) gene is responsible for majority of the primary microcephaly patients. Here, we aim to establish mutational spectrum of ASPM gene in Saudi population.

**Materials and methods**

We have ascertained 50 patient samples with microcephaly. Detailed clinical information was taken from each patient. Fluorescent labeled Microsatellite markers are used to link the ASPM gene using Gene Scan method. Massive sequencing using Sanger sequencing method was carried out to identify the mutations involved in the ASPM gene.

**Results**

The sequencing of ASPM gene from patient samples has revealed number of known and novel mutations associated with primary microcephaly and mental retardation.

**Conclusions**

Once the genetic basis of primary microcephaly is known in Saudi population, it will help in the provision of molecular diagnosis and genetic counseling that may help to decrease the frequency of this disorder.

**Acknowledgements**

This project was funded by the KACST (King Abdulaziz City for Science and Technology) - the Kingdom of Saudi Arabia – large grant project number (APR-34-13).

**References**

1. Faheem M, Naseer MI, Rasool M, Chaudhary AG, Kumosani TA, Ilyas AM, Pushparaj PN, Ahmed F, Algahtani HA, Al-Qahtani MH. **Molecular genetics of human primary microcephaly: An overview**. BMC Med Genomics 2015, **8** Suppl 1:S4.

## P70 Identification and characterization of novel genes and mutations of primary microcephaly in Saudi Arabian population

### Muhammad I Naseer^1^, Adeel G Chaudhary^1^, Shilu Mathew^1^, Lobna S Mira^1^, Mohammad S Jamal^1^, Sameera Sogaty^1^, Randa I Bassiouni^1^, Mahmood Rasool^1,2,3,4^, Mohammed H AlQahtani^1^

#### ^1^Center of Excellence in Genomic Medicine Research, King Abdulaziz University, Jeddah, Saudi Arabia; ^2^King Fahd Medical Research Center, King Abdulaziz University, Jeddah, Saudi Arabia; ^3^Department of Medical Genetics, King Fahad General Hospital, Jeddah, Saudi Arabia; ^4^Children Hospital, Taif, Saudi Arabia

##### **Correspondence:** Mahmood Rasool (mahmoodrasool@yahoo.com) – Center of Excellence in Genomic Medicine Research, King Abdulaziz University, Jeddah, Saudi Arabia

**Background**

Primary microcephaly is a genetic disorder characterized by reduced head circumference that is at least 4 standard deviation (SD), accompanied with non-progress mental retardation. The brain is architecturally normal but the size of cerebral cortex is significantly reduced causing the microcephaly. In this research project we aim to identify the mutated genes behind the primary microcephaly patients in the Saudi population.

**Materials and methods**

We have ascertained 10 consanguineous families with primary microcephaly from Jeddah, Makkah and Taif cities of Saudi Arabia. Detail clinical information and Pedigree (family tree) of the families were analyzed. DNA was extracted from peripheral blood of affected and normal individuals of the families. Fluorescent labeled microsatellite markers were used to link the known genes previously identified with the disease. Sanger sequencing method was used to identify the known and novel genes.

**Results**

Our linkage analysis results showed that four out of ten families were linked with the ASPM gene and three families were linked with MCPH1 gene. Three families were excluded from the previously known genes associated with primary microcephaly. Furthermore we have done the sequencing of ASPM and MCPH1 gene that has revealed novel and known mutations.

**Conclusions**

Identification of exact genotype phenotype correlation would help in genetic counseling and prenatal diagnosis for primary microcephaly and would enable us to reduce the incidence of microcephaly in a highly consanguineous population of Saudi Arabia.

**References**

1. Faheem M, Naseer MI, Rasool M, Chaudhary AG, Kumosani TA, Ilyas AM, Pushparaj PN, Ahmed F, Algahtani HA, Al-Qahtani MH. **Molecular genetics of human primary microcephaly: An overview**. *BMC Med Genomics* 2015, **8** Suppl 1:S4.

**Acknowledgements**

This project was funded by the King Abdulaziz City for Science and Technology (KACST), Riyadh, Kingdom of Saudi Arabia, under grant no. (APR-34-13). The authors therefore acknowledge with thanks KACST technical and financial support.

## P71 Molecular genetic analysis of hereditary nonpolyposis colorectal cancer (Lynch Syndrome) in Saudi Arabian population

### Mahmood Rasool^1^, Shakeel A Ansari^1^, Mohammad S Jamal^2^, Peter N Pushparaj^1^, Abdulrahman MS Sibiani^3^, Waseem Ahmad^1^, Abdelbaset Buhmeida^1^, Mohammad A Jafri^1^, Mohiuddin K Warsi^4^, Muhammad I Naseer^1^, Mohammed H Al-Qahtani^1^

#### ^1^Center of Excellence in Genomic Medicine Research, King Abdulaziz University, Jeddah, Saudi Arabia; ^2^King Fahd Medical Research Center, King Abdulaziz University, Jeddah, Saudi Arabia; ^3^King Abdulaziz University Hospital, Jeddah, Saudi Arabia; ^4^Mohammad Ali Jauhar University, Rampur, UP, India

##### **Correspondence:** Mahmood Rasool (mahmoodrasool@yahoo.com) – Center of Excellence in Genomic Medicine Research, King Abdulaziz University, Jeddah, Saudi Arabia

**Background**

Hereditary non-polyposis colorectal cancer (HNPCC), also referred to as Lynch syndrome, represents 1-7 % of all cases of colorectal cancer and is characterized by autosomal dominant inheritance caused by germline mutations in many DNA mismatch repair genes [1,2]. The aim of current research involves the molecular characterization of Lynch syndrome to provide predictive information of greater accuracy regarding the risks of colon and extracolonic cancer in Saudi population.

**Materials and methods**

Detailed clinical information was taken from patients suffering from lynch syndrome. Amsterdam criteria and Bethesda guidelines were used to confirm the lynch syndrome. Immunohistochemistry, microsatellite instablitity and testing of mismatch repair (MMR) genes performed to diagnose the disease.

**Results**

On first stage we have established state of art facility for the efficient diagnosis of HNPCC in families of Saudi Arabian origin at risk and to find the novel genetic factors associated with it by using cutting edge technology. So far 20 samples of hereditary colorectal cancer involving two or more patients in a family have been collected. The sequencing of mismatch repair genes carried out to find the mutation spectrum in the population and exclude the families for possible novel genes involving lynch syndrome.

**Conclusions**

Once the full spectrum of MMR gene mutations is known in Saudi population, it will help in screening and intensive surveillance or some other measures such as hysterectomy and colectomy that will reduce the risk for colorectal cancer development. This will result in better quality medical facility for counselling and diagnosis as well as reduce the health expenditures to the Saudi families affected with lynch syndrome.

**Acknowledgements**

This project was funded by the National Plan for Science, Technology and Innovation (MAARIFAH) – King Abdulaziz City for Science and Technology - the Kingdom of Saudi Arabia – award number (12-MED3078-03).

**References**

1. Lynch HT, Smyrk TC, Watson P, et al: **Genetics, natural history, tumor spectrum, and pathology of hereditary nonpolyposis colorectal cancer: an update review**. Gastroenterology 1993; **104**(5): 1535-49.

2. Marra G, Boland CR: **Hereditary nonpolyposis colorectal cancer: the syndrome, the genes, and historical perspectives**. J Natl Cancer Inst. 1995, **87** (15): 1114-25.

## P72 Function predication of hypothetical proteins from genome database of chlamydia trachomatis

### Rubi^1^, Kundan Kumar^1^, Ahmad AT Naqvi^2^, Faizan Ahmad^1^, Md I Hassan^1^, Mohammad S Jamal^3^, Mahmood Rasool^4^, Mohammed H AlQahtani^4^

#### ^1^Center for Interdisciplinary Research in Basic Sciences, Jamia Millia Islamia, Jamia Nagar, New Delhi, India; ^2^Department of Computer Science, Jamia Millia Islamia, Jamia Nagar, New Delhi, India; ^3^King Fahd Medical Research Center, King Abdulaziz University, Jeddah, Saudi Arabia; ^4^Center of Excellence in Genomic Medicine Research, King Abdulaziz University, Jeddah, Saudi Arabia

##### **Correspondence:** Md I Hassan (imtiyaz.hassan@gmail.com) – Center for Interdisciplinary Research in Basic Sciences, Jamia Millia Islamia, Jamia Nagar, New Delhi, India

**Abstract**

**Background**

Chlamydia trachomatis strain D/UW-3/Cx is a Gram-negative intracellular bacterium which belongs to bacterial family Chlamydiaceae [1]. C. trachomatis causes sexually transmitted disease Chlamydia as well as blinding trachoma [2].

**Materials and methods**

In our study, we used a number of bioinformatics tools to predict the functions of HPs of C. trachomatis. A combination of latest protein family databases, pathway and genome databases are available to assign an appropriate function to HPs whose sequence is available. We also performed sub-cellular localization and signal peptide prediction.

**Results**

After analysis of the proteome data of C. trachomatis strain D/UW-3/Cx, it was found that ~30 % (272) proteome is listed as conserved hypothetical protein (HP). Extensive analysis of all 272 HPs resulted in the putative function prediction of 60 HPs with high precision. We further categorized HPs on the basis of predicted functions as enzymes, transporters, binding proteins, biosynthesis proteins, type III secretion system effectors, and proteins with miscellaneous functions.

**Conclusions**

The outcome of this study will be helpful in studying the mechanism of pathogenesis of C. trachomatis to identify the potential drug targets, thus helping in the discovery of effective drug against the pathogen.

**References**

1. Horn M. **Chlamydiae as symbionts in eukaryotes**. *Annu. Rev. Microbiol.* 2008, **62**:113–131.

2. Handsfield HH. **Questioning azithromycin for chlamydial infection**. *Sex Transm Dis.* 2011, **38**:1028-9.

## P73 Transcription factors as novel molecular targets for skin cancer

### Ashraf Ali^1^, Jummanah Jarullah^1^, Mahmood Rasool^2^, Abdelbasit Buhmeida^2^, Shahida Khan^1^, Ghufrana Abdussami^3^, Maryam Mahfooz^3^, Mohammad A Kamal^1^, Ghazi A Damanhouri^1^, Mohammad S Jamal^1^

#### ^1^King Fahd Medical Research Center, King Abdulaziz University, Jeddah, Saudi Arabia; ^2^Center of Excellence in Genomic Medicine Research, King Abdulaziz University, Jeddah, Saudi Arabia; ^3^Jamia Millia Islamia, New Delhi, India

##### **Correspondence:** Mohammad S Jamal (sarwar4u@gmail.com) – King Fahd Medical Research Center, King Abdulaziz University, Jeddah, Saudi Arabia

**Background**

Melanoma is often considered one of the most aggressive and treatment-resistant human cancers. Presence of melanin pigment makes it easier to detect than other malignancies, and so it has been subjected to countless therapies. Approximately one in each three patient with cutaneous melanoma develops metastatic stage with poor rate of survival. Current conventional methods used for melanoma detection are Breslow thickness, Clark level invasion and ulceration, but they cannot perfectly predict the melanoma at individual level [1]. Breakthrough in fundamental understanding of molecular basis of disease by new techniques has increased survival rate of melanoma patients.

**Materials and methods**

PubMed database was searched for research articles, reviews and case reports related to skin cancer or melanoma. Other resources used for getting proper information are MEDLINE, EMBASE, Cochrane, Database of Systematic Reviews, Cochrane Central Register of Controlled Trials, ISI Web of Science Proceedings, ISI Web of Science Citation Index, CINAHL, TOXLINE, PUBMED, and Scopus. Pre-specified search terms and keywords like melanoma, skin cancer, transcription factors in melanoma, signaling pathways involved in melanoma and therapeutic options in melanoma etc, were used to identify information regarding randomized clinical trials, nonrandomized intervention studies, and observational studies. A list of transcription factors were prepared and explained accordingly. Citations from 1990 to 2015 were used to generate data and assemble the article.

**Results**

A number of transcription factors are found to be overactive in most of human cancers which are ideal target for anticancer drug development [2]. Several transcription factors like GATA-1, NF-kB, AP1, Nrf2, STAT, Cox, C-Jun and Src and pathways like MAPK play significant role in melanoma development and prognosis [3, 4]. Discoveries of frequent mutations involving several transcription factors can make the pathway easier to understand, and can help in making therapies for their effective treatment.

**Conclusions**

These transcription factors are immediate and potential targets for treating cancers. This study elaborates the role of current list of transcription factors and signaling molecules in melanoma prognosis and treatment.

**References**

1. Federman DG, Concato J, Kirsner RS. Screening **for skin cancer: absence of evidence**. 2009, *Arch Dermatol,***145**: 926.

2. Brach MA, Kauer M, Herrmann F. **Contribution of transcription factors to oncogenesis**. 1996, *Cytokines Mol Ther,***2**: 81–7.

3. Jin JY, Ke H, Hall RP *et al.***c-Jun promotes whereas JunB inhibits epidermal neoplasia**. 2011, *J Invest Dermatol,***131**: 1149–58.

4. Rorke EA, Adhikary G, Jans R *et al.***AP1 factor inactivation in the suprabasal epidermis causes increased epidermal hyperproliferation and hyperkeratosis but reduced carcinogen-dependent tumor formation**. 2010, *Oncogene,***29**: 5873–82.

## P74 An *In Silico* analysis of Plumbagin binding to apoptosis executioner: Caspase-3 and Caspase-7

### Bushra Jarullah^1^, Jummanah Jarullah^2^, Mohammad SS Jarullah^3^, Ashraf Ali^2^, Mahmood Rasool^4^, Mohammad S Jamal^2^

#### ^1^Department of Biotechnology, Kadi Sarva Vishwavidhyalaya, Gandhinagar, Gujarat, India; ^2^King Fahd Medical Research Center, King Abdulaziz University, Jeddah, Saudi Arabia; ^3^Clinical Biochemistry Department, College of Medicine, King Abdulaziz University, Jeddah, Saudi Arabia; ^4^Center of Excellence in Genomic Medicine Research, King Abdulaziz University, Jeddah, Saudi Arabia

##### **Correspondence:** Mohammad S Jamal (sarwar4u@gmail.com) – King Fahd Medical Research Center, King Abdulaziz University, Jeddah, Saudi Arabia

**Background**

Plumbagin (PL) is derived from the root of *Plumbago indica*, has various medicinal properties and reported to induce the apoptosis by activating caspases [1]. Caspase-3 and Caspase-7 has been driving force behind execution of apoptosis. *In vitro* and *In vivo* studies has shown PL activating effect on caspases for inducing apoptosis [2] but the structural insight into the binding mechanism of PL mediated caspases actiation is not defined yet. Here, for the first time we have used *In Silico* studies using docking approach to reveal the molecular insight into the binding of PL with Caspase-3 and Caspase-7.

**Materials and methods**

Docking calculations were carried out on *Plumbagin from Plumbago indica* protein model using AutoDock and Autogrid programs. Each docking experiment was derived from 100 different runs that were set to terminate after a maximum of 250000 energy evaluations. The population size was set to 150.

**Results**

The study demonstrate binding mode of Plumbagin to caspase-3 and caspase-7 using docking and simulations analysis. Our results affirm the role of various important residues 251-SER, 252-PHE, 253-ASP, 256-PHE of caspase-3 and 159-ILE, 211-TYR, 213-ILE, 214-PRO, 221-PHE, 223-TYR, 292-VAL of caspase-7. We found very negative value for dock score and binding energy was also very close to well known inhibitors.

**Conclusions**

For the very first time we have shown the binding mechanism of Plumabgin to apoptosis executioner using *In Silico* approach. In this report we found that total number of four and seven critical residues of Caspase-3 and Caspase-7 respectively play very important role in binding to Plumbagin.

**Acknowledgements**

We thank Deanship of Scientific Research (DSR), King Abdulaziz University for funding grant number- DSR (1434-141-456).

**References**

1. Subramaniya BR, Srinivasan G, Sadullah SS, Davis N, Subhadara LB, Halagowder D, Sivasitambaram ND. **Apoptosis inducing effect of plumbagin on colonic cancer cells depends on expression of COX-2**. *PLoS One*. 2011 Apr 29; **6**(4):e18695. doi:10.1371/journal.pone.0018695.

2. Seshadri P, Rajaram A, Rajaram R**.** Plumbagin and juglone induce caspase-3-dependent apoptosis involving the mitochondria through ROS generation in human peripheral blood lymphocytes. *Free Radic Biol Med*. 2011 Dec **1**; 51(11):2090-107.

## P75 Single cell genomics applications for preimplantation genetic screening optimization: Comparative analysis of whole genome amplification technologies

### Mourad Assidi^1,2^, Muhammad Abu-Elmagd^1,2^, Osama Bajouh^2,3^, Peter Natesan Pushparaj^1^, Mohammed Al-Qahtani^1^, Adel Abuzenadah^1,2,4^

#### ^1^Center of Excellence in Genomic Medicine Research, King Abdulaziz University, Jeddah, Saudi Arabia; ^2^Center of Innovation in Personalized Medicine, King Abdulaziz University, Jeddah, Saudi Arabia; ^3^Obstetrics and Gynecology Department, King AbdulAziz University Hospital, Jeddah, Saudi Arabia; ^4^Faculty of Applied Medical Sciences, King Abdulaziz University, Jeddah, Saudi Arabia

##### **Correspondence:** Mourad Assidi (mourad.assidi@gmail.com) – Center of Innovation in Personalized Medicine, King Abdulaziz University, Jeddah, Saudi Arabia

**Background**

The emerging of single cell genomics (SCGs) has revolutionized the OMICs scale approaches to target extremely small amounts of biomolecules at the single cell level. Such powerful screening advanced approach has permitted comprehensive analysis of molecular heterogeneity within tissues, and the development of pioneering applications in experimental, clinical and forensic applications [1]. Preimplantation Genetic Screening (PGS) for aneuploidy is one of the main SCGs clinical applications used as an adjunct in IVF clinics to select embryos prior their transfer. PGS is offered to patients with advanced maternal age, repeated implantation failure, recurrent pregnancy loss, and infertility [2, 3]. Given the limitation of starting material, single cell whole genome amplification (scWGA) is a crucial step in PGS that needs to be carefully optimized in order to get an amplified DNA with high affinity and reproducibility [4]. The aim of this study is to optimize PGS efficiency through the selection of the best scWGA among three commercial kits allowing thus an efficient detection of copy-number variations (CNVs) and translocations with high reproducibility.

**Materials and methods**

**Fig. 25 (abstract P75):**
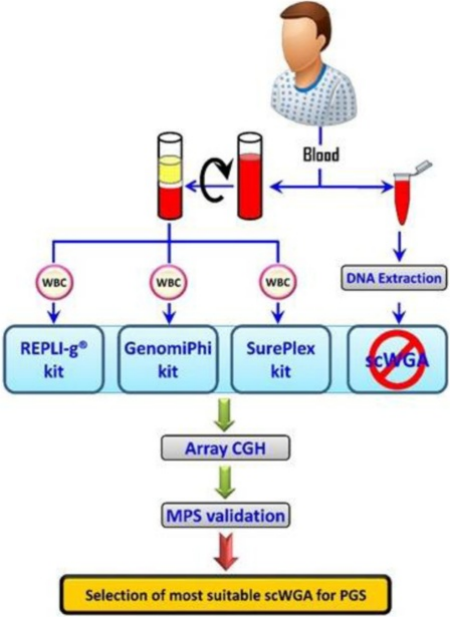
Study flowchart to select most suitable scWGA commercial kit for PGS using an unamplified DNA as a reference. (WBC): single white blood cell; (Array CGH): array Comparative Genomic Hybridization; (MPS): Massively Parallel Sequencing

Two scWGA kits based on multiple displacement amplification (MDA): REPLI-g® Single Cell (Qiagen) and Single Cell GenomiPhi DNA Amplification (GE Healthcare) were compared to the PCR-based SurePlex DNA Amplification System (Blue Genome) using a single white blood cell (WBC) from the same patient as a template and unamplified genomic DNA as a control (Fig. 25). All three methods were compared using array comparative genomic hybridization (aCGH) to detect potential differences in amplification uniformity and/or errors, CNVs, and detection of structural aberrations larger than 10 Mbs.

**Results**

All three scWGA methods provided satisfactory DNA yields when starting from single cell (≥3 μg). Following aCGH data analysis, selected scWGA methods had similar genome coverage and representativity compared to the unamplified reference (P > 0.05). Preliminary evidence showed better CNVs detection with SurePlex kit.

**Conclusions**

This study showed that SurePlex DNA Amplification System is more suitable for PGS to ensure a pregnancy-free disease but further validation using massively parallel sequencing (MPS) is needed. Since most scWGA have some imperfections, a better selection of the scWGA that fits best with both the clinical application and the target genomic variation is recommended.

**Acknowledgements**

This project was funded by the National Plan for Science, Technology and Innovation (MAARIFAH) – King Abdulaziz City for Science and Technology, Saudi Arabia – Award number (APR-34-210).

**References**

1. Macaulay IC, Voet T: **Single Cell Genomics: Advances and Future Perspectives**. *PLoS genetics* 2014, **10**(1):e1004126.

2. Van der Aa N, Zamani Esteki M, Vermeesch JR, Voet T: **Preimplantation genetic diagnosis guided by single-cell genomics**. *Genome medicine* 2013, **5**(8):71.

3. Harper JC, Sengupta SB: **Preimplantation genetic diagnosis: state of the art 2011**. *Hum Genet* 2012, **131**(2):175-186.

4. Munne S: **Preimplantation genetic diagnosis for aneuploidy and translocations using array comparative genomic hybridization**. *Current genomics* 2012, **13**(6):463-470.

## P76 ZFP36 regulates miRs-34a in anti-IgM triggered immature B cells

### Mohammad S Jamal^1^, Jummanah Jarullah^1^, Abdulah EA Mathkoor^1^, Hashim MA Alsalmi^1^, Anas MM Oun^2^, Ghazi A Damanhauri^1^, Mahmood Rasool^3^, Mohammed H AlQahtani^3^

#### ^1^King Fahd Medical Research Center, King Abdulaziz University, Jeddah, Saudi Arabia; ^2^Applied Medical Science Laboratory Department, King Abdulaziz University, Jeddah, Saudi Arabia; ^3^Center of Excellence in Genomic Medicine Research, King Abdulaziz University, Jeddah, Saudi Arabia

##### **Correspondence:** Mohammad S Jamal (sarwar4u@gmail.com) – King Fahd Medical Research Center, King Abdulaziz University, Jeddah, Saudi Arabia

**Background**

In our previous report we tried to understand the mechanism of G1 cell cycle arrest upon stimulation of immature B-lymphocytes through B cell receptors in which gene expression and Ingenuity Pathway Analysis drew our attention that ZFP36 is an important hub among the various genes identified. Because it recruits various kinds of miRNAs (miRs) by binding itself at the 3′ AU-rich regions of various mRNA it is called as RNA Binding protein and miRNA recruiter [1]. In this study we decipher the ZFP36 recruited miRs regulating anti-IgM triggered Immature B cell G1 cell cycle arrest.

**Materials and methods**

CH1 cell were used for study. Literature survey was carried out; we found the list of miRs involved in regulation of immature B cell cycle arrest [2]. So we employed the ZFP36 siRNA system from Santa cruz in anti-IgM triggered immature B cells followed by real-time analysis against listed miRs.

**Results**

Our real-time analysis suggests that upon ZFP36 silencing in immature CH1 cells triggered with anti-IgM miR-34a which was down-regulated in normal BCR triggered CH-1 Cells, shows slight increase in expression at RNA level.

**Conclusions**

Our results suggest that in normal triggered CH1 cells ZFP36 leads to down regulation of miRs-34a may be downregulating some protein at RNA level which might be directly indirectly involved in regulation of miRs.

**Acknowledgements**

We thank Deanship of Scientific Research (DSR), King Abdulaziz University for funding grant number- DSR (1434-141-456**).**

**References**

1. Sanduja S, Blanco FF, Dixon DA. **The roles of TTP and BRF proteins in regulated mRNA decay.** Wiley Interdiscip Rev RNA. 2011, **2**(1):42-57.

2. Kluiver JL^1^, Chen CZ. **MicroRNAs regulate B-cell receptor signaling-induced apoptosis. Genes Immun.** 2012, **13**(3): 239-44.

## P77 Identification of a novel mutation in the *STAMBP* gene in a family with microcephaly-capillary malformation syndrome

### Muhammad I. Naseer^1^, Mahmood Rasool^1^, Sameera Sogaty^2^, Adeel G. Chudhary^1^, Yousif A. Abutalib^3^, Daniele Merico^4^, Susan Walker^4^, Christian R. Marshall^4^, Mehdi Zarrei^4^, Stephen W. Scherer^1,4,5^, Mohammad H. Al-Qahtani^1^

#### ^1^Center of Excellence in Genomic Medicine Research, King Abdulaziz University, 21589, Jeddah, Kingdom of Saudi Arabia; ^2^Department of Medical Genetics, King Fahad General Hospital, Jeddah 21196, Saudi Arabia; ^3^Department of Pediatric Neurologist. Maternity and Children Hospital Jeddah, Saudi Arabia; ^4^The Centre for Applied Genomics, The Hospital for Sick Children, Toronto, Ontario M5G 1 L7, Canada; ^5^McLaughlin Centre and Department of Molecular Genetics, University of Toronto, Toronto, Ontario M5G 1 L7, Canada

##### **Correspondence:** Muhammad I. Naseer (mimrannaseer@kau.edu.sa) – Center of Excellence in Genomic Medicine Research, King Abdulaziz University, 21589, Jeddah, Kingdom of Saudi Arabia

**Background**

Microcephaly-capillary malformation syndrome (MIC-CAP syndrome; OMIM#614261) is an extremely rare disorder of the central nervous system disorder that is characterized by microcephaly, developmental delay, generalized cutaneous capillary malformation, and seizures. This syndrome has been reported in patients, both male and female, born to unrelated or consanguineous parents of various ethnicities, including Arabs. Exonic and intronic mutations (including missense mutations) in the STAM-binding protein (*STAMBP*) gene are well-established causes of this syndrome in dozens of patients. This gene encodes deubiquitinating isopeptidase, which has a key role in cell surface receptor-mediated endocytosis and sorting.

**Materials and methods**

We present two affected male children from a consanguineous family having developmental delay and seizures. We performed exome sequencing on one of the siblings and both parents on the Illumina HiSeq 2500. Base calling was performed using CASAVA v1.8.2 and reads were mapped to the hg19 reference sequence using the BWA-backtrack algorithm from BWA v0.5.9. Variant calling was performed using GATK 1.1-28. Variants were classified into loss of functions and missense. The rare variants were defined as those at < 5 % frequency in 1000 Genomes, NHBLI Exome Sequencing Project, Exome Aggregation Consortium and one in-house databases.

**Results**

In the proband, we found a homozygous missense single-nucleotide variant in exon 7 of the *STAMBP* gene. With Sanger sequencing, we found the same homozygous mutation in the affected sibling. Both parents are heterozygous at this position. The A > G substitution (c.A908G) results in an amino acid change of lysine to arginine (p.K303R). This exonic mutation (chr2:74077543:A:G) has not previously been reported in the *STAMBP* gene, therefore it constitutes a novel mutation, presumed to be disease-causing. We further interrogated the genome of one of siblings for copy number variation, using the Affymetrix CytoScan HD platform, and did not find any potentially pathogenic CNVs.

**Conclusions**

Our results showed a homozygous missense single-nucleotide variant in exon 7 of the *STAMBP* gene and no other phenotype-relevant mutations were found, we attribute the cause of the syndrome to the recessive mutations in *STAMBP*.

**Acknowledgement**

This project was funded by the King Abdulaziz City for Science and Technology (KACST), Riyadh, Kingdom of Saudi Arabia, under grant number APR-34-13. The authors acknowledge, with thanks, KACST, Science and technology unit King Abdulaziz University for technical and financial support.

**Consent to publish**

Written informed consent for publication of their clinical details and/or clinical images was obtained from the patient/parent/guardian/relative of the patient. A copy of the consent form is available for review by the Editor of this journal.

## P78 Copy number variations in Saudi patients with intellectual disability and epilepsy

### Muhammad I. Naseer ^1^, Muhammad Faheem^2^, Adeel G. Chaudhary ^1^, Mahmood Rasool^1^, Gauthaman Kalamegam^1^, Fai Talal Ashgan^1^, Mourad Assidi^1^, Farid Ahmed^1^, Syed Kashif Zaidi^1^, Mohammed M. Jan^3^, Mohammad H. Al-Qahtani^1^

#### ^1^Center of Excellence in Genomic Medicine Research, King Abdulaziz University, Jeddah, Saudi Arabia; ^2^Department of Biochemistry, Faculty of Science, King Abdulaziz University, Jeddah, KSA; ^3^Department of Pediatrics, Faculty of Medicine, King Abdulaziz University, Jeddah, Kingdom of Saudi Arabia

##### **Correspondence:** Muhammad I. Naseer (mimrannaseer@kau.edu.sa) – Center of Excellence in Genomic Medicine Research, King Abdulaziz University, Jeddah Saudi Arabia

**Background**

Epilepsy is genetically complex disorder affecting 1 % population of people of the world irrespective of their age, groups and fluctuating in its type and severity. Now a day’s high throughput sequencing such as DNA array-based studies have reported relevant copy number variations (CNVs) in 5-30 % of patients with epilepsy. These CNVs in epileptic patients is always remaining a challenge to find the causative genes for epilepsy in the population.

**Results**

In this study to observe novel CNVs deletion and duplication in the patients with epilepsy we investigated 40 patients with numerous patterns of seizures with epilepsy, minor dysmorphism and intellectual disability (ID). We used the high density whole genome Agilent sure print G3 Hmn CGH 2x 400 K array-CGH chips to find these variations. Our outcomes showed many novel CNVs including the deletions and duplications along with deletion plus duplication in the patients on different chromosomal regions. Duplications were detected in the chromosomal regions 2q13, 5p14.3, 6q23.2, 7p15.2, 19p12.13 respectively and deletions were observed in the chromosomal regions 1q29, 5p14.3, 6p25.3, 7q32.3, 14q11.2 and 19p13.13 respectively. Furthermore, duplication plus deletions were observed in 3q12.3, 5p14.3, 19p13.13. We found several genes related to ion channel and genes involved in neuron differentiation, so that the frequently occurring seizures may be due to loss or haploinsufficiency of one or more of these genes. Moreover, the array CGH results were also validated by using primer design of deleted regions utilizing the flanked SNPs using simple PCR and also by using real time PCR.

**Conclusions**

In our study we found some of the novel deletions and duplication for the first time in Saudi population. A large proportion of our patients showed at least one rare copy number variant. Our results suggest unless there is a strong indication for a specific monogenic syndrome otherwise the array-CGH should be considered as a first line of genetic test for epilepsy. The use of advanced technologies to identify novel mechanisms for fundamental epileptic disorder may help to recover the clinical management of the patients in lowering the load of epilepsy in the Saudi population.

**Acknowledgements**

This project was funded by the National Plan for Science, Technology and Innovation (MAARIFAH) – King Abdulaziz City for Science and Technology - the Kingdom of Saudi Arabia – award number (12-BIO3059-03). The authors also, acknowledge with thanks Science and Technology Unit, King Abdulaziz University for technical support”.

## P79 Prognostic significance of CD44 expression profile in colorectal carcinoma

### Maryam Al-Zahrani^1^, Sahira Lary^1^, Sahar Hakamy^2^, Ashraf Dallol^2^, Mahmoud Al-Ahwal^3^, Jaudah Al-Maghrabi^4^, Emmanuel Dermitzakis^5^, Adel Abuzenadah^2^, Abdelbaset Buhmeida^2^, Mohammed Al-Qahtani^2^

#### ^1^Biochemistry Department, King Abdulaziz University, Jeddah, Saudi Arabia; ^2^Center of Excellence in Genomic Medicine Research, King Abdulaziz University, Jeddah, Saudi Arabia; ^3^Department of Medicine, Faculty of Medicine, King Abdulaziz University, Jeddah, Saudi Arabia; ^4^Department of Pathology, Faculty of Medicine, King Abdulaziz University, Jeddah, Saudi Arabia; ^5^Department of Genetic Medicine and Development, Medical School, University of Geneva, Geneva, Switzerland

##### **Correspondence:** Abdelbaset Buhmeida (abuhmeida@kau.edu.sa) – Center of Excellence in Genomic Medicine Research, King Abdulaziz University, Jeddah, Saudi Arabia

**Background**

Antibodies naturally exist as part of the specific immune system and their main function is to detect foreign substances and target them for elimination.CD44 (HCAM) (homing cell adhesion molecule) antibody is a multifunctional class I transmembrane glycoprotein (80 kDa) present on T lymphocytes, granulocytes, red blood cells, brain, and epithelial cells. CD44 is expressed on malignant cells, as well as on cancer stem cells. Therefore, CD44 plays an essential role in tumour progression by helping in cancer invasion and metastasis (Jaggupilli and Elkord, 2012).The aim of this study was to elucidate the prognostic impact of the cancer marker CD44 by immunohistochemistry (IHC) in colorectal cancer (CRC) and determine its value as potential biomarker of clinical outcome.

**Patients and methods**

The expression of CD44 was evaluated by automated immunohistochemistry in 149 Formalin-fixed and paraffin-embedded tissues of CRC. The expression profile of immunostaining was evaluated by objective method (index score) that considered both intensity and fraction/extension of expression pattern.

**Results**

**Fig. 26 (abstract P79):**
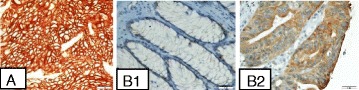
Immunohistochemical staining of CD44. **a** Normal tissue (no expression); **b** colorectal cancer tissue (B1-moderate and B2-strong expression)

The expression of CD44 was predominantly membranous and /or cytoplasmic (Fig. 26). Immunostaining results showed that there was no association between CD44 immunoexpression and age, gender and grade of the tumour, however it was found to have a significant association with tumour location (*p* = 0.039) and tumour stage (*p* = 0.007). In univariate analysis, there was no correlation between CD44 expression patterns and disease-free survival (DFS). However, there was a significant correlation, with disease-specific survival (DSS), in that patients with CD44 low expression patterns tumours have longer survival outcome (p <0.03).

**Conclusions**

Our study primarily showed that CD44 expression profile has a potential prognostic value in CRC; however, larger cohort with complete follow up data, in addition to deep functional molecular work is highly recommended in order to assess the correlation between CD44 and CRC carcinogenesis and progression.

**References**

1. Jaggupilli A and Elkord E: **Significance of CD44 and CD24 as cancer stem cell markers: an enduring ambiguity.***Clin Dev Immunol*. 2012, 708036.

**Table 15 (abstract 79):** Correlation of CD44 expression patterns with selected clinic-pathological features of CRC

Characteristic	No. of patients (%)	p value
Gander:		0.14
Male	126 (56.3%)	
Female	98 (43.7%)	
Age (yrs.)*:		0.71
≤60 years	112 (50%)	
>60 years	112 (50%)	
Pathological stage:		0.007
Stage I	3 (2.1%)	
Stage II	20 (14%)	
Stage III	106 (74.1%)	
Stage VI	14 (9.8%)	
Localization:		0.03
Rt colon	61(28%)	
Lt colon and rectum	157 (72%)	
Status of patient:		0.03
Alive	(106) 77.4%	
Dead	(31) 22.6%

## P80 Association of the endothelial nitric oxide synthase (eNOS) gene G894T polymorphism with hypertension risk and complications

### Abeer A Al-refai^1,4^, Mona Saleh^1^, Rehab I Yassien^2^, Mahmmoud Kamel^2^, Rabab M Habeb^3^

#### ^1^Medical Biochemistry Departments, Faculty of Medicine, Menoufia University, Shibin Al Kawm, Egypt; ^2^Departments of Cardiology, Faculty of Medicine, Menoufia University, Shibin Al Kawm, Egypt; ^3^Departments of Anesthesia, Faculty of Medicine, Menoufia University, Shibin Al Kawm, Egypt; ^4^Biochemistry Departments, Faculty of Medicine, Umm Al-Qura University, Makkha, KSA

##### **Correspondence:** Abeer A Al-refai (drabeer1975@hotmail.com) – Biochemistry Departments, Faculty of Medicine, Umm Al-Qura University, Makkha, KSA

**Background**

Hypertension is a complex, multifactorial disease, influenced by a large number of genetic and environmental factors and their interaction. Objective: Our study aims to assess the association of eNOS (G894T) single neuclotide gene polymorphism (SNP) hypertension risk and its relation with variable hypertension predisposing conventional risk factors. Methodology: eNOS (G894T) SNP by real-time PCR was performed in 70 hypertensive patients (25 have CAD proven by coronary angiography& 20 are diabetic) and 30 age and sex matched apparently healthy individuals. Lipid profile (TG, TC, LDL and HDL) glucose profile were assessed by colorimetry.

**Results**

Hypertensive patients had significantly increased systolic and diastolic blood pressure (P < 0.001), lipid profile (P < 0.001), fasting blood glucose, 2 h-PPG (P < 0.001) and smoking status (P = 0.01) as compared to control. No significant difference between the 2 participants groups regarding genotypic distribution of (G894T) SNP (P > 0.05). However, the combination of (GT + TT) eNOS genotype and T allele significantly increase the risk of hypertension (OR = 3.86& 4.33) respectively. Subgroup analysis based on associated complications showed significant association between CAD and eNOS (G894T) in mutant genotype (P = 0.002) and allele frequency (P < 0.001). Moreover, the combined mutant homozygous and heterozygous eNOS genotype are significantly associated with higher TC, LDLc, (P < 0.001) and TG (P = 0.001). Thus dyslipidemia (not shown), CAD (P = 0.002 & OR = 5.01) and hypercholesterolemia (P < 0.001(&12.48) increase the risk of hypertension among T carrier CI (1.68-14.98) & (3.679-42.33) respectively.

**Conclusions**

These results indicated that the T carriers which are weakly associated with hypertension, could increase the hypertension risk with hypercholestolemia (increased both TC and LDLc) and complications (CAD).

**References**

1. Bauer UE, Briss PA, Goodman RA, Bowman BA. **Prevention of chronic disease in the 21st century: elimination of the leading preventable causes of premature death and disability in the USA**. *Lancet* 2014; **384**:45-52.

2. Dodhia H, Phillips K, Zannou MI, Airoldi M, Bevan G. **Modelling the impact on avoidable cardiovascular disease burden and costs of interventions to lower SBP in the England population**. *Journal of Hypertens* 2012; **30**:217-26.

3. Montezano A, Harvey A, Dulak-Lis M, Briones A, Tsiropoulou S and Touyz R. **Oxidative Stress and Human Hypertension: Vascular Mechanisms, Biomarkers, and Novel Therapies**. *Canadian Journal of Cardiology* 2015; **31**: 631-641.

**Fig. 27 (abstract P80):**
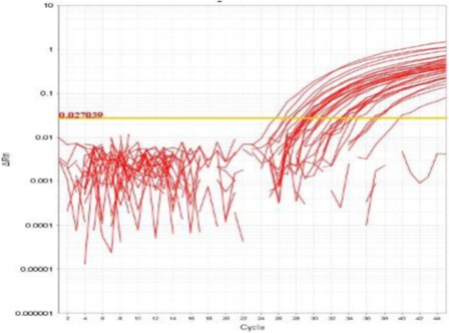
Amplification plot (Rn vs. cycle )- assay 1-Allele 2

**Table 16 (abstract P80):** eNOS gene polymorphism data of the studied patients and controls

	Groups				Test	P-Value	Odds Ratio
	Patients (N= 70)		Control (N= 30)				
	No	%	No	%			
Genotype					Z		
GG	49	70.0	27	90.0	1.89	0.058	-
GT	16	22.9	3	10.0	1.23	0.219	TT + GT
TT	5	7.1	0	0.0	1.0	0.319	3.86(1.05-14.12)
G	114	81.4	57	95.0		χ^2^	
T	26	18.6	3	5.0	6.24	0.012(S)	4.33(1.26-14.92)

## P81 SNPs array to screen genetic variation among diabetic patients

### Najlaa Filimban^1^, Ashraf Dallol^1,2^, Nadia Ghannam^3^, Mohammed Al-Qahtani^2^, Adel Mohammed Abuzenadah^1,2,4^

#### ^1^Center of Innovations in Personalized Medicine, King Abdulaziz University, Jeddah, Kingdom of Saudi Arabia; ^2^Center of Excellence in Genomic Medicine Research, King Abdulaziz University, Jeddah, Kingdom of Saudi Arabia; ^3^Diabetes & Endocrinology Center of Excellence, Clinical Nutrition Department, International Medical Center, Jeddah, Kingdom of Saudi Arabia; ^4^Faculty of Applied Medical Sciences, King Abdulaziz University, Jeddah, Kingdom of Saudi Arabia

##### **Correspondence:** Ashraf Dallol (adallol@kau.edu.sa) – Center of Excellence in Genomic Medicine Research, King Abdulaziz University, Jeddah, Kingdom of Saudi Arabia

**Background**

The accurate determination of single nucleotide polymorphisms (SNPs) has received immense attention, particularly in genome-wide association studies (GWAS). Although enormous SNPs were identified, only the ones that are known to cause diseases are considered to investigate the genetic predisposition to complex diseases such as diabetes. However, it is conceptually known that allele with low frequency might have genetic effects influencing diabetic phenotypic traits. Therefore, we address the importance of detecting the allele and genotype frequency and eventually examine the common genetic variants that are significantly associated with diabetic traits. The study aimed to screen a spectrum of SNPs in a one single run taking advantage of the large-scale genotyping technology.

**Materials and methods**

Selected genetic loci that are located on chromosome 16, and previously known to be associated with diabetes and obesity were screened utilizing the availability of Taqman Genotyping Open Array plate. The frequency was estimated for each individual allele and genotype in the total sample population.

**Results**

Data have shown the presence of uncommon and rare SNPs variants in VKORC1 gene.

**Conclusions**

Identifying SNPs- related diabetes is a very challenging approach creating a clinical debate whether these variants have a meaningful value in predicting diabetes risk. Further study has to be conducted to assess the implication extent of the genetic variations in the development of the disease.

## P82 Detection and genotyping of *Helicobacter pylori* among gastric cancer patients from Saudi Arabian population

### Fehmida Bibi^1^, Sana Akhtar^1^, Esam I. Azhar^1,2^, Muhammad Yasir^1^, Muhammad I. Nasser^3^, Asif A. Jiman-Fatani^4^, Ali Sawan^5^

#### ^1^Special Infectious Agents Unit, King Fahd Medical Research Center, King Abdulaziz University, Jeddah, 21589, Kingdom of Saudi Arabia; ^2^Department of Medical Laboratory Technology, Faculty of Applied Medical Sciences, King Abdulaziz University, Jeddah, Saudi Arabia; ^3^Center of Excellence in Genomic Medicine Research (CEGMR), King Abdulaziz University, Jeddah, 21589, Saudi Arabia; ^4^Department of Medical Microbiology and Parasitology, Faculty of Medicine, King Abdulaziz University, Jeddah, Saudi Arabia; ^5^Department of Anatomical pathology, Faculty of Medicine, King Abdulaziz University, Jeddah, Saudi Arabia

##### **Correspondence:** Fehmida Bibi (fnaseer@kau.edu.sa) – Special Infectious Agents Unit, King Fahd Medical Research Center, King Abdulaziz University, Jeddah, 21589, Kingdom of Saudi Arabia

**Background**

Gastric cancer (GC) is frequent and second cause of cancer related deaths. The pathogenesis of gastric cancer includes a sequence of events that begins with *Helicobacter pylori* (*H. pylori*) induced chronic superficial gastritis, progressing towards atrophic gastritis, intestinal metaplasia, dysplasia and eventually GC. In this study we aim to determine the presence and identification of *H. pylori* from different gastric biopsies. Further *H. pylori* virulence factors cagA and vacA genotypes will be determined by PCR.

**Materials and methods**

This study includes 30 paraffin embedded gastric specimens from normal and gastric cancer patients, pathologically diagnosed for gastric cancer from King Abdulaziz University Jeddah Saudi Arabia. Detection of *H. pylori* strain was performed by using specific primers targeting 16S rRNA and *ureA* genes. The *cagA, vacA, GlmM, IceA1, IceA2* and *HPU1* presence was determined from *H. pylori* positive samples by PCR using their respective primers.

**Results**

Molecular identification of *H. pylori* using specific genes (ureA and 16S rRNA) revealed that (26) 86 % of samples were *H. pylori* positive. We found that prevalence of *cagA, vacA* and *GlmM* were more as compared to other genotypes such as *IceA1, IceA2* and *HPU1* in gastric cancer patients. All samples negative for 16S rRNA were also negative for *cagA, vacA* and *GlmM.*

**Conclusions**

Our results show high prevalence of *cagA and vacA* in gastric cancer patients. This study might be of clinical significance in precise and early diagnoses of gastric cancer and treat gastric patients by understanding the trend of *H. pylori* infection in Saudi Population.

**Acknowledgements**

This project was supported by the NSTIP strategic technologies program in the Kingdom of Saudi Arabia-Project No. (12-BIO2725-03). The authors also acknowledge with thanks Science and Technology Unit, King Abdulaziz University for technical support.

## P83 Antimicrobial drug resistance and molecular detection of susceptibility to Fluoroquinolones among clinical isolates of Salmonella species from Jeddah-Saudi Arabia

### Ruaa A Lahzah, Asho Ali

#### Department of Microbiology, King Abdulaziz University, Jeddah, Saudi Arabia

##### **Correspondence:** Ruaa A Lahzah (Ruaa.lahzah@gmail.com) – Department of Microbiology, King Abdulaziz University, Jeddah, Saudi Arabia

**Background**

Non-Typhoid Salmonellosis (NTS) is one of the leading zoonotic food-borne illnesses. Infections caused by NTS ranges from 250 to 3200 per 100,000 population across the globe. High number of cases (ranges between 44-132) have been reported from Makkah, Saudi Arabia (KSA) during Hajj season. Increased antimicrobial resistance in NTS from across the world has further compounded the problem. Fluoroquinolones (FQs) are the drugs of choice for the treatment of drug-resistant NTS infections. However, over use of FQs in human and misuse in animal feeds has led to increase in FQ resistance as well throughout the world. No data is available about NTS infections and FQ resistance in NTS from Jeddah, KSA, therefore this study primarily explored the phenotypic FQs susceptibility among NTS isolates from Jeddah. Secondly, phenotypic FQ resistance was also correlated with mutations in FQ resistance detection gyrase (*gyrA*) and topoisomerase (*parC*) genes.

**Materials and methods**

A total of 48 NTS isolates were collected during 2014 in one of the public sector hospital from patients in Jeddah, KSA. Antimicrobial susceptibility was determined using Clinical and Laboratory Standards Institute methodology. The presence of mutations for FQs resistance was detected in *gyrA* and *parC* genes by PCR- based gene-sequencing method.

**Results**

Thirty-eight percent of (18/48) patients’ NTS isolates were resistant to ciprofloxacin phenotypically. Gene sequencing revealed mutations in two codons of *gyrA* and *parC* genes each among 13 out of 18 FQ resistant isolates. Whereas one FQ resistant isolate showed mutation only in *parC* gene. Mutations were observed at codons 83 and 87 (S83F, S83Y, D87G, D87Y,D87W and D87N) in gyrA and on codons 57 and 80 (*S57T, S80I and S80W)* in *pyrC* gene. None of the FQ susceptible isolates showed mutations in *gyrA* and *parC*.

**Conclusions**

This study exhibits prevalence of FQ resistant NTS infections in Jeddah. Prevalent mutations in *gyrA* and *parC* genes among FQ resistant isolates may assist in development of rapid FQ resistance detection method. However, wild type *gyrA* and *parC* genes among 4/18 phenotypic FQ resistant NTS isolates also indicates presence of an alternate mechanism, such as drug resistance pump, for FQ resistance which needs further investigation.

## P84 Identification of the toxic and virulence nature of MAP1138c protein of *Mycobacterium avium* subsp. *paratuberculosis*

### Syed A Hassan^1^, Seyed E Hasnain^2^, Iftikhar A Tayubi^1^, Hamza A Abujabal^3^, Alaa O Magrabi^4^

#### ^1^Department of Computer Science, Faculty of computing and Information Technology Rabigh, King Abdulaziz University, Rabigh, 21911, Saudi Arabia; ^2^Kusuma School of biological Sciences, Indian Institute of Technology, Delhi (IITD), Hauz Khas, New Delhi 110016, India; ^3^Mathematics Department, Faculty of Sciences King Abdul Aziz University P. O. Box 80003 Jeddah 21589 Saudi Arabia; ^4^Department of Information Technology, Faculty of computing and Information Technology Rabigh, King Abdulaziz University, Rabigh, 21911, Saudi Arabia

##### **Correspondence:** Syed A Hassan (asif_srmcbt@yahoo.com) – Department of Computer Science, Faculty of computing and Information Technology Rabigh, King Abdulaziz University, Rabigh, 21911, Saudi Arabia

**Background**

The complete genome of the reference *Mycobacterium avium* subspecies *paratuberculosis* (MAP) strain K-10 has been sequenced [1]. This has provided us with many opportunities to study the interaction of many putative virulence genes with its hosts and the environment. Recently, through an *in silico* analysis of a putative virulence factor (MAP1138c), it was postulated that since MAP1138c share a sequential, structural and functional homology with Rv1141c (LprG) of *Mycobacterium tuberculosis* it can possibly play a role in the escaping the cell-mediated immune response within host macrophages in paratuberculosis infection [2]. Therefore, considering this fact the *in vitro* characterization of the putative ORF MAP1138c is useful in understanding nature of the protein and its role in the virulence of MAP in ruminants.

**Materials and methods**

In this study, we have cloned, purified MAP1138c and have performed protein toxicity assays and ELISA to detect the presence of MAP1138c in MAP infected sheep serum. Data were analyzed using *GraphPad Prism 5* software. ELISA data were compared using a nonparametric Mann–Whitney U test on O.D values at 490 nm. The level of significance was set at p < 0.05.

**Results**

**Fig. 28 (abstract P84):**
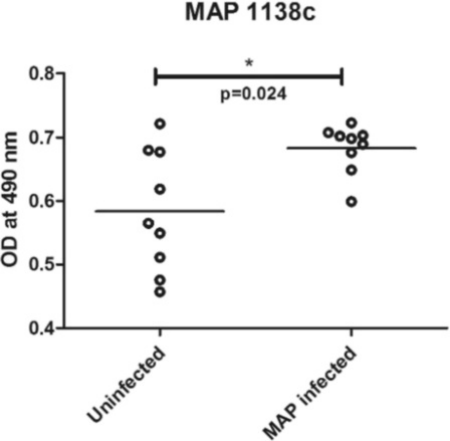
Serum antibody response for a MAP1138c protein expressed as optical density for infected animals (N = 20) and control animals (Neg) (N = 10). The horizontal line marks the group average value

MAP1138c was cloned, purified and confirmed using western blot. The purified MAP1138c protein was used to perform immunoassay and toxicity assay to work out a possible functional role for the putative MAP1138c protein. From our analyses, it appears that MAP1138c plays a role in infection of MAP in sheep. Despite the conserved nature of this protein, a significant difference (p < 0.05) was observed in serum antibody levels between control animals (N = 10) and infected sheep’s (N = 20) serum for MAP1138c (Fig. 28). The toxic nature of MAP1138c was also established as the presence and expression of protein were negligible in pRSETA-MAP1138c expression vector while contrary results were obtained using pET28a-MAP1138c expression vector system (Table 17).

**Conclusions**

These studies reveal the possible role of MAP1138c protein in virulence and pathogenesis of MAP. Further, biochemical and functional studies of MAP1138c protein will surely enhance our understanding of the role of MAP1138c protein in the pathogenesis of MAP in ruminants and humans.

**References**

1. Li L, Bannantine JP, Zhang Q, Amonsin A, May BJ, Alt D, Banerji N, Kanjilal S, Kapur V: **The complete genome sequence of Mycobacterium avium subspecies paratuberculosis.***Proc Natl Acad Sci* 2005, **102**:12344-12349.

2. Hassan SA: **In silico approach to identify the role of a putative protein MAP1138c in the virulence of Johne’s disease.***Genes Genom* 2015, **37**:327–338.

**Table 17 (abstract P84):** The table depicts the results for the transformation efficiency, expression and toxicity studies of pRESTA-MAP1138c and pET28a-MAP1138c expression vector constructs in BL21DE3, pLysS and pLysE host cells

Expression vectors	Transformation efficiency (transformants/μg DNA) of strains			Toxicity Antibiotics + 1mM IPTG over night			Expression in liquid media (Antibiotics + 1mM IPTG for 3 h)		
	BL21DE3	BL21(DE3)-pLysS	BL21(DE3)-pLysE	BL21DE3	BL21(DE3)-pLysS	BL21(DE3)-pLysE	BL21DE3	BL21(DE3)-pLysS	BL21(DE3)-pLysE
pRSETA-MAP1138c	1400 ± 100	14434 ± 208	16646 ± 150	Yes	Yes	Yes	No	No	Yes
									Very Low
pET28a-MAP1138c	3050 ± 50	24533 ± 416	2500 ± 208	Yes	No	No	Yes	Yes	Yes
							Low	High	Low

## P85 *In vitro* and *in silico* evaluation of miR137 in human breast cancer

### Fazal Khan^1,2,3^, Gauthaman Kalamegam^1^, Peter Natesan Pushparaj^1^, Adel Abuzenada^1,2^, Taha Abduallah Kumosani^3^, Elie Barbour^4^, Mohammed Al-Qahtani^1^

#### ^1^Center of Excellence in Genomic Medicine Research, King Abdulaziz University, Jeddah, Saudi Arabia; ^2^Center of Innovation in Personalized Medicine, King Fahd Medical Research Center, King Abdulaziz University, Jeddah, Saudi Arabia; ^3^Biochemistry Department, Faculty of Science; Production of Bioproducts for Industrial Applications Research Group; and Experimental Biochemistry Unit, King Fahd Medical Research Center King, Abdulaziz University, Jeddah, Saudi Arabia; ^4^Department of Agriculture, Faculty of Agricultural and Food Sciences, American University of Beirut (AUB), Beirut, Lebanon; adjuncted to Biochemistry Department, King Abdulaziz University, Jeddah, Saudi Arabia

##### **Correspondence:** Gauthaman Kalamegam (kgauthaman@kau.edu.sa) – Center of Excellence in Genomic Medicine Research, King Abdulaziz University, Jeddah, Saudi Arabia

**Background**

Cancer is one of the leading cause of deaths worldwide, and there has been an increase in the development of multidrug resistance to cancer [1]. Micro RNAs (miRs) are small 21-25 nucleotides long single stranded molecules that interact with their complementary sequences and regulate the different genes [2]. MiR-137 is overexpressed in mammogenesis during embryonic development and significant overexpression of miR137 in MDA-MB231 breast cancer cell line inhibited breast cancer formation in nude mice [3]. We aim to identify the pathways and mechanisms influenced by miR137 in breast cancer using *in vitro* and *in silico* studies.

**Materials and methods**

Human breast adenocarcinoma cells (MCF-7) were seeded in a 24 well plate (2 × 10^4^/well) and allowed to attach overnight. The cells were transfected with miR137 at 30nM and 100nM concentrations using lipofectamine 2000^©^ according to the manufacturer’s protocol. Following culture of transfected MCF-7 cells for 48 h, any changes in their morphology (Phase microscopy), cell proliferation (MTT assay), cell cycle and apoptosis (AnnexinV-FITC and PI staining using FACS) were studied. Ingenuity Pathway Analysis (IPA, Ingenuity Systems, Qiagen, USA) was utilized to determine miR137 targets and pathways in human breast cancer.

**Results**

**Fig. 29 (abstract P85):**
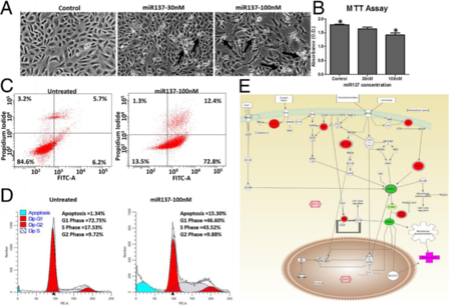
**a** Morphological changes in miR137 treated MCF-7 cells (arrows indicate cell death); **b** MTT assay showing decrease in cell proliferation; **c** AnnexinV-FITC and PI staining showing apoptotic cells; **d** Cell cycle and **e** IPA analysis of miR137 targets

MCF-7 cells treated with 100nM miR137 revealed cell shrinkage and cell death (Fig. 29a). MTT results showed inhibition of cell proliferation by 20.70 % compared to control and this decrease was statistically significant (Fig. 29b). AnnexinV-FITC and PI staining showed high percentage (72.8 %) of cells in the pre-apoptotic phase and low percentage (12.4 %) in late-apoptotic stage (Fig. 29c). Cell cycle analysis also showed ‘S’ phase arrest and increase in sub G1-phase (15.20 %) indicative of apoptosis compared to control (Fig. 29d). The IPA analysis for miR137 in breast cancer revealed several target proteins and pathways that are implicated in breast cancer pathogenesis (Fig. 29e).

**Conclusions**

miR137 demonstrated inhibition of MCF-7 cells *in vitro* and induced mophological changes leading to cell death via an apoptotic mechanism. *In silico* analysis identified several key proteins which are involved in breast cancer pathogenesis in addition to the most commonly involved HER-2 signaling and stathmin regulated pathways. IPA analysis thus provides additional insights to explore novel therapeutics.

**Acknowledgements**

This financial support by King Abdulaziz City for Science and Technology (KACST) through postgraduate grant funding (AT-34-237) is greatly acknowledged.

**References**

1. Zhu, X., et al., **miR-137 restoration sensitizes multidrug-resistant MCF-7/ADM cells to anticancer agents by targeting YB-1***. Acta biochimica et biophysica Sinica*, 2012: p. gms099.

2. Pushparaj, P.N., et al., **RNAi and RNAa-the yin and yang of RNAome***. Bioinformation,* 2008. **2**(6): p. 235.

3. Lee, J.-M., et al., **A contrasting function for miR-137 in embryonic mammogenesis and adult breast carcinogenesis***. Oncotarget*, 2015. **6**(26): p. 22048-22059.

## P86 Auruka gene is over-expressed in Saudi breast cancer

### Manal Shabaad^1^, Shilu Mathew^1^, Ashraf Dallol^1^, Adnan Merdad^2^, Abdelbaset Buhmeida^1^, Mohammed Al-Qahtani^1^

#### ^1^Center of Excellence in Genomic Medicine Research, King Abdulaziz University, Jeddah, Saudi Arabia; ^2^Department of Surgery, King Abdulaziz University, Jeddah, Saudi Arabia

##### **Correspondence:** Abdelbaset Buhmeida (abuhme@utu.fi) – Center of Excellence in Genomic Medicine Research, King Abdulaziz University, Jeddah, Saudi Arabia

**Background**

Prognosis of breast cancer (BC) is mainly based on the tumor staging system and other traditional/conventional clinico-pathological features; however, staging system alone is in adequate in predicting the outcome of patients within same stage. Moreover, early stage BC patients, who subjected to surgery, have a tendency of recurrence within upcoming 10 years of follow up time. Therefore, searching for molecular markers that could help in prognosticating patients within same stage is of high priority in BC research. In this study, we evaluated the expression patterns of *AURKA* gene, a cell cycle regulator, which has tumorigenic activity, and verify its prognostic value. ,

**Patients and methods**

The retrospective study cohort consists of 137 female breast primary invasive ductal carcinoma samples and 2 non-cancerous tissues representing normal. The patients were diagnosed at the Department of Pathology, King Abdulaziz University and Bakhash Hospital, Jeddah, Saudi Arabia during years from 2000 to 2008. RNA extraction was carried out using an RNeasy FFPE Kit and was transcripted by using Sensiscript reverse transcription kit from Qiagen.

**Results**

In our study, 70 % of the breast cancer patients had shown over expression in *AURKA* genes for tumor samples versus benign samples. We found also the significant correlation between *AURKA* and recurrence rate (*p* < 0.009). *AURKA* gene is also over expressed in patients with high reoccurrence score (RS).

**Conclusions**

Our study showed that *AURKA* gene is highly expressed in tumors vs. benign cases and significantly associated with disease recurrence. These preliminary data confirmed that high *AURKA* gene expression patterns might be helpful in selecting a group of patients of early stage BC who have high recurrence rate in order to be subjected to adjuvant therapy.

## P87 The potential of immunogenomics in personalized healthcare

### Mourad Assidi^1,2^, Muhammad Abu-Elmagd^1,2^, Kalamegam Gauthaman^1^, Mamdooh Gari^1^, Adeel Chaudhary^1^, Adel Abuzenadah^1^, Peter Natesan Pushparaj^1^, Mohammed Al-Qahtani^1^

#### ^1^Center of Excellence in Genomic Medicine Research, King Abdulaziz University, Jeddah, Saudi Arabia; ^2^Center of Innovation in Personalized Medicine, King Abdulaziz University, Jeddah, Saudi Arabia

##### **Correspondence:** Peter Natesan Pushparaj (peter.n.pushparaj@gmail.com) – Center of Excellence in Genomic Medicine Research, King Abdulaziz University, Jeddah, Saudi Arabia

**Background**

Immunogenomics is an expanding field which will allow expanding basic knowledge about the contribution of the genomic immunology and its interaction with the environment including the microbiome of both human health and disease [1]. It helps us to understand the immune mechanisms in both health and disease and provides critical clues to foster the medical transition towards precision medicine through the provision of individualized diagnostics and therapeutics [2, 3]. In this study, we provide an overview of the recent developments in Immunogenomics and their potent role to develop novel preventive and therapeutic strategies for the wellbeing of humans.

**Materials and methods**

High throughput data, obtained from individual patients, using advanced technological platforms such as microarrays, next generation sequencing (NGS) methodologies were examined using freewares like R and commercial platforms like Genespring GX13.1 (Agilent, USA), Partek Genomics Suite (Partek Inc., USA), JMP Genomics Software (SAS, USA) etc., The differential expression patterns of immune genes has been analysed using the Database for Annotation and Visualization and Integrated Discovery (DAVID), and commercial knowledge bases like Pathway Analysis (IPA) (Ingenuity Systems, Qiagen USA), Pathway Studio (Elsevier, Netherlands) etc., to decipher biomarkers and novel immune-regulatory pathways.

**Results**

The screening of available high throughput data and further analysis of molecular using computational and comparative genomics tools provide us key biomarkers and/or pathways driving human immunogenomic behaviour at both physiological and pathological contexts.

**Conclusions**

Immunogenomics is, thus, an expanding field that offers unprecedented opportunities for better understanding of the intricate mechanisms of disease regulation and progression using genomics of the immune system. Therefore, precise prediction of susceptibility, early and accurate diagnosis using immunogenomic methodologies can be done frequently to improve personalized healthcare in the near future.

**References**

1. Mavrommatis B, Young GR, Kassiotis G: **Counterpoise between the microbiome, host immune activation and pathology**. *Curr Opin Immunol* 2013, **25**(4):456-462.

2. Green ED, Guyer MS: **Charting a course for genomic medicine from base pairs to bedside**. *Nature* 2011, **470**(7333):204-213.

3. Buonaguro L, Pulendran B: **Immunogenomics and systems biology of vaccines**. *Immunological reviews* 2011, **239**(1):197-208.

## P88 *In Silico* physiochemical and structural characterization of a putative ORF MAP0591 and its implication in the pathogenesis of *Mycobacterium paratuberculosis* in ruminants and humans

### Syed A Hassan^1^, Iftikhar A Tayubi^1^, Hani MA Aljahdali^2^

#### ^1^Deaprtment of Computer Science, Faculty of computing and Information Technology Rabigh, King Abdulaziz University, Rabigh, 21911, Saudi Arabia; ^2^Department of Information Systems**,** Faculty of computing and Information Technology Rabigh, King Abdulaziz University, Rabigh, 21911, Saudi Arabia

##### **Correspondence:** Syed A Hassan (asif_srmcbt@yahoo.com) – Department of Computer Science, Faculty of computing and Information Technology Rabigh, King Abdulaziz University, Rabigh, 21911, Saudi Arabia

**Background**

*Mycobacterium avium* subsp. *paratuberculosis* (MAP) is an intracellular pathogen of ruminants and humans [1-2]. However, the identification of virulence factors of these diseases and their mode of action in causing pathogenesis in human and ruminant are still partly understood. In this regard, identifying the role of the “two-component systems” in the pathogenesis and survival of MAP within host cells; considering the fact that it plays a role in the establishment and propagation of tuberculosis infection in humans [3].

**Materials and methods**

Therefore, a comprehensive sequential, structural and functional comparative study between phoP a response regulatory component of the “two-component regulatory system” of Mtb and its ortholog in MAP genome was conducted using computational tools namely EggNOG, ProtScan, ProtPram, Hydropathy plot, and PSIPRED. Swiss-model server was used to build the three-dimensional structure of an MAP0591 protein using the crystal structure of the Rv0757 protein as a template. The quality and reliability of generated model were evaluated using QMEAN and GMQE structure assessment tools of SWISS-MODEL server.

**Results**

**Fig. 30 (abstract P88):**
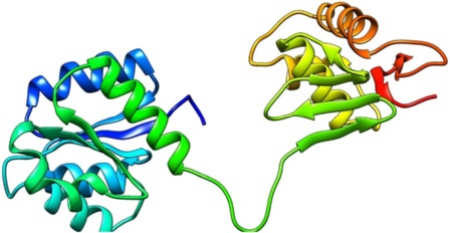
3D model of MAP0591 protein generated by SWISSMODEL server

EggNOG analysis showed that the MAP0591 protein of MAP is an ortholog of PhoP (Rv0757) protein of *Mtb* and shares sequential homology of 97.12 %. Further sequential analysis displayed that MAP0591 protein has a signal receiver domain and winged helix-turn-helix DNA binding domain suggesting its role in signal transduction and gene regulation. The ProtPram analysis shows similarity in various physical and chemical parameters between MAP0591 and Rv0757 proteins (Table 18). The hydropathy plot of MAP0591 and Rv0757 proteins showed strong negative peaks indicating the presence of high antigenic region along the protein sequence. Comparative PSIPRED analysis of MAP0591 with Rv0757 protein reveals a similarity of secondary structure between the two proteins. The predicted three-dimensional model of MAP0591 protein (Fig. 30) was found to be reliable using QMEAN and GMQE global structural evaluation tools of Swiss-model server.

**Conclusions**

This study highlights the physiochemical, sequential and structural similarity between the MAP0591 and Rv0757 proteins and opens the prospects that MAP0591 protein might play a role in the survival of MAP in host macrophages leading to latency and subsequent infection in human and ruminants.

**References**

1. Nielsen SS, Toft N: **A review of prevalences of paratuberculosis in farmed animals in Europe.***Prev Vet Med* 2009, **88**:1-14.

2. Naser SA, Sagramsingh SR, Naser AS, Thanigachalam S: ***Mycobacterium avium*subspecies*paratuberculosis*causes Crohn’s disease in some inflammatory bowel disease patients.***World J Gastroenterol* 2014, **20(23)**:7403–7415.

3. *Smith I:****Mycobacterium tuberculosis*Pathogenesis and Molecular Determinants of Virulence.***Clin Microbiol Rev* 2003, **16**:463-496.

**Table 18 (abstract P88):** A comparative study of the physiochemical parameters of MAP0591 and Rv0757 (PhoP) proteins

Protein	Protein Molecular Weight	Amino Acid Composition	Instability Index ( >40 = unstable)	Aliphatic Index	Grand Average of Hydropathicity (GRAVY)*
MAP0591	26476.2	239	39.70	98.70	-0.159
Rv0757 (PhoP)	27513.5	247	36.18	98.99	-0.191

*GRAVY (-ve) = hydrophilic nature and (+ve) = hydrophobic nature

## P89 Effects of heat shock on human bone marrow mesenchymal stem cells (BM-MSCs): Implications in regenerative medicine

### Reham Al Nono^1^, Mamdooh Gari^1,2,3^, Haneen Alsehli^4^, Farid Ahmed^2^, Mohammed Abbas^5^, Gauthaman Kalamegam^2^, Mohammed Al-Qahtani^2^

#### ^1^Department of Medical Laboratory Technology and Haematology, Faculty of Applied Medical Sciences, King Abdulaziz University, Jeddah, Saudi Arabia; ^2^Centre of Excellence in Genomic Medicine Research, King Abdulaziz University, Jeddah, Saudi Arabia; ^3^Sheikh Salem Bin Mahfouz Scientific Chair for Treatment of Osteoarthritis by Stem Cells, King Abdulaziz University, Jeddah, Saudi Arabia; ^4^Center of Innovation in Personalized Medicine, King Abdulaziz University, Saudi Arabia; ^5^Department of Orthopaedic Surgery, Faculty of Medicine, King Abdulaziz University Hospital, Jeddah, Saudi Arabia

##### **Correspondence:** Gauthaman Kalamegam (kgauthaman@kau.edu.sa) – Centre of Excellence in Genomic Medicine Research, King Abdulaziz University, Jeddah, Saudi Arabia

**Background**

Autologus stem cell transplantation for articular cartilage repair appears promising. However, increase or decrease in temperatures as associated with the use of arthroscope or laser drilling during stem cell based cartilage repair procedures may have detrimental effects on the transplanted stem cells [1]. In the present study, we attempt to evaluate the effect of heat shock on human bone marrow mesenchymal stem cells (hBM-MSCs) in relation to its proliferation and survival.

**Materials and methods**

Primary cultures of hBM-MSCs were established and characterized. Early passages of hBM-MSCs (1x10^5^cells) were exposed to different tempaeratures (37°C, 40°C, 50°C, 55°C) and duration (30s, 60s, 90s, 120 s) either as cell pellet or as cell suspensions. Following heat shock the hBM-MSCs were cultured under standard culture conditions for 24 hrs and changes in morphology (Phase-contrast imaging), cell proliferation (MTT assay), cell cycle (PI staining) and appoptosis (AnnexinV-FITC and PI) were studied.

**Results**

**Fig. 31 (abstract P89):**
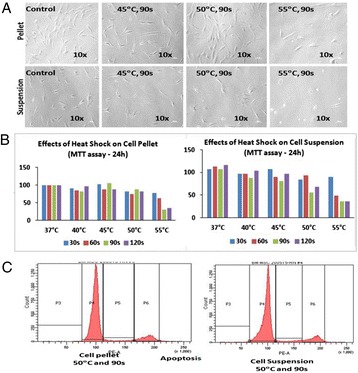
Effect of heat shock on hBM-MSCs. **a** - Cell morphology; **b** - Cell proliferation (MTT assay); **c** - Apoptosis (AnnexinV-FITC&PI) assay

Exposure to heat shock affected the cellular functions which was more pronounced in the suspension cells than pelleted cells. Cell death were observed in both groups at eleveated temperatures (50°C, 55°C) and longer durations (90s, 120 s) (Fig. 31a). There was an overall inhibition of cell proliferation in both cell suspension and cell pellet groups at 24 h (Fig. 31b). However, there was nearly 50 % decrease in inhbition in the cell suspension group compared to the cell pellet group at higher temperatures and duration studied (Fig. 31b). There were no changes in either the cell cycle or apoptosis (Fig. 31c) between the two groups.

**Conclusions**

Cell pellet have better proliferation and survival compared to cell suspension in response to heat shock. The observed cell death may be due to predominantly necrosis although current results wre negative for apoptois this cannot be ruled out. Further studies on differential expression of genes related to cell death, heat shock proteins are currently being pursued to ascertain the underlying mechanisms.

**Acknowledgements**

The Department of Orthopaedics at the King Abdulaziz University Hospital, King Abdulaziz University and the “Sheikh Salem Bin Mahfouz Scientific Chair for Treatment of Osteoarthritis by Stem Cells” which provided the clinical material are greatly acknowledged.

**References**

1. *Steadman JR, Ramappa AJ, Maxwell RB, Briggs KK.****An arthroscopic treatment regimen for osteoarthritis of the knee.****Arthroscopy 2007,****23****(9): 948–55.*

2. Muhamed M. H. Farhan-Alanie. Andrew C Hall: **Temperature changes and chondrocyte death during drilling in a bovine cartilage model and chondroprotection by modified irrigation solutions.***International Orthopedics (SICOT)* 2014, **38**: 2407-2412.

## P90 *In Silico* analyses of the molecular targets of Resveratrol unravels its importance in mast cell mediated allergic responses

### Shilu Mathew^1^, Fazal Khan^1,2,3^, Mahmood Rasool^1^, Mohammed Sarwar Jamal^4^, Muhammad Imran Naseer^1^, Zeenat Mirza^4^, Sajjad Karim^1^, Shakeel Ansari^1^, Mourad Assidi^1^, Gauthaman Kalamegam^1^, Mamdooh Gari^1^, Adeel Chaudhary^1^, Adel Abuzenadah^1^, Peter Natesan Pushparaj^1^, Mohammed Al-Qahtani^1^

#### ^1^Center of Excellence in Genomic Medicine Research, Faculty of Applied Medical Sciences, King Abdulaziz University, Jeddah, Saudi Arabia; ^2^Center of Innovation in Personalized Medicine, King Abdulaziz University, Jeddah, Saudi Arabia; ^3^Biochemistry Department, Faculty of Science; Production of Bioproducts for Industrial Applications Research Group; and Experimental Biochemistry Unit, King Fahd Medical Research Center King, Abdulaziz University, Jeddah, Saudi Arabia; ^4^King Fahd Medical Research Center, Faculty of Applied Medical Sciences, King Abdulaziz University, Jeddah, Saudi Arabia

##### **Correspondence:** Peter Natesan Pushparaj (peter.n.pushparaj@gmail.com) – Center of Excellence in Genomic Medicine Research, Faculty of Applied Medical Sciences, King Abdulaziz University, Jeddah, Saudi Arabia

**Background**

Resveratrol (RSV) is a phytoalexin produced by plants in environmental stress or pathogenic bout [1]. Phytoalexins offer resistance against an array of infectious agents in plants [1, 2]. RSV has earlier been shown to possess anti-inflammatory effects using *in vitro* and *in vivo* model systems [3, 4]. Mast cells are innate immune cells that play a pivotal role in the regulation of allergy, allergic rhinitis, anaphylaxis, atopic dermatitis, asthma and other related disorders [5]. In our present study, we specifically dissect the effect of RSV in mast cell mediated signaling process using *in silico* approaches.

**Materials and methods**

The list of target molecules for RSV was obtained using the Ingenuity Pathway Analysis (IPA) software (Ingenuity Systems, Qiagen, USA). The RSV target genes were further clarified using Fisher’s Exact Test and Benjamini Hochberg Multiple Testing Correction (P < 0.05) and subjected to core analysis using IPA to decipher proteins implicated in mast cell signaling. In order to understand the binding efficiencies of RSV with proteins implicated in mast cell signaling, computational docking software named CLC Drug Discovery Workbench version 2.5 (CLC Bio, Qiagen, USA) was used.

**Results**

**Fig. 32 (abstract P90):**
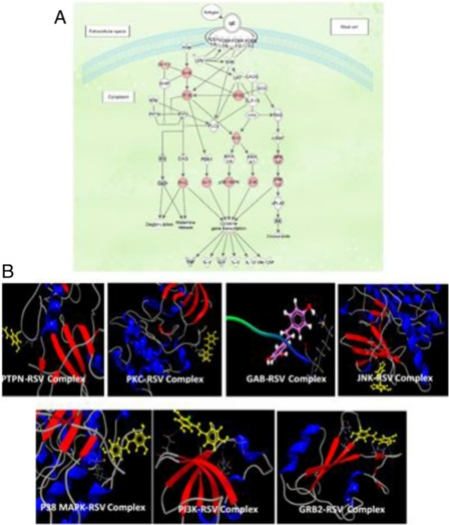
**a**. IPA core analysis of the molecules regulated by RSV showed that the Fc epsilon RI mediated signaling molecules in mast cells were significantly inhibited (*Red*). **b**. Molecular docking studies of PTPN, PKC, GAB and JNK (*top panel*), and p38 MAPK, PI3K and GRB2 (*bottom panel*) showing significant interactions with RSV

Ingenuity knowledge base showed that RSV potently regulates proteins involved in the mast cell signaling pathway such as GRB2, GAB, PI3K, PTPN 11, PKC, JNK, and p38 MAPK (Fig. 32a). These intracellular proteins specifically control the Fc epsilon mediated mast cell signaling in allergic responses. Furthermore, the *in silico* docking approach (Fig. 32b) showed that RSV significantly interacts and binds strongly (Docking Score Range: - 4 to -16 Kcal/mol) with GRB2, GAB, PTPN 11, PKC, PI3K, JNK and p38 MAPK (Table 19).

**Conclusions**

Our *in silico* study reiterates the importance of mast cell mediated signaling in innate immune responses. The inhibition of proteins involved in Fc epsilon signalling by RSV is essential for the attenuation of transcription of proinflammatory cytokines and chemokines in mast cells and could lead to the reduction of allergic responses. However, further *in vitro* studies in human cord blood derived mast cells (hCBMCs) are required to precisely translate the anti-inflammatory mechanisms of RSV.

**Acknowledgements**

This work was funded by the National Plan for Science, Technology and Innovation (MAARIFAH) – King Abdulaziz City for Science and Technology - the Kingdom of Saudi Arabia – award numbers (12-BIO2719-03) and (12-BIO2267-03). The authors also, acknowledge with thanks Science and Technology Unit, King Abdulaziz University for their excellent technical support.

**References**

1. Hain R, Reif HJ, Krause E, Langebartels R, Kindl H, Vornam B, Wiese W, Schmelzer E, Schreier PH, Stöcker RH: **Disease resistance results from foreign phytoalexin expression in a novel plant.***Nature* 1993, **361:** 153-56.

2. Sajish M, Schimmel P: **A human tRNA synthetase is a potent PARP1-activating effector target for resveratrol.***Nature* 2015 519:370-73.

3. Han SY, Bae JY, Park SH, Kim YH, Park JH, Kang YH: **Resveratrol inhibits IgE-mediated basophilic mast cell degranulation and passive cutaneous anaphylaxis in mice.***J Nutr* 2013, **143:**632-39.

4. Kang OH, Jang HJ, Chae HS, Oh YC, Choi JG, Lee YS, Kim JH, Kim YC, Sohn DH, Park H, Kwon DY: **Anti-inflammatory mechanisms of resveratrol in activated HMC-1 cells: pivotal roles of NF-kappaB and MAPK.***Pharmacol Res* 2009, **59:**330-37.

5. Manikandan J, Kothandaraman N, Hande MP, Pushparaj PN: **Deciphering the structure and function of FcεRI/mast cell axis in the regulation of allergy and anaphylaxis: a functional genomics paradigm*.****Cell Mol Life Sci* 2012, **69:** 1917-29.

**Table 19 (abstract P90):** RSV docking results obtained with proteins implicated in Fc epsilon mediated signaling in mast cells

Ligands	Docking score ( Kcal/mol)	H-Bonds ( Kcal/mol)	Interactions	Binding Residues
GRB2	-4.236	-8.365	4	O-SER96(A)(2)
				O-ARG112(A)(2)
GAB	-10.313	-4.236	1	N-LYS1(B)
PI3K	-5.462	-6.616	3	O-ASN57(A)(2)
				O-GLU61(A)
PTPN 11	-16.357	-9.362	1	O-GLY182(A)
PKC	-10.369	-7.161	1	O-LYS654(A)
JNK	-5.655	-6.656	2	O-GLU138(A)
				O-ILE139(A)
p38 MAPK	-6.366	-11.366	1	O-LYS36(A)

## P91 Effects of environmental particulate matter on bone-marrow mesenchymal stem cells

### Muhammad Abu-Elmagd^1,2^, Gauthaman Kalamegam^1^, Roaa Kadam^1^, Mansour A Alghamdi^3^, Magdy Shamy^3^, Max Costa^4^, Mamdouh I Khoder^3^, Mourad Assidi^1,2^, Peter Natesan Pushparaj^1^, Mamdooh Gari^1^, Mohammed Al-Qahtani^1^

#### ^1^Center of Excellence in Genomic Medicine Research, King Abdulaziz University, Jeddah, Saudi Arabia; ^2^Center of Innovation in Personalized Medicine, King Abdulaziz University, Jeddah, Saudi Arabia; ^3^Department of Environmental sciences, Faculty of Meteorology, Environment and Arid Land Agriculture, King Abdulaziz University, Jeddah, Saudi Arabia; ^4^New York University School of Medicine, Nelson Institute of Environmental Medicine, New York, USA

##### **Correspondence:** Gauthaman Kalamegam (kgauthaman@kau.edu.sa) – Center of Excellence in Genomic Medicine Research, King Abdulaziz University, Jeddah, Saudi Arabia

**Background**

Airborne particulate matter (PM) less than 2.5 μm are classified as ‘fine particles’ while those above are classified as ‘coarse particles’. Fine and coarse PM comprise organic and inorganic substances and both carry inhalational hazard. Suspended PM include but not limited to dust, smoke, and liquid droplets which can cause adverse health effects in humans especially those related to the respiratory and circulatory systems [1]. PM collected from Jeddah increased expression of genes associated with the pathogenesis of metabolic syndrome and atherosclerosis both *in vitro* [1] and *in vivo* [2]. The objective of this study is to assess the *in vitro* effects of PM on bone-marrow mesenchymal stem cells (BM-MSCs) proliferation, death and gene expression profiling.

**Materials and methods**

Dust were collected on Polypropylene filters and immersed in an aqueous/ alcohol extraction followed by sonication. Particles were then lyophilized, weighed and stored at -80 °C. Human BM-MSCs were derived from bone marrow aspirates obtained following Institutional Ethical Committee approval [11-557/KAU]. BM-MSCs cells were plated at a seeding density of 2 × 10^4^ cells/well in a 24 well plate and exposed to two different sizes of PM (2.5 μm and 10 μm) at five different concentrations (15, 25, 20, 150 and 300 μg/mL). Cell morphology and cell proliferation (MTT assay) were carried out.

**Results**

**Fig. 33 (abstract P91):**
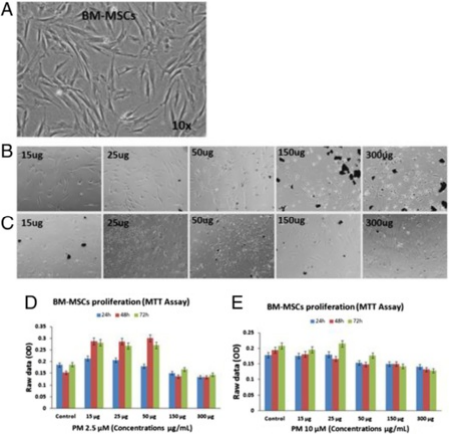
Primary cultures of BM-MSCs (**a**); Morphological changes following treatment with PM [2.5μm (**b**) and 10μm(**c**)] at varying concentrations (15, 25, 50, 150 and 300 μg/mL); cell proliferation following treatment with PM [2.5μm (**d**) and 10μm (**e**)]

Primary cultures of BM-MSCs demonstrated their characteristic spindle shaped morphology (Fig. 33a). Treatment with PM (2.5 μm and 10 μm) at varying concentrations (15, 25, 50, 150 and 300 μg/mL) led to morphological changes which resulted in death of BM-MSCs at higher concentrations (50, 150 and 300 μg/mL) (Fig. 33b, c). Concentration dependent inhibition of BM-MSCs proliferation (MTT assay) were also observed. Mean maximal decreases in proliferations were 19.35 % (150 μg, 24 h) and 28.49 % (300 μg, 24 h) for PM2.5 μm respectively (Fig. 33d). Mean maximal decreases in proliferations were 23.71 % (50 μg, 48 h), 31.73 % (150 μg, 72 h) and 38.46 % (300 μg, 72 h) for PM10μm respectively (Fig. 33e).

**Conclusions**

Particulate matter at concentrations of 2.5 μm and 10 μm inhibit BM-MSCs proliferation *in vitro* leading to cell death. The actual mechanism that caused BM-MSCs cell death in the present study needs further investigations.

**Acknowledgements**

The financial support provided by King Abdulaziz University (KAU), Jeddah, under grant number 4/00/00/252 and the “Sheikh Salem Bin Mahfouz Scientific Chair for Treatment of Osteoarthritis by Stem Cells” which provided the clinical material are greatly acknowledged.

**References:**

1. Sun, H, Shamy, M, Kluz, T, Munoz, AB, Zhong, M, Laulicht, F, Alghamdi, MA, Khoder, MI, Chen, LC, Costa, M, **Gene expression profiling and pathway analysis of human bronchial epithelial cells exposed to air borne particulate matter collected from Saudi Arabia**. *Toxicol. Appl. Pharmacol.* 2012. **265**,147–157.

2. Brocato, J, Sun, H, Shamy, M, Kluz, T, Alghamdi, MA, Khoder, MI, Chen, LC, Costa, M. **Particulate matter from Saudi Arabia induces genes involved in inflammation, metabolic syndrome and atherosclerosis**. *J. Toxicol. Environ. Health Part A*. 2014, **77**, 751–766.

## P92 Distinctive charge clusters in human virus proteomes

### Najla Kharrat^1^, Sabrine Belmabrouk^1^, Rania Abdelhedi^1^, Riadh Benmarzoug^1^, Mourad Assidi^2,3^, Mohammed H. Al Qahtani^2^ and Ahmed Rebai^1^

#### ^1^Centre of Biotechnology of Sfax, Laboratory of Molecular and Cellular Screening Processes, Bioinformatics Group, Po. Box: 1177, 3018, Sfax, Tunisia; ^2^Center of Excellence in Genomic Medicine Research, King Abdulaziz University, Jeddah, Saudi Arabia; ^3^Center of Innovation in Personalized Medicine, King Abdulaziz University, Jeddah, Saudi Arabia

##### **Correspondence:** Ahmed Rebai (ahmed.rebai@cbs.rnrt.tn) – Centre of Biotechnology of Sfax, Laboratory of Molecular and Cellular Screening Processes, Bioinformatics Group, Po. Box: 1177, 3018, Sfax, Tunisia

**Background**

The identification of charge clusters (runs of charged residues) in proteins and their mapping within the sequence is an important step toward a comprehensive analysis of how these particular motifs mediate, via electrostatic interactions, various molecular processes such as protein sorting, translocation, docking, orientation and binding to DNA and to other proteins. Few algorithms that specifically identify these charge clusters have been designed and described in the literature [1]. In this study, 197 distinctive human viral proteomes were screened for the occurrence of charge clusters (CC) using a new computational tool.

**Results**

373 CC have been identified within the 2549 viral protein sequences screened. The number of protein sequences that are CC-free is 2176 (85.3 %) while 150 and 180 proteins contained positive charge (PCC) and negative charge clusters (NCC), respectively. The NCCs (211 detected) were more prevalent than PCC (162). PCC-containing proteins are significantly longer than those having NCCs (*p* = 2. 10^16^). The most prevalent virus families having PCC and NCC were *Herpesviridae* followed by *Papillomaviridae*. However, the single-strand RNA group has on average three times more NCC than PCC. According to the functional domain classification, a significant difference in distribution was observed between PCC and NCC (*p* = 2. 10^-8^) with the occurrence of NCCs being more frequent in C-terminal region while PCC more often fall within functional domains. Only 29 proteins sequences contained both NCC and PCC. Moreover, 101 NCC were conserved in 84 proteins while only 62 PCC were conserved in 60 protein sequences. To understand the mechanism by which the membrane translocation functionalities are embedded in viral proteins, we screened our PCC for sequences corresponding to cell-penetrating peptides (CPPs) using two online databases: *CellPPd* and *CPPpred*. We found that all our PCCs, having length varying from 7 to 30 amino-acids were predicted as CPPs. Experimental validation is needed to improve our understanding of the role of PCCs in viral infection process.

**Conclusions**

Screening distinctive cluster charges in viral proteomes suggested a functional role of these protein regions and might provide potential clues to improve the current understanding of viral diseases.

**References**

1. Belmabrouk S, Kharrat N, Benmarzoug R, Rebai A: **Exploring proteome-wide occurrence of clusters of charged residues in eukaryotes**. *Proteins* 2015.

## P93 ***In vitro*experimental model and approach in identification of new biomarkers of inflammatory forms of arthritis**

### Ghazi Dhamanhouri^1^, Peter Natesan Pushparaj^2^, Abdelwahab Noorwali^1^, Mohammad Khalid Alwasiyah^2,3^, Afnan Bahamaid^2,4^, Saadiah Alfakeeh^2,4^, Aisha Alyamani^2,4^, Haneen Alsehli^5^, Mohammed Abbas^6,7^, Mamdooh Gari^2,6^, Ali Mobasheri^2,8,9^, Gauthaman Kalamegam^2,6^, Mohammed Al-Qahtani^2^

#### ^1^King Fahd Medical Research Centre (KFMRC), King Abdulaziz University, Jeddah, Saudi Arabia; ^2^Centre of Excellence in Genomic Medicine Research (CEGMR), King Abdulaziz University, Jeddah, Saudi Arabia; ^3^Aziziah Maternity and Children Hospital, Jeddah, Saudi Arabia; ^4^Department of Biochemistry, Faculty of Science, King Abdulaziz University, Jeddah, Saudi Arabia; ^5^Center of Innovation in Personalized Medicine, King Abdulaziz University, Saudi Arabia; ^6^Sheikh Salem Bin Mahfouz Scientific Chair for Treatment of Osteoarthritis by Stem Cells, King Abdulaziz University, Jeddah, Saudi Arabia; ^7^Department of Orthopaedic Surgery, Faculty of Medicine, King Abdulaziz University Hospital, Jeddah, Saudi Arabia; ^8^The D-BOARD European Consortium for Biomarker Discovery, The APPROACH Innovative Medicines Initiative (IMI) Consortium, Faculty of Health and Medical Sciences, University of Surrey, Guildford, Surrey, GU2 7XH, UK; ^9^Arthritis Research UK Centre for Sport, Exercise and Osteoarthritis, Arthritis Research UK Pain Centre, Medical Research Council and Arthritis Research UK Centre for Musculoskeletal Ageing Research, University of Nottingham, Queen’s Medical Centre, Nottingham, NG7 2UH, UK

##### **Correspondence:** Gauthaman Kalamegam (kgauthaman@kau.edu.sa) – Sheikh Salem Bin Mahfouz Scientific Chair for Treatment of Osteoarthritis by Stem Cells, King Abdulaziz University, Jeddah, Saudi Arabia

**Background**

Arthritic diseases are major causes of disability in the elderly population throughout the world, including the Middle East and the Kingdom of Saudi Arabia. Osteoarthritis (OA) is a degenerative disease of the synovial joint, where matrix metalloproteinases, inflammatory cytokines and reactive oxygen species orchestrate the cartilage degradation process [1]. As early detection and intervention have better prognosis it is important to identify the expressed biomarkers at various stages of OA. Use of experimental model systems to assay such biomarkers will greatly aid in understanding their role in diagnostic, prognostic and therapeutic management of OA.

**Materials and methods**

Screening for biomarkers in OA was done using nomenclature of signalling molecules and pathways involved in cartilage formation and degradation using Ingenuity Pathway Analysis (IPA) knowledgebase (Ingenuity Systems, Qiagen, USA). Target molecules identified by IPA were further analyzed using Fisher’s Exact Test (P < 0.05) and subjected to core analysis to understand the diseases and biological functions. Human cartilage and synovial fluid were collected following ethical approval [11-557]. Primary cell cultures of synovial fluid MSCs (SF-MSCs) from normal and OA patients were established and characterized. Pharmacologicals/nutraceutics that are known to influence OA will be evaluated at different concentrations on both SF-MSCs and cartilage explants and proteomic analysis performed.

**Results**

**Fig. 34 (abstract P93):**
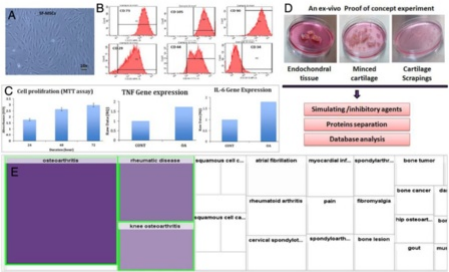
**a** - Primary cultures of SF-MSCs; **b** - SF-MSCs Stemness characterization; **c** - SF-MSCs proliferation and inflammatory gene expression in SF-MSCs from normal and osteoarthritis; **d** - *In vitro* model system and work flow; **e** - IPA analysis of diseases and biological functions in OA

SF-MSCs demonstrated characteristic fibroblastic morphology and these cells were positive for the MSC related CD markers (Fig. 34a, b). SF-MSCs showed slow growth and cell proliferation; and inflammatory related genes (TNF, IL6) were increased in OA (Fig. 34c). Secreted proteins will be analyzed using proteomic depicted workflow (Fig. 34d). IPA analysis core analysis identified predominant molecules (~200) associated with connective tissue disorders, inflammatory diseases and skeletal/muscular disorders (Fig. 34e). These potential targets/biomarkers will be evaluated using primary cultures and explant *in vitro* models.

**Conclusions**

*In vitro* explant and primary cell cultures from controls and OA patients are excellent models for proteomics analysis and IPA prediction analysis enables identification of biomarkers that have diagnostic and prognostic value. Combination of biological and systems analysis helps to identify important molecules of interest for analysis in a cost effect manner.

**Acknowledgements**

The financial support provided by the Deanship of Scientific Research (DSR), King Abdulaziz University (grant no. 1-141/1434 HiCi) and the “Sheikh Salem Bin Mahfouz Scientific Chair for Treatment of Osteoarthritis by Stem Cells” which provided the clinical material are greatly acknowledged.

**References**

1. Clutterbuck AL, Smith JR, Allaway D, Harris P, Liddell S, Mobasheri A**. High throughput proteomic analysis of the secretome in an explant model of articular cartilage inflammation.** J *Proteomics* 2011, **74**(5):704-71

## P94 Molecular docking of GABA_A_ receptor subunit γ-2 with novel anti-epileptic compounds

### Muhammad Faheem^1^, Shilu Mathew^2^, Peter Natesan Pushparaj^2^, Mohammad H. Al-Qahtani^2^

#### ^1^Department of Biochemistry, Faculty of Science, King Abdulaziz University, Jeddah, Saudi Arabia; ^2^Center of Excellence in Genomic Medicine Research, King Abdulaziz University, Jeddah, Saudi Arabia

##### **Correspondence:** Peter Natesan Pushparaj (peter.n.pushparaj@gmail.com) – Center of Excellence in Genomic Medicine Research, King Abdulaziz University, Jeddah, Saudi Arabia

**Background**

An inhibitory neuronal transmission is chiefly mediated through γ-aminobutyric acid (GABA) via GABA_A_ receptors (GABA_A_Rs). The GABA_A_Rs are heteropentameric chloride channel receptors, expressed in neurons, and their deficiency could lead to epilepsy. Therefore, the GABA_A_Rs might be considered as the primary targets against epilepsy. Several antiepileptic drugs, natural, and synthetic compounds have been shown to enhance the GABA_A_Rs action and reduce epileptic seizures [1, 2]. Hence, the objective of present the study is to find out potential binding ligands with anti-epileptic properties against the active sites of GABA_A_R subunit γ-2.

**Materials and methods**

Homology model of GABA_A_R subunit γ-2 has been built, and the docking studies were carried out to deduce the possible binding ligands with anti-epileptic properties against GABA_A_R subunit γ-2. For this purpose, four different plant derived compounds, such as Chrysin, Rutin, Montanine, and Vitexin, were selected. Their quantitative structure-activity relationships have been investigated to find the inhibitory activity of these four compounds.

**Results**

Our results have shown a maximum docking score for Chrysin (79.6174) Kcal/mol along with maximum number of hydrogen bond interactions at the active sites Thr87-O, Phe77-N, and Phe78-N. The other three compounds including Rutin, Montanine, and Vitexin have shown their interactions at active sites Leu29-O, Phe77-N, Arg22-N, respectively.

**Conclusions**

We can conclude that Chrysin could be the best-fit ligand for GABA_A_R subunit γ-2 and might be considered as an alternate treatment for epileptic patients. However, both *in vitro*, and *in vivo* studies are necessary for further characterization and validation to design effective anti-epileptic drugs (AEDs) in the near future.

**References**

**1**. Fritschy JM: **Epilepsy, E/I balance and GABA**_**A**_**receptor plasticity**. *Front Mol. Neurosci* 2008, **1**: 5.

**2**. Faheem M, Chaudhary AG, Kumosani TA, Al-Qahtani MH, Yasir M, Bibi F, Kim MO, Rasool M and Naseer MI. **Interaction of different proteins with GABA**_**A**_**receptor and their modulatory effect on inhibitory neural transmission leads to epilepsy*.****CNS Neurol Disord Drug Targets* 2014; **13(7)**: 1148-1159.

**Fig. 35 (abstract P94):**
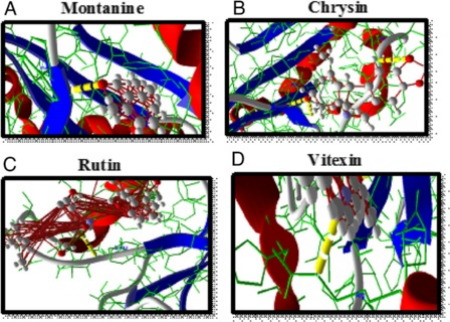
Potential anti-epileptic ligands and their interactions with GABA_A_R subunit γ-2 (a) Montanine, (b) Chrysin, (c) Rutin and (d) Vitexin

## P95 Breast cancer knowledge, awareness, and practices among Saudi females residing in Jeddah

### Shilu Mathew^1^, Muhammad Faheem^2^, Shiny Mathew^3^, Peter Natesan Pushparaj^1^, Mohammad H. Al-Qahtani^1^

#### ^1^Center of Excellence in Genomic Medicine Research, King Abdulaziz University, Jeddah, Kingdom of Saudi Arabia; ^2^Department of Biochemistry, Faculty of Science, King Abdulaziz University, Jeddah, Kingdom of Saudi Arabia; ^3^Department of Multimedia Technology, Karunya University, Coimbatore, India

##### **Correspondence:** Peter Natesan Pushparaj (peter.n.pushparaj@gmail.com) – Center of Excellence in Genomic Medicine Research, King Abdulaziz University, Jeddah, Kingdom of Saudi Arabia

**Background**

Breast cancer is the most common type of cancer among women worldwide. It ranks first amongst cancer in Saudi females with an incidence of 19.8 % [1]. Few studies have shown that the knowledge, awareness, and protective measures against this disease are very low in Saudi females [1, 2, 3]. Objective of this study was to assess the level of awareness, knowledge, and practices of breast cancer among Saudi females living in Jeddah.

**Materials and methods**

This study was conducted through a self-administered 25-questions regarding the level of awareness, knowledge, and practices about breast cancer. 200 females participated in this survey. Questionnaires were distributed hand-by-hand and through the creation of an online survey.

**Results**

Most of the contributors (76 %) were below 40 years, married (92.5 %) or ever married (5.4 %). 64.8 % participants have three or more children, and 9.4 % were without any children. 81.5 % females have regular menstruation, 3.5 % with stopped menstruation, 38 % faced abortion in their life, and 46.5 % have used pills as contraceptives. 82.5 % got information about breast cancer through health professionals, 52.5 % through friends/neighbors, 58.5 % through electronic media, and 61.5 % through print media. 18 % has reported a positive family history of breast cancer, 78.6 % know about the incidence of breast mass, 73 % know about an increase in the neighboring lymph nodes, 68.5 % know about blood discharge from nipple, 66.3 % know about the breast pain, and 43 % know about the nipple retraction. 42.1 % were aware of the effect of hormonal replacement therapy, 56 % know the smoking affect, 43.5 % do not know about breast self-examination (BSE), 26.0 % aware of the procedure but never go for it, and 13.5 % have applied it. Moreover, 80 % never go to the health professionals for breast examination, and 84 % do not know about mammography.

**Conclusions**

The study showed an inadequate knowledge about breast cancer among Saudi females. This study could help to increase the awareness and knowledge of breast cancer in the Saudi society. However, extensive public awareness programs, campaigns, and seminars will be required to significantly reduce the socio-economic burden of this disease in KSA.

**References**

**1.** Ravichandran K, Hamdan NA, Dyab AR: **Population based survival of female breast cancer cases in Riyadh Region, Saudi Arabia**. *Asian Pacific Journal of Cancer Prevention* 2005, **6**: 72-6.

**2. International Agency for Research on Cancer**, Breast cancer statistics. [http://globocan.iarc.fr/factsheet.asp]. 2008.

**3**. Parkin DM, Bray F, Ferlay J, Pisani P: **Global Cancer Statistics**. *CA Cancer J Clin* 2005, **55**: 74-108

## P96 Anti-inflammatory role of Sesamin by Attenuation of Iba1/TNF-α/ICAM-1/iNOS signaling in Diabetic Retinopathy

### Mohammad Sarwar Jamal^1^, Syed Kashif Zaidi^2^, Raziuddin Khan^3^, Kanchan Bhatia^3,4^, Mohammed H. Al-Qahtani^3,4^, Saif Ahmad^3,4^

#### ^1^King Fahad Center for Medical Research, King Abdulaziz University, Jeddah, Saudi Arabia; ^2^Center of Excellence in Genomic Medicine Research, King Abdulaziz University, Jeddah, Saudi Arabia; ^3^Department of Biological Sciences, College of Science and Arts-Rabigh, King Abdulaziz University, Jeddah, Kingdom of Saudi Arabia; ^4^Center of Emphasis in Neuroscience, Texas Tech University Health Science Center, El Paso-79905, Texas, USA

##### **Correspondence:** Saif Ahmad (saabdualazez@kau.edu.sa) – Center of Emphasis in Neuroscience, Texas Tech University Health Science Center, El Paso-79905, Texas, USA

**Background**

Diabetic retinopathy (DR) is one of the leading diabetic vascular complications in eye, which results to the blindness. Inflammation plays significant role in pathophysiology of DR. Earlier reports demonstrated that inflammatory mediators like TNF-α, ICAM-1 and iNOS signaling are implicated in the pathogenesis of DR complications. Hyper activation of retinal microglia in DR could be one of the main causes that trigger excess release of inflammatory cytokines, which further activates MAPKinase casacade that results neuronal degeneration. Sesamin (SES) is the main component of sesame seed and oil, and has been reported as potent antioxidant and neuroprotective. Here, we investigated therapeutic effect of SES as anti-inflammatory in Streptozotocin (STZ) induced diabetic mice model.

**Materials and methods**

Eight weeks post diabetic establishment, mice received SES (30 mg/kg BW, i.p, alternate day) for four weeks. Mice body weight and Blood glucose level was measured. Microglia activation was determined by immunohistochemistry (Iba-1 antibody was used as microglia marker). Retinal mRNA levels of Iba-1, Tumor necrosis factor-α (TNF-α), Inducible nitric oxide synthase (iNOS) and Intercellular Adhesion Molecule 1 (ICAM-1) were examined by real Time-PCR. Western Blot analysis was done to assess the iNOS protein expression level in different group of mice retinal samples.

**Results**

The results showed that SES significantly lowered the progression of diabetic retinal injury by: 1) decreasing blood glucose level, 2) suppressing microglia activation, 3) reducing retinal inflammatory mediators TNF-α and ICAM-1 levels and 4) quenching iNOS expression.

**Conclusions**

In conclusion, our results suggested that SES could be of therapeutic benefit in slowing the progression of DR by ameliorating hyperglycemia and inflammation in diabetic retina.

**Acknowledgements**

This work was supported by the Deanship of Scientific Research (DSR; D1435-573-662), King Abdulaziz University, Jeddah, Kingdom of Saudi Arabia. Authors gratefully acknowledge technical and financial support of DSR.

## P97 Identification of drug lead molecule against vp35 protein of Ebola virus: An *In-Silico* approach

### Iftikhar AslamTayubi^1^, Manish Tripathi^2^, Syed Asif Hassan^1^, Rahul Shrivastava^2^

#### ^1^Faculty of Computing and Information Technology, King Abdulaziz University, Rabigh-21911, Kingdom of Saudi Arabia; ^2^Department of Biological Sciences and Engineering, Maulana Azad National Institute of Technology Bhopal 462051, India

##### **Correspondence:** Iftikhar AslamTayubi (iftikhar.tayubi@gmail.com) – Faculty of Computing and Information Technology, King Abdulaziz University, Rabigh-21911, Kingdom of Saudi Arabia

**Background**

Current global scenario faced with an infectious disease crisis, which has been anticipated for decades. The fatal filoviruses, Ebola and Marburg is one of the utmost virulent pathogens, which causes adverse viral hemorrhagic fever in humans and non-human primates and has higher fatality rates up to 90 % have been reported [1]. There are no approved vaccine or therapeutics described to counter filovirus infection. Mortality after their infection is the result of severe bleeding and multi-organ failure. The genome of Ebola virus (EBOV) encodes a single polypeptide with enzymatic activity, viral large (L) RNA-dependent RNA polymerase protein. Presently, with fewer evidence is added about the L protein due to which it hindered the development of antivirals. Viral protein 35 (VP35) is capable of copping dsRNA and plays important roles in viral replication, pathogenesis and innate immune response. Therefore, antifiloviral therapeutic efforts must comprise added targets for the development of vaccines to counter their infection [2,3]. In the current study, structure-based in silico screening method can be used to recognize and characterize the small molecules targeting the binding pocket within VP35 glycoprotein.

**Materials and methods**

**Fig. 36 (abstract P97):**
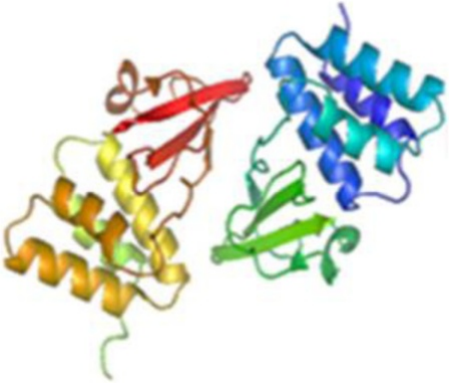
Vp35 protein PDB ID 3FKE

Proteins selected for the present study are VP35 glycoprotein PDB ID: 3FKE (Fig. 36), whose 3D structure was acquired from PDB [http://www.rcsb.org/pdb/] with resolution range 1.40 Ǻ. Antiviral compounds used for virtual screening were taken from PubChem database. The active site residues in the target proteins were determined by using CastP. Ligand preparation was done using Discovery studio software and their binding affinity with target components were analysed using Autodock 4.0.

**Results**

Based on the binding affinity, we conclude that antiviral compound perfectly blends with and ALA-291, PRO-292 amino acid residues involved in this binding and their binding energy are -4.48 Kcal/mole. Results indicate that Idoxuridine can be used as best antiviral compounds against Ebola virus. The outcome helps and provides an early outline for the development of antifiloviral compounds against VP35 proteins.

**Conclusions**

Results indicate that Idoxuridine can be used as best antiviral compounds against Ebola virus. The outcome helps and offers an early framework for the development of antifiloviral compounds against VP35 proteins.

**References**

1. Feldmann H, Geisbert TW: **Ebola haemorrhagic fever**. *Lancet* 2011, **377**:849–62.

2. Leung DW, Ginder ND, Fulton DB, Nix J, Basler CF, Honzatko RB, et al: **Structure of the Ebola VP35 interferon inhibitory domain**. *Proc Natl Acad Sci USA* 2009, **106**:411–6.

3. Leung DW, Shabman RS, Farahbakhsh M, Prins KC, Borek DM, Wang T, et al: **Structural and functional characterization of Reston Ebola VP35 Interferon Inhibitory Domain**. *J Mol Biol* 2010, **399** (3): 347-57.

## P98 An approach to personalized medicine from SNP-calling through disease analysis using whole exome-sequencing of three sub-continental populations

### Iftikhar A Tayubi, Syed Hassan, Hamza A.S Abujabal

#### Faculty of Computing and Information Technology, King Abdulaziz University, Rabigh, Saudi Arabia

##### **Correspondence:** Iftikhar A Tayubi (iftikhar.tayubi@gmail.com) – Faculty of Computing and Information Technology, King Abdulaziz University, Rabigh, Saudi Arabia

**Background**

The protein coding genes consist only approx 1 % of the human genome, which anchorage 85 % of the mutations with large effects on disease-related traits. The effective approaches for the selective sequencing of completely coding regions, or “whole exome” have a possible contribution to the understanding human diseases [1]. Exome-sequencing is a cost-effective and innovative tools for dissecting the genetic basis of diseases [2]. These progresses have also set the stage for applying exome- and whole-genome sequencing to simplify clinical diagnosis and personalized medicine. Here we have performed SNP, INDEL profiling and deduce their functional role by using whole exome-sequencing of a wide range of Human populations from HapMap projects.

**Materials and methods**

The Data set were acquired from NCBI Short Reads under ID SRP004054**.**There are total 93 exomes data available from African and American populations sequenced under HapMap project.The 22 samples (9 exome samples from Asian, 6 exome samples from American and 5 exome samples from African population) were considered for the computational study. Genes containing the novel variations were determined via Genome analysis Toolkit [3].

**Results**

**Fig. 37 (abstract P98):**
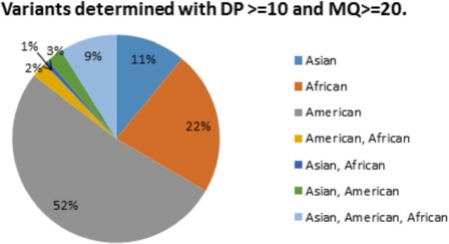
Depicting the percentage of variants determined and studied across the different subsets formed from the exome data of the three populations

Around 15410 genes exhibited novel variants across the sample groups originating from the three populations, primarily from Asian, African and American ethnicity (Fig. 37), 425 novel SNPs and 264 novel INDELS were determined across the three populations (Table 20). Further, 20,037 variants were observed in the nucleus region, whereas 22,154 genomic variants were found in the cytoplasm region. Genomic variants are responsible for the individual differences and hence the novel variants determined via the study will help in understanding their impact on biological processes.

**Conclusions**

The calling resulted in 105,050 novel variants across the three populations. The novel variants determined via the computational analysis is significant for decoding the interference and role of the SNPs and INDELs on the genes and their related biological functions.

**References**

1. Iftikhar Aslam Tayubi, Ahmad Firoz, Omar M. Barukab, Adeel Malik: **Identification of hub genes and their SNP analysis in West Nile virus infection for designing therapeutic methodologies using RNA-Seq data.***Genes Genom* 2015, **37**(8):679–691.

2. Bamshad MJ, Ng SB, Bigham AW, Tabor HK, Emond MJ, Nickerson DA, Shendure J: **Exome-sequencing as a tool for Mendelian disease gene discovery.***Nat Rev Genet* 2011, 12(11):745-55.

3. McKenna A, Hanna M, Banks E, Sivachenko A, Cibulskis K, Kernytsky A, Garimella K Altshuler D, Gabriel S, Daly M, DePristo MA: **The Genome Analysis Toolkit: a MapReduce framework for analyzing next-generation DNA sequencing data.***Genome Res* 2010, **20**(9):1297-303.

**Table 20 (abstract P98):** Denoting the number of genomic variants (including SNPs and INDELs) determined across the three populations, namely Asian, African and American considered for the study

Combination	No. of SNP	Novel SNP	No. of Indel	Novel Indel
Asian	58194	1078	6231	957
African	115886	17320	14723	7061
American	287662	66197	17148	7128
American, African	13042	3198	1072	564
Asian, African	3793	109	439	138
Asian, American	13903	440	1029	171
Asian, American, African	48127	425	3641	264

## P99 Low versus high frequency of Glucose –6 – Phosphate Dehydrogenase (G6PD) deficiency in urban against tribal population of Gujarat – A signal to natural selection

### Ishani Shah^1^, Bushra Jarullah^1^, Mohammad S Jamal^2^, Jummanah Jarullah^2^

#### ^1^Department of Biotechnology, KadiSarvaVishwavidhyalaya, Gandhinagar, Gujarat, India; ^2^King Fahd Medical Research Center, King Abdulaziz University, Jeddah, Saudi Arabia

##### **Correspondence:** Bushra Jarullah (bjarullah@yahoo.com) – Department of Biotechnology, KadiSarvaVishwavidhyalaya, Gandhinagar, Gujarat, India

**Abstract**

**Background**

An important parameter for human genetic variation is known to be natural selection. Natural selection is a theory that states that individuals with certain genotypes best adapted to live in an area are more likely than other individuals to survive and reproduce. Natural selection is however interlinked to genetic drift and gene flow. Interplay amongst these influences evolution in natural populations. Directional selection leads to increase over time in the frequency of a favoured allele. Previous reports suggest that G6PD-deficient alleles show some signatures of selection. Glucose-6- Phosphate Dehydrogenase (G6PD) deficiency is the most common enzyme deficiency of human erythrocyte which affects more than 400 million people worldwide. Previous studies have reported prevalence of G6PD deficiency ranging from 0 % to 27 % amongst various castes, ethnic and linguistic groups in India. Although few studies have reported the prevalence of G6PD deficiency in populations of Gujarat these studies are limited to specific castes and regions of Gujarat. There is no comprehensive information available about the prevalence of this disease across the entire map of Gujarat. The focus of the present study was therefore to determine the prevalence of G6PD deficiency in population of Gujarat. Survey of 3467 samples suspected to be G6PD deficient frequenting the hospitals and leading laboratories across Gujarat were analyzed to confirm the deficiency.

**Results**

It was interesting to find drastic variation in the prevalence amongst the tribal and urban population. Frequency varied from as high as from 11.18 % in tribal populations to as low as 1.2 % in the urban population. Urban areas such as Kutch, Bhuj, Lunawada and Kapadwanj showing relatively high prevalence have been known to be inhabited by tribal population.

**Conclusions**

Heterozygosity levels, linkage disequilibrium patterns and frequencies of alleles segregating in a population play a vital role in the prevalence of any genetic deficiency. However how this polymorphism is being maintained is yet to be deciphered. Our study signals the need for rigorous research to understand the pattern of natural selection and establishment of selection coefficients for the different genotypes.

## P100 Spontaneous preterm birth and single nucleotide gene polymorphisms: a recent update

### Ishfaq A Sheikh^1^, Ejaz Ahmad^1^, Mohammad S Jamal^1^, Mohd Rehan^1^, Muhammad Abu-Elmagd^2^, Iftikhar A Tayubi^3^, Samera F AlBasri^4^, Osama S Bajouh^4^, Rola F Turki^4,5^, Adel M Abuzenadah^2,5^, Ghazi A Damanhouri^1^, Mohd A Beg^1^, Mohammed Al-Qahtani^2^

#### ^1^King Fahd Medical Research Center, King Abdulaziz University, Jeddah, Kingdom of Saudi Arabia; ^2^Center of Excellence in Genomic Medicine Research, King Abdulaziz University, Jeddah, Kingdom of Saudi Arabia; ^3^Faculty of Computing and Information Technology, King Abdulaziz University, Rabigh, Kingdom of Saudi Arabia; ^4^Department of Obstetrics and Gynecology, Faculty of Medicine, King Abdulaziz University, Jeddah, Kingdom of Saudi Arabia; ^5^KACST Technology Innovation Center in Personalized Medicine, King Abdulaziz University, Jeddah, Kingdom of Saudi Arabia

##### **Correspondence:** Mohd A Beg (mabeg51@gmail.com) – King Fahd Medical Research Center, King Abdulaziz University, Jeddah, Kingdom of Saudi Arabia

**Background**

Preterm birth (PTB), birth before the completion of 37 weeks of gestation, is a significant global public health problem. About 15 million babies are born preterm each year accounting for more than a million deaths of children. During last two decades (1990–2010), PTB rate has increased in almost all 65 countries for which consistent data are available. Preterm neonates are more prone to low blood sugar, jaundice, sepsis, intensive care hospitalization, pulmonary dysfunction, ophthalmological disorders, and long-term neurocognitive deficits. Surviving children also encounter health problems more frequently in early adulthood and even continuing into the next generation. Global Burden of Disease estimates show that PTB accounts for 3.1 % of all Disability Adjusted Life Years, higher than HIV and malaria. Majority (70 %) of PTBs are spontaneous. About half of these are without any apparent cause and other half assigned to an increasing number of risk factors. Risks include behavioral, sociodemographic, genetic, medical, environmental, and biological factors that individually or in combination interact during pregnancy resulting in PTB.

**Materials and methods**

A comprehensive evaluation of published studies on single nucleotide polymorphisms (SNPs) conferring potential risk for PTB was done by performing a targeted PubMed search for association of SNPs and PTB for the years 2007-2015 and systematically reviewing all relevant studies.

**Results**

Our evaluation resulted in more than 150 studies identifying about 120 candidate genes with SNPs that have potential association with PTB. These genes were related to diverse tissues including endocrine, tissue remodeling, vascular, metabolic, immune, and inflammatory systems. The majority of the potential associations were for the inflammation related genes. Many of the polymorphisms exhibited inconsistency and remained inconclusive.

**Conclusions**

The challenge of the PTB is to identify high risk women and provide them a personalized medical care for reducing the burden of PTB. Recent studies on functional candidate gene variants and their association with PTB have thrown forward a large number of potential predisposing genes. Inconsistencies in different studies preclude pinpointing of any definitive targets. Understanding the complex genomic landscape of PTB needs lateral thinking and multicenter studies using high-throughput approaches.

## P101 Prevalence of congenital heart diseases among Down syndrome cases in Saudi Arabia: role of molecular genetics in the pathogenesis

### Sahar AF Hammoudah^1,2^, Khalid M AlHarbi^2^, Lama M El-Attar^2,3^, Ahmed MZ Darwish^4^

#### ^1^Department of Clinical and Chemical Pathology, Faculty of Medicine, Tanta University, Tanta, Egypt; ^2^Cardiogenetic Team, Department of Pediatrics, College of Medicine, Taibah University, AL Madinah , Saudi Arabia; ^3^Department of Human Genetics, Medical Research Institute, Alexandria University, Alexandria Egypt; ^4^Department of Cardiology, Faculty of Medicine, Tanta University, Tanta, Egypt

##### **Correspondence:** Sahar AF Hammoudah (saharfathi2011@yahoo.com) – Cardiogenetic Team, Department of Pediatrics, College of Medicine, Taibah University, AL Madinah , Saudi Arabia

**Background**

Genetic and congenital disorders are the main causes of increased infant/child death, illnesses and handicap among Arab population. Congenital heart diseases (CHDs) represent a major category of world-wide birth defects. We had searched MEDLINE databases, clinical-science journals and reports from the earlier reviews. We used the search term “Congenital heart diseases”, “Saudi Arabia”, “Down syndrome”, “Molecular genetics”.

**Results**

CHDs were reported in about 6–13 per 1000 live births and implicated in increased incidence of early childhood mortality. CHDs represent one of the most important health problems in Saudi Arabia. CHDs occur in about 50 % of neonates born with Down Syndrome (DS) and almost 40 % of the DS children who survive. Increased incidence of CHDs in children with DS in certain populations has been reported to be associated with widespread consanguinity. Exploration of genes and molecular genetic pathways involved in heart development greatly helps to understand the genetic basis of CHDs. Genetic variations that have not been identified up-till now might contribute to this complex genetic disorder.

**Conclusions**

This study aims to highlight the importance of CHDs as a major health problem in Saudi Arabia and to emphasize the role of molecular genetics in the pathogenesis of CHDs in children with DS.

## P102 Combinatorial efficacy of specific pathway inhibitors in breast cancer cells

### Sara M Ibrahim^1^, Ashraf Dallol^2,^ Hani Choudhry^1,2^, Adel Abuzenadah^2^, Jalaludden Awlia^1^, Adeel Chaudhary^3^, Farid Ahmed^3^, Mohammed Al-Qahtani^3^

#### ^1^Department of Biochemistry, Faculty of Science, King Abdulaziz University, Jeddah, Saudi Arabia; ^2^Centre for Innovation in Personalized Medicine, King Abdulaziz University, Jeddah, Saudi Arabia; ^3^Centre for Excellence in Genomic Medicine Research, King Abdulaziz University, Jeddah, Saudi Arabia

##### **Correspondence:** Farid Ahmed (fahmed1@kau.edu.sa) – Centre for Excellence in Genomic Medicine Research, King Abdulaziz University, Saudi Arabia

**Background**

Breast Cancer (BC) is the most frequent cancer in women, producing the second highest mortality globally. Although initially effective, current treatments fail to prevent eventual resistance and relapse of the disease. Recently, the concept of oncogenic addiction has gained immense significance, wherein the cells excessively rely on particular pathways for malignancy and resistance[1]. Several single molecule inhibitors, affecting different aspects of neoplasticity, are rapidly entering clinical trials and being explored for their therapeutic relevance. However, from trials using single targeted agents, it is becoming evident that often single target agents are not sufficient and multiple components need to be targeted to disrupt the neoplastic pathways in cells[2]. The aim of this work was to study the effect of novel drug combinations targeting multiple members of signaling pathways in BC cell lines.

**Materials and methods**

All small molecule inhibitors were purchased from Selleckchem (Munich, Germany). The cytotoxicites of individual drugs were assessed using Cell Titer Blue assay (Promega) on MCF-7 cells in quadruplicates. Fluorescence was measured on SpectraMax i3 MiniMax 300. The IC_50_ values for each drug was determined using Graphpad Prism 6. Two drugs combination experiments were perfomed at IC_50_ value for each drug with dose ranges above and below IC_50_ as described by Chou and Talalay [3]. Combination Index (CI) values were calculated using compusyn software. CI = 1 indicates additive effect, CI < 1 indicates synergism, CI > 1 indicates antagonism.

**Results**

Here we report the observation of additive, but not synergistic effect on combination of curcumin, a BCL2 inhibitor and PP242, an active site mTOR inhibitor with CI of 1.06 (ED75), 1.00 (ED90) and 0.97138 (ED95). The combination of PP242 with BH3 mimetic inhibitors of Bcl-2 such as ABT-199 and ABT-737 showed antagonism with CI > 1 at all doses studied.

**Conclusions**

Combination treatment with curcumin and PP242 exerts an additive antitumoral effect on MCF-7 cells. Alterations in the signaling pathways that results in additive effects are currently being investigated. Furthermore, we aim to test more combinations in our lab, targeting complementary pathways, that can potentially diffuse the robust malignant signaling in BC.

**Acknowledgements**

This work was supported by generous funds from the National Plan for Science, Technology and Innovation (MAARIFAH), King Abdulaziz City for Science and Technology, Kingdom of Saudi Arabia – award number (09-BIO-693-03).

**References**

1. Goncalves R, Warner WA, Luo J, Ellis MJ: **New concepts in breast cancer genomics and genetics**. *Breast Cancer Res* 2014, **16**(5):460.

2. Papadatos-Pastos D, De Miguel Luken MJ, Yap TA: **Combining targeted therapeutics in the era of precision medicine**. *Br J Cancer* 2015, **112**(1):1-3.

3. Chou TC, Motzer RJ, Tong Y, Bosl GJ: **Computerized quantitation of synergism and antagonism of taxol, topotecan, and cisplatin against human teratocarcinoma cell growth: a rational approach to clinical protocol design**. *Journal of the National Cancer Institute* 1994, **86**(20):1517-1524.

## P103 MiR-143 and miR-145 cluster as potential replacement medicine for the treatment of cancer

### Mohammad A Jafri, Muhammad Abu-Elmagd, Mourad Assidi, Mohammed Al-Qahtani

#### Center of Excellence for Genomic Medicine Research, King Abdulaziz University, Jeddah, Saudi Arabia

##### **Correspondence:** Mohammad A Jafri (smajaffrey@gmail.com) – Center of Excellence for Genomic Medicine Research, King Abdulaziz University, Jeddah, Saudi Arabia

**Abstract**

**Background**

In cancer, oncogenes or tumor suppressor genes lose or gain vital functions leading to the aberrant expression of oncogenic pathways and malignant transformation. The treatment of cancer is extremely difficult due to inherent complexity of the disease as well as limitations of current chemotherapy and target-based anticancer drugs in terms of toxicity and resistance development. Therefore, novel therapeutic approaches are required. MicroRNAs (miRNA) have emerged as master regulator of gene expression in cells. Their deregulation is associated with cancer initiation and progression. Downregulated miRNAs may be delivered to tumors in order to repair lost gene expression. In this review we highlight the specific role of miR-143 and miR-145 in various cancers and their downstream targets. We also critically evaluate them as possible RNA medicine for the treatment of cancer in the light of recent reports.

**Results**

MicroRNAs regulate various cancer-specific processes including angiogenesis, invasion, migration, apoptosis, metastasis, and chemo-resistance. MicroRNAs can function as either tumor suppressors or oncogenes. The tumor suppressor miRNAs are usually down-regulated in cancer. The observation that certain miRNAs acting as tumor suppressors are downregulated in many cancer types has led to the concept of miRNA replacement therapy. The downregulation of miRNAs could be overcome by introducing exogenous synthetic oligonucleotides known as miRNA mimics to restore the lost gene regulatory network and signaling pathways. MiRNA mimics may be delivered to the cells by using modern advanced delivery techniques. Mir-143 and miR-145 encoding genes, located on chromosome 5 position 33 as a cluster, are co-transcribed to regulate a variety of cellular pathways. These two miRNAs have been reported to be regularly downregulated in many cancer types including breast, bladder, pancreatic, prostrate and colorectal cancer and act as tumor suppressor through inhibition of various downstream targets.

**Conclusions**

While there is a substantial amount of evidence that suggests a possible use of miR-143 and -145 for combination replacement therapy in cancers in which both miRNAs are downregulated but recent reports also have revealed that they can promote tumor growth by stimulating cell proliferation. Therefore, a cautious approach is required to use them as therapeutic intervention in cancer.

## P104 Metagenomic profile of gut microbiota during pregnancy in Saudi population

### Imran khan^1^, Muhammad Yasir^2^, Esam I. Azhar^2,3^, Sameera Al-basri^4^, Elie Barbour^5^, Taha Kumosani^1^

#### ^1^Biochemistry Department, Faculty of Science, King Abdulaziz University, Jeddah, Saudi Arabia; ^2^Special Infectious Agents Unit, King Fahd Medical Research Center, King Abdulaziz University, Jeddah, Saudi Arabia; ^3^Medical Laboratory Technology Department, Faculty of Applied Medical Sciences, King Abdulaziz University, Jeddah, Saudi Arabia; ^4^Department of Obstetrics & Gynaecology, King Abdul Aziz University Hospital, Jeddah, Saudi Arabia; ^5^Faculty of Agriculture, American University of Beirut, Beirut, Lebanon; Adjunct to Biochemistry Department, King Abdulaziz University, Jeddah, Saudi Arabia

##### **Correspondence:** Muhammad Yasir (yasirkhattak.mrl@gmail.com) – Special Infectious Agents Unit, King Fahd Medical Research Center, King Abdulaziz University, Jeddah, Saudi Arabia

**Background**

Pregnant women often suffer with gastrointestinal problems [1] and gut microbiota is one of the contributing factors [2]. The gut microbiota variate with geography, diet, gender and age [3]. Any aberration in the natural composition of gut microbiota can lead to obesity, high risk of infections, inflammation and miscarriages [2]. In this study, Illumine MiSeq deep sequencing was carried out to analyze gut microbiota composition and richness during pregnancy among Saudi females. Statistical, alpha and beta diversity analysis, were performed to identify pregnancy-induced changes in gut microbiota of local population.

**Results**

**Fig. 38 (abstract 104):**
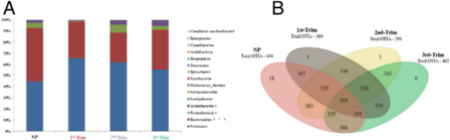
**a** Average percentile densities of detected phyla **b** Venn and Euler diagrammatic representation of shared and unique operational taxonomic unites (OTUs) among the control non-pregnant and pregnant groups of different trimesters.

**Fig. 39 (abstract 104):**
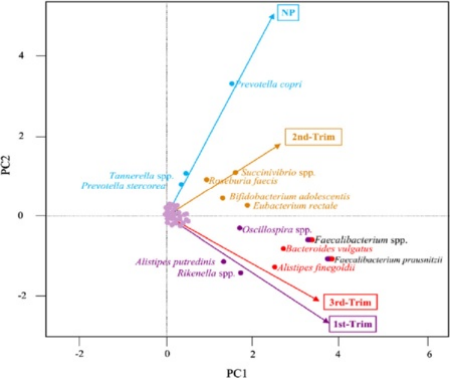
Multivariate principal coordinate analysis of species enrichment. The total variance is 3.594 which is assigned to PC1 = 2.8017, PC2 = 0.4242, PC3 = 0.2207 and PC3 = 0.1472. NP stands for non-pregnant group; 1^st^-Trim stands for 1^st^ trimester; 2^nd^-Trim stands for 2^nd^ trimester and 3^rd^-Trim stands for 3^rd^ trimester.

Around 2.971 million filtered sequences were obtained that were coded for 17 bacterial phyla, 98 families, 230 genera and 454 different bacterial species. The phyla *Firmicutes*, *Proteobacteria, Bacteriodetes* and *Actinobacteria* are dominating healthy Saudi women that substantially modulate during pregnancy*.* The phyla *Firmicutes* and *Actinobacteria* enriched with pregnancy whereas *Bacteroidetes* significantly decreased during second (p = 0.008) and thrid trimester (p = 0.037) (Fig. 38a). The *Prevotellaceae* is the second most dominant family that significantly (p ≤ 0.05) reduced with pregnancy. Among the dominant species (density > 2 %), *Prevotella copri* significantly (p ≤ 0.01) decreased. The species *Faecalibacterium prausnitzii* and *Faecalibacterium* sp*.* enriched with pregnancy. Additionally, third trimester significantly (p ≤ 0.05) enriched with *Bacteroides vulgatus* and *Alistipes finegoldii* (Fig. 39). The statistical analysis indicated that species diversity significantly decreased in 1^st^ trimester (p = 0.01), second and third trimesters (p = 0.05) (Fig. 38b).

**Conclusions**

Pregnancy significantly modulated gut microbiota structure and diversity. The dominant species remained constant with modulated richness among the groups.

**Acknowledgements**

The authors are thankful to King Abdulaziz city of science and technology for supporting this work under the grant no. 84-34-ط آ.

**References**

1. O’Brien B, Zhou Q: **Variables related to nausea and vomiting during pregnancy**. *Birth* 1995, **22**:93-100.

2. Khan I, Yasir M, Azhar EI, Kumosani T, Barbour EK, Bibi F, Kamal MA: **Implication of gut microbiota in human health.***CNS Neurol Disord Drug Targets* 2014, **13**(8):1325-33.

3. Yatsunenko T, Rey FE, Manary MJ, Trehan I, Dominguez-Bello MG, Contreras M, Magris M, Hidalgo G, Baldassano RN, Anokhin AP, Heath AC, Warner B, Reeder J, Kuczynski J, Caporaso JG, Lozupone CA, Lauber C, Clemente JC, Knights D, Knight R, Gordon JI: **Human gut microbiome viewed across age and geography**. *Nature* 2012, **486**(7402):222-7.

## P105 Exploration of anticancer targets of selected metabolites of *Phoenix dactylifera L.* using systems biological approaches

### Fazal Khan^1,2,3^, Gauthaman Kalamegam^1^, Peter Natesan Pushparaj^1^, Adel Abuzenada^1,2^, Taha Abduallah Kumosani^3^, Elie Barbour^4^

#### ^1^Center of Excellence in Genomic Medicine Research, King Abdulaziz University, Jeddah, Saudi Arabia; ^2^Center of Innovation in Personalized Medicine, King Fahd Medical Research Center, King Abdulaziz University, Jeddah, Saudi Arabia; ^3^Biochemistry Department, Faculty of Science; Production of Bioproducts for Industrial Applications Research Group; and Experimental Biochemistry Unit, King Fahd Medical Research Center King, Abdulaziz University, Jeddah, Saudi Arabia; ^4^Department of Agriculture, Faculty of Agricultural and Food Sciences, American University of Beirut (AUB), Beirut, Lebanon; adjuncted to Biochemistry Department, King Abdulaziz University, Jeddah, Saudi Arabia

##### **Correspondence:** Gauthaman Kalamegam (kgauthaman@kau.edu.sa) **–** Center of Excellence in Genomic Medicine Research, King Abdulaziz University, Jeddah, Saudi Arabia

**Background**

Cancer is one of the major cause of death world-wide. Wide spectrum of anticarcinogenic agents exists including synthetic chemicals, natural compounds, small molecules and stem cells. Natural compounds have great appeal as they are both cost-effective and have less or no side effects. Camptothecin, cisplatin, quercetin and etoposide are some of the plant derived potential anticancer agents for cancer treatment [1]. Phoenix dactylifera L. (Date fruit) is claimed to have medical benefits such as hepatoprotective, nephroprotective, antioxidant, anti-inflammatory and anticancer properties. These beneficial properties may be due to the presence of flavonoids, carotenoids, phytosterols, polyphenols, β-D-glucans, procyanidins and anthocyanidins [2]. We attempt to screen some of the important metabolites of date fruit using in silico analysis to identify potential targets which may develop into novel therapeutics.

**Materials and methods**

Based on the literature search eight important compounds that are present in date fruits namely luteolin, β (1 → 3) D-glucan, apigenin, carotenoids, lutein, proanthocyanidins, stigmasterol were selected for exploration of molecular targets utilizing Ingenuity Pathway Analysis (IPA) (Ingenuity System, Qiagen, USA). Furthermore, we performed core analysis of the molecules (Fisher’s Exact Test, P < 0.05) regulated by these compounds by IPA to decipher top canonical pathways, diseases and biological functions as well as toxicological functions.

**Results**

**Fig. 40 (abstract P105):**
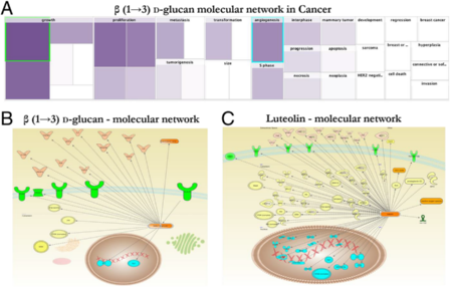
**a** Molecular network of β (1 → 3) D-glucan with p value differentiation; **b** Molecular network of targeted genes of β (1 → 3) D-glucan; **c** Molecular network of targeted genes of Luteolin

IPA analysis revealed targeted proteins and pathways related to cancer signaling (Fig. 40a). Especially the luteolin, quercetin and β (1 → 3) D-glucan share common target and pathways controlling antioxidant system (SOD, CAT, glutathione and NOS), cell growth and differentiation (TGFβ, MAPK, ERK, AKT, PI3K & VEGF), apoptosis (bax, bcl2, p53, TNF-alfa, caspases) and metastasis (MMP1, 2, 9 & 13)(Fig. 40b, c). Carotenoids and lutein mainly are involved with reactive oxygen species management. Proanthocyanidins are identified to target developmental, differentiation (MAPK, Jnk) and autophagy (ATG5, ATG7) related genes.

**Conclusions**

IPA analysis of important metabolites of date fruit targeted specifically the cell growth and differentiation pathways including the process of metastasis and apoptosis. Secondary metabolites of date fruit may thus have anticancer properties which need further validation using biological systems. Given the nutritional benefits of date fruits, their daily intake may additionally provide a prophylactic or synergistic effects against cancers.

**Acknowledgements**

This financial support by King Abdulaziz City for Science and Technology (KACST) through postgraduate grant funding [AT-34-237] is greatly acknowledged.

**References**

1. Newman DJ and Cragg GM. **Natural products as sources of new drugs over the 30 years from 1981 to 2010. Journal of natural products**, 2012. **75**(3); 311-335.

2. Manjeshwar Shrinath Baligaa, Bantwal Raghavendra Vittaldas Baligab, Shaun Mathew Kandathilc, Harshith P. Bhatd, Praveen Kumar Vayalile. **A review of the chemistry and pharmacology of the date fruits (Phoenix dactylifera L.).***Food Research International*, 2011. **44**(7): 1812-1822.

## P106 CD226 and CD40 gene polymorphism in susceptibility to Juvenile rheumatoid arthritis in Egyptian patients

### Heba M. EL Sayed^1^, Eman A. Hafez^2^

#### ^1^Clinical Pathology Department, Mansoura University, Faculty of Medicine, Mansoura, Egypt; ^2^Rheumatology and Rehabilitation Department, Mansoura University, Faculty of Medicine, Mansoura, Egypt

##### **Correspondence:** Heba M. EL Sayed (mosaad_heba@yahoo.com) – Clinical Pathology Department, Mansoura University, Faculty of Medicine, Mansoura, Egypt

**Background**

Juvenile idiopathic arthritis (JIA) is the most common rheumatic disease of the childhood with a high risk of disability in Egyptian children. JIA has many genetic factors affecting its pathogenesis including CD226 and CD40 genes. These genetic factors vary in different races proved by previous studies on European and Chinese populations. The association between JIA and CD226 and CD40 is yet to be evaluated in non-European populations including Egypt. So we studied the association of CD226 rs1883832 (-1C > T) and CD40 rs1883832 (-1C > T) gene polymorphism and disease susceptibility and severity of in an Egyptian cohort.

**Subjects and methods**

In this case control study we recruited 79 Egyptian children with JIA and 93 healthy controls. We studied CD226 rs763361 (C > T) using the tetra amplification refractory mutation system - polymerase chain reaction assay (ARMS-PCR) for detection of polymorphism while for CD40 rs1883832 (-1C > T) we used restriction fragment length polymorphism (RFLP).

**Results**

The statistical results showed that the rs763361 (C > T) SNP in the CD226 gene is significantly associated with JIA group as regard to TT genotypes (p = 0.0001). The frequency of the T allele was significantly higher in JIA patients in comparison with the control group (p = 0.0001). Also this allele was significantly higher in patients with moderate and sever JIA when compared to controls (p = 0.003). This allele correlated to the disease severity (OR = 2.4). Study of CD40 rs1883832 (-1C > T) showed that the distribution of the C allele was significantly higher in JIA patients (p = 0.003). Also it was significantly higher in patients with moderate and sever JIA when compared to controls (p = 0.01).

**Conclusions**

These results demonstrate a genetic association between the CD226 and CD40 gene polymorphism and JIA with an impact on disease severity in an Egyptian cohort.

**References**

1-Lee YH, Bae SC, Song GG: Association between the CTLA-4, CD226, FAS polymorphisms and rheumatoid arthritis susceptibility: a meta-analysis. *Hum Immunol.* 2015 Mar;76(2-3):83-9.

2-Hashemi M, Moazeni-Roodi AK, Fazaeli A, Sandoughi M, Taheri M, Bardestani GR, et al.: **The L55M polymorphism of paraoxonase-1 is a risk factor for rheumatoid arthritis.***Genet Mol Res.* 2010 Aug;9(3):1735–41.

## P107 Paediatric exome sequencing in autism spectrum disorder ascertained in Saudi families

### Hans-Juergen Schulten^1,2^, Aisha Hassan Elaimi^1,2^, Ibtessam R Hussein^1,2^, Randa Ibrahim Bassiouni^3^, Mohammad Khalid Alwasiyah ^1,4^, Richard F Wintle^5^, Adeel Chaudhary^1,2^, Stephen W Scherer^5^, Mohammed Al-Qahtani^1^

#### ^1^Center of Excellence in Genomic Medicine Research, King Abdulaziz University, Jeddah, Saudi Arabia; ^2^KACST Technology Innovation Center in Personalized Medicine, King Abdulaziz University, Jeddah, Saudi Arabia; ^3^Children Hospital, Genetics Department, Taif, Saudi Arabia; ^4^Aziziah Maternity and Children Hospital, Jeddah, Saudi Arabia; ^5^The Centre for Applied Genomics, The Hospital for Sick Children, Toronto, Canada

##### **Correspondence:** Hans-Juergen Schulten (hschulten@kau.edu.sa) – KACST Technology Innovation Center in Personalized Medicine, King Abdulaziz University, Jeddah, Saudi Arabia

**Background**

Autism spectrum disorder (ASD) is a group of multifactorial neurodevelopmental conditions resulting in mental disability. Estimated prevalence of ASD in Arab countries varies widely between 1.4 and 29 per 10,000 children. Early diagnosis and intervention, can substantially improve outcome and reduce demands within the health care system. Several studies reported a significant genetic background, with a certain risk for heritability, and a 4:1 male to female ratio.

**Materials and methods**

Examination of study participants shall be conducted according to the criteria of the Diagnostic_ and Statistical_ Manual of Mental Disorders, (DSM-IV-TR). Furthermore, defined in-and exclusion criteria shall apply. Whole exome sequencing shall be performed on the Illumina next sequencing platform at the King Fahad Medical Research Center, including methodology for sample preparation and quality control assessment, and production sequencing. Informatics upstream data analysis and interpretation shall be employed at the The Centre for Applied Genomics, The Hospital for Sick Children, Toronto, Canada.

**Results**

This project has started to recruit study participants according to the criteria outlined above. Literature review identified a number of case-control studies on biomarkers in ASD patients from Saudi Arabia which may support interpretation of our anticipated results. We propose that application of whole exome sequencing shall not only lead to the identification of possible pathogenic impairments in known ASD-related genes as listed in the Autism Database, (AutDB http://autism.mindspec.org/autdb/Welcome. do), but shall also result in identification of suspected ASD-related genes and ASD-related biological networks and pathways. Furthermore, we attempt to unravel novel genes not yet known to be related to ASD but may have a regional prevalence. This study aims also to contribute to leverage the involvement of the Canadian project partners in the international Autism Sequencing Consortium, which aims to decode approximately 10,000 autistic genomes. Furthermore, a major component of this project is to transfer expertise in exome sequencing from the Canadian to our Jeddah facility.

**Conclusions**

We assume that by detecting the genetic causes in a part of the study probands and by identifying affected biological networks and pathways, our study shall strengthen with its cutting-edge approach already existing ASD research in Saudi Arabia.

**Acknowledgements**

This study is supported by KAU grant 117-36-HiCi.

## P108 Crystal structure of the complex formed between Phospholipase A_2_ and the central core hydrophobic fragment of Alzheimer’s β- amyloid peptide: a reductionist approach

### Zeenat Mirza^1,2^, Vikram Gopalakrishna Pillai^2,3^, Sajjad Karim^4^, Sujata Sharma^2^, Punit Kaur^2^, Alagiri Srinivasan^2^, Tej P Singh^2^, Mohammed Al-Qahtani^4^

#### ^1^King Fahd Medical Research Center, King Abdulaziz University, P.O. Box 80216, Jeddah 21589, Saudi Arabia; ^2^Department of Biophysics, All India Institute of Medical Sciences, New Delhi 110029, India. ^3^Children’s Hospital of Philadelphia, Philadelphia, PA 19104, USA; ^4^Center of Excellence in Genomic Medicine Research, King Abdulaziz University, P.O. Box 80216, Jeddah 21589, Saudi Arabia

##### **Correspondence:** Zeenat Mirza (zmirza1@kau.edu.sa) – Department of Biophysics, All India Institute of Medical Sciences, New Delhi 110029, India

**Background**

Alzheimer’s disease (AD), is pathologically hallmarked by misfolding of amyloid-β peptide (Aβ); which favors conversion from a native, often soluble form, to a nonnative, often insoluble structure and subsequent aggregation [1, 2]. Myriad of different biophysical and structural techniques have been employed to elucidate the secondary structure, conformational dynamics, aggregation propensity and morphology of Aβ [3, 4].

**Materials and methods**

We adopted a reductionist strategy and co-crystallised central core fragment of Aβ_(16-21)_ with phospholipase A_2_ (PLA_2_) and report the complex structure at 1.2 Å resolution (PDB id: 3JQL) determined by X-ray crystallography. The X-ray intensity data were collected on EMBL beamline X-11 at DESY, Hamburg with λ = 0.98 Å, using MAR CCD detector. The data were processed using the programs DENZO and SCALEPACK [5]. The crystals belong to space group P4_1_ with unit cell parameters a = b = 42.0 Å, c = 64.1 Å containing four molecules in the unit cell. Good electron density for the peptide was observed at the active site of PLA_2._

**Results**

**Fig. 41 (abstract P108):**
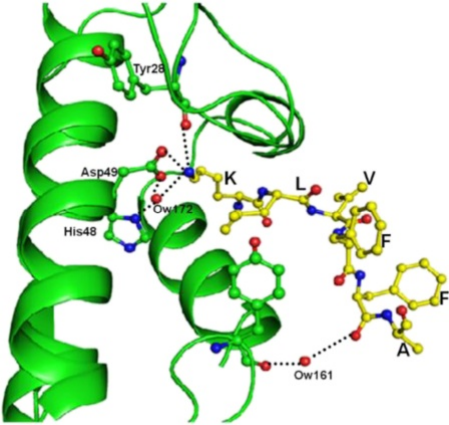
The critical interactions between PLA_2_ (green) and the peptide KLVFFA (yellow) shown by dotted line

All six residues of hexapeptide Lys-Leu-Val-Phe-Phe-Ala can be traced from their electron densities and positioned well in the substrate-binding hydrophobic channel of PLA_2_. Final R_cryst_ and R_free_ factors for the complete data in the resolution range of 20.0 - 1.1 Å were 0.18 and 0.192 respectively. Significant interactions are observed involving Nζ of terminal lysine of the peptide with Asp49 Oδ1 and Tyr28 O of the active site and also with active site water molecule OW172 which in turn interacts with active site residues (Fig. 41).

**Conclusions**

Our finding establishes possibility of interaction between Aβ and PLA_2_*in vivo* and sheds light on structure adopted by central hexapeptide. PLA_2_ inhibits the aggregation of Aβ by interacting with the peptide and keeping the two peptide chains apart. The selected peptide includes a pentapeptide sequence necessary for Aβ-Aβ binding and aggregation and can form fibrils on its own indistinguishable from those formed by full-length Aβ and probably forms the core of the fibril. This may potentially aid in future therapeutic interventions for AD.

**Acknowledgements**

Authors acknowledge the support from the Department of Biophysics, AIIMS, New Delhi, India.

**References**

1. Lansbury PT Jr.: **Evolution of amyloid: What normal protein folding may tell us about fibrillogenesis and disease.***Proc Nat Acad Sci USA* 1999, **96**:3342-3344.

2. Tomaselli S, Esposito V, Vangone P, van Nuland NA, Bonvin AM, Guerrini R, Tancredi T, Temussi PA, Picone D: **The alpha-to-beta conformational transition of Alzheimer’s Abeta-(1-42) peptide in aqueous media is reversible: a step by step conformational analysis suggests the location of beta conformation seeding.***Chembiochem* 2006, **7(2)**:257-67.

3. Coles M, Bicknell W, Watson AA, Fairlie DP, Craik DJ: **Solution structure of amyloid beta-peptide (1-40) in a water-micelle environment. Is the membrane-spanning domain where we think it is?***Biochemistry* 1998, **37**:11064-11077.

4. Das U, Hariprasad G, Ethayathulla AS, Manral P, Das TK, Pasha S, Mann A, Ganguli M, Verma AK, Bhat R, Chandrayan SK, Ahmed S, Sharma A, Kaur P, Singh TP, Srinivasan A: **Inhibition of Protein Aggregation: Supramolecular Assemblies of Arginine Hold the Key.***PLoS One* 2007, **2(11)**:e1176.

5. Otwinowski Z, Minor W: **Processing of X-ray diffraction data collected in oscillation mode.***Methods Enzymol* 1997, **276**:307-326.

## P109 Differential expression profiling between meningiomas from female and male patients

### Reem Alotibi^1^, Alaa Al-Ahmadi^1^, Fatima Al-Adwani^1,2^, Deema Hussein^3^, Sajjad Karim^1,4^, Mona Al-Sharif^2^, Awatif Jamal^5^, Fahad Al-Ghamdi^5^, Jaudah Al-Maghrabi^5,6^, Saleh S Baeesa^7^, Mohammed Bangash^7^, Adeel Chaudhary^1,4^, Hans-Juergen Schulten^1,4^, Mohammed Al-Qahtani^1,4^

#### ^1^Center of Excellence in Genomic Medicine Research, King Abdulaziz University, Jeddah, Saudi Arabia; ^2^Department of Biology, King Abdulaziz University, Jeddah, Saudi Arabia; ^3^King Fahad Medical Research Center, King Abdulaziz University, Jeddah, Saudi Arabia; ^4^KACST Technology Innovation Center in Personalized Medicine, King Abdulaziz University, Jeddah, Saudi Arabia; ^5^Department of Pathology, Faculty of Medicine, King Abdulaziz University Hospital, Jeddah, Saudi Arabia; ^6^Department of Pathology, King Faisal Specialist Hospital and Research Center, Jeddah, Saudi Arabia; ^7^Division of Neurosurgery, Department of Surgery, King Abdulaziz University Hospital, Jeddah, Saudi Arabia

##### **Correspondence:** Hans-Juergen Schulten (hschulten@kau.edu.sa) – KACST Technology Innovation Center in Personalized Medicine, King Abdulaziz University, Jeddah, Saudi Arabia

**Background**

Meningiomas arise from the meningothelial cap cells of the arachnoidal membrane and have a predominance of about 70 % in females. Hormones are regarded as a predisposition factor as a subset of meningiomas express hormone-receptors; however, the molecular mechanism underlying the female predominance are not thoroughly understood [1].

**Materials and methods**

RNA isolation and array sample processing for Affymetrix HuGene 1.0 ST array hybridization has been described earlier [2]. Whole transcript expression profiles were generated from 10 female and four from male patients. A set of differentially expressed genes (DEGs) was generated based on established criteria [2]. Bioinformatics software packages were utilized to interpret data sets.

**Results**

Microarray expression analysis identified nearly 100 genes that were differentially expressed between meningiomas from female and male patients. More than 10 % of the DEGs were transcribed from the Y chromosome. Among the autosomal located genes that were higher expressed in meningiomas from females were *rhotekin 2* (*RTKN2*), and *neuritin 1* (*NRN1*) and that were lower expressed were *fibroblast growth factor 10* (*FGF10*), *mucin 12, cell surface associated* (*MUC12*), Top upstream regulators include the inositol 1,4,5-triphosphate receptor (*ITPR*) and (E)-2,3′,4,5′-tetramethoxystilbene. A top associated network function was entitled Post-Translational Modification, Cellular Development, Cellular Growth and Proliferation.

**Conclusions**

This microarray expression analysis identified a number genes and biofunctions which may provide molecular clues for the predominance of female meningioma patients. Candidate genes include, besides others, the critical RhoA effector *RTKN2* and *NRN1* that is known to be associated with astrocytoma progression [3]. Further studies have to assess the functional involvement of the identified genes as drivers of meningioma initiation and/or progression in female patients.

**Acknowledgements**

This study was supported by King Abdulaziz City for Science and Technology (KACST) grant AT-32-98.

**References**

1. Claus EB, Park PJ, Carroll R, Chan J, Black PM: **Specific genes expressed in association with progesterone receptors in meningioma**. *Cancer research* 2008, **68**(1):314-322.

2. Schulten H-J, Alotibi R, Al-Ahmadi A, Ata M, Karim S, Huwait E, Gari M, Al-Ghamdi K, Al-Mashat F, Al-Hamour OA *et al*: **Effect of BRAF mutational status on expression profiles in conventional papillary thyroid carcinomas**. *BMC genomics* 2015, **16**(Suppl 1):S6-S6.

3. Zhang L, Zhao Y, Wang CG, Fei Z, Wang Y, Li L, Li L, Zhen HN: **Neuritin expression and its relation with proliferation, apoptosis, and angiogenesis in human astrocytoma**. *Medical oncology* 2011, **28**(3):907-912.

## P110 Neurospheres as models of early brain development and therapeutics

### Muhammad Faheem^1^, Peter Natesan Pushparaj^2^, Shilu Mathew^1^, Taha Abdullah Kumosani^1,3^, Gauthaman Kalamegam^2^, Mohammed Al-Qahtani^2^

#### ^1^Department of Biochemistry, Faculty of Science, King Abdulaziz University, Jeddah, Saudi Arabia; ^2^Centre of Excellence in Genomic Medicine Research (CEGMR), King Abdulaziz University, Jeddah Saudi Arabia; ^3^Experimental Biochemistry Unit, King Fahd Medical Research Centre King, Abdulaziz University, Jeddah, Saudi Arabia

##### **Correspondence:** Gauthaman Kalamegam (kgauthaman@kau.edu.sa) – Centre of Excellence in Genomic Medicine Research (CEGMR), King Abdulaziz University, Jeddah Saudi Arabia

**Background**

Neurospheres (NS) serves as a good *in vitro* model system to study neurodevelopmental alterations induced by neurotoxicants on fundamental processes of brain development [1]. The objective of this study was to investigate the proliferation, migration and differentiation patterns of neural stem cells within 3D neurospheres.

**Materials and methods**

**Fig. 42 (abstract P110):**
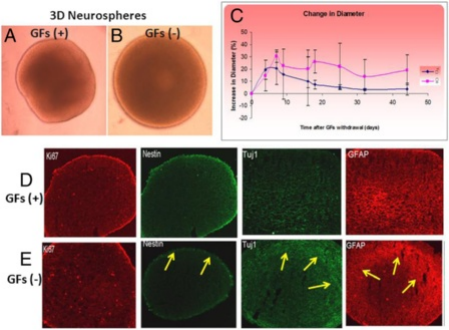
**a**, **b** - 3D neurospheres (3D-NS) with and without GFs; **c**- Change in 3D-NS diameter; **d**, **e** - Immunohistochemical images showing neuronal redistribution (arrows) in GFs(-) 3D-NS

Normal human neural progenitor (NHNP) cells were derived from male and female abortuses (16 weeks) following ethical committee approval and cultured using DMEM:F12 media supplemented with B27, EGF (20 ng/ml), FGF (20 ng/ml). Free floating 3D-NS were generated from HNHP cells and cultured in the presence or absence of growth factors (GFs) for up to 44 days (Fig. 42a, b). Effects of GFs withdrawal on 3D-NS was ascertained by studying the changes in (i) neurosphere diameter; (ii) proliferation following immunohistochemistry (IHC) staining with proliferation marker Ki67 using FACS; and (iii) cellular composition and localisation using IHC markers for nestin (neural stem cells, Tuj1 (neuronal cells) and GFAP (glial cells).

**Results**

Upon withdrawal of GFs the diameter of 3D-NS increased for up to 7 days from 0.86 μM to 0.98 μM (Fig. 42c). Thereafter, there was either shrinkage or increase by 12 % from its original size. In the presence of GFs, cell proliferation was mainly observed in the periphery of the sphere. IHC identified that nestin positive cells were restricted to the outer region of the 3D-NS, while Tuj1 and GFAP positive cells were localized in the inner region (Fig. 42d). However, following withdrawal of GFs, cellular redistribution was evident as demonstrated by migration of the neuronal (Tuj1 + ve) and glial cells (GFAP + Ve) to the outer region (Figure E). Nestin positive cells at the outer region of 3D-NS decreased and appeared as a thin outer circle (Fig. 42e).

**Conclusions**

3D-NS represent self-organized and dynamic structures in which the migration, proliferation and differentiation of stem cells can be analysed. 3D-NS model thus serve as an excellent *in vitro* biological tool to study the brain developmental stages, cellular redistribution, the effect of various known neurotxicants and screening of new pharmacological agents. This will greatly help to understand scientific inquiries and development of novel therapeutics.

**Acknowledgements**

We express our gratitue and appreciation for the financial support provided by King Abdulaziz City for Science and Technology (KACST) postgraduate student grant [AA-].

**References**

1. Fritsche E, Gassmann K, Schreiber T: **Neurospheres as a model for developmental neurotoxicity testing**. *Methods Mol Biol* 2011, **758**: 99-114.

## P111 Identification of a recurrent causative missense mutation p.(W577C) at the LDLR exon 12 in familial hypercholesterolemia affected Saudi families

### Faisal A Al-Allaf^1,2,3^, Zainularifeen Abduljaleel^1,2^, Abdullah Alashwal^4^, Mohiuddin M. Taher^1,2^, Abdellatif Bouazzaoui^1,2^, Halah Abalkhail^4^, Faisal A. Ba-Hammam^1^, Mohammad Athar^1,2^

#### ^1^Department of Medical Genetics, Umm Al-Qura University, Makkah, Saudi Arabia; ^2^Science and Technology Unit, Umm Al-Qura University, Makkah, Saudi Arabia; ^3^Department of Laboratory and Blood Bank, King Abdullah Medical City, Makkah, Saudi Arabia; ^4^King Faisal Specialist Hospital and Research Centre, Riyadh, Saudi Arabia

##### **Correspondence:** Mohammad Athar (mabedar@uqu.edu.sa) – Science and Technology Unit, Umm Al-Qura University, Makkah, Saudi Arabia

**Background**

Familial hypercholesterolemia (FH) is an autosomal dominant disease, predominantly caused by mutation in the low-density lipoprotein receptor (LDLR) gene. Herein, we describe genetic analysis of severely affected homozygous FH patients who were mostly resistant to statin therapy and were managed on an apheresis program.

**Results**

Screening for the LDLR mutations was performed by exon sequencing analysis. We identified a recurrent missense mutation c.1731G > T, p.(W577C) in exon 12 of the *Ldlr* gene in the probands and their relatives in an apparently unrelated Saudi families. All the probands were homozygous for the mutation, which is located in the EGF-precursor homology domain of the LDLR protein, and show severe FH phenotype. To the best of our knowledge this is the first report of a mutation in the *Ldlr* gene from the Arab population, including the Saudi population. We also describe a three dimensional homology model of LDLR structure and examine the consequence of the recurrent missense mutation p.(W577C), as this could affect the LDLR structure in a region involved in dimer formation, and protein stability.

**Conclusions**

This finding of a recurrent missense mutation causing FH in the Saudi population could serve to develop a rapid screening procedure for FH, and the 3D-structure analysis of the mutant LDLR, may provide a mechanistic model of the LDLR function.

## P112 Epithelial ovarian carcinoma (EOC): Systems oncological approach to identify diagnostic, prognostic and therapeutic biomarkers

### Gauthaman Kalamegam^1^, Peter Natesan Pushparaj^1^, Muhammad Abu-Elmagd^1,2^, Farid Ahmed^1^ Khalid HussainWali Sait^3^, Nisreen Anfinan^3^, Mamdooh Gari^1^, Adeel Chaudhary^1^, Adel Abuzenadah^1^, Mourad Assidi^1,2^, Mohammed Al-Qahtani^1^

#### ^1^Center of Excellence in Genomic Medicine Research (CEGMR), King Abdulaziz University, Jeddah, Saudi Arabia; ^2^Center of Innovation in Personalized Medicine, King Abdulaziz University, Jeddah, Saudi Arabia; ^3^Gynecological Oncology Unit, Department of Obstetrics and Gynaecology, Faculty of Medicine, King Abdulaziz University Hospital, Jeddah, Saudi Arabia

##### **Correspondence:** Mourad Assidi (mourad.assidi@gmail.com) – Center of Innovation in Personalized Medicine, King Abdulaziz University, Jeddah, Saudi Arabia

**Background**

Epithelial ovarian cancer is the leading gynaecological malignancy [1] which carries high mortality rate if diagnosed at late stage. As disease symptoms at early stage are obscure and the present screening methods and biomarkers lack sensitivity for early detection of EOC more cases are unfortunately diagnosed at late stage of disease. Therefore, it is very much necessary to identify new diagnostic/prognostic biomarkers to favour early detection. Towards this cause, we attempt to identify the top canonical pathways, molecular mechanisms, disease associations and toxicological functions in EOC using systems biology.

**Materials and methods**

Genes and molecules involved in EOC were analyzed using Ingenuity Pathway Analysis (IPA) (Ingenuity System, Qiagen, USA) to get the global overview of signaling mechanisms in cancer and their cross associations with other diseases and functions. Core analysis of the commonly implicated genes were then performed (Fisher’s Exact Test, P < 0.05).

**Results**

**Fig. 43 (abstract P112):**
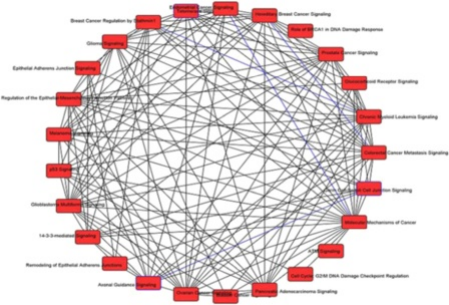
An Overlapping canonical genes in EOC

IPA analysis of molecules involved in EOC identified the top canonical pathways that were also associated with prostate cancer signalling (31.4 %), regulation of the Epithelial-Mesenchymal Transition (EMT) pathway (19.0 %), molecular mechanisms of cancer (13.1 %) and pancreatic adenocarcinoma signaling (25.0 %). There was 97.2 % (684/704) overlap of EOC molecules. The top up-stream regulators identified to be involved in EOC are beta-estradiol; EGF, TP53, TGFB1 and SP1. Molecules involved in cell survival/death, cell cycle and cell growth/proliferation were 345, 183 and 353 respectively. Overlapping canonical pathways in EOC as identified by IPA are depicted in Fig. 43. Of the 51 genes identified for their involvement in molecular mechanisms the most commonly involved genes and their biomarker applications are listed in Table 21.

**Conclusions**

Early detection of ovarian cancer is crucial for both treatment and prognosis. IPA enabled us to identify top canonical genes, top up-stream regulators and molecular mechanisms as well as the biomarker applications of most commonly involved genes. Important candidate biomarkers needs validation using biological screening of samples to ascertain their expression/inhibition patterns which could then be used to develop diagnostic and prognostic assays/kits.

**Acknowledgements**

We sincerely thank the Chair “Abdullah Basalamh of the women’s Tumors”, King Abdulaziz University for supporting this study.

**References**

1. Siegel R, Naishadham D, Jemal A: **Cancer statistics, 2012**. *CA Cancer J Clin* 2012, **62**:10–29.

**Table 21 (abstract P112):** Genes and their biomarker applications.

Gene Symbol	Biomarker Application(s)
ABL1	Efficacy, Response to therapy
APC	Diagnosis, disease progression, efficacy, prognosis, safety
BCL2	Diagnosis, efficacy, prognosis,
CDH1	
CDK4	Diagnosis, disease progression, efficacy, prognosis
CHEK1	Diagnosis
JAK2	Efficacy
KRAS	Diagnosis, efficacy, prognosis, response to therapy
PIK3CA	Efficacy, prognosis, response to therapy
TP53	Diagnosis, disease progression, efficacy, prognosis, response to therapy

## P113 Crohn’s disease phenotype in northern Tunisian population

### Naira Ben Mami^1^, Yosr Z Haffani^1^, Mouna Medhioub^2^, Lamine Hamzaoui^2^, Ameur Cherif^1^, Msadok Azouz^1,2^

#### ^1^Laboratory of Biotechnology and Valorization of Bio/Geo Resources LR11ES31 High Institute of Biotechnology Sidi Thabet, University of Manouba, Biotechpole Sidi Thabet, Tunisia; ^2^Department of Gastroenterology, Hospital Mohamed Taher Maamouri, Nabeul, Tunisia

##### **Correspondence:** Naira Ben Mami (neira22@yahoo.com) – Laboratory of Biotechnology and Valorization of Bio/Geo Resources LR11ES31 High Institute of Biotechnology Sidi Thabet, University of Manouba, Biotechpole Sidi Thabet, Tunisia

**Background**

Crohn’s disease (CD) is a chronic inflammatory disease affecting the digestive tract with extraintestinal manifestations and associated immune disorders. CD is generally classified into three entities depending on disease location: ileum, colon and ileocolon with or without complications (stricture, penetrating and perianally penetrating) [1]. The causes of inflammation are multiple implying a genetic susceptibility, a dysbiosis, autoimmune and environmental factors. To date there have been a genome-wide meta-analysis performed in CD patients leading to 71 susceptibility loci [2]. Our aim is to investigate the Crohn’s disease phenotype in North Tunisian population and to establish a correlation with genotypes for CD-associated polymorphisms (IL23R, JAK2 and SMAD3).

**Results**

The study included 91 patients with Crohn’s disease composed of 47 males and 44 females. Mean age is 41 years (41.12 ± 14.71). The diagnosis of CD was determined by standard clinical, radiological, endoscopic and histological criteria. CD is located in the terminal ileum in 35 %, colon in 19 % and ileocolon in 46 %. More than half of patients (52 %) had a non-structuring non-penetrating phenotype, 19 % a structuring, 16 % a penetrating and 13 % perianally penetrating phenotype.

**Conclusions**

We are in the process of acquiring additional biological samples (blood, serum and biopsy) of the control group and of the patients with Crohn’s disease and ulcerative colitis to investigate candidate genes polymorphisms and cytokine expression profiles. By our ongoing study we will be better able to treat CD by targeting specific cellular pathways at a molecular level.

**References**

1. Baumgart DC, Sandborn WJ: **Crohn’s disease**. *Lancet.* 2012 Nov, 380 (9853):1590-605.

2. Franke A and coll**:** Genome-wide meta-analysis increases to 71 the number of confirmed Crohn’s disease susceptibility loci. *Nat Genet*. 2010 Dec; **42(12)**:1118-2.

## P114 Establishment of *In Silico* approaches to decipher the potential toxicity and mechanism of action of drug candidates and environmental agents

### Gauthaman Kalamegam^1^, Fazal Khan^2^, Shilu Mathew^1^, Mohammed Imran Nasser^1^, Mahmood Rasool^1^, Farid Ahmed^1^, Peter Natesan Pushparaj^1,3^, Mohammed Al-Qahtani^1^

#### ^1^Center of Excellence in Genomic Medicine Research, King Abdulaziz University, Jeddah, Saudi Arabia; ^2^Center of Innovation in Personalized Medicine, King Fahd Medical Research Center, King Abdulaziz University, Jeddah, Saudi Arabia; ^3^Biochemistry Department, Faculty of Science; Production of Bioproducts for Industrial Applications Research Group; and Experimental Biochemistry Unit, King Fahd Medical Research Center King, Abdulaziz University, Jeddah, Saudi Arabia

##### **Correspondence:** Peter Natesan Pushparaj (peter.n.pushparaj@gmail.com) – Center of Excellence in Genomic Medicine Research, King Abdulaziz University, Jeddah, Saudi Arabia

**Background**

Understanding the molecular mechanisms of action of drug candidates is pivotal in drug discovery and development [1, 2]. More importantly, knowing the toxicological functions can help in evaluating the risk of genotoxicity and carcinogenesis [1-3]. Besides, it can help in the rational screening for novel candidate compounds targeting canonical pathways and novel gene networks. In the present study, we investigate, 20 different drug candidates and environmental agents, for both efficacy and toxicity using cutting-edge *in silico* approaches.

**Materials and methods**

In order to study the mechanisms of action of 20 different compounds important in both experimental therapeutics and molecular toxicology, we have used Ingenuity Pathway Analysis (IPA) knowledgebase (Ingenuity Systems, Qiagen, USA) to obtain their molecular targets in mammalian cells and tissues. The list of target molecules for each compound was further clarified using Fisher’s Exact Test and Benjamini Hochberg Multiple Testing Correction (P < 0.05) and subjected to core analysis using IPA to decipher top canonical pathways, novel molecular networks, biological and toxicological functions regulated by these agents. Furthermore, we have used the multiple comparison module in IPA to compare all the core analyses results to generate hierarchical clusters (2-fold cut-off) for top canonical pathways, diseases and biological and toxicological functions.

**Results**

**Fig. 44 (abstract P114):**
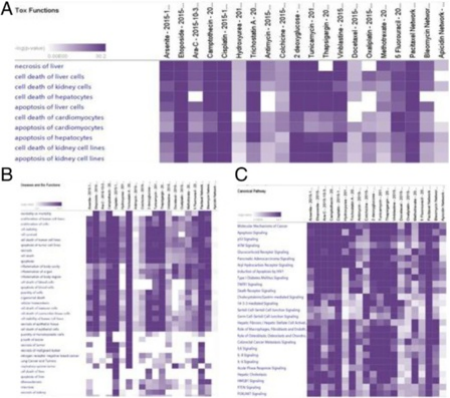
*In Silico* analyses of 20 drug candidates and environmental agents in mammalian cells and tissues using molecular interaction data obtained and clarified from Ingenuity Knowledgebase. Hierarchical clustering shows the statistically significant Tox Functions (A), Disease and Bio Functions (B) and Canonical Pathways (C) regulated by genotoxic agents in mammals

We have identified unique toxicological effects, such as hepatotoxicity, cardiotoxicity and nephrotoxicity, for Arsenite, Etoposide, Ara-C, Camptothecin, and Cisplatin (Fig. 44a). Furthermore, these compounds potently regulate cell death and apoptosis of tumor cell lines (Fig. 44b). However, most of the compounds significantly regulate the Molecular Mechanisms of Cancer, P53 Signaling, Apoptosis Signaling, Aryl Hydrocarbon Receptor Signaling in mammalian systems (Fig. 44c).

**Conclusions**

Our *in silico* study has deciphered an array of canonical pathways and novel gene networks regulated by anti-cancer drugs and environmental agents. Establishing the *in silico*- based characterization of known as well as novel compounds may provide novel cues for further investigations using *in vitro* and *in vivo* systems and helps in the effective design and development of drugs for the management of cancer and other debilitating diseases.

**Acknowledgements**

This work was funded by the National Plan for Science, Technology and Innovation (MAARIFAH) – King Abdulaziz City for Science and Technology - the Kingdom of Saudi Arabia – award number (12-BIO2267-03). The authors also, acknowledge with thanks Science and Technology Unit, King Abdulaziz University for their excellent technical support.

**References**

1. Snyder RD, Green JW: **A review of the genotoxicity of marketed pharmaceuticals**. *Mutat Res* 2001, **488**:151-69.

2. Hanahan D, Weinberg RA: **The hallmarks of cancer.***Cell* 2000, **100**:57-70.

3. Hoeijmakers JH: **Genome maintenance mechanisms for preventing cancer**. *Nature* 2001, **411**:366-74.

## P115 1q Gain predicts poor prognosis marker for young breast cancer patients

### Shereen A Turkistany^1^, Lina M Al-harbi^2^, Ashraf Dallol^1,2^, Jamal Sabir^3^, Adeel Chaudhary^1,2,4^, Adel Abuzenadah^1,2,4^

#### ^1^Center of Innovation in Personalized Medicine, King Abdulaziz University, Jeddah, Saudi Arabia; ^2^Center of Excellence in Genomic medicine Research, King Abdulaziz University, Jeddah, Saudi Arabia; ^3^Biology Department, Faculty of Science, King Abdulaziz University, Jeddah, Saudi Arabia; ^4^Medical Laboratory Department, Faculty of Applied Medical Technology, King Abdulaziz University, Jeddah, Saudi Arabia

##### **Correspondence:** Shereen A Turkistany (shereenturkistany@gmail.com) – Center of Innovation in Personalized Medicine, King Abdulaziz University, Jeddah, Saudi Arabia

**Background**

Breast cancer (BC) is the second most common cancer worldwide. Incidence rates in BC are increasing steadily in Arab countries with different age structure from US and Europe. Epidemiological studies showed 50 % of BC patients in Arab countries are below the age of 50 years compared to only 25 % in US. Young age BC patients exhibit high mortality rate with early disease relapse. There is an urge need to specify a new prognostic factor in BC young patients that can help in the selection of appropriate therapy and the assessment of patients’ risk. In this study as a starting point, we used Comparative Genomic hybridization (CGH) arrays to evaluate copy number changes in twenty breast cancer patient samples. Many cytogenetic alterations were detected in our study correlated with published studies. The most frequent aberration was amplification of chromosome1q, which was confirmed by FISH technique. Second, we used 3D digital PCR to validate amplification of 1q using two different probes in 75 BC patient samples. Finally, we assessed the correlation between the chromosomal amplification of 1q and the clinical feature of the disease.

**Results**

8 of twenty BC patients examined with CGH arrays had amplified 1q, and confirmed with FISH technique. In concordance with CGH arrays results, 32 % of seventy-five BC patients examined with 3D digital PCR showed 1q amplification. BC patient’s samples with 1q amplification had correlation with large tumor size (p=. 013). Also, Univariate Kaplan–Meier survival test revealed that there is a significant association of 1q amplification and less disease free survival duration in young BC patients (p = .028).

**Conclusions**

This study presents 1q amplification as the most frequent aberration in BC patient’s samples. In addition, the study features 1q amplification association with young BC patients (<50) with poor prognosis as indicated by less disease free survival time and large tumor size. In conclusion, 1q amplification could serve a prognosis marker for young BC patients’ sample.

## P116 Disorders of sex chromosomes in a diagnostic genomic medicine unit in Saudi Arabia: Prevalence, diagnosis and future guidelines

### Basmah Al-Madoudi^1^, Bayan Al-Aslani^1^, Khulud Al-Harbi^1^, Rwan Al-Jahdali^1^, Hanadi Qudaih^1^, Emad Al Hamzy^1^, Mourad Assidi^1,2^, Mohammed Al Qahtani^1^

#### ^1^Diagnostic Genomic Medicine Unit, Center of Excellence in Genomic Medicine Research, King Abdulaziz University, Jeddah, Saudi Arabia; ^2^Center of Innovation in Personalized Medicine, King Abdulaziz University, Jeddah, Saudi Arabia

##### **Correspondence:** Mourad Assidi (mourad.assidi@gmail.com) – Center of Innovation in Personalized Medicine, King Abdulaziz University, Jeddah, Saudi Arabia

**Background**

Disorders of Sex Chromosomes (DSCs) includes all cases of X and/or Y structural abnormalities or mosaicism which are associated with ambiguity in sex determination [1]. They include mainly the sex chromosome aneuploidies (SCAs) which are common disorders affecting 1 out of 400 newborns [2]. This high incidence among population is associated to a low abortion rate (compared to autosomal aneuploidies) [3] and encompass a range of chromosomal abnormalities mainly Klinefelter’s syndrome, Turner’s syndrome, XYY or Jacob’s syndrome and Triple X or Superwoman syndrome with an incidence range at birth from 1/500 to 1/2000 [2, 3]. These DSCs have direct impact on biological sex determination, embryonic development and require some medical, social and educational care [4]. In Saudi Arabia, a systematic screening for DSCs is not implemented yet and only referred cases receive this diagnostic service. The objective is to assess the prevalence of most common DSCs in a Genomic Diagnostic Unit in KSA, determine the characteristics of the target population, and provide recommendations toward more comprehensive and multidisciplinary care for patients with DSCs. This study was performed using blood samples from 2256 consent patients referred to DGMU at CEGMR for subsequent cytogenetic or molecular analyses.

**Results**

**Fig. 45 (abstract P116):**
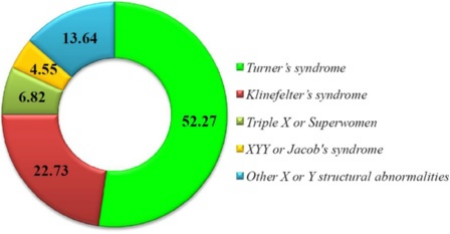
Proportion of the main DSCs in our SCAs cohort (%)

Cytogenetic data analysis revealed an incidence of chromosomal abnormalities of 18.35 % among all cases referred to DGMU. Only 10.62 % of these affected subjects have DSCs. Half of these sex chromosome abnormalities were numerical, while the structural disorders and mosaicism were 18.18 % and 31.8 %, respectively. Turner’s and Klinefelter’s syndromes are the most common DSCs in our patient cohort (Fig. 45). Surprisingly, only 25 % of the DSCs were infants (Table 22) indicating a serious delay of diagnosis.

**Conclusions**

We reported a high incidence of DSCs (10 %) in Saudi population with a late first diagnosis for these serious abnormalities mainly at adolescence and adulthood suggesting a lack of awareness in the Kingdom that need to be prioritized. This delay might have serious personal, educational, societal and psychological impacts on affected subjects. Therefore, implementation of a routine clinical service for newborn aneuploidy screening and a prevention program included in the premarital testing is highly recommended.

**References**

1. Sharma R, Agarwal A, Rohra VK, Assidi M, Abu-Elmagd M, Turki RF: **Effects of increased paternal age on sperm quality, reproductive outcome and associated epigenetic risks to offspring**. *Reproductive biology and endocrinology : RB&E* 2015, **13**:35.

2. Nabavi SM, Daglia M, Braidy N, Nabavi SF: **Natural products, micronutrients, and nutraceuticals for the treatment of depression: A short review**. *Nutritional neuroscience* 2015.

3. Gaziano JM, Concato J, Brophy M, Fiore L, Pyarajan S, Breeling J, Whitbourne S, Deen J, Shannon C, Humphries D *et al*: **Million Veteran Program: A mega-biobank to study genetic influences on health and disease**. *Journal of clinical epidemiology* 2015.

4. Dallol A, Buhmeida A, Merdad A, Al-Maghrabi J, Gari MA, Abu-Elmagd MM, Elaimi A, Assidi M, Chaudhary AG, Abuzenadah AM *et al*: **Frequent methylation of the KLOTHO gene and overexpression of the FGFR4 receptor in invasive ductal carcinoma of the breast**. *Tumour biology : the journal of the International Society for Oncodevelopmental Biology and Medicine* 2015, **36**(12):9677-9683.

**Table 22 (abstract P116):** Distribution of DSCs patient according to their age

Age category	Proportion (%)
Adult (≥15years)	47.73
Children (2-14 years)	27.27
Infant (less than 2 years)	25.00

## P117 Combination of WYE354 and Sunitinib demonstrate synergistic inhibition of acute myeloid leukemia *in vitro*

### Asad M Ilyas^1^, Youssri Ahmed^1,2,3^, Mamdooh Gari^4^, Farid Ahmed^4^, Mohammed Alqahtani^4^

#### ^1^Department of Biochemistry, King Abdulaziz University, Saudi Arabia; ^2^Production of Bioproducts for Industrial Applications Research Group and Experimental Biochemistry Unit, King Fahd Medical Research Center, King Abdulaziz University, Saudi Arabia; ^3^Microbial Biotechnology Department, National Research Center, Dokki, Cairo, Egypt; ^4^Centre for Excellence in Genomic Medicine Research, King Abdulaziz University, Saudi Arabia

##### **Correspondence:** Farid Ahmed (fahmed1@kau.edu.sa) – Centre for Excellence in Genomic Medicine Research, King Abdulaziz University, Saudi Arabia

**Background**

Acute Myeloid Leukemia (AML) is a clonal heterogeneous disease of myeloid progenitors which results in the accumulation of immature blast cells in the peripheral blood and bone marrow. Although there has been significant improvements in the survival of AML cases over the past few decades, a majority of the older AML cases and those with relapsed or secondary AML, continue to show poor outcome. The main causes attributed to failure of conventional chemotherapy in AML are treatment related mortalities and drug resistance [1]. Recent clinical trials in targeted therapy of AML has shown development of resistance against single agents. Combining single agents with existing therapies or novel combinations of new agents has therefore become imperative. In this study we evaluated the effect of co-inhibition of mTOR and receptor tyrosine kinases (RTK) in AML cell lines.

**Materials and methods**

Cell viability was assessed using Cell-Titre Blue kit (Promega) as per manufacturer’s protocol, after 48 hours of incubation with drug/s. Combination index (CI) values were calculated for synergism by CompuSyn software based on dose response curve analysis [2]. To determine the total apoptotic cells, cells were stained with Annexin V (BD bioscience) and analyzed on flow cytometer (BD FACS Aria III).

**Results**

Inhibitor of mTOR WYE-354, and pan RTK inhibitor sunitinib synergistically inhibit acute myeloid leukemia cell lines K562 and HL60. CI values for K452 are; 0.792 (ED50), 0.661 (ED75) and 0.557 (ED90). According to median effect principle CI < 1 represents synergism, CI = 1 represent additive, whereas CI > 1 represent antagonsim. Combination of these inhibitors significantly induce apoptosis (p = 0.0009 for ED75 and <0.0001 for ED90) in comparison with individual inhibitor in leukemic cell lines.

**Conclusions**

In this study we report that combination of RTK inhibitors with mTOR inhibitors show synergistic inhibition of AML cell lines and therefore offers a promising approach to deal with AML.

**Acknowledgements**

This work was supported by generous funds from the National Plan for Science, Technology and Innovation (MAARIFAH), King Abdulaziz City for Science and Technology, Kingdom of Saudi Arabia – award number (09-BIO-693-03).

**References**

1. Burnett AK: **Treatment of acute myeloid leukemia: are we making progress?***Hematology / the Education Program of the American Society of Hematology American Society of Hematology Education Program* 2012, **2012**:1-6.

2. Chou TC: **Comparison of dose-effect relationships of carcinogens following low-dose chronic exposure and high-dose single injection: an analysis by the median-effect principle**. *Carcinogenesis* 1980, **1**(3):203-213.

**Fig. 46 (abstract P117):**
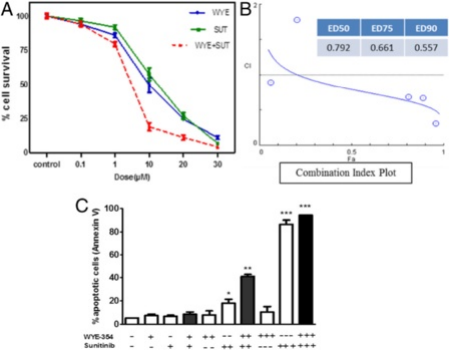
**a** Cytotoxicity of WYE354 (WYE) and Sunitinib (SUT) in K562 and the combination of these inhibitors at 48hrs. **b** Combination index plot showing CI values at different doses of WYE and SUT when combined. **c** Annexin V staining showing total % apoptotic cells using three different doses of WYE and SUT. Results represent mean of three independent experiments and unpaired t test is performed to determine the statistical significance between treated groups vs control

## P118 Integrated use of evolutionary information in GWAS reveals important SNPs in Asthma

### Nada Salem^1^, Sajjad Karim^1^, Elham M Alhathli^1^, Heba Abusamra^1^, Hend F Nour Eldin^1^, Mohammed H Al-Qahtani^1^, Sudhir Kumar^2^

#### ^1^Center of Excellence in Genomic Medicine, King Abdulaziz University, PO Box 80216, Jeddah 21589, Saudi Arabia; ^2^Institute for Genomics and Evolutionary Medicine, Temple University (SERC 602A), Philadelphia, Pennsylvania 19122 USA

##### **Correspondence:** Sajjad Karim (skarim1@kau.edu.sa) – Center of Excellence in Genomic Medicine, King Abdulaziz University, PO Box 80216, Jeddah 21589, Saudi Arabia

**Background**

Genome wide association studies (GWAS) have become standanrd tool to identify disease-associated loci. GWAS use frequencies of single nucleotide variants (SNVs), to prioritize variants with the highest P-values that discriminate between cases and controls. However, not all SNVs occur at functionally equivalent positions, and it is important to consider the strength of functional importance of positions when prioritizing variants. We conducted a reanalysis of GWAS data by using the E-rank approach [1] that integrates evolutionary dimension into P-value in order to examine if additional bona-fide variant can be discovered.

**Materials and methods**

We used data from an asthma GWAS of ~550,000 SNVs (PMID: 20860503) [2]. For these variants, E-ranks and P-ranks were obtained from myPEG server. For the top 5000 E-rank variants, we obtained their replication which was reported to be significant in 45 sthma-related studies. This was done by querying the Genome-Wide Repository of Associations between SNPs and Phenotypes (GRASP v2.0) [3]. Written codes in “R” were used to compare E-rank and P-rank with replication and to estimate the genetic variance (GV) explained for each SNV according to this formula: GVi = 2 × log(ORi)^2^ x MAFi (1-MAFi) [4].

**Results**

**Fig. 47 (abstarct P118):**
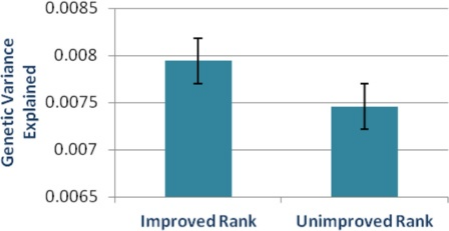
Genetic variance between E-rank - improved and unimproved SNVs groups. (Wilcoxon test P < 10^-16^; error bars = standard error of mean)

Of the top 5000 E-rank variants, 2926 were shared with the top 5000 P-rank variants. These shared variants were replicated in many studies, on average. Of the remaining, the average replication was 1.13 for E-ranks and 1.02 for P-ranks. We estimated the total heritability explained by increasing number of SNVs under an additive model and found that E-rank SNVs explain greater heritability than P-rank alleles (Wilcoxon test P < 10^-16^ ) (Fig. 47). Also, comparing E-rank and P-rank for the top replicated SNVs in multiple Asthma GWAS resulted in finding functionally missense SNVs that have improved average values of E-rank than P-rank (Table 23).

**Conclusions**

Our results show the usefulness of evolutionary ranking method of SNPs as an aid to P-values in assessing and identifying the most replicated SNPs that are functionally important. Since E-ranking approach requires the use of original full GWAS data and the MAF values, further efforts are needed to make them publically available.

***Acknowledgements***

*We would like to acknowledge the Deanship of Scientific Research, King Abdulaziz University, Jeddah, Saudi Arabia for funding the research (HiCi-1434-117-2).*

**References**

1. Dudley JT, Chen R, Sanderford M, Butte AJ, Kumar S: **Evolutionary meta-analysis of association studies reveals ancient constraints affecting disease marker discovery.***Mol Biol Evol* 2012, **29:**2087-2094.

2. Moffatt MF, Gut IG, Demenais F, Strachan DP, Bouzigon E, Heath S, von Mutius E, Farrall M, Lathrop M, Cookson WOCM: **A Large-Scale, Consortium-Based Genomewide Association Study of Asthma.***New England Journal of Medicine* 2010, **363:**1211-1221.

3. Eicher JD, Landowski C, Stackhouse B, Sloan A, Chen W, Jensen N, Lien J-P, Leslie R, Johnson AD: **GRASP v2.0: an update on the Genome-Wide Repository of Associations between SNPs and phenotypes.***Nucleic Acids Research* 2014.

4. Park J-H, Wacholder S, Gail MH, Peters U, Jacobs KB, Chanock SJ, Chatterjee N: **Estimation of effect size distribution from genome-wide association studies and implications for future discoveries.***Nat Genet* 2010, **42:**570-575.

**Table 23 (abstract P118):** Functionally important SNVs with improved E-rank among multiple Asthma GWAS

rsID	Average E-rank	Average P-rank	Times of replication	SNV class – Gene name
rs3894194	28.79	22.79	14	Missense - GSDMA
rs20541	40.08	34.15	13	Missense - IL13
rs11557467	28.64	20	11	Missense - ZPBP2
rs1877031	50.5	35.5	8	Missense - STARD3
rs10192157	27	21	6	Missense - IL1RL1

*GSDMA* gasdermin A, *IL13* interleukin 13, *ZPBP2* zona pellucida binding protein 2, *STARD3* StAR-related lipid transfer domain containing 3, *IL1RL1* interleukin1 receptor-like1

## P119 Assessment of *BRAF*, *IDH1*, *IDH2*, and *EGFR* mutations in a series of primary brain tumors

### Fatima Al-Adwani^1,2^, Deema Hussein^3^, Mona Al-Sharif^2^, Awatif Jamal^4^, Fahad Al-Ghamdi^4^, Jaudah Al-Maghrabi^4,5^, Saleh S Baeesa^6^, Mohammed Bangash^6^, Adeel Chaudhary^1,7^, Mohammed Al-Qahtani^1,7^, Hans-Juergen Schulten^1,7^

#### ^1^Center of Excellence in Genomic Medicine Research, King Abdulaziz University, Jeddah, Saudi Arabia; ^2^Department of Biology, King Abdulaziz University, Jeddah, Saudi Arabia; ^3^King Fahad Medical Research Center, King Abdulaziz University, Jeddah, Saudi Arabia; ^4^Department of Pathology, Faculty of Medicine, King Abdulaziz University Hospital, Jeddah, Saudi Arabia; ^5^Department of Pathology, King Faisal Specialist Hospital and Research Center, Jeddah, Saudi Arabia; ^6^Division of Neurosurgery, Department of Surgery, King Abdulaziz University Hospital, Jeddah, Saudi Arabia; ^7^KACST Technology Innovation Center in Personalized Medicine, King Abdulaziz University, Jeddah, Saudi Arabia

##### **Correspondence:** Hans-Juergen Schulten (hschulten@kau.edu.sa) – Center of Excellence in Genomic Medicine Research, King Abdulaziz University, Jeddah, Saudi Arabia

**Background**

Frequency and/or type of mutations in genes encoding *isocitrate dehydrogenase 1* (*IDH1*), *isocitrate dehydrogenase 2* (*IDH2*), and *epidermal growth factor receptor* (*EGFR*) vary between histological types of primary brain tumors. *BRAF* mutations in brain tumors have also been assessed in recent years [1]. We investigated the frequency of mutations in these genes in a series of primary brain tumors from the western Saudi Arabian region.

**Materials and methods**

The series of primary brain tumors consisted in the majority of astrocytomas, oligodendrogliomas, glioblastoma multiformes (GBMs), and a variety of minor histological types. Genomic DNA extraction, PCR conditions and cycle sequence reactions basically followed our standard protocols [2]. Gene specific PCR primers were used to flank mutational hotspots regions in *IDH1* exon 4, *IDH2* exon 4, *EGFR* exons 3 and 7, and *BRAF* exon 15.

**Results**

Sequence analysis identified *IDH1* R132H mutations in six of 40 (20 %) assessed brain tumors, namely in two oligodendrogliomas grade II, one oligodendroglioma grade III, two astrocytomas grade II, and one GBM. One *IDH2* R172 mutation (R172K) was identified in an oligodendroglioma grade II. No *BRAF* exon 15 and *EGFR* exons 3 and 7 mutations were identified in 54 and 31 assessed primary brain tumors, respectively.

**Conclusions**

In our series, *IDH1* mutations were identified in a considerable number of the major glioma types which is consistent with other mutational studies. Mutations in other assessed genes were rare or absent which may be attributed to the fact that *IDH2* and *BRAF* mutations are less common or more common in not assessed histological types, and only a minority of known *EGFR* aberrations were analyzed. Taken together, identifying critical gene mutations in brain tumors is gaining clinical relevance as, for example *IDH1* and *IDH2* mutations bear predictive and prognostic preposition.

**Acknowledgements**

This study was supported by King Abdulaziz City for Science and Technology (KACST) grant AT-32-98.

**References**

1. Berghoff AS, Preusser M: **BRAF alterations in brain tumours: molecular pathology and therapeutic opportunities**. *Current opinion in neurology* 2014.

2. Schulten HJ, Al-Maghrabi J, Al-Ghamdi K, Salama S, Al-Muhayawi S, Chaudhary A, Hamour O, Abuzenadah A, Gari M, Al-Qahtani M: **Mutational screening of RET, HRAS, KRAS, NRAS, BRAF, AKT1, and CTNNB1 in medullary thyroid carcinoma**. *Anticancer Res* 2011, **31**[1]:4179-4183.

## P120 Expression profiles distinguish oligodendrogliomas from glioblastoma multiformes with or without oligodendroglioma component

### Alaa Alamandi^1^, Reem Alotibi^1^, Deema Hussein^2^, Sajjad Karim^1,3^, Jaudah Al-Maghrabi^4,5^, Fahad Al-Ghamdi^4^, Awatif Jamal^4^, Saleh S Baeesa^6^, Mohammed Bangash^6^, Adeel Chaudhary^1,3^, Hans-Juergen Schulten^1,3^, Mohammed Al-Qahtani^1,3^

#### ^1^Center of Excellence in Genomic Medicine Research, King Abdulaziz University, Jeddah, Saudi Arabia; ^2^King Fahad Medical Research Center, King Abdulaziz University, Jeddah, Saudi Arabia; ^3^KACST Technology Innovation Center in Personalized Medicine, King Abdulaziz University, Jeddah, Saudi Arabia; ^4^Department of Pathology, Faculty of Medicine, King Abdulaziz University Hospital, Jeddah, Saudi Arabia; ^5^Department of Pathology, King Faisal Specialist Hospital and Research Center, Jeddah, Saudi Arabia; ^6^Division of Neurosurgery, Department of Surgery, King Abdulaziz University Hospital, Jeddah, Saudi Arabia

##### **Correspondence:** Hans-Juergen Schulten (hschulten@kau.edu.sa) – Center of Excellence in Genomic Medicine Research, King Abdulaziz University, Jeddah, Saudi Arabia

**Background**

Oligodendrogliomas (ODGs) and glioblastoma multiformes (GBMs) are common gliomas that in case of GBMs are afflicted with unfavorable prognosis. Microarray expression profiling in gliomas may become an adjunct clinical assessement tool for these heterogenous tumors.

**Materials and methods**

Whole transcript HuGene 1.0 ST arrays (Affymetrix Incorp) were employed [1], to generate expression profiles from five ODGs and four GBM. Three of the GBMs had an ODG component whereas one had no ODG component. A set of differentially expressed genes (DEGs) was generated based on a false discovery rate (FDR) p-value ≤ 0.05 and a fold change > 2. Software packages (Partek Genomics Suite version 6.6, Partek Inc., MO; Ingenuity Pathway Analysis, Ingenuity Systems, Redwood City, CA) were utilized to interpret data sets.

**Results**

Principal component analysis indicated that GBMs were more heterogenous in their expression profiles than ODGs. In particular, the number of DEGs between the GBM without ODG component and the five ODGs exceed 1000 genes whereas the respective numbers for each of the GBM with ODB component exceeded not 100 genes. Comparable upregulated genes in GBMs included matrix metallopeptidase 13 (collagenase 3)s (*MMP13*), and neurotensin (*NTS*). Comparable downregulated genes included reticulon 3 pseudogene 1 (*RTN3P1*) and G protein-coupled receptor 37 like 1 (*GPR37L1*). *TGFB1* was noticed as a top upstream regulator.

**Conclusions**

As ODGs follow a favorable clinical course, differential expression profiling between ODG and GBM may add on sub-classifying GBM, according to their ODG component, into different assessment groups. The identified DEGs contain biomarkers as *MMP13* that is known to be expressed in cancer stem-like cells of glioblastoma where it correlates with cell invasiveness [2]. In summary, this study suggests that microarray expression analysis is capable to distinguish individual cases of GBM based on the ODG component which may gain relevance for clinical assessment of gliomas.

**Acknowledgements**

This study was supported by King Abdulaziz City for Science and Technology (KACST) grant AT-32-98

**References**

1. Schulten HJ, Al-Mansouri Z, Baghallab I, Bagatian N, Subhi O, Karim S, Al-Aradati H, Al-Mutawa A, Johary A, Meccawy AA *et al*: **Comparison of microarray expression profiles between follicular variant of papillary thyroid carcinomas and follicular adenomas of the thyroid**. *BMC genomics* 2015, **16 Suppl 1**:S7.

2. Inoue A, Takahashi H, Harada H, Kohno S, Ohue S, Kobayashi K, Yano H, Tanaka J, Ohnishi T: **Cancer stem-like cells of glioblastoma characteristically express MMP-13 and display highly invasive activity**. *International journal of oncology* 2010, **37**(5):1121-1131.

## P121 Hierarchical clustering in thyroid goiters and hyperplastic lesions

### Ohoud Subhi^1^, Nadia Bagatian^1^, Sajjad Karim^1,2^, Adel Al-Johari^3^, Osman Abdel Al-Hamour^4^, Hosam Al-Aradati^5^, Abdulmonem Al-Mutawa^5^, Faisal Al-Mashat^3^, Jaudah Al-Maghrabi^5,6^, Hans-Juergen Schulten^1,2^, Mohammad Al-Qahtani^1,2^

#### ^1^Center of Excellence in Genomic Medicine Research, King Abdulaziz University, Jeddah, Saudi Arabia; ^2^KACST Technology Innovation Center in Personalized Medicine, King Abdulaziz University, Jeddah, Saudi Arabia; ^3^Department of Surgery, Faculty of Medicine, King Abdulaziz University, Jeddah, Saudi Arabia; ^4^Department of Surgery, King Faisal Specialist Hospital and Research Center, Jeddah, Saudi Arabia; ^5^Department of Pathology, King Faisal Specialist Hospital and Research Center, Jeddah, Saudi Arabia; ^6^Department of Pathology, Faculty of Medicine, King Abdulaziz University, Jeddah, Saudi Arabia

##### **Correspondence:** Hans-Juergen Schulten (hschulten@kau.edu.sa) – Center of Excellence in Genomic Medicine Research, King Abdulaziz University, Jeddah, Saudi Arabia

**Background**

Common benign lesions of the thyroid are goiters and hyperplastic lesions that; however, may bear a certain risk for neoplastic transformation. Hyperplastic lesions are also regarded as a subcategory of goiter. In a previous study we investigated the expression profiles in these diseases and found that they share similar expression profiles [1]. In the current study we regarded goiters and hyperplastic lesions as one entity aiming to identify subgroups that cluster separately based on differentially expressed profiles.

**Material and methods**

The study group comprised goiters and hyperplastic lesions from more than 30 patients and a number of PTC and normal/unaffected thyroid samples as reference. Sample processing and hybridization to HuGene 1.0 ST arrays was performed as reported earlier [2]. Bioinformatics software packages were employed to interpret data sets.

**Results**

Based on distance metrics, hierarchical cluster analysis stratified goiters and hyperplastic lesions into a number of subgroups. Two clearly separated subgroups (A and B) were selected to investigate their expression profiles in more detail. Among the most significantly upregulated genes in group A compared to group B were RNA, 5S ribosomal pseudogene 456 (*RNA5SP456*).and among the most downregulated genes were bromodomain and WD repeat domain containing 3 (*BRWD3*) and polypyrimidine tract binding protein 2 (*PTBP2*). Furthermore, the regulatory factor for X-box (*RFX5*) was significantly associated with the data set.

**Conclusions**

Inclusion of both malignant PTCs and normal/unaffected thyroid samples in hierarchical cluster analysis supported to identify differential expressed subgroups in goiters and hyperplastic lesions. *BRWD3* is a known serological biomarker in breast cancer patients [3]. *PTBP2* is a critical splicing factors in regulation of alternative splicing of pre-mRNA and is implicated in proliferation and migration of cancer cells [4]. Further studies are necessary to assess in how far the identified gene sets bear the capacity for neoplastic transformation.

**Acknowledgements**

This study was performed in support of the King Abdulaziz City for Science and Technology (KACST) grant 09-BIO2289-03.

**References**

1. Subhi O, Baqtian N, Ata M, Karim S, Al-Ghamdi K, Al-Hamour OA, Al-Qahtani MH, Schulten HJ, Al-Maghrabi J: **Nodular goiter and hyperplastic lesion of the thyroid share common deregulated expression profiles**. *BMC genomics* 2014, **15**(Suppl 2):P70.

2. Schulten HJ, Al-Mansouri Z, Baghallab I, Bagatian N, Subhi O, Karim S, Al-Aradati H, Al-Mutawa A, Johary A, Meccawy AA *et al*: **Comparison of microarray expression profiles between follicular variant of papillary thyroid carcinomas and follicular adenomas of the thyroid**. *BMC genomics* 2015, **16 Suppl 1**:S7.

3. Suh EJ, Kabir MH, Kang UB, Lee JW, Yu J, Noh DY, Lee C: **Comparative profiling of plasma proteome from breast cancer patients reveals thrombospondin-1 and BRWD3 as serological biomarkers**. *Experimental & molecular medicine* 2012, **44**(1):36-44.

4. Cheung HC, Hai T, Zhu W, Baggerly KA, Tsavachidis S, Krahe R, Cote GJ: **Splicing factors PTBP1 and PTBP2 promote proliferation and migration of glioma cell lines**. *Brain : a journal of neurology* 2009, **132**(Pt 8):2277-2288.

## P122 Differential expression analysis in thyroiditis and papillary thyroid carcinomas with or without coexisting thyroiditis

### Nadia Bagatian^1^, Ohoud Subhi^1^, Sajjad Karim^1,2^, Adel Al-Johari^3^, Osman Abdel Al-Hamour^4^, Abdulmonem Al-Mutawa^5^, Hosam Al-Aradati^5^, Faisal Al-Mashat^3^, Mohammad Al-Qahtani^1,2^, Hans-Juergen Schulten^1,2^, Jaudah Al-Maghrabi^5,6^

#### ^1^Center of Excellence in Genomic Medicine Research, King Abdulaziz University, Jeddah, Saudi Arabia; ^2^KACST Technology Innovation Center in Personalized Medicine, King Abdulaziz University, Jeddah, Saudi Arabia; ^3^Department of Surgery, Faculty of Medicine, King Abdulaziz University, Jeddah, Saudi Arabia; ^4^Department of Surgery, King Faisal Specialist Hospital and Research Center, Jeddah, Saudi Arabia; ^5^Department of Pathology, King Faisal Specialist Hospital and Research Center, Jeddah, Saudi Arabia; ^6^Department of Pathology, Faculty of Medicine, King Abdulaziz University, Jeddah, Saudi Arabia

##### **Correspondence:** Hans-Juergen Schulten (hschulten@kau.edu.sa) – Center of Excellence in Genomic Medicine Research, King Abdulaziz University, Jeddah, Saudi Arabia

**Background**

Chronic lymphocytic thyroiditis, also referred to as Hashimoto’s thyroiditis, is found in the background of about 30 % of papillary thyroid carcinomas (PTCs) [1]. The predisposition effect of this autoimmune condition is not thoroughly investigated on the transcriptional expression level.

**Materials and methods**

We analyzed retrospectively microarray expression profiles of 26 thyroid lesions including thyroiditis cases, and PTCs with or without coexisting thyroiditis. Normal/unaffected thyroid samples served as control. The processed samples were hybridized to HuGene 1.0 ST microarrays (Affymetrix, Inc., Santa Clara, CA). Sets of differentially expressed genes were generated on basis of a p-value ≤ 0.05 and a fold change > 2.

**Results**

More than 150 genes were differentially expressed between PTCs with coexisting thyroiditis and PTCs without coexisting thyroiditis and nearly 90 of these genes were also differentially expressed between thyroiditis cases and PTCs without coexisting thyroiditis. Comparably upregulated genes between the two PTC groups included nearly 50 immunoglobulin genes and comparably downregulated genes were, for example gamma-glutamylcyclotransferase (*GGCT*), and zinc finger, CCHC domain containing 16 (*ZCCHC16*). One of the genes that was not differentially expressed between thyroiditis and PTCs with coexisting thyroiditis was the bone marrow stromal cell antigen 2 (*BST2*).

**Conclusions**

This study detected a number of differentially expressed genes that are related to thyroiditis or to PTC with coexisting thyroiditis. For example, *BST2* was originally cloned from a rheumatoid-arthritis-derived synovial cell line and is known as a viral immune sensing molecule. BST2 overexpression in oral cavity cancer is known to be associated with nodal metastasis and poorer prognosis [2]. Furthermore, a fusion containing the extracellular domain of *BST2* exhibited anti-inflammatory and anti-remodelling effects in an experimental asthma model [3]. In conclusion, among the candidate genes for further investigation is for example *BST2* in order to elucidate its functions in cancer and autoimmune diseases.

**Acknowledgements**

This study was performed in support of the King Abdulaziz City for Science and Technology (KACST) grant 09-BIO2289-03.

**References**

1. Jankovic B, Le KT, Hershman JM: **Clinical Review: Hashimoto’s thyroiditis and papillary thyroid carcinoma: is there a correlation?***J Clin Endocrinol Metab* 2013, **98**(2):474-482.

2. Fang Fang KH, Kao HK, Chi LM, Liang Y, Liu SC, Hseuh C, Liao CT, Yen TC, Yu JS, Chang KP: **Overexpression of BST2 is associated with nodal metastasis and poorer prognosis in oral cavity cancer**. *The Laryngoscope* 2014, **124**(9):E354-360.

3. Herbert C, Shadie AM, Bunting MM, Tedla N, Garthwaite L, Freeman A, Yoo H, Park SH, Kumar RK: **Anti-inflammatory and anti-remodelling effects of ISU201, a modified form of the extracellular domain of human BST2, in experimental models of asthma: association with inhibition of histone acetylation**. *PloS one* 2014, **9**(3):e90436.

## P123 Metagenomic analysis of waste water microbiome in Sausdi Arabia

### Muhammad W shah^1^, Muhammad Yasir^2^, Esam I Azhar^2,3^, Saad Al-Masoodi^1^

#### ^1^Biology Department, Faculty of Science, King Abdulaziz University, Jeddah, Saudi Arabia; ^2^Special Infectious Agents Unit, King Fahd Medical Research Center, King Abdulaziz University, Jeddah, Saudi Arabia; ^3^Medical Laboratory Technology Department, Faculty of Applied Medical Sciences, King Abdulaziz University, Jeddah, Saudi Arabia

##### **Correspondence:** Muhammad Yasir (yasirkhattak.mrl@gmail.com) – Special Infectious Agents Unit, King Fahd Medical Research Center, King Abdulaziz University, Jeddah, Saudi Arabia

**Background**

The widespread use and misuses of antibiotics in both clinical and non clinical environments play vital role in mobilization, appearance and concentration of highly efficient resistance system in bacteria. For better understanding, surveillance studies of antimicrobial resistance is necessary to detect emerging resistances and to support management of infections in hospitals and other community sewage water pants.

**Materials and methods**

Samples of pre and post treated water were collected from King Abdulaziz Hospital and community waste water treatment plant Jeddah. DNA was extracted by *PowerMax® Soil* and *PowerWater® DNA Isolation kit.* The extracted DNA was purified by Gel Electrophoreses and then the samples were sequenced by using V3-V4 hyper-variable region of 16S rRNA gene (MiSeq Illumina, USA).

**Results**

From 16S rRNA gene sequence we obtain 1.5 million reads from 4 samples, and each sample was process in triplicate utilizing the amplicon sequencing (MiSeq Illumina). After sequence, processing and filtration, 1.48 million of high quality sequence reads (>200 bp) were obtained and assigned to bacteria domain with an average number of 123986.9 ± 49707.3 sequence reads per replicate per sample. Total 32 different phyla were identified. Phylum *Proteobacteria* was most dominant in both community (76 ± 0.55 %) and hospital waste (51.7 % ± 2.6). Density of *Proteobacteria* was significantly decreased to 23.5 % ± 1.3 and 40.8 % ± 2.3 after filtration in both samples. *Bacteroidetes* concentration was significantly increased from 21.9 ± 0.4 % to 70.8 ± 1.6 % and from 13.5 ± 1.4 % to 17.4 ± 3.6 % with filtration in community and in the hospital sewage respectively. Similarly, concentration of *Firmicutes* and Phylum *Actinobacteria* concentration was significantly increased with filtration in both samples. We analyzed that species specific richness, and 1651 OTUs were found at species taxonomic level classification in sequence reads. Few pathogenic i.e *Shigella sonnei*, *Vibrio cholera*, *Legionella pneumophila*, *Coxiella burnetii*, *Chlamydia pneumonia* and *Staphylococcus aureus* were present in low concentration.

**Conclusions**

The taxonomical diversity of bacterial species in waste water plants illustrates their resistance mechanism.

**Fig. 48 (abstract P123):**
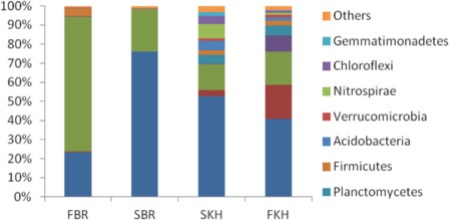
Percentage distribution of dominant phyla identified from 16S amplicon sequencing in waste water plants Jeddah

**Fig. 49 (abstract P123):**
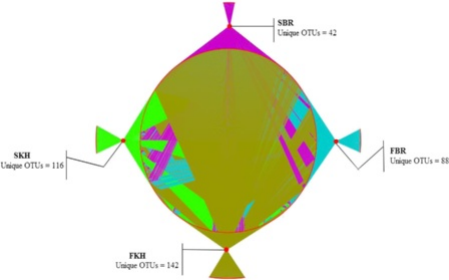
Network analysis of unique and shared level OTUs at special level of taxonomic classification among different samples collected from waste sewage plants

## P124 Molecular characterization of *Helicobacter pylori* from faecal samples of Tunisian patients with gastric cancer

### Yosr Z Haffani^1^, Msadok Azouz^1,2^, Emna Khamla^1^, Chaima Jlassi^1^, Ahmed S. Masmoudi^1^, Ameur Cherif^1^, Lassaad Belbahri^3^

#### ^1^Laboratory of Biotechnology and Valorization of Bio/Geo Resources LR11ES31, High Institute of Biotechnology Sidi Thabet, University of Manouba, Biotechpole Sidi Thabet, Sidi Thabet, Tunisia; ^2^Department of Gastroenterology, Hospital Mohamed Taher Maamouri, Nabeul, Tunisia; ^3^Laboratory of Soil Biology, University Neuchatel, Neuchatel, Swizerland

##### **Correspondence:** Yosr Z Haffani (yhaffani@gmail.com) – Laboratory of Biotechnology and Valorization of Bio/Geo Resources LR11ES31, High Institute of Biotechnology Sidi Thabet, University of Manouba, Biotechpole Sidi Thabet, Sidi Thabet, Tunisia

**Background**

*Helicobacter pylori,* a common bacterial pathogen of humans, infects gastric mucosa and is implicated in the etiology of several chronic diseases. Increasing evidence point *H. pylori* as class I carcinogen where chemical signals are secreted in the digestive tube. This molecular crosstalk generally promotes colonization by bacteria. This pathogen infects 3 billions people worldwide and can lead to severe diseases, including gastric ulcers and ultimately cancers. The secreted chemotaxic peptides help bacterial infection and function of atypical protein signal transduction system of *H. pylori*. The *H. pylori* species exhibit unusual high levels of genetic variation between strains. Interestingly, the genome of North African isolates of *H. pylori* has not yet been sequenced. North African isolates of *H. pylori* seems genetically different from the Asian isolates based on the susceptibility to affect the population with gastric cancer. Previous studies encountered difficulties to show the impact of *H. pylori* on gastric tumorigenesis and gastric microbiome due to low bacterial load in the stomach and sample availability.

**Material and methods**

To address the limitation highlighted above, we established a procedure to isolate metagenomic DNA from faecal samples and conducted PCR with *H. pylori* 16S rRNA specific primers followed by sequencing. Gastric mucosal biopsies were acquired from Tunisian patients in order to conduct anatomopathological examinations. All tissues used in this study were collected in order to examine the natural history of *H. pylori* infection in patients with and without gastric cancer.

**Results**

Ongoing results show individuals with gastric cancers are more prevalent among women under the age of 50. Gastric *H. pylori* infection is highly associated with diffuse pathological variant and adenocarcinoma are most often found in the gastric antrum of the stomach. Individuals with adenocarcinoma are classified poorly differentiated according to histology and location.

**Conclusions**

Our ongoing studies will unravel the extent of diversity of *H. pylori* populations possibly explaining why infection appears to be distinct across different samples. The application of cutting edge molecular technologies, mainly through whole genome sequencing, omic approaches and metabarcoding to the study of human associated pathogenic bacteria will make advances in our understanding of this field.

## P125 Diagnostic application of the oncoscan^©^ panel for the identification of hereditary cancer syndrome

### Shadi Al-Khayyat^1,2^, Roba Attas^3^, Atlal Abu-Sanad^1^, Mohammed Abuzinadah^2,4^, Adnan Merdad^1^, Ashraf Dallol^2^, Adeel Chaudhary^5^, Mohammed Al-Qahtani^5^, Adel Abuzenadah^2,4^

#### ^1^Faculty of Medicine, King Abdulaziz University, Jeddah, Saudi Arabia; ^2^Center of Innovation in Personalized Medicine, King Fahad Medical Research Center, King Abdulaziz University, Jeddah, Saudi Arabia; ^3^Faculty of Science, King Abdulaziz University, Jeddah, Saudi Arabia; ^4^Faculty of Applied Medical Sciences, King Abdulaziz University, Jeddah, Saudi Arabia; ^5^Centre of Excellence in Genomic Medicine Research, King Abdulaziz University, Jeddah, Saudi Arabia

##### **Correspondence:** Adel Abuzenadah (adel_abuzenadah@hotmail.com) – Center of Innovation in Personalized Medicine, King Fahad Medical Research Center, King Abdulaziz University, Jeddah, Saudi Arabia

**Background**

Cancer syndrome is considered a genetic disorder in which inherited DNA polymorphisms in one or more genes make the individual susceptible to cancer development. It has been estimated that familial cancer syndromes comprises 5 to 10 percent of all types of cancer. In addition to the high risk of developing cancer, cancer syndromes often lead to the development of various independent primary tumours. These syndromes are usually caused by mutations in tumour suppressor genes, oncogenes and DNA repair genes. The very common examples of cancer syndromes are the hereditary breast-ovarian cancer syndrome and hereditary non-polyposis colon cancer (Lynch syndrome).

**Materials and methods**

The advent of Next Generation Sequencing has resulted in an era of high throughput DNA sequencing, which has a major influence in both clinical care and cancer research. In particular, targeted resequencing is an efficient approach for mutation detection at a low cost and high turnover. Taking the importance of NGS in diagnosing inherited mutations of multiple genes, we have designed the Oncoscan panel which will allow the simultaneous screening of 74 cancer genes using the Ampliseq™ technology.

**Results**

The oncoscan panel is covering 95.22 % of 74 targeted genes with 1626 amplicon, size ranging between 125 to 175 bp and generating 192.96 kb of DNA sequence. Our results show that the panel is useful in identifying heritable susceptibility to several forms of cancer including breast and colon cancer. Additionally, we have identified novel mutations in familial breast cancer cases affecting DNA repair pathways other than BRCA1 and BRCA2.

**Conclusions**

Importantly, we suggest that the panel is also useful in identifying Lynch syndrome cases in which female patients manifest breast cancer as well as colon cancer and other tumours.

## P126 Characterization of clinical and neurocognitive features in a family with a novel *OGT* gene missense mutation c. 1193G > A/ (p. Ala319Thr)

### Habib Bouazzi^1^, Carlos Trujillo^2^, Mohammad Khalid Alwasiyah^3,4^, Mohammed Al-Qahtani^4^

#### ^1^Hôpital Necker-Enfants Malades-Université Paris descartes-Laboratoire de génétique, Paris, France; ^2^Erfan & Bagedo Hospital, Jeddah, Saudi Arabia; ^3^Aziziah Maternity and Children Hospital, Jeddah, Saudi Arabia; ^4^Center of Excellence in Genomic Medicine Research (CEGMR), King Abdullaziz University, Jeddah, Saudi Arabia

##### **Correspondence:** Habib Bouazzi (Habib.bouazzi@etu.parisdescartes.fr) – Hôpital Necker-Enfants Malades-Université Paris descartes-Laboratoire de génétique, Paris, Franaces

**Background**

X-linked intellectual disability (XLID) is a heterogeneous disorder for which many of the causative genes are still unknown [1]. So far, more than one hundred genes of the X chromosome have been associated with intellectual disability (ID). O-linked N-acetyl-Glucosamine-Transferase (*OGT)* gene is well known to be involved in endocrine alterations by the resistance of insulin in muscles and adipocytes and therefore the initiation of diabetes. It is reported to be involved also in cancer, brain development, and neurodegenerative diseases [2]. We performed X-exome sequencing in three brothers with non-syndromic XLID and developmental delay. Sanger sequencing was accomplished to confirm novel mutations. X-chromosome inactivation was executed in the mother. Affected boys had a severe ID and mild dysmorphic features. The heterozygous mother had mild cognitive impairment. Her X-chromosome inactivation pattern was not skewed. We identified a novel missense mutation (c. 1193G > A) in the *OGT* gene. This mutation was inherited by the affected males, and segregated with the abnormal phenotype.

**Rusults**

A single missense mutation (c.1193G > A) was identified by X-exome sequencing of two patients, according to our filters this mutation is considered to be pathogenic. This mutation is absent from public databases of control individuals. Sanger sequencing was performed to confirm this novel mutation in all three affected boys as well as in the mother, also the unaffected brother X-exome sequencing revealed no mutation. This substitution is predicted to be deleterious by SIFT software (score: 0) and polyphen score was one which considered to be damaging. In addition by Mutation Taster, a disease-causing variant was predicted, scoring a p-value of one.

**Conclusions**

Effect of *OGT* alteration of brain development has been confirmed, nevertheless so far this gene has not been attested to be related to ID. The mutation within *OGT* segregating in all affected males, the phenotype of our patients could be linked to the new missense mutation of the *OGT* gene nevertheless, our single case cannot be generalized and despite evidence of *OGT* gene effect on neuronal physiology and brain development, more studies with additional cases are warranted to shed light on the cognitive role of the *OGT* gene.

**References**

1. H. Bouazzi, G. Leska, C. Trujillo, M. K. Alwasiyah, and A. Munnich. **Nonsyndromic X-linked intellectual deficiency in three brothers with a novel*MED12*missense mutation [c.5922G&gt;T (p.Glu1974His)].***Clin. Case Rep.*, p. n/a–n/a, May 2015.

2. Y. Liu, X. Li, Y. Yu, J. Shi, Z. Liang, X. Run, Y. Li, C. Dai, I. Grundke-Iqbal, K. Iqbal, F. Liu, and C.-X. Gong, **Developmental Regulation of Protein O-GlcNAcylation, O-GlcNAc Transferase, and O-GlcNAcase in Mammalian Brain**. *PLoS ONE*, vol. 7, no. 8, p. e43724, Aug. 2012.

## P127 Case report: a rare homozygous deletion mutation of *TMEM70* gene associated with 3-Methylglutaconic Aciduria and cataract in a Saudi patient

### Maha Alotaibi^1^, Rami Nassir^2^

#### ^1^Department of Clinical Genetic and Metabolic Genetics, King Saud Medical Hospital, Riyadh, Saudi Arabia; ^2^Department of Pathology, School of Medicine, Umm Al-Qura University, Mecca, Saudi Arabia

##### **Correspondence:** Rami Nassir (rmnassir@gmail.com) – Department of Pathology, School of Medicine, Umm Al-Qura University, Mecca, Saudi Arabia

**Background**

Lately, there is an increase in the number of the patients with nuclear genetic defects of the mitochondrial ATP synthase. The *TMEM70* gene mutation is one of the most common nuclear encoded genes that affect the ATP synthase. Here, we report a 9-month-old Saudi girl presenting with lactic acidosis, 3-Methylglutaconic aciduria, cataract, hypertrophic cardiomyopathy and encephalopathy. The patient was genetically tested for Methylglutaconic Aciduria Nuclear Gene panel/sequencing and deletion/duplication analysis.

**Results**

She was positive for homozygous deletion of c.578_579 delCA in exon 3 of the *TMEM70* gene.

**Conclusions**

This is consistent with a diagnosis of ATP synthase deficiency. This case report hopefully helps in the diagnosis of future cases, providing important information regarding diagnosis and prognosis and as well as optimal managements for such cases.

**Consent to publish**

Written informed consent for publication of their clinical details and/or clinical images was obtained from the patient/parent/guardian/relative of the patient. A copy of the consent form is available for review by the Editor of this journal.

## P128 Isolation and purification of antimicrobial milk proteins

### Ishfaq A Sheikh^1^, Mohammad A Kamal^1^, Essam H Jiffri^2^, Ghulam M Ashraf^1^, Mohd A Beg^1^

#### ^1^King Fahd Medical Research Center, King Abdulaziz University, Jeddah, Saudi Arabia; ^2^Faculty of Applied Medical Sciences, King Abdulaziz University, Jeddah, Saudi Arabia

##### **Correspondence:** Ishfaq A Sheikh (sheikhishfaq@gmail.com) – King Fahd Medical Research Center, King Abdulaziz University, Jeddah, Saudi Arabia

**Background**

Milk is a specific diet of mammalian neonates. Besides its nutritional value, milk is rich in antimicrobial proteins, immunoglobulins, growth factors and plays a key role in enhancing immune system of infants. Milk-derived proteins and peptides play a significant role in the prevention and treatment of various human metabolic disorders and have significant therapeutic importance. These include lactoperoxidase, lactoferrin, peptidoglycan recognition protein etc. Lactoperoxidase, one of the essential constituents of milk plays a significant role during the early stages of neonatal life. In addition to improving the immune system of infants, lactoperoxidase also has antimicrobial activity. Lactoperoxidase has huge industrial applications and is commonly known as lactoperoxidase system. The system is well-established and commonly adopted in industries for preventing microbial growth. Similarly, lactoferrin and peptidoglycan recognition proteins also exhibit antimicrobial activity and are of immense medical importance. The aim of the current study is to purify antimicrobial protein from camel milk.

**Materials and methods**

Milk was purchased from the local camel farms in Jeddah. Milk was processed to separate the fats by centrifugation (skimming) at 1500 g for 10 min. The fat free milk was subjected to a linear gradient of 0.0 M-0.5 M NaCl in 50 mM Tris-HCl at pH 7.8.

**Results**

The acidic and basic proteins from camel milk were separated. We are currently making attempts to purify different fractions of proteins from camel milk.

**Conclusions**

Camel is the most important agricultural animal in the Saudi Kingdom. Camel milk derived constituents are of tremendous potential industrial applications. These proteins could be explored for medical applications like drug designing.

**Acknowledgements**

This project was funded by the National Plan for Science, Technology and Innovation (MAARIFAH) – King Abdulaziz City for Science and Technology - the Kingdom of Saudi Arabia – award number (12-BIO3082-03). The authors also acknowledge with thanks Science and Technology Unit, King Abdulaziz University for technical support.

## P129 Integrated analysis reveals association of ATP8B1 gene with colorectal cancer

### Mohammad A Aziz^1^, Rizwan Ali^1^, Mahmood Rasool^2^, Mohammad S Jamal^3^, Nusaibah samman^1^, Ghufrana Abdussami^4^, Sathish Periyasamy^1^, Mohiuddin K Warsi^5^, Mohammed Aldress^1^, Majed Al Otaibi^1^, Zeyad Al Yousef^1^, Mohamed Boudjelal^1^, Abdelbasit Buhmeida^2^, Mohammed H Al-Qahtani^2^, Ibrahim AlAbdulkarim^1^

#### ^1^King Abdullah International Medical Research Center/King Saud Bin Abdulaziz University for Health Sciences, Ministry of National Guard Health Affairs, Riyad, Saudi Arabia; ^2^Center of Excellence in Genomic Medicine Research, King Abdulaziz University, Jeddah, Saudi Arabia; ^3^King Fahd Medical Research Center, King Abdulaziz University, Jeddah, Saudi Arabia; ^4^Department of Biosciences, Jamia Milia Islamia, New Delhi, India; ^5^Mohammad Ali Jauhar University, Rampur, UP, India

##### **Correspondence:** Mohammad A Aziz (azizmo@ngha.med.sa) – King Abdullah International Medical Research Center/King Saud Bin Abdulaziz University for Health Sciences, Ministry of National Guard Health Affairs, Riyad, Saudi Arabia

**Background**

Colorectal cancer is one of the most prevalent cancers in the world population and its incidents are increasing every year. Numerous cutting edge approaches are being applied to find the basis underlying this lethal disorder [1]. Integrative analysis of multiple –omics is promising to provide new biomarkers and targets for cancer.

**Materials and methods**

We carried out integrated analysis of cytogenetic and exon array data using colorectal cancer patient samples.

**Results**

Patient wise tumor-normal comparison at cytogenetic level yielded a high priority list of 144 driver genes. Of these, 11 genes were found to be novel in their association with colorectal cancer. We analyzed these genes at exon level and found ATP8B1 to be significantly altered in expression. At cytogenetic level, ATP8B1 had a GISTIC score of 2.326 and was found in the region of heavy loss. ATP8B1 showed significant fold changes at the gene and exon level. It was downregulated by more than two fold at gene level (with a p value <0.01). ATP8B1 had no prior knowledge of association with colorectal cancer as inferred from Ingenuity Pathway analysis. Further characterization of ATP8B1 and associated genes is underway to get better insights into its functional aspect and eventual use as a biomarker for colorectal cancer.

**Conclusions**

In conclusion we have first time reported association of ATP8B1 gene associated with colorectal cancer using integrative analysis of multiple –omics technique.

**References**

1. Schmoll HJ, Van Cutsem E, Stein A, Valentini V, Glimelius B, Haustermans K, Nordlinger B, van de Velde CJ, Balmana J, Regula J, et al. **ESMO Consensus Guidelines for management of patients with colon and rectal cancer. A personalized approach to clinical decision making.** Ann Oncol. 2012, 23: 2479–2516.

## P130 Implication of IL-10 and IL-28 polymorphism with successful anti-HCV therapy and viral clearance

### Rubi Ghazala^1^, Shilu Mathew^2^, M. Haroon Hamed^3^, Mourad Assidi^2,4^, Mohammed Al-Qahtani^2^, Ishtiaq Qadri^5^

#### ^1^School of Medicine, University of Health Science, Lahore, Pakistan; ^2^Center of Excellence in Genomic Medicine Research, King Abdulaziz University, Jeddah, Saudi Arabia; ^3^Department of Biology, King Abdul-Aziz University, Jeddah, Saudi Arabia; ^4^Center of Innovation in Personalized Medicine at King Abdulaziz University, Jeddah, Saudi Arabia; ^5^King Fahd Medical Research Center, King Abdul Aziz University, Jeddah, Saudi Arabia

##### **Correspondence:** Rubi Ghazala (ishtiaq80262@yahoo.com) – School of Medicine, University of Health Science, Lahore, Pakistan; Ishtiaq Qadri (ishtiaq80262@yahoo.com) – King Fahd Medical Research Center, King Abdul Aziz University, Jeddah, Saudi Arabia

**Background**

Hepatitis C virus the main reason of chronic liver ailment and liver cancer globally and is distributed into six discrete genotypes through the world with numerous subtypes in each genotype (1-6). The response and extent of interferon treatment is genotype specific and host restriction. Number of cellular genes are involved in this process including TBXA2R (G-protein coupled receptor), TRAF2 (adapter protein), LTbeta which is a membrane protein, NFkappaB2 and RelA (transcriptional factors), SNARK and MKK7 (protein kinases) and two diligently associated TNF/lymphotoxin pathway. A reported polymorphisms (SNPs) in these and other genetic factor are involved in viral clearance and chornicity. Genetic studies have also identified several SNPs round the interferon λ3 interleukin-28B, which are sturdily related with SVR to PEG-IFN and RBV cure for chronicity. In this study we examined SNP in IL10 and IL28B in HCV-GT3 infected individuals.

**Materials and methods**

A total of 349 patients of chronic hepatitis have been included in this genetic susceptibility study. The infected individuals were diagnosed anti-HCV positive and then confirmed by HCV Polymerized Chain Reaction (PCR) qualitative test. All these selected patients had been diagnosed for HCV and taken the standard treatment of interferon and ribavirin from 20 weeks to 36 weeks. The end product of PCR was confirmed by gel electrophoresis. The following restriction enzymes Ear I/, Rsa 1 and Mae III were used to digest the PCR product. This study focused only on patients who were non-responsive and had relapsed from conventional therapy.

**Results**

Our study proposes that both the interleukin genes interleukin 28B and IL-10 are found mutual in 2 foremost castes of the province Punjab. We determined that HCV GT-3a is precisely communal (84.0 %) amongst group of responders whereas GT-1a is more acquainted in relapser (66.2 %) as well as resistant (54.0 %). Genotype 4was excluded, the remaining 5 major genotypes, known as 1a (61.40 %), 2a (0.50 %), 2b (20.00 %), 3a (13.70 %) and an unidentified (4.40 %) were reported amongst the twelve various groups of Pakistan. Our findings designate that IL-10 and IL-28 genes may be intricate in the clearance of HCV GT3 in Asian population.

**References**

1. Mathew, S. *et al.****In silico*studies of medicinal compounds against hepatitis C capsid protein from north India**. *Bioinform Biol Insights***8**, 159-68 (2014).

2. http://www.hepatitiscentral.com/hepatitis-c/hepatitis-c-genotypes/.

3. Mathew, S. *et al.***Biomarkers for virus-induced hepatocellular carcinoma (HCC)**. *Infect Genet Evol***26**, 327-39 (2014).

4. Fatima, K. *et al.***Docking studies of Pakistani HCV NS3 helicase: a possible antiviral drug target**. *PLoS One***9**, e106339 (2014).

5. Mathew, S. *et al.***Computational Docking Study of p7 Ion Channel from HCV Genotype 3 and Genotype 4 and Its Interaction with Natural Compound**s. *PLoS One***10**, e0126510 (2015).

## P131 Interactions of endocrine disruptor di-(2-ethylhexyl) phthalate (DEHP) and its metabolite mono-2-ethylhexyl phthalate (MEHP) with progesterone receptor

### Ishfaq A Sheikh^1^, Muhammad Abu-Elmagd^2,3^, Rola F Turki^3,4^, Ghazi A Damanhouri^1^, Mohd A. Beg^1^

#### ^1^King Fahd Medical Research Center, King Abdulaziz University, Jeddah, Kingdom of Saudi Arabia; ^2^Centre of Excellence in Genomic Medicine Research, King Abdulaziz University, Jeddah, Kingdom of Saudi Arabia; ^3^KACST Technology Innovation Center in Personalized Medicine, King Abdulaziz University, Jeddah, Kingdom of Saudi Arabia; ^4^Department of Obstetrics and Gynecology, King Abdulaziz University Hospital, Jeddah, Kingdom of Saudi Arabia

##### **Correspondence:** Mohd A. Beg (mabeg51@gmail.com) – King Fahd Medical Research Center, King Abdulaziz University, Jeddah, Kingdom of Saudi Arabias

**Background**

Endocrine disrupting chemicals (EDCs) contaminating our ecosystems are increasingly shown to impact the reproductive health in human population. Di-(2-ethylhexyl)phthalate (DEHP) is a high production volume EDC used as a plasticizer in personal and industrial products such as dolls, toys, medical tubing, floor tiles, upholstery, food packaging plastics, cosmetics etc. Constant leaching from these products leads to the ubiquitous presence of DEHP in the environment. Clinico-epidemiological reports have incriminated DEHP and its metabolites in reproductive developmental abnormalities in neonates, endometriosis and miscarriage in women, and abnormal spermiogram in men. Experimental studies in rats also induce similar developmental and reproductive abnormalities. Many EDCs have agonistic and antagonistic interactions with the steroid receptors. Binding of DEHP and its metabolites to progesterone receptor (PR) represents a potential interfering mechanism for the steroid target tissues. Progesterone plays a central role in diverse reproductive events including ovarian function and pregnancy. We present here a study on the structural binding of DEHP and its metabolite mono (2-ethylhexyl)phthalate (MEHP) with PR using *in silico* approaches.

**Materials and methods**

The crystal structure of human PR (Id: 1SQN) was retrieved from Protein Data Bank as a template for docking DEHP and MEHP. The structures of DEHP and MEHP were obtained from PubChem database. Docking of DEHP and MEHP into the binding site of PR was performed using AutoDock 4.2 with Lamarckian Genetic Algorithm to predict the bound conformation. The interacting residues for the ligand-receptor binding were visualized using Chimera.

**Results**

Docking of DEHP and MEHP with PR displayed good binding affinity values and exhibited both hydrophobic and hydrophilic interactions. The PR residues involved in hydrogen bonding interactions were Glutamine-725 and Arg-766, and Phe-778 made Pi-Pi interactions with aromatic ring in both ligands. Altogether 22 residues contributed in hydrophobic interactions in ligand-receptor complex.

**Conclusions**

The study suggested that DEHP and its metabolite, MEHP occupy important residues in interactions with PR. These interactions apparently compete with progesterone for PR binding sites and, hence, likely disrupt the progesterone signaling resulting in abnormal reproductive function. Current study enhances our understanding of underlying molecular mechanisms of endocrine disrupting activity of DEHP and its metabolites.

**Acknowledgements**

This project was funded by the National Plan for Science, Technology and Innovation (MAARIFAH) – King Abdulaziz City for Science and Technology - the Kingdom of Saudi Arabia – award number (12-BIO3082-03). The authors also, acknowledge with thanks Science and Technology Unit, King Abdulaziz University for technical support.

## P132 Association of HCV nucleotide polymorphism in the development of hepatocellular carcinoma

### Mohd Suhail^1^, Abid Qureshi^2^, Adil Jamal^3^, Peter Natesan Pushparaj^4^, Mohammad Al-Qahtani^4^, Ishtiaq Qadri^1^

#### ^1^Medical Biotechnology and Translational Medicine Research, King Fahd Medical Research Center, King Abdulaziz University, PO Box 80216 Jeddah 21589, Saudi Arabia; ^2^Institute of Microbial Technology (IMTECH), Chandigarh 160036, India; ^3^Ummul Qura University, Makkah Saudi Arabia; ^4^Center of Excellence in Genomic Medicine Research, King Abdulaziz University, P.O. Box 80216, Jeddah 21589, Saudi Arabia

##### **Correspondence:** Ishtiaq Qadri (ishtiaq80262@yahoo.com) – Medical Biotechnology and Translational Medicine Research, King Fahd Medical Research Center, King Abdulaziz University, PO Box 80216 Jeddah 21589, Saudi Arabia

**Background**

Hepatitis C virus (HCV) infection presents a significant health problem worldwide. In the Kingdom of Saudi Arabia (KSA), liver cancer is a major problem and there is an increase in the prevalence of viral induced cirrhosis. Based on genetic differences of HCV isolates, it is classified into at least 7 genotypes (1–7) with many additional subtypes within each genotype and GT-4 is predominant in KSA. Single Nucleotide polymorphism (SNP) are most common cause of variation in viral and host genome and linked with specific disease subjected to known sequence specific polymorphic sites (mutations) in genomes that trigger mutation and disease progression. Several SNPs also contribute in the HCV infection outcomes. During HCC development, mutations are scored with in the Core, NS4B, NS5B and hyper variable region of E2 which are somehow associated with lack of strong inflammatory immune response.

**Materials and methods**

Guanosine Triphosphatase (GT) activity encoded in NS4B protein’s nucleotide binding motif (NBM) serves as single nucleotide variable in the evolution process of HCV and associated viral life cycle. The transforming activity of NS4B from HCV GT4 was analyzed using the mutants. Phylogenetic data on clustering of subtypes for E1 & NS5B genes was performed to identify the variation in viral genomes.

**Results**

The pathogenicity was progressively inhibited and completely abrogated by increasing genetic impairment with the nucleotide binding motif (NBM) of NS4B. This transformation ability of NS4B in NIH3TC cells was independent of co-transfection with activated H-ras. Phylogenetic data on clustering of subtypes for E1 & NS5B genes revealed that genome types (1-6) differ around 30-35 % while the subtypes differ only about 20-25 %.

**Conclusions**

HCV GT4 encoded NS4B has both *in vitro* and *in vivo* tumorigenic potential and this transforming activity is mediated by its NBM as single nucleotide variable factor. Moreover, results suggest that pharmacological inhibition of this motif may be exploited to inhibit HCV replication but also the associated hepatocellular carcinoma (HCC). Further studies should help in the development of non-invasive techniques for evaluating the NBM distribution, patient’s condition and the risk of HCC development.

## P133 Gene expression profiling by DNA microarrays in colon cancer treated with chelidonine alkaloid

### Mahmoud Z El-Readi^1,2^, Safaa Y Eid^1,3^, Michael Wink^3^

#### ^1^Department of Biochemistry, Faculty of Medicine, Umm Al-Qura University, Makkah, KSA; ^2^Department of Biochemistry, Faculty of Pharmacy, Al-Azhar University, 71524 Assiut, Egypt; ^3^Institute of Pharmacy and Molecular Biotechnology, Heidelberg University, Im Neuenheimer Feld 364, 69120 Heidelberg, Germany

##### **Correspondence:** Mahmoud Z El-Readi (mzreadi@uqu.edu.sa) – Department of Biochemistry, Faculty of Medicine, Umm Al-Qura University, Makkah, KSA

**Background**

Microarray studies play an important role in identification and classification the genes whose expressions are impacted by chemotherapies for colorectal cancer. Therefore, the application of gene expression profiling using microarray of DNA could potentially use to predict the response to treatments. The anticancer mechanisms of chelidonine (alkaloid) were tested on colon cancer using Caco-2 cell line.

**Materials and methods**

Caco2 cells were treated with 20 μM chelidonine for 48 h. RNA isolation and cDNA synthesis, hybridization, data quality assessment, filtering, normalization, and subsequent analysis were performed by procedure that meet or exceed the MIAME-criteria of microarray analysis [1]. The numbers of significantly up and down regulated genes in treated Caco-2 cell were subjected to correspondence cluster analysis [2]. Among the differentially expressed genes we selected highly *P-*value genes for Ingenuity Pathway Analysis Knowledge database (version 6.5) (www.ingenuity.com) to identify the biological responses of treated colon adenocarcinoma.

**Results**

**Fig. 50 (abstract P133):**
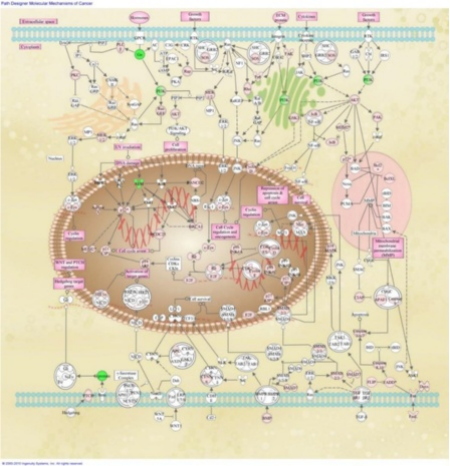
The cancer molecular mechanisms in Caco-2 cells after treatment with 20 μM chelidonine for 48 hr. Red colored for up-regulated genes, green for down-regulated genes

The top 10 networks of the molecules that have impact on the diversity of biological functions in treated colon cancer with chelidonine are summarized in Table 24. These molecules have an important role in modulation of cancer, cell cycle, cell death, cellular proliferation, and cellular function. Biomarker Analysis shows that chelidonine able to modulate many biomarkers that involved in molecular mechanism of cancer, apoptotic signaling, and PXR signaling (Fig. 50).

**Conclusions**

Our results provide new insights into chelidonine-related signaling activities, which may facilitate the development of chelidonine-based anticancer strategies and/or combination therapies.

**References**

1. Brazma A, Hingamp P, Quackenbush J, Sherlock G, Spellman P, Stoeckert C, et al.: **Minimum information about a microarray experiment (MIAME) - toward standards for microarray data**. *Nature Genetics*. 2001;**29**(4):365-71.

2. Fellenberg K, Hauser NC, Brors B, Neutzner A, Hoheisel JD, Vingron M.: **Correspondence analysis applied to microarray data.***Proceedings of the National Academy of Sciences of the United States of America.* 2001;**98**(19):10781-6.

**Table 24 (abstract P133):** The top 10 networks associated with the functions affected upon treatment of Caco-2 cells with 20 μM chelidonine for 48 hr

Network	Score	Focus Molecules	Top Functions
1	37	28	Cancer, Cellular Development, Cellular Growth, and Proliferation
2	36	27	Cell Death, Cellular Assembly and Organization, Cellular Function, and Maintenance
3	33	26	Cellular Development, Embryonic Development, Cellular Growth, and Proliferation
4	30	24	Cell Cycle, Embryonic Development, Connective Tissue Development, and Function
5	29	24	DNA Replication, Recombination, and Repair, Cancer, and Genetic Disorder
6	27	23	Cancer, Hematological Disease, Cellular Function and Maintenance
7	26	24	Cell Death, Cell Cycle, and Cancer
8	26	22	Cell Morphology, Cellular Assembly and Organization, Nervous System Development and Function
9	26	22	Cellular Compromise, Cellular Function and Maintenance, Energy Production
10	25	25	Cancer, Hematological Disease, Immunological Disease

## P134 Successful *in vitro* fertilization after eight failed trials

### Ahmed M. Isa^1^, Lulu Alnuaim^1^, Johara Almutawa^1^, Basim Abu-Rafae^1^, Saleh Alasiri^1^, Saleh Binsaleh^2^

#### ^1^Assisted Conception Unit, Obstetrics and Gynecology Department, College of Medicine, King Saud University, Riyadh, Kingdom of Saudi Arabia; ^2^Urology Division, Surgery Department, College of Medicine, King Saud University, Riyadh, Kingdom of Saudi Arabia

##### **Correspondence:** Ahmed M. Isa (isaahmed@hotmail.com) – Assisted Conception Unit, Obstetrics and Gynecology Department, College of Medicine, King Saud University, Riyadh, Kingdom of Saudi Arabia

**Background**

Many infertile couples worldwide resort to donated gametes to overcome their infertility problems, however many others cannot for cultural or religious reasons. They have no other options but probably to keep trying In Vitro Fertilization (IVF) treatment using their own gametes. We wanted to see if we would be able at some point to help such cases fulfil their dreams.

**Subjects and methods**

Four infertility cases with a history of many unsuccessful IVF trials; eight, seven, six, and four, respectfully; suggestively because of very poor fertilization and/or poor embryo development due to sever male factor that ranged from azoospermia to severe oligoteratoasthenozoospermia. A short ovarian stimulation (OS) protocol was employed with all cases. Follicles growth was monitored by ultrasound examinations until the eggs were retrieved. The cumulus oocyte complexes (COCs) were incubated in culture medium then denuded before the intracytoplasmic sperm injection (ICSI). Normally fertilized eggs were kept in culturing conditions till the embryos were transferred to each patient’s uterus. Fourteen days later, the patient’s blood serum hCG was analyzed for pregnancy. Six weeks later the ultrasound exam was done for detecting the viable gestational sacs. Informed consents were collected from all patients prior to any data collection.

**Results**

All four cases were finally successful with normal live births in three cases and a 16-week ongoing pregnancy in the fourth case.

**Conclusions**

In some complex infertility cases, when the use of donor-gametes is not optional, probably hopelessness in IVF treatment with autologous gametes is not an option as well.

**Acknowledgements**

The author would like to appreciate the College of Medicine Research Center, the Deanship of Scientific Research, King Saud University, for supporting this work and its authors. The authors would like to thank all the IVF team of the Assisted Conception Unit at King Khalid University Hospital in Saudi Arabia for their dedication and professionalism in serving our patients.

**References**

1. De Lauretis L., Scarduelli C., Bailo U. et al. (1994). **IVF in natural cycle: our experience**. *Hum. Reprod*. **9** (Suppl. 4), 131.

2. Kawwass JF, Mansour M, Crawford S, Kissin DM, Session DR, Kulkami AD, Jamieson DJ (2013). **Trends and outcomes for donor cycles in the United States**, 2000-2010. *JAMA* Dec. **11**; 310(22): 2426-2434.

3. Janssens RMJ, Lambalk CB, Schats R, Schoemaker J (1999). **Successful in-vitro fertilization in a natural cycle after four previously failed attempts in stimulated cycles**. *Hum. Reprod*., **14**(10): 2497-2498.

## P135 Genetic sensitivity analysis using SCGE, cell cycle and mitochondrial membrane potential in OPs stressed leukocytes in *Rattus norvegicus* through flow cytometric input

### Nazia Nazam^1^, Mohamad I Lone^1^, Waseem Ahmad^2^, Shakeel A Ansari^2^, Mohamed H Alqahtani^2^

#### ^1^Toxicogenomics Laboratory, Division of Genetics, Department of Zoology, Aligarh Muslim University, Aligarh, India; ^2^Centre of Excellence in Genomic and Medicine Research, King Abdulaziz University, Jeddah, Saudi Arabia

##### **Correspondence:** Waseem Ahmad (waseemahmad2007@hotmail.com) – Toxicogenomics Laboratory, Division of Genetics, Department of Zoology, Aligarh Muslim University, Aligarh, India

**Background**

Oxidative damage to DNA and other biological processes can be disrupted by ROS. The organophosphates are known to produce oxidative stress by inhibiting enzymes and antioxidants. Incorporating flow cytometry in assessing DNA damage, cell cycle, mitochondrial membrane potential and apoptosis due to endogenous cysteine proteases- get credence.

**Materials and methods**

Detection of DNA strand break was performed by alkaline comet assay involving; single cell suspension, lysis, electrophoresis, image analysis and comet measurements. Detection of apoptosis and analysis of cell cycle used leukocyte cells; involving RNase digestion after incubation by propidium iodide and analyzed on flow cytometer. The fluorescence intensity of SubG_1_ cell fraction gave clue for apoptotic cell population. Number of events were scored for cell cycle analysis. Flow cytometric measurements were facilitated by Rh 123 dye in ΔΨm study. Two OPs - dichlorovos (DCV) and dimethoate (DM) were explored using standard protocol*.*

**Results**

Digitized comet images showed nuclei with increasing degree of DNA damage in exposed cells. The mean value of frequencies of olive tail moments and tail lengths indicated genotoxicity. Image analysis and comet measure results show nuclei with damage. There was a statistically significant increase by DCV. DM, also inflicted highly significant DNA damage. The single cell DNA not repaired, owing to excessive ROS production. The subG_1_ cells provided clue for cells undergoing apoptosis/necrosis- increased considerably. Comet images showed damage to nucleus. Cell cycle analysis confirmed decreased G2/M cell cycle arrest. The conspicuous ΔΨm loss, was a cellular event in the mitochondrial driven apoptosis.

**Conclusions**

Results contribute to understanding the acute exposition of OPs on cell survival and carcinogenicity. Clinical interest in evaluating the damage to humans, involuntarily exposed to chemical stressors, is further vindicated.

